# Prediction and characterization of protein-protein interaction network in *Bacillus licheniformis* WX-02

**DOI:** 10.1038/srep19486

**Published:** 2016-01-19

**Authors:** Yi-Chao Han, Jia-Ming Song, Long Wang, Cheng-Cheng Shu, Jing Guo, Ling-Ling Chen

**Affiliations:** 1 College of Informatics, Agricultural Bioinformatics Key Laboratory of Hubei Province, Huazhong Agricultural University, Wuhan 430070, P.R. China

## Abstract

In this study, we constructed a protein-protein interaction (PPI) network of *B. licheniformis* strain WX-02 with interolog method and domain-based method, which contained 15,864 edges and 2,448 nodes. Although computationally predicted networks have relatively low coverage and high false-positive rate, our prediction was confirmed from three perspectives: local structural features, functional similarities and transcriptional correlations. Further analysis of the COG heat map showed that protein interactions in *B. licheniformis* WX-02 mainly occurred in the same functional categories. By incorporating the transcriptome data, we found that the topological properties of the PPI network were robust under normal and high salt conditions. In addition, 267 different protein complexes were identified and 117 poorly characterized proteins were annotated with certain functions based on the PPI network. Furthermore, the sub-network showed that a hub protein CcpA jointed directly or indirectly many proteins related to γ-PGA synthesis and regulation, such as PgsB, GltA, GltB, ProB, ProJ, YcgM and two signal transduction systems ComP-ComA and DegS-DegU. Thus, CcpA might play an important role in the regulation of γ-PGA synthesis. This study therefore will facilitate the understanding of the complex cellular behaviors and mechanisms of γ-PGA synthesis in *B. licheniformis* WX-02.

*Bacillus licheniformis* (*B. licheniformis*) is a gram-positive spore-forming bacterium widely used in industry and agriculture^1^. For example, it can be used to produce many commercial enzymes^2^, biofuels and chemicals by fermentation, including poly-gamma-glutamic acid (γ-PGA)^3^, acetoin^4^ and antibiotics^5^, and even can be directly used to convert plumage into nutritious food for livestock^6^. Currently, the studies of *B. licheniformis* are mainly focused on one specific protein or several proteins in a single pathway^7^,^8^,^9^,^10^, while no comprehensive protein-protein interaction (PPI) network has been reported.

Proteins seldom perform their biological functions independently, and most complex cellular processes must be understood via large-scale PPI networks^11^,^12^. The availability of *B. licheniformis* strain WX-02 genome makes it possible to perform genome-scale analysis based on PPI network^13^,^14^. Genome-wide PPI networks have become powerful tools to study the cellular behaviors with a global view, and they can reveal the relationships between different kinds of proteins with various functions. Proteins involved in important biological processes and controlling the entire network can also be detected with the organization of the interactome^11^,^15^,^16^. In addition, the constructed PPI network is conducive to elucidating some protein functions that are poorly characterized with genome annotation^17^,^18^.

Currently, a large number of PPI networks have been constructed with high-throughput experimental methods, such as yeast two-hybrid system and tandem affinity purification^19^. However, these methods are quite costly in time and money^20^,^21^. With the increasing number of experimentally-determined PPIs and 3D-structures of proteins, a series of computational methods have been developed and attracted researchers by economical, rapid and convenient characters. In this study, we predicted the PPI network of *B. licheniformis* WX-02 by using two independent computational methods (interolog method and domain-based method) and analyzed the network from different perspectives. Finally, a PPI network containing 15,864 edges and 2,448 nodes was obtained. Based on this network, we investigated some species-specific properties of the network to explore the features of *B. licheniformis* WX-02 and dissected the functional modules related to γ-PGA biosynthesis to provide insights into its regulatory mechanism. The predicted PPI network can be used as a valuable resource for studying the physiology and metabolisms of *B. licheniformis* WX-02.

## Results and Discussion

### Construction of the genome-scale PPI network

**Figure 1:**
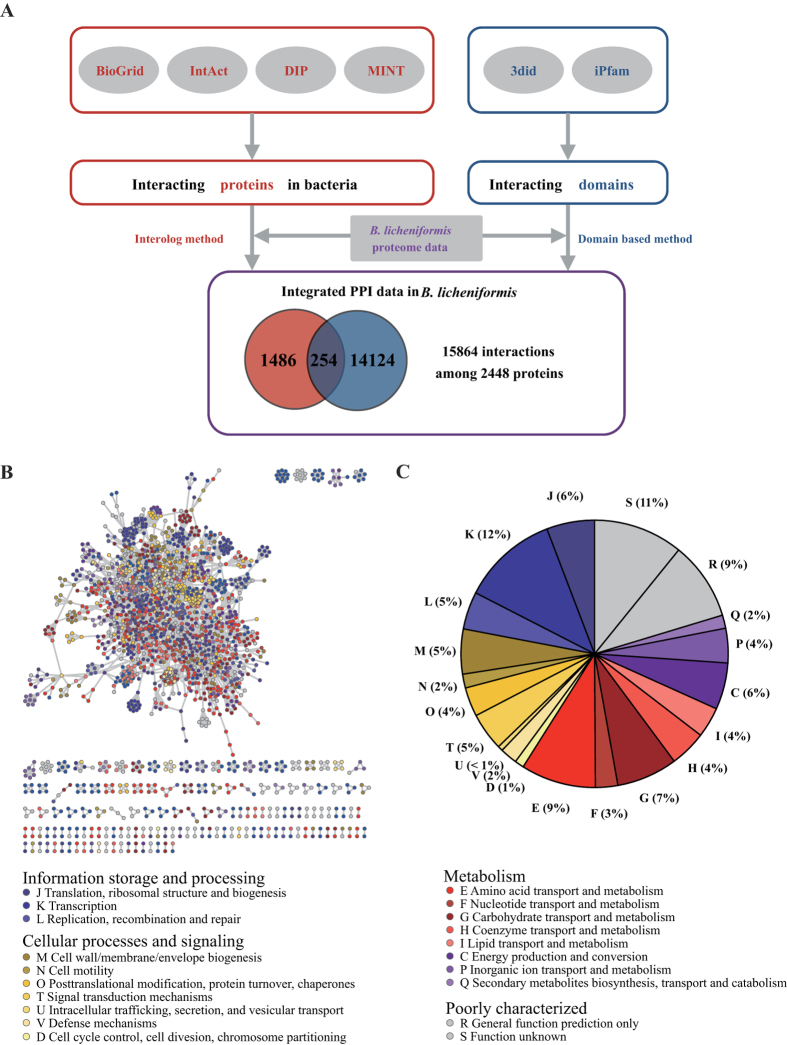
Flowchart for constructing PPI network in *B. licheniformis* WX-02 and overview of the network. (**A**) Flowchart for constructing PPI network. (**B**) Nodes of the network are colored according to their COG categories, and therefore nodes with the same color belong the same functional category. (**C**) The proportions of COG functional categories in the PPI network.

The PPI network was constructed by interolog method and domain-based method (Fig. 1A). These two methods predicted 1,740 and 14,378 PPIs respectively, and shared 254 PPIs. Finally, the merged non-redundant PPI network contains 15,864 edges and 2,448 nodes (see Supplementary Table S1 online). As homomeric interactions may cause bias in subsequent analysis, we excluded them from the network when investigating the relationships of interacting proteins^22^,^23^. As a result, the remained network comprised 13,664 interactions among 2,165 proteins.

The network was visualized by Cytoscape^24^ and nodes were colored according to their cluster of orthologous groups (COG) functional categories (Fig. 1B). The distribution of COG in PPI network is shown in Fig. 1C. Proteins involved in ‘transcription (K)’ accounted for the largest proportion (12%), which are highlighted in deep blue; while the proteins related to ‘intracellular trafficking, secretion, and vesicular transport (U)’ accounted for the smallest proportion (less than 1%), which are marked with light yellow. The above results suggest that many transcriptional regulation processes in *B. licheniformis* can be performed through the PPI network, which is similar to some cases reported in *Bacillus subtilis* (*B. subtilis*)^25^,^26^.

### Quality assessment of the PPI network

**Figure 2:**
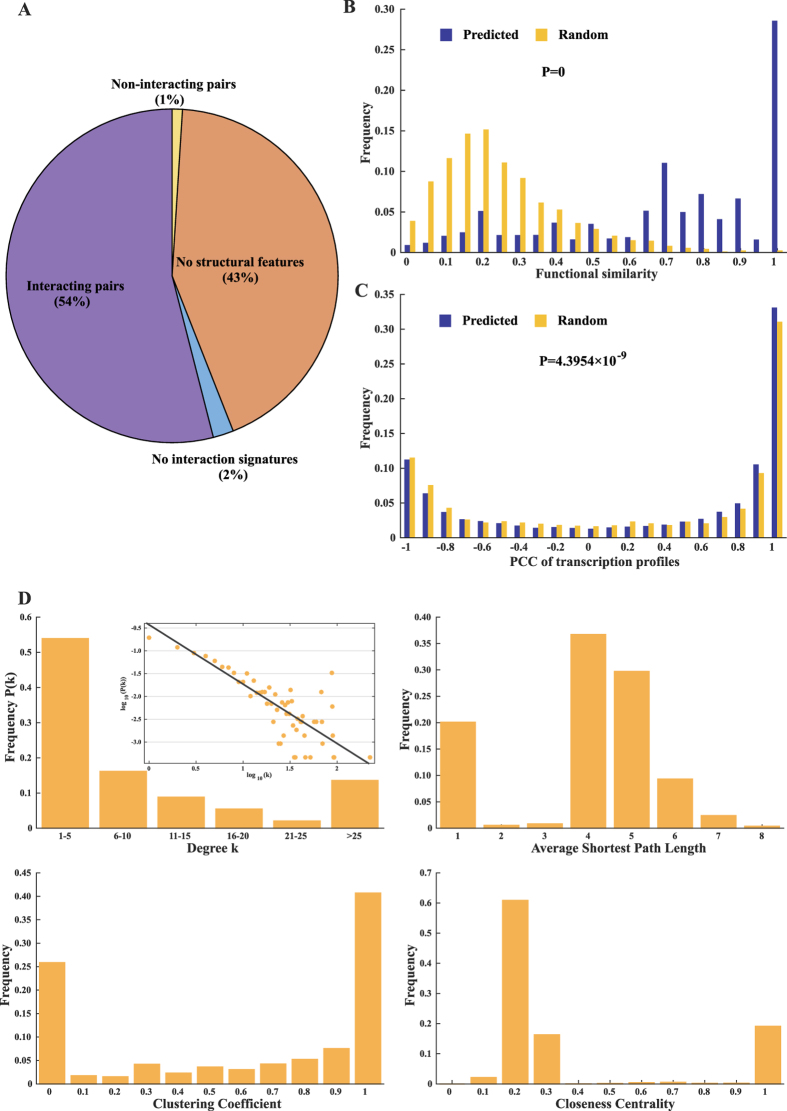
Validation and topological properties of the *B. licheniformis* WX-02 PPI network. (**A**) 1,000 randomly selected PPIs validated by iLoop web server. (**B**) Comparison of the GO similarity between the predicted PPI network and random networks with same topology. (**C**) Comparison of the PCC of gene transcription profiles between protein pairs derived from the PPI network and random networks with same topology. (**D**) Topological properties.

The accuracy of the predicted PPI network was evaluated from three perspectives: local structural features, functional similarities and gene transcription correlations. Firstly, we evaluated 1,000 randomly selected PPIs with a structural context method^27^,^28^. As well-characterized structural templates in available databases are limited, 43% of the selected PPIs contained at least one protein that had no structural features. Surprisingly, 54% of the PPIs could be confirmed and only 1% were classified as non-interacting pairs (Fig. 2A), indicating that more than half of our PPIs can be validated by local structural features and the PPI network is relatively reliable.

Functional similarities of interacting proteins can also be used to evaluate the quality of PPIs, since interacting proteins are prone to have similar functions^29^,^30^. We calculated the functional similarities of protein pairs in the PPI network and in random networks with the same topology according to their semantic similarities of gene ontology (GO) annotations based on reference^31^. Figure 2B shows that the functional similarities of protein pairs in the PPI network (mainly falling within 0.65 ~ 1) are significantly higher than those in random networks (most of which are less than 0.4).

In addition, we compared the Pearson correlation coefficient (PCC) of normalized transcription profiles between interacting and random protein pairs. Previous studies have demonstrated that interacting proteins tend to have similar transcription patterns^32^. Hence, an accurate PPI network should contain significantly more interacting protein pairs with similar transcription patterns than random networks. Based on gene transcription, we calculated the PCC between protein pairs in the PPI network and those in random networks with the same topology, respectively. Figure 2C demonstrates that the PCC value of transcription profiles of protein pairs in the PPI network is significantly higher than that in random networks.

Despite the fact that the resolution of theoretical methods is lower than that of some structural modeling methods^33^,^34^, and the PPIs detected in our study do not cover all the actually existing PPIs, the above results indicate a high accuracy of the predicted *B. licheniformis* PPI network.

### Properties of the PPI network

We calculated and analyzed the topological parameters of PPI network with Network Analysis plugins in Cytoscape^24^. As the case for many complex networks^35^, degree distribution of the PPI network in *B. licheniformis* WX-02 follows the power law, which characterizes the PPI network as a scale-free network (Fig. 2D). The average degree of this network is 12.6 and the degrees of 70% proteins are lower than 10. The average path length, cluster coefficient and the number of sub-networks are 4.7, 0.61 and 150, respectively. The largest sub-network contains 13,057 interactions and 1,718 proteins. Figure 2D shows that the distribution of average short path length, clustering coefficient and closeness centrality has two peaks, indicating the existence of many small sub-networks, whose topological parameters are quite different from those of the largest sub-network.

For the predicted PPI network, the degree exponent γ was calculated as 1.6 by the maximum likelihood estimate. It is well known that if the degree exponent is smaller than 2, relatively fewer nodes are needed to control the entire network^36^. These nodes were identified by minimum dominating set (MDS), since a previous study has reported that they play an important role in controlling the network^16^. In the present study, we determined a MDS in the *B. licheniformis* WX-02 PPI network by solving an integer-based linear programming problem. The resulting MDS contains 406 nodes, which account for less than 20% of the total nodes. To further analyze these important nodes, we performed COG enrichment analysis for them, finding that the proteins in MDS are significantly enriched in ‘carbohydrate transport and metabolism (G, fisher’s exact test, P < 0.05)’, ‘replication, recombination and repair (L, fisher’s exact test, P < 0.05)’ and ‘unknown function (S, fisher’s exact test, P < 0.01)’ (see Supplementary Table S2 online). Since the proteins in MDS are enriched in essential functional categories, such as cancer-related and virus-targeted genes in the PPI network of *Homo sapiens* and *Saccharomyces cerevisiae*^16^, the proteins with unknown function belonging to MDS in our PPI network might be involved in some important biological processes.

### Heat map of COG functions in the PPI network

**Figure 3:**
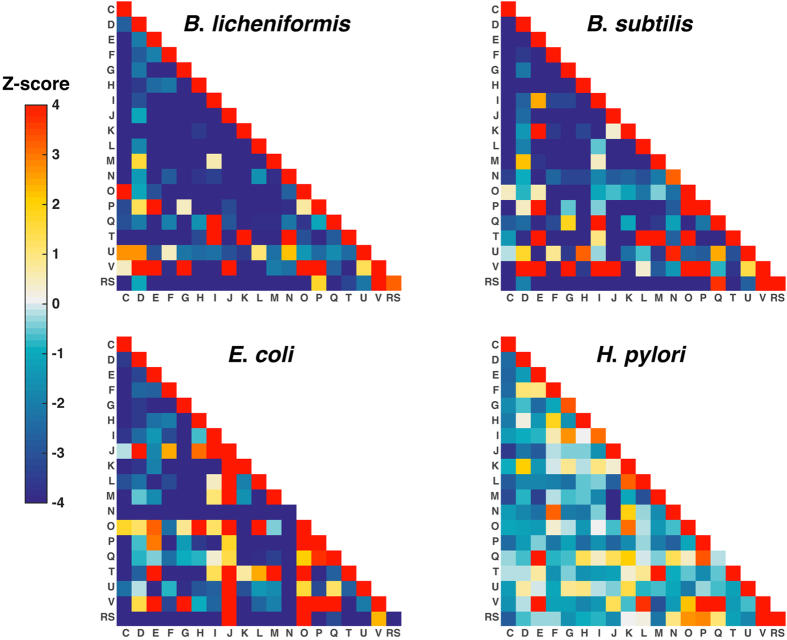
Heat map of COG functional categories of PPIs for four organisms (*B. licheniformis*, *B. subtilis*, *E. coli* and *H. pylori*). The numbers of PPIs among various COG functional categories were normalized by Z-score from a statistical model. The color indicates the enrichment degree of interactions between COG function categories.

In this study, we performed PPI enrichment analysis by presenting the PPI network as a heat map based on different COG categories (Fig. 3)^32^,^37^,^38^,^39^. The PPI networks of other three model species (*B. subtilis* 168, *E. coli* K12 and *H. pylori* 26695) and their corresponding heat maps were constructed for comparison. To ensure the reliability of the comparative results, we used the same computational methods and reference PPI data to establish their PPI networks as *B. licheniformis*. Finally, the networks of *B. subtilis*, *E. coli* and *H. pylori* include 15,862, 23,900 and 2,965 PPIs, among which 15,304, 22,945 and 2,287 have COG annotations respectively. From Fig. 3, it can be observed that the PPI data of these four strains are mainly enriched in diagonal regions, suggesting that most of the interactions occur within the same functional categories.

Nevertheless, the differences among the four heat maps are obvious, indicating the species-specific functional features of these bacterial strains. In *E. coli*, the majority of PPIs are related to ‘translation, ribosomal structure and biogenesis (J)’ or ‘posttranslational modification, protein turnover, chaperones (O)’, while in the other three strains, most PPIs are not dominated by one or two classes of proteins. In *Bacillus* species, the proteins related to ‘defense mechanisms (V)’ tend to interact with the proteins from ‘Intracellular trafficking, secretion, and vesicular transport (U)’, while this phenomenon was not observed in other two gram-negative bacteria. Therefore, it can be speculated that *Bacillus* species might have specific defense mechanism to protect themselves. Moreover, several specific functional features were discovered in *B. licheniformis* WX-02. For instance, we found that the proteins in ‘signal transduction mechanisms (T)’ category are highly connected with those in ‘transcription (K)’ category and ‘cell motility (N)’ category. On the other hand, it is interesting that the interactions between ‘Cell wall/membrane/envelope biogenesis (M)’ and ‘Signal transduction mechanisms (T)’ proteins are all enriched in the networks of *B. subtilis*, *E. coli* and *H. pylori*, except for in that of *B. licheniformis*. These different features suggest that there might be unique complex regulatory mechanisms in *B. licheniformis* WX-02, which provide an effective way to explain its physiological characteristics and complex cellular behaviors.

### Analysis and comparison of the PPI networks under normal and high salt conditions

**Figure 4:**
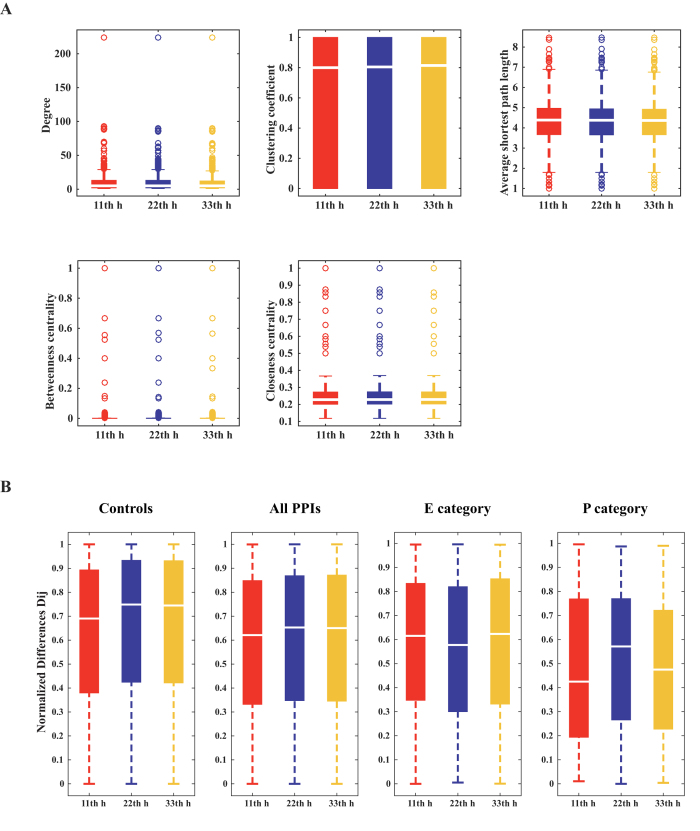
Comparison of local topological properties and normalized difference under normal and high salt conditions. (**A**) Comparison of degree, clustering coefficient, average shortest path length, betweenness centrality and closeness centrality by Wilcoxon rank sum test. (**B**) Comparison of normalized differences for three time points and four groups of protein pairs. ‘Control’ represents all the pairwise proteins in the PPI network; ‘All PPI’ represents all the interacting protein pairs in the PPI network. ‘E category’ represents the interacting protein pairs belonging to ‘amino acid transport and metabolism (E)’; ‘P category’ represents the interacting protein pairs belonging to ‘inorganic ion transport and metabolism (P)’.

To investigate the dynamics of the PPI networks under normal and high salt conditions, we incorporated the strand-specific RNA-seq (ssRNA-seq) data into the PPI network and obtained three sub-networks with expressed genes at different time points (network1 for normal condition at 11th h, network2 for early long-term salt adaption at 22th h and network3 for late long-term salt adaption at 33th h)^14^. In order to explore the differences and similarities of these three networks, we performed analysis from two perspectives: topology and transcription differences between the interacting proteins. Firstly, we analyzed their topological properties by calculating 5 local topology metrics for each node in the corresponding networks, including degree, clustering coefficient, average shortest path length, betweenness centrality and closeness centrality. Interestingly, no significant differences were detected in the distributions of these local topology metrics for the three networks (Fig. 4A). These comparative results suggest that though the transcription levels and phenotypes are significantly different under normal and high salt conditions^14^, the topological properties of the PPI network are robust.

On the other hand, we investigated the absolute transcription levels between the interacting proteins, because their relative stoichiometrical amounts can affect productivity and efficiency. To this end, we defined the normalized transcription difference as the proportion of the difference value between the reads per kilobase of ORF per million mapped reads (RPKM) of two interacting proteins to the sum of their RPKM values^40^. Figure 4B shows the normalized difference distribution of the protein pairs at three time points for four groups *i.e*., ‘control’ group (all possible protein pairs in the network), ‘all PPI’ group (all interacting protein pairs in the network), sub-networks related to ‘amino acid transport and metabolism (E category)’ and ‘inorganic ion transport and metabolism (P category)’. From Fig. 4B, it is observed that the normalized difference distribution of ‘control’ group (all possible protein pairs in the network) is higher than that of ‘all PPI’ group (all interacting protein pairs in the network), revealing that the transcription levels of the interacting proteins are more approximate. By comparing the normalized difference distribution of PPI networks for three time points, we found that the median of the normalized difference distribution of network1 was smaller than that of network2 and network3 (Fig. 4B), demonstrating that the normalized difference distribution of PPIs is affected under high salt condition, which is consistent with the analysis of transcription profiles. Interestingly, sub-networks of ‘E category’ and ‘P category’ exhibit opposite trends. The normalized difference distribution of interactions between the proteins related to ‘E category’ is decreased at 22th h and then is restored to the normal level at 33th h. These changes might result in a more rational ratio of interacting proteins that are responsible for amino acid metabolism and acceleration of amino acid synthesis. However, the normalized difference distribution of interactions between the proteins related to ‘P category’ proteins is increased at 22th h relative to the normal condition. This change might contribute to the weakening of ion transport processes, the diminishing of ion-exchange amount and the maintaining of a stable osmotic pressure under long-term salt adaption. At 33th h, the transcription levels of many ‘P category’ proteins decrease to the levels under normal condition. The above results might explain the change of colony forming units (CFU), as the CFU decreased rapidly after the addition of 6% NaCl solution to the medium at 11th h, then the strain slowly resumed growth at about 22th h and the biomass reached almost the same level as in 11th h at 33th h^14^.

### Identification of the protein complexes and prediction of the functions for uncharacterized proteins

**Figure 5:**
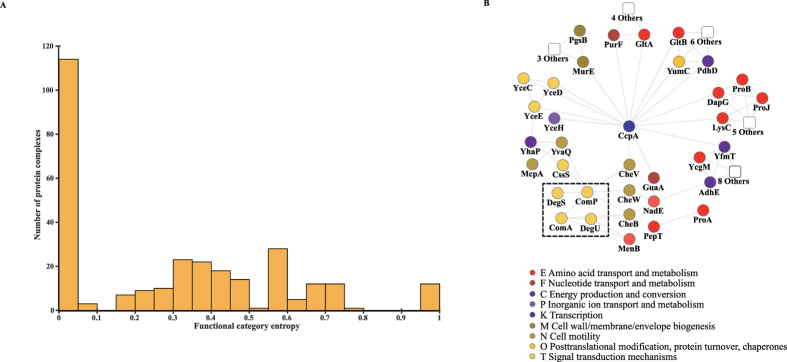
Functional category entropy distribution of protein complexes and sub-network related to γ-PGA synthesis and regulation. (**A**) Functional category entropy distribution of protein complexes. (**B**) The proteins are colored based on their COG functional categories.

PPI network is a powerful tool to predict the functions of poorly characterized proteins. In this study, we proposed a two-step approach to determine the protein functions: identifying the protein complexes in the PPI network, and then predicting the protein functions based on these protein complexes. By using a clustering algorithm TSN-PCD^41^, we finally obtained 267 different protein complexes (see Supplementary Table S3 online). After obtaining the protein complexes, the functional category entropy for each protein complex was calculated according to COG functional categories. As expected, the functions of proteins belonging to the same protein complex are prone to be consistent (Fig. 5A), indicating that the protein function within a certain complex can be predicted through the enriched COG functional categories. With this module-assisted method^42^, we finally annotated 117 proteins with unknown functions (see Supplementary Table S4 online).

### Analysis of the sub-network related to γ-PGA synthesis and regulation

Some studies have reported that *B. licheniformis* WX-02 can produce γ-PGA under normal condition and has a much higher yield under high salt environment^14^,^43^. Up to now, genes (*pgsB*, *pgsC*, *pgsA* and *pgsE*) related to γ-PGA synthesis have been reported in *B. subtilis* and *B. licheniformis*. Although a series of molecular and cellular studies have been performed on *B. licheniformis*, the regulation mechanism of γ-PGA is still not clear. Here, we analyzed the sub-network related to γ-PGA synthesis and regulation. Figure 5B shows that a hub protein CcpA directly or indirectly joints many proteins related to γ-PGA synthesis (PgsB) and regulation, such as GltA and GltB (which together encode glutamate-oxoglutarate amidotransferase), proteins related to proline metabolism (ProB, ProJ, YcgM) and two signal transduction systems ComP-ComA and DegS-DegU (Fig. 5B).

The CcpA transcriptional regulator is a central regulatory factor in the intersection between carbon and nitrogen metabolism^44^, and can regulate the metabolisms by interacting with other proteins^45^. According to Fig. 5B, it can be inferred that CcpA might also be related to γ-PGA synthesis through the PPI network. To illustrate this point, we further analyzed the sub-network. First of all, the γ-PGA synthesis protein PgsB can interact indirectly with CcpA through UDP-N-acetylmuramoyl-L-alanyl-D-glutamate-2,6-diaminopimelate ligase (murE). Also, CcpA can interact directly with proteins GltA and GltB encoding glutamate-oxoglutarate amidotransferase (GOGAT), which play an important role in the upstream pathway of γ-PGA synthesis^46^. Thus, it can be speculated that CcpA might affect the γ-PGA synthesis by regulating the GOGAT through protein interactions. In addition, CcpA is connected with several chemotaxis proteins, and further interacts with two signal transduction systems ComP-ComA and DegS-DegU. It is well known that the synthesis of γ-PGA is under the control of these two signal transduction systems^47^,^48^. These results suggest that CcpA might first interact with chemotaxis proteins and regulate their expression, and then these chemotaxis proteins affect the regulation of ComP-ComA and DegS-DegU to regulate the γ-PGA synthesis of *B. licheniformis* WX-02. Based on the above analyses, CcpA can play an important central role in the regulation of γ-PGA synthesis through interacting with some related proteins.

## Conclusions

In this work, we presented a genome-wide PPI network with 15,864 edges and 2,448 nodes of *B. licheniformis* WX-02 by combining interolog method and domain based method. The PPI network was subsequently verified from three perspectives: local structural features, functional similarities and transcription correlations. Although the predicted PPI network is far from perfect, it can provide new insights into the research of *B. licheniformis* WX-02. By analyzing and comparing the networks under normal and high salt conditions based on transcriptome data, we found that the topological properties of the PPI network are robust to tolerate fluctuations in transcription levels as well as changes in environmental conditions. In addition, we predicted 267 different protein complexes and annotated 117 poorly uncharacterized proteins based on the network. Further analyses of the sub-network show that the hub protein CcpA interacts directly or indirectly with many proteins involved in γ-PGA synthesis and regulation, indicating that CcpA might play an important role in regulating γ-PGA synthesis through the PPI network. The predicted PPI network will provide a significant foundation for exploring the molecular mechanisms of *B. licheniformis* WX-02 and developing optimized industry strains for producing chemicals.

## Material and Methods

### Data source

To construct the PPI network of *B. licheniformis*, we collected both the experimental interacting protein pairs and domain pairs from the databases. Totally, 44,648 experimental PPIs among 11,196 proteins for bacteria were downloaded from BioGRID^49^, IntAct^50^, DIP^51^ and MINT^52^ databases (Table 1). 9,590 domain-domain interactions (DDIs) among 5,619 domains were collected from iPfam^53^ and 3did^54^ databases. All the protein sequences were retrieved from NCBI RefSeq and UniProt. Domain alignment profiles were obtained from Pfam database^55^.

### Interolog method

This prediction method is based on the conserved proteins in different species^56^. We detected the potential orthologs between *B. licheniformis* and reference organisms using BLASTP (*E*-value ≤ 10^−5^, sequence identity ≥30%, and alignment coverage ≥60%). To ensure the accuracy of the predicted results, protein pairs with the highest alignment score were kept if a protein corresponded to multiple homologs in one organism. This process might reduce the number of predicted interactions, but it could minimize the false positive rate. For any two proteins in *B. licheniformis*, if their orthologs in the reference genomes had at least one experimentally determined interaction, the two proteins were considered to have interaction.

### Domain-domain interaction based method

The method attempts to predict protein interactions based on the experimentally and structurally determined DDIs. For a protein pair (*X* and *Y*) in *B. licheniformis*, we assumed that *m* and *n* were one domain in protein *X* and *Y* respectively. If *m* and *n* were proved to be an experimental interacting domain pair, *X* and *Y* were considered to have interaction. Domains of proteins in *B. licheniformis* were predicted based on the Pfam domain database and HMMER program (*E*-value ≤ 10^−5^, bias ≤ 1)^57^. The interacting domain pairs were checked based on the data from iPfam and 3did databases.

### Network validation

To confirm the predicted PPIs, we randomly selected 1,000 PPIs and submitted them to the PPI prediction web server (http://sbi.imim.es/iLoops.php)^28^. This web server, which defines protein structural features based on the loops from ArchDB^58^ and domains from SCOP^59^, was used to validate the PPIs by evaluating whether loop or domain patterns from two input proteins had interaction signatures with random forest classifier.

In addition, we used a method based on GO functional similarities to confirm the PPIs. It is well known that two interacting proteins tend to have similar or related functions. Based on this assumption, we compared the GO functional similarities between the predicted PPI network and 100 random networks (with the same topology as the PPI network). The GO annotations of *B. licheniformis* genome were downloaded from GO database^60^. Totally, 1,682 of 2,165 proteins in the predicted PPI network had GO annotation. Then, the semantic similarities of GO terms and functional similarities of proteins in the PPI and random networks were calculated with the algorithms proposed by reference^31^. The comparison of functional similarity distributions between PPI network and random networks was performed with Wilcoxon rank-sum test.

Moreover, gene transcription correlations of interacting proteins were also used to access the reliability of the PPI network. The ssRNA-seq data of *B. licheniformis* WX-02 for three time points (11th h, 22th h and 33th h) were obtained from the previous study^14^. The PCC of gene transcription profiles of the protein pairs in the PPI and 100 random networks was compared. The statistical difference between the predicted PPI network and random networks was also measured by *P*-value from Wilcoxon rank-sum test.

### Analysis of COG functional heat map

Based on COG functional categories, the PPI data were presented as heat map. Colors in the heat map indicate *Z*-scores calculated by a statistical model. Considering that a randomized network contained same nodes as the predicted PPI network, the probability for a protein in functional class *i* to interact with a protein in functional class *j* in the randomized network was calculated as:


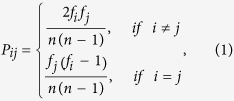


where *n* is the total number of proteins in the predicted PPI network and *f*_*i*_ is the number of proteins belonging to functional class *i*. In the randomized network, the number of interactions between proteins from functional class *i* and *j* was assumed to follow a binomial distribution. Finally, the *Z*-scores were calculated as:


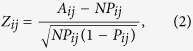


where *A*_*ij*_, *NP*_*ij*_ and *NP*_*ij*_(1 − *P*_*ij*_) represent the actual value, expected value and variance of the number of interactions between proteins from functional class *i* and *j*, respectively.

### Dynamic changes of the PPI network under normal and high salt conditions

The transcription profiles were obtained from three sample points: 11th h (0 h after the onset of 6% NaCl), 22th h (11 h after the onset of exposure to 6% NaCl) and 33th h (22 h after the onset of exposure to 6% NaCl), which have been reported in the previous study^14^. Firstly, we used RPKM to represent the normalized transcription levels of genes. Then, we assigned the RPKM values of each time point to the corresponding nodes in PPI network to obtain three networks. Here we defined a rule: if the RPKM value of a gene was lower than 1, this gene was considered to have no effect on the PPI network and would be removed from the network. Based on this process, we obtained three new PPI networks (network1 for 11th h, network2 for 22th h and network3 for 33th h) for different experimental conditions.

We calculated the normalized transcription difference *D*_*ij*_ between a pair of proteins *i* and *j* as defined in the previous study^40^:


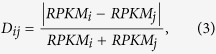


where *RPKM*_*i*_ represents the RPKM value of gene *i*, and this value ranges from 0 to 1.

### Prediction of the protein complexes and annotation of the protein functions

Protein complexes in the PPI network were identified using a clustering algorithm named TSN-PCD^41^. The inputs of TSN-PCD were PPIs and gene transcription data, which were used to generate time-series sub-networks. Then the clustering was performed based on these subnetworks. In this algorithm, the threshold of gene transcription level (RPKM value), λ (a parameter affecting the clustering results) and size value were set as 1, 1 and 3, respectively. In information theory, entropy is used to measure uncertainty or variability of complex systems. In this study, we defined the functional category entropy of a protein complex to indicate the function homogeneity of the protein complex. The functional category entropy was calculated as follows:


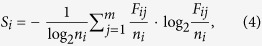


where *n*_*i*_ is the number of proteins in the complex *i* and *F*_*ij*_ is the number of proteins annotated with the function *j* in the complex *i*. The lower entropy means greater homogeneity. The homogeneity is ascribed to a specific function enriched in a protein complex. Therefore, we could assign functions to the uncharacterized proteins with the functions enriched in a protein complex. The function enrichment analysis was performed based on fisher’s exact test.

## Additional Information

**How to cite this article**: Han, Y.-C. *et al.* Prediction and characterization of protein-protein interaction network in *Bacillus licheniformis* WX-02. *Sci. Rep.* **6**, 19486; doi: 10.1038/srep19486 (2016).

## Supplementary Material

Supplementary Information

## Figures and Tables

**Table 1:** High-quality PPIs and DDIs obtained from different public databases.

Database	No. Proteins/Domains	No. PPIs/DDIs
BioGRID	70	66
IntAct	10,232	38,758
DIP	4,259	14,595
MINT	1,384	3,782
3did	5,466	8,651
iPfam	4,775	9,516
