# Supplementary material for: Prediction and characterization of protein-protein interaction network in Bacillus licheniformis WX-02
Source: Sci Rep. 2016 Jan 19;6:19486. doi: 10.1038/srep19486 (PMC4726086; doi:10.1038/srep19486)
Supplement: Supplementary Information [file srep19486-s1.pdf]

Supplementary information for

Prediction and characterization of protein-protein interaction network in *Bacillus licheniformis* WX-02

Yi-Chao Han[1, *], Jia-Ming Song[1, *], Long Wang[1], Cheng-Cheng Shu[1], Jing Guo[1, #] and Ling-Ling Chen[1, #]

[1] College of Informatics, Agricultural Bioinformatics Key Laboratory of Hubei Province, Huazhong Agricultural University, Wuhan 430070, P. R. China

[*]These authors contributed equally to this work.

[#]Correspondence and requests for materials should be addressed to: L.-L. C. (email: llchen@mail.hzau.edu.cn) or J. G. (gj30501@163.com)

Supplementary Table S1. The predicted protein-protein interactions in *B. licheniformis* WX-02.

Supplementary Table S2. The functional categories of proteins in MDS.

Supplementary Table S3. The predicted protein complexes.

Supplementary Table S4. Functional annotation for poorly uncharacterized proteins. *P* -values were calculated for each function (*P<0.05, **P<0.01, ***P<0.001).

Supplementary Table S1. The predicted protein-protein interactions in B. licheniformis WX-02.

Interaction partner protein A	Interaction partner protein B
MUY_01001	MUY_01000
MUY_01002	MUY_01002
MUY_01002	MUY_02132
MUY_01002	MUY_01000
MUY_01003	MUY_01003
MUY_01010	MUY_01010
MUY_01010	MUY_02110
MUY_01011	MUY_01011
MUY_01011	MUY_01832
MUY_01011	MUY_03069
MUY_01011	MUY_00037
MUY_01012	MUY_01012
MUY_01012	MUY_03388
MUY_01012	MUY_00578
MUY_01015	MUY_01015
MUY_01015	MUY_01060
MUY_01015	MUY_01062
MUY_01015	MUY_01492
MUY_01015	MUY_00010
MUY_01015	MUY_01719
MUY_01015	MUY_01857
MUY_01015	MUY_02376
MUY_01015	MUY_02991
MUY_01015	MUY_03222
MUY_01015	MUY_03263
MUY_01015	MUY_03292
MUY_01015	MUY_03434
MUY_01015	MUY_04020
MUY_01015	MUY_04022
MUY_01016	MUY_01016
MUY_01016	MUY_00200
MUY_01016	MUY_02834
MUY_01016	MUY_03186
MUY_01016	MUY_03876
MUY_01016	MUY_04337
MUY_01016	MUY_00708
MUY_01017	MUY_01017
MUY_01017	MUY_01049
MUY_01017	MUY_01075
MUY_01017	MUY_01244
MUY_01017	MUY_01609
MUY_01017	MUY_01693
MUY_01017	MUY_01735
MUY_01017	MUY_02030

MUY_01017	MUY_02361
MUY_01017	MUY_02588
MUY_01017	MUY_00297
MUY_01017	MUY_03518
MUY_01017	MUY_03667
MUY_01017	MUY_04463
MUY_01017	MUY_00557
MUY_01017	MUY_00583
MUY_01017	MUY_00674
MUY_01019	MUY_01019
MUY_01019	MUY_01088
MUY_01019	MUY_01089
MUY_01019	MUY_01095
MUY_01019	MUY_01113
MUY_01019	MUY_01130
MUY_01019	MUY_00110
MUY_01019	MUY_01283
MUY_01019	MUY_01284
MUY_01019	MUY_01297
MUY_01019	MUY_01298
MUY_01019	MUY_01461
MUY_01019	MUY_01466
MUY_01019	MUY_00134
MUY_01019	MUY_00152
MUY_01019	MUY_00153
MUY_01019	MUY_01652
MUY_01019	MUY_01653
MUY_01019	MUY_01656
MUY_01019	MUY_01664
MUY_01019	MUY_01762
MUY_01019	MUY_00189
MUY_01019	MUY_00194
MUY_01019	MUY_02151
MUY_01019	MUY_02208
MUY_01019	MUY_02209
MUY_01019	MUY_02225
MUY_01019	MUY_02597
MUY_01019	MUY_02598
MUY_01019	MUY_02609
MUY_01019	MUY_02643
MUY_01019	MUY_02700
MUY_01019	MUY_02744
MUY_01019	MUY_02745
MUY_01019	MUY_02760
MUY_01019	MUY_00265
MUY_01019	MUY_02894
MUY_01019	MUY_02929

MUY_01019	MUY_02945
MUY_01019	MUY_02946
MUY_01019	MUY_00285
MUY_01019	MUY_03154
MUY_01019	MUY_03330
MUY_01019	MUY_03332
MUY_01019	MUY_03354
MUY_01019	MUY_00322
MUY_01019	MUY_03575
MUY_01019	MUY_03576
MUY_01019	MUY_03582
MUY_01019	MUY_03598
MUY_01019	MUY_03634
MUY_01019	MUY_03649
MUY_01019	MUY_03676
MUY_01019	MUY_03785
MUY_01019	MUY_03805
MUY_01019	MUY_03900
MUY_01019	MUY_03946
MUY_01019	MUY_00372
MUY_01019	MUY_03978
MUY_01019	MUY_04144
MUY_01019	MUY_04172
MUY_01019	MUY_04248
MUY_01019	MUY_04254
MUY_01019	MUY_04266
MUY_01019	MUY_04269
MUY_01019	MUY_04282
MUY_01019	MUY_04296
MUY_01019	MUY_00388
MUY_01019	MUY_04348
MUY_01019	MUY_04357
MUY_01019	MUY_00394
MUY_01019	MUY_04403
MUY_01019	MUY_04416
MUY_01019	MUY_04445
MUY_01019	MUY_04453
MUY_01019	MUY_00420
MUY_01019	MUY_00434
MUY_01019	MUY_00442
MUY_01019	MUY_00516
MUY_01019	MUY_00585
MUY_01019	MUY_00800
MUY_01019	MUY_00815
MUY_01019	MUY_00937
MUY_01019	MUY_00938
MUY_01019	MUY_00939

MUY_01019	MUY_00968
MUY_01019	MUY_00975
MUY_01019	MUY_00996
MUY_01019	MUY_01000
MUY_01021	MUY_01021
MUY_01023	MUY_01023
MUY_01023	MUY_01419
MUY_01023	MUY_01422
MUY_01023	MUY_01936
MUY_01023	MUY_02132
MUY_01023	MUY_03965
MUY_01023	MUY_00554
MUY_01024	MUY_01024
MUY_01026	MUY_01026
MUY_01026	MUY_01767
MUY_01026	MUY_02095
MUY_01026	MUY_03170
MUY_01026	MUY_03691
MUY_01026	MUY_04192
MUY_01026	MUY_04434
MUY_01026	MUY_04482
MUY_01026	MUY_00583
MUY_01026	MUY_00998
MUY_01027	MUY_01027
MUY_01027	MUY_01163
MUY_01027	MUY_01683
MUY_01027	MUY_02127
MUY_01027	MUY_02171
MUY_01027	MUY_02269
MUY_01027	MUY_02703
MUY_01027	MUY_02841
MUY_01027	MUY_02941
MUY_01027	MUY_03513
MUY_01027	MUY_03515
MUY_01027	MUY_03524
MUY_01027	MUY_00461
MUY_01028	MUY_01028
MUY_01028	MUY_00185
MUY_01028	MUY_03267
MUY_01030	MUY_01030
MUY_01030	MUY_01152
MUY_01030	MUY_01382
MUY_01030	MUY_02707
MUY_01030	MUY_02708
MUY_01030	MUY_02882
MUY_01030	MUY_02883
MUY_01030	MUY_02889

MUY_01030	MUY_02892
MUY_01030	MUY_02900
MUY_01030	MUY_03708
MUY_01030	MUY_03769
MUY_01030	MUY_03778
MUY_01030	MUY_03830
MUY_01030	MUY_04185
MUY_01030	MUY_04271
MUY_01030	MUY_04291
MUY_01030	MUY_04501
MUY_01030	MUY_04505
MUY_01030	MUY_00551
MUY_01030	MUY_00599
MUY_01030	MUY_00602
MUY_01030	MUY_00628
MUY_01030	MUY_00691
MUY_01030	MUY_00795
MUY_01030	MUY_00086
MUY_01030	MUY_00973
MUY_01031	MUY_01031
MUY_01031	MUY_02534
MUY_01032	MUY_01032
MUY_01032	MUY_01108
MUY_01032	MUY_01109
MUY_01032	MUY_01203
MUY_01032	MUY_01308
MUY_01032	MUY_01432
MUY_01032	MUY_01527
MUY_01032	MUY_01582
MUY_01032	MUY_01626
MUY_01032	MUY_01670
MUY_01032	MUY_01874
MUY_01032	MUY_01875
MUY_01032	MUY_00205
MUY_01032	MUY_02178
MUY_01032	MUY_02366
MUY_01032	MUY_00229
MUY_01032	MUY_02521
MUY_01032	MUY_02523
MUY_01032	MUY_00249
MUY_01032	MUY_02655
MUY_01032	MUY_02663
MUY_01032	MUY_00263
MUY_01032	MUY_00264
MUY_01032	MUY_02912
MUY_01032	MUY_02939
MUY_01032	MUY_02940

MUY_01032	MUY_02964
MUY_01032	MUY_00288
MUY_01032	MUY_00289
MUY_01032	MUY_03059
MUY_01032	MUY_03117
MUY_01032	MUY_03127
MUY_01032	MUY_03139
MUY_01032	MUY_03183
MUY_01032	MUY_03201
MUY_01032	MUY_03202
MUY_01032	MUY_03252
MUY_01032	MUY_03306
MUY_01032	MUY_03374
MUY_01032	MUY_03463
MUY_01032	MUY_03477
MUY_01032	MUY_03478
MUY_01032	MUY_03483
MUY_01032	MUY_03609
MUY_01032	MUY_03619
MUY_01032	MUY_03647
MUY_01032	MUY_03648
MUY_01032	MUY_03924
MUY_01032	MUY_04096
MUY_01032	MUY_04146
MUY_01032	MUY_04177
MUY_01032	MUY_04211
MUY_01032	MUY_04212
MUY_01032	MUY_04293
MUY_01032	MUY_04294
MUY_01032	MUY_04358
MUY_01032	MUY_04359
MUY_01032	MUY_04379
MUY_01032	MUY_04417
MUY_01032	MUY_04418
MUY_01032	MUY_04447
MUY_01032	MUY_04448
MUY_01032	MUY_04471
MUY_01032	MUY_00418
MUY_01032	MUY_00436
MUY_01032	MUY_00575
MUY_01032	MUY_00576
MUY_01032	MUY_00790
MUY_01032	MUY_00961
MUY_01033	MUY_01033
MUY_01033	MUY_02054
MUY_01033	MUY_04188
MUY_01033	MUY_00993

MUY_01036	MUY_01036
MUY_01036	MUY_02129
MUY_01036	MUY_02320
MUY_01036	MUY_03015
MUY_01036	MUY_03490
MUY_01036	MUY_04046
MUY_01037	MUY_01037
MUY_01037	MUY_01783
MUY_01037	MUY_03608
MUY_01037	MUY_00344
MUY_01038	MUY_00935
MUY_01040	MUY_01040
MUY_01040	MUY_01372
MUY_01040	MUY_01406
MUY_01040	MUY_01623
MUY_01040	MUY_01969
MUY_01040	MUY_02062
MUY_01040	MUY_02258
MUY_01040	MUY_02321
MUY_01040	MUY_02508
MUY_01040	MUY_02515
MUY_01040	MUY_00021
MUY_01040	MUY_03050
MUY_01041	MUY_01041
MUY_01041	MUY_01365
MUY_01041	MUY_01368
MUY_01041	MUY_01634
MUY_01041	MUY_00207
MUY_01041	MUY_02203
MUY_01041	MUY_02978
MUY_01041	MUY_00290
MUY_01041	MUY_03320
MUY_01041	MUY_03640
MUY_01041	MUY_03984
MUY_01041	MUY_04198
MUY_01041	MUY_00401
MUY_01041	MUY_00563
MUY_01042	MUY_01042
MUY_01042	MUY_03206
MUY_01042	MUY_04243
MUY_01043	MUY_01043
MUY_01044	MUY_01044
MUY_01044	MUY_01164
MUY_01044	MUY_01245
MUY_01044	MUY_01280
MUY_01044	MUY_01329
MUY_01044	MUY_01421

MUY_01044	MUY_01611
MUY_01044	MUY_01625
MUY_01044	MUY_01923
MUY_01044	MUY_02100
MUY_01044	MUY_02153
MUY_01044	MUY_02211
MUY_01044	MUY_02331
MUY_01044	MUY_02615
MUY_01044	MUY_02633
MUY_01044	MUY_02950
MUY_01044	MUY_02958
MUY_01044	MUY_00292
MUY_01044	MUY_03128
MUY_01044	MUY_03225
MUY_01044	MUY_00313
MUY_01044	MUY_03494
MUY_01044	MUY_03606
MUY_01044	MUY_03627
MUY_01044	MUY_03638
MUY_01044	MUY_03964
MUY_01044	MUY_03998
MUY_01044	MUY_04038
MUY_01044	MUY_00486
MUY_01044	MUY_00681
MUY_01044	MUY_00933
MUY_01046	MUY_01046
MUY_01046	MUY_01239
MUY_01046	MUY_00392
MUY_01047	MUY_01047
MUY_01047	MUY_01437
MUY_01047	MUY_02240
MUY_01047	MUY_03314
MUY_01047	MUY_03980
MUY_01047	MUY_04043
MUY_01047	MUY_00385
MUY_01047	MUY_04343
MUY_01047	MUY_00722
MUY_01048	MUY_01048
MUY_01048	MUY_01114
MUY_01048	MUY_01141
MUY_01048	MUY_01237
MUY_01048	MUY_01418
MUY_01048	MUY_01520
MUY_01048	MUY_01616
MUY_01048	MUY_01919
MUY_01048	MUY_01937
MUY_01048	MUY_01989

MUY_01048	MUY_00190
MUY_01048	MUY_02094
MUY_01048	MUY_02152
MUY_01048	MUY_00206
MUY_01048	MUY_02439
MUY_01048	MUY_02467
MUY_01048	MUY_02534
MUY_01048	MUY_02627
MUY_01048	MUY_02780
MUY_01048	MUY_02797
MUY_01048	MUY_00261
MUY_01048	MUY_03146
MUY_01048	MUY_03191
MUY_01048	MUY_00308
MUY_01048	MUY_03333
MUY_01048	MUY_03386
MUY_01048	MUY_03449
MUY_01048	MUY_03454
MUY_01048	MUY_03496
MUY_01048	MUY_03637
MUY_01048	MUY_03810
MUY_01048	MUY_04070
MUY_01048	MUY_04086
MUY_01048	MUY_04242
MUY_01048	MUY_00382
MUY_01048	MUY_04302
MUY_01048	MUY_04334
MUY_01048	MUY_04383
MUY_01048	MUY_04433
MUY_01048	MUY_04483
MUY_01048	MUY_00435
MUY_01048	MUY_00038
MUY_01048	MUY_00450
MUY_01048	MUY_00464
MUY_01048	MUY_00780
MUY_01048	MUY_00808
MUY_01048	MUY_00840
MUY_01049	MUY_01049
MUY_01049	MUY_01075
MUY_01049	MUY_01244
MUY_01049	MUY_01609
MUY_01049	MUY_01693
MUY_01049	MUY_01735
MUY_01049	MUY_02030
MUY_01049	MUY_02361
MUY_01049	MUY_02588
MUY_01049	MUY_00297

MUY_01049	MUY_03518
MUY_01049	MUY_03667
MUY_01049	MUY_04463
MUY_01049	MUY_00557
MUY_01049	MUY_00583
MUY_01049	MUY_00674
MUY_01050	MUY_01051
MUY_01051	MUY_01052
MUY_01052	MUY_01052
MUY_01052	MUY_00105
MUY_01052	MUY_00114
MUY_01052	MUY_00115
MUY_01052	MUY_01390
MUY_01052	MUY_01504
MUY_01052	MUY_01696
MUY_01052	MUY_01760
MUY_01052	MUY_01761
MUY_01052	MUY_01879
MUY_01052	MUY_00181
MUY_01052	MUY_00182
MUY_01052	MUY_02516
MUY_01052	MUY_02560
MUY_01052	MUY_02784
MUY_01052	MUY_02845
MUY_01052	MUY_02852
MUY_01052	MUY_02889
MUY_01052	MUY_02892
MUY_01052	MUY_02900
MUY_01052	MUY_03708
MUY_01052	MUY_03918
MUY_01052	MUY_04026
MUY_01052	MUY_04258
MUY_01052	MUY_04322
MUY_01052	MUY_04504
MUY_01052	MUY_00541
MUY_01052	MUY_00571
MUY_01052	MUY_00628
MUY_01053	MUY_01053
MUY_01053	MUY_01176
MUY_01053	MUY_01230
MUY_01053	MUY_01523
MUY_01053	MUY_03721
MUY_01053	MUY_00512
MUY_01057	MUY_01057
MUY_01057	MUY_01058
MUY_01058	MUY_01058
MUY_01059	MUY_01059

MUY_01059	MUY_01140
MUY_01059	MUY_01374
MUY_01059	MUY_01970
MUY_01059	MUY_01999
MUY_01059	MUY_02036
MUY_01059	MUY_02049
MUY_01059	MUY_02231
MUY_01059	MUY_02238
MUY_01059	MUY_02297
MUY_01059	MUY_02580
MUY_01059	MUY_02982
MUY_01059	MUY_03140
MUY_01059	MUY_03250
MUY_01059	MUY_03431
MUY_01059	MUY_00323
MUY_01059	MUY_03553
MUY_01059	MUY_03612
MUY_01059	MUY_00445
MUY_01059	MUY_00677
MUY_01059	MUY_00749
MUY_01059	MUY_00779
MUY_01059	MUY_00882
MUY_01060	MUY_01060
MUY_01060	MUY_01062
MUY_01060	MUY_01114
MUY_01060	MUY_01492
MUY_01060	MUY_00010
MUY_01060	MUY_01719
MUY_01060	MUY_01857
MUY_01060	MUY_02198
MUY_01060	MUY_02376
MUY_01060	MUY_02991
MUY_01060	MUY_03222
MUY_01060	MUY_03263
MUY_01060	MUY_03292
MUY_01060	MUY_03434
MUY_01060	MUY_04020
MUY_01060	MUY_04022
MUY_01060	MUY_00780
MUY_01061	MUY_01061
MUY_01061	MUY_01494
MUY_01061	MUY_02015
MUY_01061	MUY_02134
MUY_01061	MUY_00241
MUY_01061	MUY_02947
MUY_01061	MUY_00277
MUY_01061	MUY_00337

MUY_01061	MUY_04086
MUY_01061	MUY_00433
MUY_01061	MUY_00813
MUY_01062	MUY_01062
MUY_01062	MUY_01492
MUY_01062	MUY_00010
MUY_01062	MUY_01719
MUY_01062	MUY_01857
MUY_01062	MUY_02376
MUY_01062	MUY_02991
MUY_01062	MUY_03222
MUY_01062	MUY_03263
MUY_01062	MUY_03292
MUY_01062	MUY_03434
MUY_01062	MUY_04020
MUY_01062	MUY_04022
MUY_01066	MUY_01066
MUY_01066	MUY_00160
MUY_01066	MUY_01993
MUY_01066	MUY_02659
MUY_00089	MUY_01313
MUY_00089	MUY_03162
MUY_00089	MUY_04035
MUY_00089	MUY_00935
MUY_01069	MUY_01069
MUY_01069	MUY_02658
MUY_01069	MUY_04307
MUY_01069	MUY_04400
MUY_01074	MUY_01074
MUY_01074	MUY_00157
MUY_01074	MUY_02820
MUY_01074	MUY_03062
MUY_01074	MUY_03703
MUY_01074	MUY_00979
MUY_01075	MUY_01075
MUY_01075	MUY_01244
MUY_01075	MUY_01609
MUY_01075	MUY_01693
MUY_01075	MUY_01735
MUY_01075	MUY_02030
MUY_01075	MUY_02361
MUY_01075	MUY_02588
MUY_01075	MUY_00297
MUY_01075	MUY_03518
MUY_01075	MUY_03667
MUY_01075	MUY_04463
MUY_01075	MUY_00557

MUY_01075	MUY_00583
MUY_01075	MUY_00674
MUY_01076	MUY_01076
MUY_01076	MUY_00165
MUY_01076	MUY_01907
MUY_01076	MUY_02518
MUY_01076	MUY_04301
MUY_01076	MUY_00698
MUY_01076	MUY_00871
MUY_01078	MUY_01078
MUY_01078	MUY_03103
MUY_01078	MUY_00082
MUY_01081	MUY_01081
MUY_01081	MUY_01344
MUY_01081	MUY_03317
MUY_01081	MUY_03934
MUY_01081	MUY_04183
MUY_01081	MUY_04335
MUY_01081	MUY_04430
MUY_01081	MUY_00847
MUY_01081	MUY_00921
MUY_00091	MUY_00091
MUY_00091	MUY_00092
MUY_01088	MUY_01088
MUY_01088	MUY_01089
MUY_01088	MUY_01095
MUY_01088	MUY_01113
MUY_01088	MUY_01130
MUY_01088	MUY_01181
MUY_01088	MUY_00110
MUY_01088	MUY_01283
MUY_01088	MUY_01284
MUY_01088	MUY_01297
MUY_01088	MUY_01298
MUY_01088	MUY_01461
MUY_01088	MUY_01466
MUY_01088	MUY_00134
MUY_01088	MUY_00152
MUY_01088	MUY_00153
MUY_01088	MUY_01652
MUY_01088	MUY_01653
MUY_01088	MUY_01656
MUY_01088	MUY_01664
MUY_01088	MUY_01762
MUY_01088	MUY_00189
MUY_01088	MUY_00194
MUY_01088	MUY_02151

MUY_01088	MUY_02208
MUY_01088	MUY_02209
MUY_01088	MUY_02225
MUY_01088	MUY_02597
MUY_01088	MUY_02598
MUY_01088	MUY_02609
MUY_01088	MUY_02643
MUY_01088	MUY_02700
MUY_01088	MUY_02744
MUY_01088	MUY_02745
MUY_01088	MUY_02760
MUY_01088	MUY_00265
MUY_01088	MUY_02894
MUY_01088	MUY_02929
MUY_01088	MUY_02945
MUY_01088	MUY_02946
MUY_01088	MUY_00285
MUY_01088	MUY_03154
MUY_01088	MUY_03330
MUY_01088	MUY_03332
MUY_01088	MUY_03354
MUY_01088	MUY_00322
MUY_01088	MUY_03575
MUY_01088	MUY_03576
MUY_01088	MUY_03582
MUY_01088	MUY_03598
MUY_01088	MUY_03634
MUY_01088	MUY_03649
MUY_01088	MUY_03676
MUY_01088	MUY_03785
MUY_01088	MUY_03805
MUY_01088	MUY_03900
MUY_01088	MUY_03946
MUY_01088	MUY_00372
MUY_01088	MUY_03978
MUY_01088	MUY_04144
MUY_01088	MUY_04172
MUY_01088	MUY_04248
MUY_01088	MUY_04254
MUY_01088	MUY_04266
MUY_01088	MUY_04269
MUY_01088	MUY_04282
MUY_01088	MUY_04296
MUY_01088	MUY_00388
MUY_01088	MUY_04348
MUY_01088	MUY_04357
MUY_01088	MUY_00394

MUY_01088	MUY_04403
MUY_01088	MUY_04416
MUY_01088	MUY_04445
MUY_01088	MUY_04453
MUY_01088	MUY_00420
MUY_01088	MUY_00434
MUY_01088	MUY_00442
MUY_01088	MUY_00516
MUY_01088	MUY_00585
MUY_01088	MUY_00800
MUY_01088	MUY_00815
MUY_01088	MUY_00937
MUY_01088	MUY_00938
MUY_01088	MUY_00939
MUY_01088	MUY_00968
MUY_01088	MUY_00975
MUY_01088	MUY_00996
MUY_01088	MUY_01000
MUY_01089	MUY_01089
MUY_01089	MUY_01095
MUY_01089	MUY_01113
MUY_01089	MUY_01130
MUY_01089	MUY_00110
MUY_01089	MUY_01283
MUY_01089	MUY_01284
MUY_01089	MUY_01297
MUY_01089	MUY_01298
MUY_01089	MUY_01461
MUY_01089	MUY_01466
MUY_01089	MUY_00134
MUY_01089	MUY_00152
MUY_01089	MUY_00153
MUY_01089	MUY_01652
MUY_01089	MUY_01653
MUY_01089	MUY_01656
MUY_01089	MUY_01664
MUY_01089	MUY_01762
MUY_01089	MUY_00189
MUY_01089	MUY_00194
MUY_01089	MUY_02151
MUY_01089	MUY_02208
MUY_01089	MUY_02209
MUY_01089	MUY_02225
MUY_01089	MUY_02597
MUY_01089	MUY_02598
MUY_01089	MUY_02609
MUY_01089	MUY_02643

MUY_01089	MUY_02700
MUY_01089	MUY_02744
MUY_01089	MUY_02745
MUY_01089	MUY_02760
MUY_01089	MUY_00265
MUY_01089	MUY_02894
MUY_01089	MUY_02929
MUY_01089	MUY_02945
MUY_01089	MUY_02946
MUY_01089	MUY_00285
MUY_01089	MUY_03154
MUY_01089	MUY_03330
MUY_01089	MUY_03332
MUY_01089	MUY_03354
MUY_01089	MUY_00322
MUY_01089	MUY_03575
MUY_01089	MUY_03576
MUY_01089	MUY_03582
MUY_01089	MUY_03598
MUY_01089	MUY_03634
MUY_01089	MUY_03649
MUY_01089	MUY_03676
MUY_01089	MUY_03785
MUY_01089	MUY_03805
MUY_01089	MUY_03900
MUY_01089	MUY_03946
MUY_01089	MUY_00372
MUY_01089	MUY_03978
MUY_01089	MUY_04144
MUY_01089	MUY_04172
MUY_01089	MUY_04248
MUY_01089	MUY_04254
MUY_01089	MUY_04266
MUY_01089	MUY_04269
MUY_01089	MUY_04282
MUY_01089	MUY_04296
MUY_01089	MUY_00388
MUY_01089	MUY_04348
MUY_01089	MUY_04357
MUY_01089	MUY_00394
MUY_01089	MUY_04403
MUY_01089	MUY_04416
MUY_01089	MUY_04445
MUY_01089	MUY_04453
MUY_01089	MUY_00420
MUY_01089	MUY_00434
MUY_01089	MUY_00442

MUY_01089	MUY_00516
MUY_01089	MUY_00585
MUY_01089	MUY_00800
MUY_01089	MUY_00815
MUY_01089	MUY_00937
MUY_01089	MUY_00938
MUY_01089	MUY_00939
MUY_01089	MUY_00968
MUY_01089	MUY_00975
MUY_01089	MUY_00996
MUY_01089	MUY_01000
MUY_01090	MUY_01090
MUY_01090	MUY_01245
MUY_01090	MUY_01973
MUY_01090	MUY_02615
MUY_01090	MUY_02633
MUY_01090	MUY_02950
MUY_01090	MUY_02958
MUY_01090	MUY_03128
MUY_01090	MUY_00313
MUY_01090	MUY_03494
MUY_01090	MUY_03606
MUY_01090	MUY_03638
MUY_01090	MUY_03964
MUY_01090	MUY_03998
MUY_01090	MUY_04045
MUY_01090	MUY_00486
MUY_01090	MUY_00681
MUY_01090	MUY_00721
MUY_01093	MUY_01093
MUY_01093	MUY_01506
MUY_01093	MUY_03242
MUY_01093	MUY_00050
MUY_00092	MUY_00092
MUY_00092	MUY_01166
MUY_00092	MUY_01302
MUY_00092	MUY_01531
MUY_00092	MUY_01847
MUY_00092	MUY_02012
MUY_00092	MUY_03091
MUY_00092	MUY_03267
MUY_00092	MUY_03844
MUY_01095	MUY_01095
MUY_01095	MUY_01113
MUY_01095	MUY_01130
MUY_01095	MUY_00110
MUY_01095	MUY_01283

MUY_01095	MUY_01284
MUY_01095	MUY_01297
MUY_01095	MUY_01298
MUY_01095	MUY_01461
MUY_01095	MUY_01466
MUY_01095	MUY_00134
MUY_01095	MUY_00152
MUY_01095	MUY_00153
MUY_01095	MUY_01652
MUY_01095	MUY_01653
MUY_01095	MUY_01656
MUY_01095	MUY_01664
MUY_01095	MUY_01762
MUY_01095	MUY_00189
MUY_01095	MUY_00194
MUY_01095	MUY_02151
MUY_01095	MUY_02208
MUY_01095	MUY_02209
MUY_01095	MUY_02225
MUY_01095	MUY_02597
MUY_01095	MUY_02598
MUY_01095	MUY_02609
MUY_01095	MUY_02643
MUY_01095	MUY_02700
MUY_01095	MUY_02744
MUY_01095	MUY_02745
MUY_01095	MUY_02760
MUY_01095	MUY_00265
MUY_01095	MUY_02894
MUY_01095	MUY_02929
MUY_01095	MUY_02945
MUY_01095	MUY_02946
MUY_01095	MUY_00285
MUY_01095	MUY_03154
MUY_01095	MUY_03330
MUY_01095	MUY_03332
MUY_01095	MUY_03354
MUY_01095	MUY_00322
MUY_01095	MUY_03575
MUY_01095	MUY_03576
MUY_01095	MUY_03582
MUY_01095	MUY_03598
MUY_01095	MUY_03634
MUY_01095	MUY_03649
MUY_01095	MUY_03676
MUY_01095	MUY_03785
MUY_01095	MUY_03805

MUY_01095	MUY_03900
MUY_01095	MUY_03946
MUY_01095	MUY_00372
MUY_01095	MUY_03978
MUY_01095	MUY_04144
MUY_01095	MUY_04172
MUY_01095	MUY_04248
MUY_01095	MUY_04254
MUY_01095	MUY_04266
MUY_01095	MUY_04269
MUY_01095	MUY_04282
MUY_01095	MUY_04296
MUY_01095	MUY_00388
MUY_01095	MUY_04348
MUY_01095	MUY_04357
MUY_01095	MUY_00394
MUY_01095	MUY_04403
MUY_01095	MUY_04416
MUY_01095	MUY_04445
MUY_01095	MUY_04453
MUY_01095	MUY_00420
MUY_01095	MUY_00434
MUY_01095	MUY_00442
MUY_01095	MUY_00516
MUY_01095	MUY_00585
MUY_01095	MUY_00800
MUY_01095	MUY_00815
MUY_01095	MUY_00937
MUY_01095	MUY_00938
MUY_01095	MUY_00939
MUY_01095	MUY_00968
MUY_01095	MUY_00975
MUY_01095	MUY_00996
MUY_01095	MUY_01000
MUY_01096	MUY_01096
MUY_01103	MUY_01103
MUY_01103	MUY_01260
MUY_01103	MUY_01676
MUY_01103	MUY_03969
MUY_01103	MUY_03995
MUY_01103	MUY_04080
MUY_01103	MUY_04404
MUY_01103	MUY_00474
MUY_01106	MUY_01106
MUY_01106	MUY_01322
MUY_01106	MUY_01671
MUY_01106	MUY_01955

MUY_01106	MUY_00218
MUY_01106	MUY_03393
MUY_01106	MUY_03438
MUY_01108	MUY_01108
MUY_01108	MUY_01203
MUY_01108	MUY_01308
MUY_01108	MUY_01432
MUY_01108	MUY_01874
MUY_01108	MUY_02138
MUY_01108	MUY_02139
MUY_01108	MUY_00205
MUY_01108	MUY_02178
MUY_01108	MUY_02521
MUY_01108	MUY_02523
MUY_01108	MUY_00249
MUY_01108	MUY_02663
MUY_01108	MUY_00263
MUY_01108	MUY_02912
MUY_01108	MUY_02940
MUY_01108	MUY_00288
MUY_01108	MUY_03139
MUY_01108	MUY_03183
MUY_01108	MUY_03202
MUY_01108	MUY_03374
MUY_01108	MUY_03463
MUY_01108	MUY_03477
MUY_01108	MUY_03591
MUY_01108	MUY_03592
MUY_01108	MUY_03609
MUY_01108	MUY_03619
MUY_01108	MUY_03648
MUY_01108	MUY_03924
MUY_01108	MUY_04096
MUY_01108	MUY_04105
MUY_01108	MUY_04106
MUY_01108	MUY_04146
MUY_01108	MUY_04177
MUY_01108	MUY_04212
MUY_01108	MUY_04293
MUY_01108	MUY_04359
MUY_01108	MUY_04418
MUY_01108	MUY_04448
MUY_01108	MUY_04471
MUY_01108	MUY_00418
MUY_01108	MUY_00436
MUY_01108	MUY_00576
MUY_01109	MUY_01109

MUY_01109	MUY_01203
MUY_01109	MUY_01308
MUY_01109	MUY_01432
MUY_01109	MUY_01582
MUY_01109	MUY_01874
MUY_01109	MUY_00205
MUY_01109	MUY_02521
MUY_01109	MUY_02523
MUY_01109	MUY_00249
MUY_01109	MUY_02663
MUY_01109	MUY_00263
MUY_01109	MUY_02912
MUY_01109	MUY_02940
MUY_01109	MUY_03012
MUY_01109	MUY_00288
MUY_01109	MUY_03117
MUY_01109	MUY_03183
MUY_01109	MUY_03202
MUY_01109	MUY_03306
MUY_01109	MUY_03463
MUY_01109	MUY_03477
MUY_01109	MUY_03609
MUY_01109	MUY_03619
MUY_01109	MUY_03648
MUY_01109	MUY_03863
MUY_01109	MUY_03924
MUY_01109	MUY_04096
MUY_01109	MUY_04146
MUY_01109	MUY_04177
MUY_01109	MUY_04212
MUY_01109	MUY_04293
MUY_01109	MUY_04359
MUY_01109	MUY_04418
MUY_01109	MUY_04448
MUY_01109	MUY_04471
MUY_01109	MUY_00418
MUY_01109	MUY_00436
MUY_01109	MUY_00576
MUY_01109	MUY_00790
MUY_01109	MUY_00961
MUY_01113	MUY_01113
MUY_01113	MUY_01130
MUY_01113	MUY_00110
MUY_01113	MUY_01283
MUY_01113	MUY_01284
MUY_01113	MUY_01297
MUY_01113	MUY_01298

MUY_01113	MUY_01461
MUY_01113	MUY_01466
MUY_01113	MUY_00134
MUY_01113	MUY_00152
MUY_01113	MUY_00153
MUY_01113	MUY_01652
MUY_01113	MUY_01653
MUY_01113	MUY_01656
MUY_01113	MUY_01664
MUY_01113	MUY_01762
MUY_01113	MUY_00189
MUY_01113	MUY_00194
MUY_01113	MUY_02151
MUY_01113	MUY_02208
MUY_01113	MUY_02209
MUY_01113	MUY_02225
MUY_01113	MUY_02597
MUY_01113	MUY_02598
MUY_01113	MUY_02609
MUY_01113	MUY_02643
MUY_01113	MUY_02700
MUY_01113	MUY_02744
MUY_01113	MUY_02745
MUY_01113	MUY_02760
MUY_01113	MUY_00265
MUY_01113	MUY_02894
MUY_01113	MUY_02929
MUY_01113	MUY_02945
MUY_01113	MUY_02946
MUY_01113	MUY_00285
MUY_01113	MUY_03154
MUY_01113	MUY_03330
MUY_01113	MUY_03332
MUY_01113	MUY_03354
MUY_01113	MUY_00322
MUY_01113	MUY_03575
MUY_01113	MUY_03576
MUY_01113	MUY_03582
MUY_01113	MUY_03598
MUY_01113	MUY_03634
MUY_01113	MUY_03649
MUY_01113	MUY_03676
MUY_01113	MUY_03785
MUY_01113	MUY_03805
MUY_01113	MUY_03900
MUY_01113	MUY_03946
MUY_01113	MUY_00372

MUY_01113	MUY_03978
MUY_01113	MUY_04144
MUY_01113	MUY_04172
MUY_01113	MUY_04248
MUY_01113	MUY_04254
MUY_01113	MUY_04266
MUY_01113	MUY_04269
MUY_01113	MUY_04282
MUY_01113	MUY_04296
MUY_01113	MUY_00388
MUY_01113	MUY_04348
MUY_01113	MUY_04357
MUY_01113	MUY_00394
MUY_01113	MUY_04403
MUY_01113	MUY_04416
MUY_01113	MUY_04445
MUY_01113	MUY_04453
MUY_01113	MUY_00420
MUY_01113	MUY_00434
MUY_01113	MUY_00442
MUY_01113	MUY_00516
MUY_01113	MUY_00585
MUY_01113	MUY_00800
MUY_01113	MUY_00815
MUY_01113	MUY_00937
MUY_01113	MUY_00938
MUY_01113	MUY_00939
MUY_01113	MUY_00968
MUY_01113	MUY_00975
MUY_01113	MUY_00996
MUY_01113	MUY_01000
MUY_01114	MUY_01114
MUY_01114	MUY_01141
MUY_01114	MUY_01415
MUY_01114	MUY_01437
MUY_01114	MUY_01469
MUY_01114	MUY_01660
MUY_01114	MUY_01666
MUY_01114	MUY_01712
MUY_01114	MUY_01740
MUY_01114	MUY_01741
MUY_01114	MUY_01750
MUY_01114	MUY_01754
MUY_01114	MUY_01816
MUY_01114	MUY_01825
MUY_01114	MUY_01878
MUY_01114	MUY_01978

MUY_01114	MUY_00206
MUY_01114	MUY_02198
MUY_01114	MUY_00231
MUY_01114	MUY_00255
MUY_01114	MUY_00256
MUY_01114	MUY_02780
MUY_01114	MUY_02797
MUY_01114	MUY_02983
MUY_01114	MUY_00283
MUY_01114	MUY_03069
MUY_01114	MUY_03314
MUY_01114	MUY_03425
MUY_01114	MUY_00329
MUY_01114	MUY_03535
MUY_01114	MUY_03610
MUY_01114	MUY_03789
MUY_01114	MUY_03863
MUY_01114	MUY_03967
MUY_01114	MUY_04086
MUY_01114	MUY_04334
MUY_01114	MUY_04341
MUY_01114	MUY_04466
MUY_01114	MUY_00435
MUY_01114	MUY_00514
MUY_01114	MUY_00590
MUY_01114	MUY_00698
MUY_01114	MUY_00068
MUY_01114	MUY_00780
MUY_01115	MUY_01115
MUY_01115	MUY_01116
MUY_01115	MUY_01624
MUY_01115	MUY_02506
MUY_01115	MUY_03121
MUY_01115	MUY_03418
MUY_01116	MUY_01740
MUY_01116	MUY_01750
MUY_01116	MUY_01754
MUY_01116	MUY_00206
MUY_01116	MUY_00231
MUY_01116	MUY_02785
MUY_01116	MUY_03069
MUY_01116	MUY_03070
MUY_01116	MUY_04334
MUY_01116	MUY_00068
MUY_01117	MUY_01117
MUY_01117	MUY_02233
MUY_01117	MUY_02448

MUY_01117	MUY_02797
MUY_01117	MUY_02829
MUY_01117	MUY_03251
MUY_01117	MUY_03314
MUY_01117	MUY_03847
MUY_01117	MUY_04122
MUY_01117	MUY_04476
MUY_01118	MUY_04501
MUY_01119	MUY_01754
MUY_01119	MUY_03267
MUY_01119	MUY_04476
MUY_00093	MUY_00093
MUY_01123	MUY_02671
MUY_01124	MUY_01124
MUY_01124	MUY_01482
MUY_01124	MUY_01497
MUY_01124	MUY_01528
MUY_01124	MUY_01701
MUY_01124	MUY_02050
MUY_01124	MUY_02216
MUY_01124	MUY_02232
MUY_01124	MUY_02364
MUY_01124	MUY_02695
MUY_01124	MUY_02708
MUY_01124	MUY_00272
MUY_01124	MUY_03126
MUY_01124	MUY_03240
MUY_01124	MUY_03659
MUY_01124	MUY_03701
MUY_01124	MUY_03880
MUY_01124	MUY_04014
MUY_01124	MUY_04194
MUY_01124	MUY_00556
MUY_01124	MUY_00710
MUY_01128	MUY_01128
MUY_01128	MUY_01987
MUY_01128	MUY_02916
MUY_01128	MUY_02918
MUY_01128	MUY_03014
MUY_01128	MUY_03054
MUY_01128	MUY_03244
MUY_01128	MUY_03556
MUY_01128	MUY_03573
MUY_01128	MUY_03574
MUY_01128	MUY_03660
MUY_01128	MUY_00766
MUY_01129	MUY_01129

MUY_01129	MUY_02827
MUY_01130	MUY_01130
MUY_01130	MUY_00110
MUY_01130	MUY_01283
MUY_01130	MUY_01284
MUY_01130	MUY_01297
MUY_01130	MUY_01298
MUY_01130	MUY_01461
MUY_01130	MUY_01466
MUY_01130	MUY_00134
MUY_01130	MUY_00152
MUY_01130	MUY_00153
MUY_01130	MUY_01652
MUY_01130	MUY_01653
MUY_01130	MUY_01656
MUY_01130	MUY_01664
MUY_01130	MUY_01762
MUY_01130	MUY_00189
MUY_01130	MUY_00194
MUY_01130	MUY_02151
MUY_01130	MUY_02208
MUY_01130	MUY_02209
MUY_01130	MUY_02225
MUY_01130	MUY_02597
MUY_01130	MUY_02598
MUY_01130	MUY_02609
MUY_01130	MUY_02643
MUY_01130	MUY_02700
MUY_01130	MUY_02744
MUY_01130	MUY_02745
MUY_01130	MUY_02760
MUY_01130	MUY_00265
MUY_01130	MUY_02894
MUY_01130	MUY_02929
MUY_01130	MUY_02945
MUY_01130	MUY_02946
MUY_01130	MUY_00285
MUY_01130	MUY_03154
MUY_01130	MUY_03330
MUY_01130	MUY_03332
MUY_01130	MUY_03354
MUY_01130	MUY_00322
MUY_01130	MUY_03575
MUY_01130	MUY_03576
MUY_01130	MUY_03582
MUY_01130	MUY_03598
MUY_01130	MUY_03634

MUY_01130	MUY_03649
MUY_01130	MUY_03676
MUY_01130	MUY_03785
MUY_01130	MUY_03805
MUY_01130	MUY_03900
MUY_01130	MUY_03946
MUY_01130	MUY_00372
MUY_01130	MUY_03978
MUY_01130	MUY_04144
MUY_01130	MUY_04172
MUY_01130	MUY_04248
MUY_01130	MUY_04254
MUY_01130	MUY_04266
MUY_01130	MUY_04269
MUY_01130	MUY_04282
MUY_01130	MUY_04296
MUY_01130	MUY_00388
MUY_01130	MUY_04348
MUY_01130	MUY_04357
MUY_01130	MUY_00394
MUY_01130	MUY_04403
MUY_01130	MUY_04416
MUY_01130	MUY_04445
MUY_01130	MUY_04453
MUY_01130	MUY_00420
MUY_01130	MUY_00434
MUY_01130	MUY_00442
MUY_01130	MUY_00516
MUY_01130	MUY_00585
MUY_01130	MUY_00800
MUY_01130	MUY_00815
MUY_01130	MUY_00937
MUY_01130	MUY_00938
MUY_01130	MUY_00939
MUY_01130	MUY_00968
MUY_01130	MUY_00975
MUY_01130	MUY_00996
MUY_01130	MUY_01000
MUY_01135	MUY_01135
MUY_01135	MUY_01943
MUY_01135	MUY_02589
MUY_01136	MUY_01136
MUY_01136	MUY_02434
MUY_01136	MUY_00251
MUY_01136	MUY_03071
MUY_01136	MUY_03459
MUY_01136	MUY_04116

MUY_00094	MUY_00094
MUY_00094	MUY_00183
MUY_01137	MUY_01137
MUY_01137	MUY_00747
MUY_01138	MUY_01138
MUY_01138	MUY_04264
MUY_01139	MUY_01139
MUY_01139	MUY_01952
MUY_01139	MUY_01953
MUY_01139	MUY_03567
MUY_01140	MUY_01140
MUY_01140	MUY_01374
MUY_01140	MUY_01970
MUY_01140	MUY_01999
MUY_01140	MUY_02036
MUY_01140	MUY_02049
MUY_01140	MUY_02231
MUY_01140	MUY_02238
MUY_01140	MUY_02297
MUY_01140	MUY_02580
MUY_01140	MUY_02982
MUY_01140	MUY_03140
MUY_01140	MUY_03250
MUY_01140	MUY_03431
MUY_01140	MUY_00323
MUY_01140	MUY_03553
MUY_01140	MUY_03612
MUY_01140	MUY_00445
MUY_01140	MUY_00677
MUY_01140	MUY_00749
MUY_01140	MUY_00779
MUY_01140	MUY_00882
MUY_01141	MUY_01141
MUY_01141	MUY_01415
MUY_01141	MUY_01437
MUY_01141	MUY_01469
MUY_01141	MUY_01660
MUY_01141	MUY_01740
MUY_01141	MUY_01741
MUY_01141	MUY_01750
MUY_01141	MUY_01754
MUY_01141	MUY_01825
MUY_01141	MUY_01866
MUY_01141	MUY_00206
MUY_01141	MUY_02198
MUY_01141	MUY_00231
MUY_01141	MUY_02780

MUY_01141	MUY_02797
MUY_01141	MUY_03069
MUY_01141	MUY_03314
MUY_01141	MUY_03610
MUY_01141	MUY_03967
MUY_01141	MUY_04086
MUY_01141	MUY_00381
MUY_01141	MUY_04334
MUY_01141	MUY_04466
MUY_01141	MUY_00435
MUY_01141	MUY_00514
MUY_01141	MUY_00068
MUY_01141	MUY_00780
MUY_01141	MUY_00075
MUY_01142	MUY_01142
MUY_01142	MUY_01279
1129957..1130994	MUY_03567
MUY_01148	MUY_01148
MUY_01148	MUY_01673
MUY_01148	MUY_01986
MUY_01148	MUY_02136
MUY_01148	MUY_02622
MUY_01148	MUY_03257
MUY_01149	MUY_01149
MUY_01149	MUY_01246
MUY_01149	MUY_01353
MUY_01149	MUY_02149
MUY_01149	MUY_02242
MUY_01149	MUY_02316
MUY_01149	MUY_02447
MUY_01149	MUY_02696
MUY_01149	MUY_02788
MUY_01149	MUY_03055
MUY_01149	MUY_03267
MUY_01149	MUY_04126
MUY_01149	MUY_04239
MUY_01149	MUY_00469
MUY_01149	MUY_00485
MUY_01149	MUY_00696
MUY_01150	MUY_01150
MUY_01150	MUY_01198
MUY_01150	MUY_02176
MUY_01150	MUY_02182
MUY_01150	MUY_02410
MUY_01150	MUY_00273
MUY_01150	MUY_03141
MUY_01150	MUY_03241

MUY_01150	MUY_03261
MUY_01150	MUY_03373
MUY_01150	MUY_00376
MUY_01150	MUY_04033
MUY_01150	MUY_04034
MUY_01150	MUY_04036
MUY_01150	MUY_00377
MUY_01150	MUY_00378
MUY_01150	MUY_04228
MUY_01150	MUY_04230
MUY_01150	MUY_00379
MUY_01152	MUY_01152
MUY_01152	MUY_01382
MUY_01152	MUY_02707
MUY_01152	MUY_02708
MUY_01152	MUY_02820
MUY_01152	MUY_02882
MUY_01152	MUY_02883
MUY_01152	MUY_02889
MUY_01152	MUY_02892
MUY_01152	MUY_02900
MUY_01152	MUY_03708
MUY_01152	MUY_03769
MUY_01152	MUY_03778
MUY_01152	MUY_03830
MUY_01152	MUY_04185
MUY_01152	MUY_04271
MUY_01152	MUY_04291
MUY_01152	MUY_04501
MUY_01152	MUY_04505
MUY_01152	MUY_00551
MUY_01152	MUY_00599
MUY_01152	MUY_00602
MUY_01152	MUY_00628
MUY_01152	MUY_00691
MUY_01152	MUY_00795
MUY_01152	MUY_00086
MUY_01152	MUY_00973
MUY_01155	MUY_01155
MUY_01156	MUY_01156
MUY_01156	MUY_01490
MUY_01156	MUY_01756
MUY_01156	MUY_02229
MUY_01156	MUY_04159
MUY_01158	MUY_01158
MUY_01158	MUY_02730
MUY_01158	MUY_02977

MUY_01158	MUY_03601
MUY_01158	MUY_03792
MUY_01158	MUY_04042
MUY_01158	MUY_04408
MUY_01158	MUY_00443
MUY_00097	MUY_00097
MUY_00097	MUY_00098
MUY_01161	MUY_01161
MUY_01161	MUY_01176
MUY_01161	MUY_01486
MUY_01161	MUY_02295
MUY_01161	MUY_02578
MUY_01161	MUY_03356
MUY_01161	MUY_03860
MUY_01161	MUY_04473
MUY_01162	MUY_03425
MUY_01163	MUY_01163
MUY_01163	MUY_01683
MUY_01163	MUY_02127
MUY_01163	MUY_02171
MUY_01163	MUY_02269
MUY_01163	MUY_02703
MUY_01163	MUY_02841
MUY_01163	MUY_02941
MUY_01163	MUY_03513
MUY_01163	MUY_03515
MUY_01163	MUY_03524
MUY_01163	MUY_00461
MUY_01164	MUY_01164
MUY_01164	MUY_01245
MUY_01164	MUY_01280
MUY_01164	MUY_01329
MUY_01164	MUY_01421
MUY_01164	MUY_01611
MUY_01164	MUY_01625
MUY_01164	MUY_01923
MUY_01164	MUY_02100
MUY_01164	MUY_02153
MUY_01164	MUY_02211
MUY_01164	MUY_02331
MUY_01164	MUY_02615
MUY_01164	MUY_02633
MUY_01164	MUY_02950
MUY_01164	MUY_02958
MUY_01164	MUY_00292
MUY_01164	MUY_03128
MUY_01164	MUY_03225

MUY_01164	MUY_00313
MUY_01164	MUY_03494
MUY_01164	MUY_03606
MUY_01164	MUY_03627
MUY_01164	MUY_03638
MUY_01164	MUY_03964
MUY_01164	MUY_03998
MUY_01164	MUY_04038
MUY_01164	MUY_00486
MUY_01164	MUY_00681
MUY_01164	MUY_00933
MUY_01165	MUY_01165
MUY_00098	MUY_00098
MUY_01169	MUY_01169
MUY_01169	MUY_01661
MUY_01169	MUY_02711
MUY_01169	MUY_00719
MUY_00099	MUY_00099
MUY_00099	MUY_03267
MUY_00099	MUY_00775
MUY_01174	MUY_01232
MUY_01174	MUY_00344
MUY_01175	MUY_01175
MUY_01175	MUY_01639
MUY_01175	MUY_02355
MUY_01175	MUY_02618
MUY_01175	MUY_03173
MUY_01175	MUY_03557
MUY_01176	MUY_01176
MUY_01176	MUY_01230
MUY_01176	MUY_01523
MUY_01176	MUY_00344
MUY_01176	MUY_03721
MUY_01176	MUY_00512
MUY_01177	MUY_01177
MUY_01177	MUY_01178
MUY_01178	MUY_01178
MUY_01178	MUY_00344
MUY_01181	MUY_01181
MUY_01189	MUY_01189
MUY_01189	MUY_01232
MUY_01189	MUY_01602
MUY_01189	MUY_01936
MUY_01189	MUY_02159
MUY_01189	MUY_03267
MUY_01189	MUY_03644
MUY_01189	MUY_03962

MUY_01189	MUY_03975
MUY_01189	MUY_04082
MUY_01189	MUY_04328
MUY_01189	MUY_04428
MUY_01189	MUY_00700
MUY_01193	MUY_01193
MUY_01193	MUY_00307
MUY_01193	MUY_00344
MUY_01193	MUY_04235
MUY_01193	MUY_00899
MUY_01194	MUY_02175
MUY_01195	MUY_01197
MUY_00101	MUY_00101
MUY_00101	MUY_03267
MUY_01197	MUY_01197
MUY_01197	MUY_02045
MUY_01197	MUY_04018
MUY_01198	MUY_01198
MUY_01198	MUY_02176
MUY_01198	MUY_02182
MUY_01198	MUY_02410
MUY_01198	MUY_00273
MUY_01198	MUY_03141
MUY_01198	MUY_03241
MUY_01198	MUY_03261
MUY_01198	MUY_03373
MUY_01198	MUY_00376
MUY_01198	MUY_04033
MUY_01198	MUY_04034
MUY_01198	MUY_04036
MUY_01198	MUY_00377
MUY_01198	MUY_00378
MUY_01198	MUY_04228
MUY_01198	MUY_04230
MUY_01198	MUY_00379
MUY_01203	MUY_01203
MUY_01203	MUY_01308
MUY_01203	MUY_01432
MUY_01203	MUY_01527
MUY_01203	MUY_01582
MUY_01203	MUY_01626
MUY_01203	MUY_01670
MUY_01203	MUY_01874
MUY_01203	MUY_01875
MUY_01203	MUY_00205
MUY_01203	MUY_02178
MUY_01203	MUY_02366

MUY_01203	MUY_00229
MUY_01203	MUY_02521
MUY_01203	MUY_02523
MUY_01203	MUY_00249
MUY_01203	MUY_02655
MUY_01203	MUY_02663
MUY_01203	MUY_00263
MUY_01203	MUY_00264
MUY_01203	MUY_02912
MUY_01203	MUY_02939
MUY_01203	MUY_02940
MUY_01203	MUY_02964
MUY_01203	MUY_00288
MUY_01203	MUY_00289
MUY_01203	MUY_03059
MUY_01203	MUY_03117
MUY_01203	MUY_03127
MUY_01203	MUY_03139
MUY_01203	MUY_03183
MUY_01203	MUY_03201
MUY_01203	MUY_03202
MUY_01203	MUY_03252
MUY_01203	MUY_03306
MUY_01203	MUY_03374
MUY_01203	MUY_03463
MUY_01203	MUY_03477
MUY_01203	MUY_03478
MUY_01203	MUY_03483
MUY_01203	MUY_03609
MUY_01203	MUY_03619
MUY_01203	MUY_03647
MUY_01203	MUY_03648
MUY_01203	MUY_03924
MUY_01203	MUY_04096
MUY_01203	MUY_04146
MUY_01203	MUY_04177
MUY_01203	MUY_04211
MUY_01203	MUY_04212
MUY_01203	MUY_04293
MUY_01203	MUY_04294
MUY_01203	MUY_04358
MUY_01203	MUY_04359
MUY_01203	MUY_04379
MUY_01203	MUY_04417
MUY_01203	MUY_04418
MUY_01203	MUY_04447
MUY_01203	MUY_04448

MUY_01203	MUY_04471
MUY_01203	MUY_00418
MUY_01203	MUY_00436
MUY_01203	MUY_00575
MUY_01203	MUY_00576
MUY_01203	MUY_00790
MUY_01203	MUY_00961
MUY_01204	MUY_01204
MUY_01204	MUY_03720
MUY_01205	MUY_01205
MUY_01205	MUY_03580
MUY_01205	MUY_00371
MUY_01205	MUY_00702
MUY_01207	MUY_01207
MUY_01207	MUY_01208
MUY_01207	MUY_01341
MUY_01207	MUY_02534
MUY_01207	MUY_00758
MUY_00102	MUY_00102
MUY_00102	MUY_00745
MUY_01208	MUY_01208
MUY_01208	MUY_01341
MUY_01208	MUY_00758
MUY_00103	MUY_01781
MUY_00103	MUY_02514
MUY_00103	MUY_00983
MUY_01209	MUY_01209
MUY_01209	MUY_01321
MUY_01209	MUY_01888
MUY_01209	MUY_01959
MUY_01209	MUY_02025
MUY_01209	MUY_02289
MUY_01209	MUY_02548
MUY_01209	MUY_02620
MUY_01209	MUY_03012
MUY_01209	MUY_03341
MUY_01209	MUY_03375
MUY_01209	MUY_03403
MUY_01209	MUY_03451
MUY_01209	MUY_03775
MUY_01209	MUY_00005
MUY_01209	MUY_03863
MUY_01209	MUY_00526
MUY_01209	MUY_00857
MUY_01210	MUY_02785
MUY_01210	MUY_03425
MUY_01218	MUY_01218

MUY_01218	MUY_01526
MUY_01221	MUY_04136
MUY_01223	MUY_01223
MUY_01225	MUY_02814
MUY_01225	MUY_02821
MUY_01225	MUY_03925
MUY_01226	MUY_01226
MUY_01226	MUY_01786
MUY_01226	MUY_02413
MUY_01226	MUY_03025
MUY_01226	MUY_03345
MUY_01226	MUY_04174
MUY_01226	MUY_00074
MUY_00105	MUY_00105
MUY_00105	MUY_00114
MUY_00105	MUY_00115
MUY_00105	MUY_01390
MUY_00105	MUY_01504
MUY_00105	MUY_01696
MUY_00105	MUY_01760
MUY_00105	MUY_01761
MUY_00105	MUY_01879
MUY_00105	MUY_00181
MUY_00105	MUY_00182
MUY_00105	MUY_02516
MUY_00105	MUY_02560
MUY_00105	MUY_02784
MUY_00105	MUY_02845
MUY_00105	MUY_02852
MUY_00105	MUY_02889
MUY_00105	MUY_02892
MUY_00105	MUY_02900
MUY_00105	MUY_03708
MUY_00105	MUY_03918
MUY_00105	MUY_04026
MUY_00105	MUY_04258
MUY_00105	MUY_04322
MUY_00105	MUY_04504
MUY_00105	MUY_00541
MUY_00105	MUY_00571
MUY_00105	MUY_00628
MUY_01228	MUY_01228
MUY_01228	MUY_01265
MUY_01228	MUY_01479
MUY_01228	MUY_02187
MUY_01228	MUY_03406
MUY_01228	MUY_04317

MUY_01228	MUY_00439
MUY_01230	MUY_01230
MUY_01230	MUY_01523
MUY_01230	MUY_03721
MUY_01230	MUY_00512
MUY_01231	MUY_01231
MUY_01231	MUY_01370
MUY_01231	MUY_01524
MUY_01231	MUY_02039
MUY_01231	MUY_02115
MUY_01231	MUY_02118
MUY_01231	MUY_02124
MUY_01231	MUY_02307
MUY_01231	MUY_02411
MUY_01231	MUY_02574
MUY_01231	MUY_00250
MUY_01231	MUY_03442
MUY_01231	MUY_04171
MUY_01231	MUY_04318
MUY_01231	MUY_04410
MUY_01231	MUY_00504
MUY_01231	MUY_00782
MUY_01231	MUY_00920
MUY_01232	MUY_01232
MUY_01232	MUY_01602
MUY_01232	MUY_02159
MUY_01232	MUY_03267
MUY_01232	MUY_03644
MUY_01232	MUY_03962
MUY_01232	MUY_03975
MUY_01232	MUY_04082
MUY_01232	MUY_04219
MUY_01232	MUY_04328
MUY_01232	MUY_04428
MUY_01232	MUY_00700
MUY_01233	MUY_01233
MUY_01233	MUY_03310
MUY_01233	MUY_03422
MUY_01233	MUY_03703
MUY_01233	MUY_04079
MUY_01233	MUY_04391
MUY_01233	MUY_00852
MUY_00108	MUY_00127
MUY_00108	MUY_00979
MUY_01237	MUY_01237
MUY_01237	MUY_01418
MUY_01237	MUY_01520

MUY_01237	MUY_01616
MUY_01237	MUY_01919
MUY_01237	MUY_01937
MUY_01237	MUY_01989
MUY_01237	MUY_00190
MUY_01237	MUY_02094
MUY_01237	MUY_02152
MUY_01237	MUY_02439
MUY_01237	MUY_02467
MUY_01237	MUY_02627
MUY_01237	MUY_00261
MUY_01237	MUY_00308
MUY_01237	MUY_03333
MUY_01237	MUY_03386
MUY_01237	MUY_03449
MUY_01237	MUY_03454
MUY_01237	MUY_03637
MUY_01237	MUY_03810
MUY_01237	MUY_04070
MUY_01237	MUY_04242
MUY_01237	MUY_00382
MUY_01237	MUY_04302
MUY_01237	MUY_04383
MUY_01237	MUY_04433
MUY_01237	MUY_04483
MUY_01237	MUY_00450
MUY_01237	MUY_00464
MUY_01237	MUY_00808
MUY_01237	MUY_00840
MUY_01238	MUY_01238
MUY_01238	MUY_01266
MUY_01238	MUY_02358
MUY_01238	MUY_03082
MUY_01238	MUY_00398
MUY_01238	MUY_00451
MUY_01238	MUY_00807
MUY_01238	MUY_00809
MUY_01238	MUY_00944
MUY_01239	MUY_01239
MUY_01239	MUY_00392
MUY_01240	MUY_01240
MUY_01240	MUY_01243
MUY_01240	MUY_03597
MUY_01241	MUY_01241
MUY_01241	MUY_03864
MUY_00109	MUY_00109
MUY_00109	MUY_02810

MUY_01242	MUY_01242
MUY_01243	MUY_01243
MUY_01243	MUY_03597
MUY_01244	MUY_01244
MUY_01244	MUY_01609
MUY_01244	MUY_01693
MUY_01244	MUY_01735
MUY_01244	MUY_02030
MUY_01244	MUY_02361
MUY_01244	MUY_02588
MUY_01244	MUY_00297
MUY_01244	MUY_03518
MUY_01244	MUY_03667
MUY_01244	MUY_04463
MUY_01244	MUY_00557
MUY_01244	MUY_00583
MUY_01244	MUY_00674
MUY_01245	MUY_01245
MUY_01245	MUY_01329
MUY_01245	MUY_01421
MUY_01245	MUY_01611
MUY_01245	MUY_01625
MUY_01245	MUY_01923
MUY_01245	MUY_01973
MUY_01245	MUY_02100
MUY_01245	MUY_02153
MUY_01245	MUY_02211
MUY_01245	MUY_02331
MUY_01245	MUY_02615
MUY_01245	MUY_02633
MUY_01245	MUY_02950
MUY_01245	MUY_02958
MUY_01245	MUY_00292
MUY_01245	MUY_03128
MUY_01245	MUY_03225
MUY_01245	MUY_00313
MUY_01245	MUY_03494
MUY_01245	MUY_03606
MUY_01245	MUY_03627
MUY_01245	MUY_03638
MUY_01245	MUY_03964
MUY_01245	MUY_03998
MUY_01245	MUY_04038
MUY_01245	MUY_04045
MUY_01245	MUY_00486
MUY_01245	MUY_00681
MUY_01245	MUY_00721

MUY_01245	MUY_00933
MUY_01246	MUY_01246
MUY_01246	MUY_01353
MUY_01246	MUY_02149
MUY_01246	MUY_02242
MUY_01246	MUY_02447
MUY_01246	MUY_02696
MUY_01246	MUY_02788
MUY_01246	MUY_03055
MUY_01246	MUY_04126
MUY_01246	MUY_04239
MUY_01246	MUY_00469
MUY_01246	MUY_00485
MUY_01246	MUY_00696
MUY_00110	MUY_00110
MUY_00110	MUY_01283
MUY_00110	MUY_01284
MUY_00110	MUY_01297
MUY_00110	MUY_01298
MUY_00110	MUY_01461
MUY_00110	MUY_01466
MUY_00110	MUY_00134
MUY_00110	MUY_00152
MUY_00110	MUY_00153
MUY_00110	MUY_01652
MUY_00110	MUY_01653
MUY_00110	MUY_01656
MUY_00110	MUY_01664
MUY_00110	MUY_01762
MUY_00110	MUY_00189
MUY_00110	MUY_00194
MUY_00110	MUY_02151
MUY_00110	MUY_02208
MUY_00110	MUY_02209
MUY_00110	MUY_02225
MUY_00110	MUY_02597
MUY_00110	MUY_02598
MUY_00110	MUY_02609
MUY_00110	MUY_02643
MUY_00110	MUY_02700
MUY_00110	MUY_02744
MUY_00110	MUY_02745
MUY_00110	MUY_02760
MUY_00110	MUY_00265
MUY_00110	MUY_02894
MUY_00110	MUY_02929
MUY_00110	MUY_02945

MUY_00110	MUY_02946
MUY_00110	MUY_00285
MUY_00110	MUY_03089
MUY_00110	MUY_03154
MUY_00110	MUY_03267
MUY_00110	MUY_03330
MUY_00110	MUY_03332
MUY_00110	MUY_03354
MUY_00110	MUY_00322
MUY_00110	MUY_03576
MUY_00110	MUY_03582
MUY_00110	MUY_03598
MUY_00110	MUY_03634
MUY_00110	MUY_03649
MUY_00110	MUY_03676
MUY_00110	MUY_03785
MUY_00110	MUY_03805
MUY_00110	MUY_03900
MUY_00110	MUY_03946
MUY_00110	MUY_00372
MUY_00110	MUY_03978
MUY_00110	MUY_04144
MUY_00110	MUY_04172
MUY_00110	MUY_04248
MUY_00110	MUY_04254
MUY_00110	MUY_04266
MUY_00110	MUY_04269
MUY_00110	MUY_04282
MUY_00110	MUY_04296
MUY_00110	MUY_00388
MUY_00110	MUY_04348
MUY_00110	MUY_04357
MUY_00110	MUY_00394
MUY_00110	MUY_04403
MUY_00110	MUY_04416
MUY_00110	MUY_04445
MUY_00110	MUY_04453
MUY_00110	MUY_00420
MUY_00110	MUY_00434
MUY_00110	MUY_00442
MUY_00110	MUY_00516
MUY_00110	MUY_00585
MUY_00110	MUY_00800
MUY_00110	MUY_00815
MUY_00110	MUY_00937
MUY_00110	MUY_00938
MUY_00110	MUY_00939

MUY_00110	MUY_00968
MUY_00110	MUY_00975
MUY_00110	MUY_00996
MUY_00110	MUY_01000
MUY_01247	MUY_01247
MUY_01247	MUY_01248
MUY_01247	MUY_00238
MUY_01247	MUY_04245
MUY_01247	MUY_00083
MUY_01248	MUY_01248
MUY_01248	MUY_00238
MUY_01248	MUY_00083
MUY_01249	MUY_01249
MUY_01254	MUY_01254
MUY_01254	MUY_02373
MUY_01254	MUY_03923
MUY_01255	MUY_01255
MUY_01255	MUY_02454
MUY_01255	MUY_02762
MUY_01255	MUY_03902
MUY_01255	MUY_04312
MUY_01255	MUY_00561
MUY_01255	MUY_00734
MUY_00111	MUY_03089
MUY_00111	MUY_03267
MUY_01256	MUY_02983
MUY_01257	MUY_01257
MUY_01260	MUY_01260
MUY_01260	MUY_01676
MUY_01260	MUY_03969
MUY_01260	MUY_03995
MUY_01260	MUY_04080
MUY_01260	MUY_04404
MUY_01260	MUY_00474
MUY_01261	MUY_01261
MUY_01261	MUY_02334
MUY_01261	MUY_03318
MUY_01261	MUY_03491
MUY_01261	MUY_00854
MUY_00112	MUY_03089
MUY_00112	MUY_03267
MUY_01263	MUY_01263
MUY_01263	MUY_01912
MUY_01263	MUY_02179
MUY_01263	MUY_00256
MUY_01263	MUY_03099
MUY_01263	MUY_03267

MUY_01264	MUY_03267
MUY_01265	MUY_01265
MUY_01265	MUY_01477
MUY_01265	MUY_01479
MUY_01265	MUY_01913
MUY_01265	MUY_02187
MUY_01265	MUY_03134
MUY_01265	MUY_04317
MUY_01265	MUY_00439
MUY_01266	MUY_01266
MUY_01266	MUY_02358
MUY_01266	MUY_02780
MUY_01266	MUY_03082
MUY_01266	MUY_00398
MUY_01266	MUY_00451
MUY_01266	MUY_00807
MUY_01266	MUY_00809
MUY_01266	MUY_00944
MUY_01267	MUY_01267
MUY_01267	MUY_01781
MUY_01267	MUY_03868
MUY_01267	MUY_00730
MUY_01267	MUY_00081
MUY_00113	MUY_00113
MUY_00113	MUY_04083
MUY_01268	MUY_01268
MUY_01268	MUY_01710
MUY_01268	MUY_01782
MUY_01268	MUY_02181
MUY_01268	MUY_02316
MUY_01268	MUY_02675
MUY_01268	MUY_03267
MUY_01269	MUY_01269
MUY_01269	MUY_01779
MUY_01269	MUY_04315
MUY_00114	MUY_00114
MUY_00114	MUY_00115
MUY_00114	MUY_01504
MUY_00114	MUY_00150
MUY_00114	MUY_01696
MUY_00114	MUY_01760
MUY_00114	MUY_01761
MUY_00114	MUY_01879
MUY_00114	MUY_00181
MUY_00114	MUY_02516
MUY_00114	MUY_02560
MUY_00114	MUY_02784

MUY_00114
MUY_00114
MUY_00114
MUY_00114
MUY_00114
MUY_00114
MUY_00114
MUY_00114
MUY_00114
MUY_00114
MUY_00114
MUY_01279
MUY_01279
MUY_01279
MUY_01279
MUY_01279
MUY_01280
MUY_01280
MUY_01280
MUY_01280
MUY_01280
MUY_01280
MUY_01280
MUY_01280
MUY_01280
MUY_01280
MUY_01280
MUY_01280
MUY_01280
MUY_01280
MUY_01280
MUY_01280
MUY_01283
MUY_01283
MUY_01283
MUY_01283
MUY_01283
MUY_01283
MUY_01283
MUY_01283
MUY_01283
MUY_01283
MUY_01283
MUY_01283
MUY_01283
MUY_01283
MUY_01283

MUY_02845
MUY_02852
MUY_03267
MUY_03708
MUY_04026
MUY_04258
MUY_04322
MUY_04504
MUY_00541
MUY_00571
MUY_00758
MUY_01279
MUY_01846
MUY_02250
MUY_03034
MUY_00851
MUY_01280
MUY_01329
MUY_01421
MUY_01611
MUY_01625
MUY_01923
MUY_02100
MUY_02153
MUY_02211
MUY_02331
MUY_00292
MUY_03225
MUY_03591
MUY_03627
MUY_04038
MUY_00933
MUY_01283
MUY_01284
MUY_01297
MUY_01298
MUY_01461
MUY_01466
MUY_00134
MUY_00152
MUY_00153
MUY_01652
MUY_01653
MUY_01656
MUY_01664
MUY_01762
MUY_00189

MUY_01283	MUY_00194
MUY_01283	MUY_02151
MUY_01283	MUY_02208
MUY_01283	MUY_02209
MUY_01283	MUY_02225
MUY_01283	MUY_02597
MUY_01283	MUY_02598
MUY_01283	MUY_02609
MUY_01283	MUY_02643
MUY_01283	MUY_02700
MUY_01283	MUY_02744
MUY_01283	MUY_02745
MUY_01283	MUY_02760
MUY_01283	MUY_00265
MUY_01283	MUY_02894
MUY_01283	MUY_02929
MUY_01283	MUY_02945
MUY_01283	MUY_02946
MUY_01283	MUY_00285
MUY_01283	MUY_03154
MUY_01283	MUY_03330
MUY_01283	MUY_03332
MUY_01283	MUY_03354
MUY_01283	MUY_00322
MUY_01283	MUY_03575
MUY_01283	MUY_03576
MUY_01283	MUY_03582
MUY_01283	MUY_03598
MUY_01283	MUY_03634
MUY_01283	MUY_03649
MUY_01283	MUY_03676
MUY_01283	MUY_03785
MUY_01283	MUY_03805
MUY_01283	MUY_03900
MUY_01283	MUY_03946
MUY_01283	MUY_00372
MUY_01283	MUY_03978
MUY_01283	MUY_04144
MUY_01283	MUY_04172
MUY_01283	MUY_04248
MUY_01283	MUY_04254
MUY_01283	MUY_04266
MUY_01283	MUY_04269
MUY_01283	MUY_04282
MUY_01283	MUY_04296
MUY_01283	MUY_00388
MUY_01283	MUY_04348

MUY_01283	MUY_04357
MUY_01283	MUY_00394
MUY_01283	MUY_04403
MUY_01283	MUY_04416
MUY_01283	MUY_04445
MUY_01283	MUY_04453
MUY_01283	MUY_00420
MUY_01283	MUY_00434
MUY_01283	MUY_00442
MUY_01283	MUY_00516
MUY_01283	MUY_00585
MUY_01283	MUY_00800
MUY_01283	MUY_00815
MUY_01283	MUY_00937
MUY_01283	MUY_00938
MUY_01283	MUY_00939
MUY_01283	MUY_00968
MUY_01283	MUY_00975
MUY_01283	MUY_00996
MUY_01283	MUY_01000
MUY_01284	MUY_01284
MUY_01284	MUY_01297
MUY_01284	MUY_01298
MUY_01284	MUY_01461
MUY_01284	MUY_01466
MUY_01284	MUY_00134
MUY_01284	MUY_00152
MUY_01284	MUY_00153
MUY_01284	MUY_01652
MUY_01284	MUY_01653
MUY_01284	MUY_01656
MUY_01284	MUY_01664
MUY_01284	MUY_01762
MUY_01284	MUY_00189
MUY_01284	MUY_00194
MUY_01284	MUY_02151
MUY_01284	MUY_02208
MUY_01284	MUY_02209
MUY_01284	MUY_02225
MUY_01284	MUY_02597
MUY_01284	MUY_02598
MUY_01284	MUY_02609
MUY_01284	MUY_02643
MUY_01284	MUY_02700
MUY_01284	MUY_02744
MUY_01284	MUY_02745
MUY_01284	MUY_02760

MUY_01284	MUY_00265
MUY_01284	MUY_02894
MUY_01284	MUY_02929
MUY_01284	MUY_02945
MUY_01284	MUY_02946
MUY_01284	MUY_00285
MUY_01284	MUY_03154
MUY_01284	MUY_03330
MUY_01284	MUY_03332
MUY_01284	MUY_03354
MUY_01284	MUY_00322
MUY_01284	MUY_03575
MUY_01284	MUY_03576
MUY_01284	MUY_03582
MUY_01284	MUY_03598
MUY_01284	MUY_03634
MUY_01284	MUY_03649
MUY_01284	MUY_03676
MUY_01284	MUY_03785
MUY_01284	MUY_03805
MUY_01284	MUY_03900
MUY_01284	MUY_03946
MUY_01284	MUY_00372
MUY_01284	MUY_03978
MUY_01284	MUY_04144
MUY_01284	MUY_04172
MUY_01284	MUY_04248
MUY_01284	MUY_04254
MUY_01284	MUY_04266
MUY_01284	MUY_04269
MUY_01284	MUY_04282
MUY_01284	MUY_04296
MUY_01284	MUY_00388
MUY_01284	MUY_04348
MUY_01284	MUY_04357
MUY_01284	MUY_00394
MUY_01284	MUY_04403
MUY_01284	MUY_04416
MUY_01284	MUY_04445
MUY_01284	MUY_04453
MUY_01284	MUY_00420
MUY_01284	MUY_00434
MUY_01284	MUY_00442
MUY_01284	MUY_00516
MUY_01284	MUY_00585
MUY_01284	MUY_00800
MUY_01284	MUY_00815

MUY_01284	MUY_00937
MUY_01284	MUY_00938
MUY_01284	MUY_00939
MUY_01284	MUY_00968
MUY_01284	MUY_00975
MUY_01284	MUY_00996
MUY_01284	MUY_01000
MUY_01291	MUY_01291
MUY_01291	MUY_03260
MUY_01291	MUY_00709
MUY_01297	MUY_01297
MUY_01297	MUY_01298
MUY_01297	MUY_01461
MUY_01297	MUY_01466
MUY_01297	MUY_00134
MUY_01297	MUY_00152
MUY_01297	MUY_00153
MUY_01297	MUY_01652
MUY_01297	MUY_01653
MUY_01297	MUY_01656
MUY_01297	MUY_01664
MUY_01297	MUY_01762
MUY_01297	MUY_00189
MUY_01297	MUY_00194
MUY_01297	MUY_02151
MUY_01297	MUY_02208
MUY_01297	MUY_02209
MUY_01297	MUY_02225
MUY_01297	MUY_02597
MUY_01297	MUY_02598
MUY_01297	MUY_02609
MUY_01297	MUY_02643
MUY_01297	MUY_02700
MUY_01297	MUY_02744
MUY_01297	MUY_02745
MUY_01297	MUY_02760
MUY_01297	MUY_00265
MUY_01297	MUY_02894
MUY_01297	MUY_02929
MUY_01297	MUY_02945
MUY_01297	MUY_02946
MUY_01297	MUY_00285
MUY_01297	MUY_03154
MUY_01297	MUY_03330
MUY_01297	MUY_03332
MUY_01297	MUY_03354
MUY_01297	MUY_00322

MUY_01297	MUY_03575
MUY_01297	MUY_03576
MUY_01297	MUY_03582
MUY_01297	MUY_03598
MUY_01297	MUY_03634
MUY_01297	MUY_03649
MUY_01297	MUY_03676
MUY_01297	MUY_03785
MUY_01297	MUY_03805
MUY_01297	MUY_03900
MUY_01297	MUY_03946
MUY_01297	MUY_00372
MUY_01297	MUY_03978
MUY_01297	MUY_04144
MUY_01297	MUY_04172
MUY_01297	MUY_04248
MUY_01297	MUY_04254
MUY_01297	MUY_04266
MUY_01297	MUY_04269
MUY_01297	MUY_04282
MUY_01297	MUY_04296
MUY_01297	MUY_00388
MUY_01297	MUY_04348
MUY_01297	MUY_04357
MUY_01297	MUY_00394
MUY_01297	MUY_04403
MUY_01297	MUY_04416
MUY_01297	MUY_04445
MUY_01297	MUY_04453
MUY_01297	MUY_00420
MUY_01297	MUY_00434
MUY_01297	MUY_00442
MUY_01297	MUY_00516
MUY_01297	MUY_00585
MUY_01297	MUY_00800
MUY_01297	MUY_00815
MUY_01297	MUY_00937
MUY_01297	MUY_00938
MUY_01297	MUY_00939
MUY_01297	MUY_00968
MUY_01297	MUY_00975
MUY_01297	MUY_00996
MUY_01297	MUY_01000
MUY_01298	MUY_01298
MUY_01298	MUY_01461
MUY_01298	MUY_01466
MUY_01298	MUY_00134

MUY_01298	MUY_00152
MUY_01298	MUY_00153
MUY_01298	MUY_01652
MUY_01298	MUY_01653
MUY_01298	MUY_01656
MUY_01298	MUY_01664
MUY_01298	MUY_01762
MUY_01298	MUY_00189
MUY_01298	MUY_00194
MUY_01298	MUY_02151
MUY_01298	MUY_02208
MUY_01298	MUY_02209
MUY_01298	MUY_02225
MUY_01298	MUY_02597
MUY_01298	MUY_02598
MUY_01298	MUY_02609
MUY_01298	MUY_02643
MUY_01298	MUY_02700
MUY_01298	MUY_02744
MUY_01298	MUY_02745
MUY_01298	MUY_02760
MUY_01298	MUY_00265
MUY_01298	MUY_02894
MUY_01298	MUY_02929
MUY_01298	MUY_02945
MUY_01298	MUY_02946
MUY_01298	MUY_00285
MUY_01298	MUY_03154
MUY_01298	MUY_03330
MUY_01298	MUY_03332
MUY_01298	MUY_03354
MUY_01298	MUY_00322
MUY_01298	MUY_03575
MUY_01298	MUY_03576
MUY_01298	MUY_03582
MUY_01298	MUY_03598
MUY_01298	MUY_03634
MUY_01298	MUY_03649
MUY_01298	MUY_03676
MUY_01298	MUY_03785
MUY_01298	MUY_03805
MUY_01298	MUY_03900
MUY_01298	MUY_03946
MUY_01298	MUY_00372
MUY_01298	MUY_03978
MUY_01298	MUY_04144
MUY_01298	MUY_04172

MUY_01298	MUY_04248
MUY_01298	MUY_04254
MUY_01298	MUY_04266
MUY_01298	MUY_04269
MUY_01298	MUY_04282
MUY_01298	MUY_04296
MUY_01298	MUY_00388
MUY_01298	MUY_04348
MUY_01298	MUY_04357
MUY_01298	MUY_00394
MUY_01298	MUY_04403
MUY_01298	MUY_04416
MUY_01298	MUY_04445
MUY_01298	MUY_04453
MUY_01298	MUY_00420
MUY_01298	MUY_00434
MUY_01298	MUY_00442
MUY_01298	MUY_00516
MUY_01298	MUY_00585
MUY_01298	MUY_00800
MUY_01298	MUY_00815
MUY_01298	MUY_00937
MUY_01298	MUY_00938
MUY_01298	MUY_00939
MUY_01298	MUY_00968
MUY_01298	MUY_00975
MUY_01298	MUY_00996
MUY_01298	MUY_01000
MUY_01299	MUY_01299
MUY_01299	MUY_01348
MUY_01299	MUY_02537
MUY_01299	MUY_02576
MUY_01300	MUY_01300
MUY_01300	MUY_01313
MUY_01300	MUY_00115
MUY_01300	MUY_00150
MUY_01300	MUY_02723
MUY_01300	MUY_03589
MUY_01302	MUY_02320
MUY_01305	MUY_01305
MUY_01305	MUY_00204
MUY_01308	MUY_01308
MUY_01308	MUY_01432
MUY_01308	MUY_01527
MUY_01308	MUY_01582
MUY_01308	MUY_01626
MUY_01308	MUY_01670

MUY_01308	MUY_01874
MUY_01308	MUY_01875
MUY_01308	MUY_00205
MUY_01308	MUY_02178
MUY_01308	MUY_02366
MUY_01308	MUY_00229
MUY_01308	MUY_02521
MUY_01308	MUY_02523
MUY_01308	MUY_00249
MUY_01308	MUY_02655
MUY_01308	MUY_02663
MUY_01308	MUY_00263
MUY_01308	MUY_00264
MUY_01308	MUY_02912
MUY_01308	MUY_02939
MUY_01308	MUY_02940
MUY_01308	MUY_02964
MUY_01308	MUY_00288
MUY_01308	MUY_00289
MUY_01308	MUY_03059
MUY_01308	MUY_03117
MUY_01308	MUY_03127
MUY_01308	MUY_03139
MUY_01308	MUY_03183
MUY_01308	MUY_03201
MUY_01308	MUY_03202
MUY_01308	MUY_03252
MUY_01308	MUY_03306
MUY_01308	MUY_03374
MUY_01308	MUY_03463
MUY_01308	MUY_03477
MUY_01308	MUY_03478
MUY_01308	MUY_03483
MUY_01308	MUY_03609
MUY_01308	MUY_03619
MUY_01308	MUY_03647
MUY_01308	MUY_03648
MUY_01308	MUY_03924
MUY_01308	MUY_04096
MUY_01308	MUY_04146
MUY_01308	MUY_04177
MUY_01308	MUY_04211
MUY_01308	MUY_04212
MUY_01308	MUY_04293
MUY_01308	MUY_04294
MUY_01308	MUY_04358
MUY_01308	MUY_04359

MUY_01308	MUY_04379
MUY_01308	MUY_04417
MUY_01308	MUY_04418
MUY_01308	MUY_04447
MUY_01308	MUY_04448
MUY_01308	MUY_04471
MUY_01308	MUY_00418
MUY_01308	MUY_00436
MUY_01308	MUY_00575
MUY_01308	MUY_00576
MUY_01308	MUY_00790
MUY_01308	MUY_00961
MUY_01309	MUY_01309
MUY_01309	MUY_00206
MUY_01309	MUY_03462
MUY_01311	MUY_03267
MUY_01311	MUY_00435
MUY_01311	MUY_00780
MUY_01313	MUY_01313
MUY_01313	MUY_01737
MUY_01313	MUY_01866
MUY_01313	MUY_03458
MUY_01313	MUY_03611
MUY_01313	MUY_03955
MUY_01313	MUY_00450
MUY_01313	MUY_00603
MUY_01314	MUY_01314
MUY_01315	MUY_01315
MUY_01315	MUY_01405
MUY_01315	MUY_00275
MUY_00115	MUY_00115
MUY_00115	MUY_01504
MUY_00115	MUY_01696
MUY_00115	MUY_01760
MUY_00115	MUY_01761
MUY_00115	MUY_01879
MUY_00115	MUY_00181
MUY_00115	MUY_02516
MUY_00115	MUY_02560
MUY_00115	MUY_02784
MUY_00115	MUY_02785
MUY_00115	MUY_02845
MUY_00115	MUY_02852
MUY_00115	MUY_02889
MUY_00115	MUY_02892
MUY_00115	MUY_02900
MUY_00115	MUY_03267

MUY_00115	MUY_03707
MUY_00115	MUY_03708
MUY_00115	MUY_04026
MUY_00115	MUY_04258
MUY_00115	MUY_04322
MUY_00115	MUY_04504
MUY_00115	MUY_00541
MUY_00115	MUY_00571
MUY_00115	MUY_00628
MUY_01318	MUY_01318
MUY_01318	MUY_03024
MUY_01319	MUY_01319
MUY_01319	MUY_02064
MUY_01319	MUY_03237
MUY_01320	MUY_01320
MUY_01320	MUY_01775
MUY_01320	MUY_02320
MUY_01320	MUY_02528
MUY_01320	MUY_02668
MUY_01320	MUY_03299
MUY_01320	MUY_00065
MUY_01321	MUY_01321
MUY_01321	MUY_01888
MUY_01321	MUY_01959
MUY_01321	MUY_02025
MUY_01321	MUY_02289
MUY_01321	MUY_02548
MUY_01321	MUY_02620
MUY_01321	MUY_03012
MUY_01321	MUY_03341
MUY_01321	MUY_03375
MUY_01321	MUY_03403
MUY_01321	MUY_03451
MUY_01321	MUY_03775
MUY_01321	MUY_00005
MUY_01321	MUY_03863
MUY_01321	MUY_00526
MUY_01321	MUY_00857
MUY_01322	MUY_01322
MUY_01322	MUY_01671
MUY_01322	MUY_01955
MUY_01322	MUY_03393
MUY_01322	MUY_03438
MUY_01323	MUY_03267
MUY_01324	MUY_01324
MUY_01324	MUY_04196
MUY_01325	MUY_01325

MUY_01325	MUY_01326
MUY_01325	MUY_01327
MUY_01325	MUY_01646
MUY_01325	MUY_01649
MUY_01325	MUY_01650
MUY_01325	MUY_00012
MUY_01325	MUY_02376
MUY_01325	MUY_02991
MUY_01325	MUY_03017
MUY_01325	MUY_03292
MUY_01326	MUY_01326
MUY_01326	MUY_01650
MUY_01326	MUY_00012
MUY_01326	MUY_03267
MUY_01327	MUY_01327
MUY_01327	MUY_01646
MUY_01327	MUY_01650
MUY_01327	MUY_03017
MUY_01327	MUY_03267
MUY_01328	MUY_01328
MUY_01328	MUY_01659
MUY_01328	MUY_03034
MUY_01328	MUY_03681
MUY_01328	MUY_03961
MUY_01328	MUY_03976
MUY_01328	MUY_04153
MUY_01328	MUY_04218
MUY_01328	MUY_04395
MUY_01329	MUY_01329
MUY_01329	MUY_01421
MUY_01329	MUY_01611
MUY_01329	MUY_01625
MUY_01329	MUY_01923
MUY_01329	MUY_02100
MUY_01329	MUY_02153
MUY_01329	MUY_02211
MUY_01329	MUY_02331
MUY_01329	MUY_02615
MUY_01329	MUY_02633
MUY_01329	MUY_02950
MUY_01329	MUY_02958
MUY_01329	MUY_00292
MUY_01329	MUY_03128
MUY_01329	MUY_03225
MUY_01329	MUY_03267
MUY_01329	MUY_00313
MUY_01329	MUY_03494

MUY_01329	MUY_03606
MUY_01329	MUY_03627
MUY_01329	MUY_03638
MUY_01329	MUY_03964
MUY_01329	MUY_03998
MUY_01329	MUY_04038
MUY_01329	MUY_00486
MUY_01329	MUY_00681
MUY_01329	MUY_00933
MUY_01331	MUY_01332
MUY_01332	MUY_01332
MUY_01334	MUY_01335
MUY_01341	MUY_01341
MUY_01341	MUY_00758
MUY_01344	MUY_01344
MUY_01344	MUY_03317
MUY_01344	MUY_03934
MUY_01344	MUY_04183
MUY_01344	MUY_04335
MUY_01344	MUY_04430
MUY_01344	MUY_00847
MUY_01344	MUY_00921
MUY_01345	MUY_01345
MUY_01345	MUY_01346
MUY_01345	MUY_00243
MUY_01345	MUY_03342
MUY_01345	MUY_03382
MUY_01345	MUY_03383
MUY_01345	MUY_03945
MUY_01345	MUY_00056
MUY_01346	MUY_01346
MUY_01346	MUY_00243
MUY_01346	MUY_03342
MUY_01346	MUY_03382
MUY_01346	MUY_03383
MUY_01346	MUY_03945
MUY_01346	MUY_00056
MUY_01348	MUY_01348
MUY_01348	MUY_02537
MUY_01348	MUY_02576
MUY_01349	MUY_01349
MUY_01350	MUY_01350
MUY_01350	MUY_04039
MUY_01350	MUY_00384
MUY_01352	MUY_01352
MUY_01352	MUY_01474
MUY_01352	MUY_00193

MUY_01352	MUY_02234
MUY_01353	MUY_01353
MUY_01353	MUY_02149
MUY_01353	MUY_02242
MUY_01353	MUY_02447
MUY_01353	MUY_02696
MUY_01353	MUY_02788
MUY_01353	MUY_03055
MUY_01353	MUY_04126
MUY_01353	MUY_04239
MUY_01353	MUY_00469
MUY_01353	MUY_00485
MUY_01353	MUY_00696
MUY_01354	MUY_01354
MUY_01354	MUY_01355
MUY_01354	MUY_02990
MUY_01354	MUY_03267
MUY_01355	MUY_01355
MUY_01355	MUY_02990
MUY_01357	MUY_01357
MUY_01357	MUY_02207
MUY_01357	MUY_03786
MUY_01357	MUY_04432
MUY_01357	MUY_00423
MUY_01359	MUY_01359
MUY_01359	MUY_00167
MUY_01359	MUY_00174
MUY_01359	MUY_01964
MUY_01359	MUY_00301
MUY_01363	MUY_01363
MUY_01363	MUY_00243
MUY_01363	MUY_03062
MUY_01363	MUY_03342
MUY_01363	MUY_04460
MUY_01365	MUY_01365
MUY_01365	MUY_01368
MUY_01365	MUY_01634
MUY_01365	MUY_00207
MUY_01365	MUY_02203
MUY_01365	MUY_02978
MUY_01365	MUY_00290
MUY_01365	MUY_03320
MUY_01365	MUY_03640
MUY_01365	MUY_03984
MUY_01365	MUY_04198
MUY_01365	MUY_00401
MUY_01365	MUY_00563

MUY_01366	MUY_01366
MUY_01366	MUY_01635
MUY_01366	MUY_01637
MUY_01366	MUY_01783
MUY_01366	MUY_01915
MUY_01366	MUY_02210
MUY_01366	MUY_02616
MUY_01366	MUY_02725
MUY_01366	MUY_02910
MUY_01366	MUY_02981
MUY_01366	MUY_03284
MUY_01366	MUY_03699
MUY_01366	MUY_04467
MUY_01366	MUY_00750
MUY_01366	MUY_00811
MUY_01366	MUY_00824
MUY_01367	MUY_01367
MUY_01367	MUY_03267
MUY_01368	MUY_01368
MUY_01368	MUY_01634
MUY_01368	MUY_00207
MUY_01368	MUY_02203
MUY_01368	MUY_02978
MUY_01368	MUY_00290
MUY_01368	MUY_03320
MUY_01368	MUY_03640
MUY_01368	MUY_03984
MUY_01368	MUY_04198
MUY_01368	MUY_00401
MUY_01368	MUY_00563
MUY_01370	MUY_01370
MUY_01370	MUY_01524
MUY_01370	MUY_02039
MUY_01370	MUY_02052
MUY_01370	MUY_02124
MUY_01370	MUY_02307
MUY_01370	MUY_02411
MUY_01370	MUY_02574
MUY_01370	MUY_02606
MUY_01370	MUY_00250
MUY_01370	MUY_03442
MUY_01370	MUY_04085
MUY_01370	MUY_04171
MUY_01370	MUY_04410
MUY_01370	MUY_00504
MUY_01370	MUY_00695
MUY_01370	MUY_00782

MUY_01370	MUY_00906
MUY_01370	MUY_00920
MUY_01372	MUY_01372
MUY_01372	MUY_01406
MUY_01372	MUY_01623
MUY_01372	MUY_01969
MUY_01372	MUY_02062
MUY_01372	MUY_02258
MUY_01372	MUY_02321
MUY_01372	MUY_02508
MUY_01372	MUY_02515
MUY_01372	MUY_00021
MUY_01372	MUY_03050
MUY_01374	MUY_01374
MUY_01374	MUY_01970
MUY_01374	MUY_01999
MUY_01374	MUY_02036
MUY_01374	MUY_02049
MUY_01374	MUY_02231
MUY_01374	MUY_02238
MUY_01374	MUY_02297
MUY_01374	MUY_02580
MUY_01374	MUY_02982
MUY_01374	MUY_03140
MUY_01374	MUY_03250
MUY_01374	MUY_03431
MUY_01374	MUY_00323
MUY_01374	MUY_03553
MUY_01374	MUY_03612
MUY_01374	MUY_00445
MUY_01374	MUY_00677
MUY_01374	MUY_00749
MUY_01374	MUY_00779
MUY_01374	MUY_00882
MUY_01377	MUY_01378
MUY_01378	MUY_01378
MUY_00119	MUY_00119
MUY_00119	MUY_00120
MUY_00119	MUY_01699
MUY_00119	MUY_01754
MUY_00119	MUY_01788
MUY_00119	MUY_01882
MUY_00119	MUY_02193
MUY_00119	MUY_02320
MUY_00119	MUY_02816
MUY_00119	MUY_03267
MUY_00119	MUY_04476

MUY_01379	MUY_01379
MUY_01379	MUY_02550
MUY_01379	MUY_02614
MUY_01381	MUY_01381
MUY_01381	MUY_01550
MUY_01381	MUY_03771
MUY_01381	MUY_00598
MUY_01382	MUY_01382
MUY_01382	MUY_02707
MUY_01382	MUY_02708
MUY_01382	MUY_02882
MUY_01382	MUY_02883
MUY_01382	MUY_02889
MUY_01382	MUY_02892
MUY_01382	MUY_02900
MUY_01382	MUY_03708
MUY_01382	MUY_03769
MUY_01382	MUY_03778
MUY_01382	MUY_03830
MUY_01382	MUY_04185
MUY_01382	MUY_04271
MUY_01382	MUY_04291
MUY_01382	MUY_04501
MUY_01382	MUY_04505
MUY_01382	MUY_00551
MUY_01382	MUY_00599
MUY_01382	MUY_00602
MUY_01382	MUY_00628
MUY_01382	MUY_00691
MUY_01382	MUY_00795
MUY_01382	MUY_00086
MUY_01382	MUY_00973
MUY_01385	MUY_01385
MUY_01385	MUY_03190
MUY_01385	MUY_04476
MUY_01385	MUY_00610
MUY_01390	MUY_01390
MUY_01390	MUY_01504
MUY_01390	MUY_01696
MUY_01390	MUY_01760
MUY_01390	MUY_01761
MUY_01390	MUY_01879
MUY_01390	MUY_00181
MUY_01390	MUY_02516
MUY_01390	MUY_02560
MUY_01390	MUY_02845
MUY_01390	MUY_02852

MUY_01390	MUY_04026
MUY_01390	MUY_04258
MUY_01390	MUY_04322
MUY_01390	MUY_04504
MUY_01390	MUY_00541
MUY_01390	MUY_00571
MUY_01392	MUY_01392
MUY_01392	MUY_03109
MUY_01392	MUY_00631
MUY_01393	MUY_01393
MUY_01393	MUY_03743
MUY_01394	MUY_02348
MUY_01401	MUY_01401
MUY_01402	MUY_01402
MUY_01402	MUY_00415
MUY_01405	MUY_01405
MUY_01405	MUY_01456
MUY_01405	MUY_01740
MUY_01405	MUY_01741
MUY_01405	MUY_01750
MUY_01405	MUY_01754
MUY_01405	MUY_01755
MUY_01405	MUY_00206
MUY_01405	MUY_02348
MUY_01405	MUY_00231
MUY_01405	MUY_00275
MUY_01405	MUY_04334
MUY_01405	MUY_04466
MUY_01405	MUY_00068
MUY_01406	MUY_01406
MUY_01406	MUY_01623
MUY_01406	MUY_01969
MUY_01406	MUY_02062
MUY_01406	MUY_02258
MUY_01406	MUY_02321
MUY_01406	MUY_02508
MUY_01406	MUY_02515
MUY_01406	MUY_00021
MUY_01406	MUY_03050
MUY_00120	MUY_00120
MUY_00120	MUY_01699
MUY_00120	MUY_01754
MUY_00120	MUY_01788
MUY_00120	MUY_01881
MUY_00120	MUY_01882
MUY_00120	MUY_02193
MUY_00120	MUY_02816

MUY_00120	MUY_03071
MUY_00120	MUY_03267
MUY_00120	MUY_04012
MUY_00120	MUY_04476
MUY_01415	MUY_01415
MUY_01415	MUY_01437
MUY_01415	MUY_01469
MUY_01415	MUY_01660
MUY_01415	MUY_01712
MUY_01415	MUY_01740
MUY_01415	MUY_01741
MUY_01415	MUY_01750
MUY_01415	MUY_01754
MUY_01415	MUY_01825
MUY_01415	MUY_01875
MUY_01415	MUY_02058
MUY_01415	MUY_00206
MUY_01415	MUY_02198
MUY_01415	MUY_00231
MUY_01415	MUY_00255
MUY_01415	MUY_00256
MUY_01415	MUY_02780
MUY_01415	MUY_02797
MUY_01415	MUY_03211
MUY_01415	MUY_03314
MUY_01415	MUY_03496
MUY_01415	MUY_03610
MUY_01415	MUY_03789
MUY_01415	MUY_04086
MUY_01415	MUY_04334
MUY_01415	MUY_04466
MUY_01415	MUY_00435
MUY_01415	MUY_00514
MUY_01415	MUY_00590
MUY_01415	MUY_00068
MUY_01415	MUY_00780
MUY_01415	MUY_00075
MUY_01417	MUY_01417
MUY_01417	MUY_01572
MUY_01417	MUY_02500
MUY_01417	MUY_03715
MUY_01417	MUY_00656
MUY_01418	MUY_01418
MUY_01418	MUY_01520
MUY_01418	MUY_01616
MUY_01418	MUY_01919
MUY_01418	MUY_01937

MUY_01418	MUY_01989
MUY_01418	MUY_00190
MUY_01418	MUY_02094
MUY_01418	MUY_02152
MUY_01418	MUY_02439
MUY_01418	MUY_02467
MUY_01418	MUY_02627
MUY_01418	MUY_00261
MUY_01418	MUY_00308
MUY_01418	MUY_03333
MUY_01418	MUY_03386
MUY_01418	MUY_03449
MUY_01418	MUY_03454
MUY_01418	MUY_03637
MUY_01418	MUY_03810
MUY_01418	MUY_04070
MUY_01418	MUY_04242
MUY_01418	MUY_00382
MUY_01418	MUY_04302
MUY_01418	MUY_04383
MUY_01418	MUY_04433
MUY_01418	MUY_04483
MUY_01418	MUY_00450
MUY_01418	MUY_00464
MUY_01418	MUY_00808
MUY_01418	MUY_00840
MUY_01419	MUY_01419
MUY_01419	MUY_01422
MUY_01419	MUY_01936
MUY_01419	MUY_02132
MUY_01419	MUY_00344
MUY_01419	MUY_03965
MUY_01419	MUY_04290
MUY_01419	MUY_00554
MUY_01420	MUY_01420
MUY_01420	MUY_02154
MUY_01421	MUY_01421
MUY_01421	MUY_01611
MUY_01421	MUY_01625
MUY_01421	MUY_01923
MUY_01421	MUY_02100
MUY_01421	MUY_02153
MUY_01421	MUY_02211
MUY_01421	MUY_02331
MUY_01421	MUY_02615
MUY_01421	MUY_02633
MUY_01421	MUY_02950

MUY_01421	MUY_02958
MUY_01421	MUY_00292
MUY_01421	MUY_03128
MUY_01421	MUY_03225
MUY_01421	MUY_00313
MUY_01421	MUY_03494
MUY_01421	MUY_03606
MUY_01421	MUY_03627
MUY_01421	MUY_03638
MUY_01421	MUY_03964
MUY_01421	MUY_03998
MUY_01421	MUY_04038
MUY_01421	MUY_00486
MUY_01421	MUY_00681
MUY_01421	MUY_00933
MUY_01422	MUY_01422
MUY_01422	MUY_01936
MUY_01422	MUY_02132
MUY_01422	MUY_03965
MUY_01422	MUY_00554
MUY_01425	MUY_01426
MUY_00121	MUY_00121
MUY_00121	MUY_00157
MUY_00121	MUY_01805
MUY_00121	MUY_02673
MUY_00121	MUY_03267
MUY_00121	MUY_00979
MUY_01428	MUY_03425
MUY_01429	MUY_01429
MUY_01429	MUY_03985
MUY_00122	MUY_03089
MUY_01431	MUY_01431
MUY_01431	MUY_01527
MUY_01431	MUY_02320
MUY_01431	MUY_00231
MUY_01431	MUY_02520
MUY_01431	MUY_00264
MUY_01431	MUY_02939
MUY_01431	MUY_00289
MUY_01431	MUY_03478
MUY_01431	MUY_03610
MUY_01431	MUY_03620
MUY_01431	MUY_03647
MUY_01431	MUY_04211
MUY_01431	MUY_04294
MUY_01431	MUY_04358
MUY_01431	MUY_00393

MUY_01431	MUY_04417
MUY_01431	MUY_04420
MUY_01431	MUY_04447
MUY_01431	MUY_04470
MUY_01431	MUY_00438
MUY_01431	MUY_00575
MUY_01432	MUY_01432
MUY_01432	MUY_01527
MUY_01432	MUY_01582
MUY_01432	MUY_01626
MUY_01432	MUY_01670
MUY_01432	MUY_01874
MUY_01432	MUY_01875
MUY_01432	MUY_00205
MUY_01432	MUY_02178
MUY_01432	MUY_02366
MUY_01432	MUY_00229
MUY_01432	MUY_02521
MUY_01432	MUY_02523
MUY_01432	MUY_00249
MUY_01432	MUY_02655
MUY_01432	MUY_02663
MUY_01432	MUY_00263
MUY_01432	MUY_00264
MUY_01432	MUY_02912
MUY_01432	MUY_02939
MUY_01432	MUY_02940
MUY_01432	MUY_02964
MUY_01432	MUY_00288
MUY_01432	MUY_00289
MUY_01432	MUY_03059
MUY_01432	MUY_03117
MUY_01432	MUY_03127
MUY_01432	MUY_03139
MUY_01432	MUY_03183
MUY_01432	MUY_03201
MUY_01432	MUY_03202
MUY_01432	MUY_03252
MUY_01432	MUY_03306
MUY_01432	MUY_03374
MUY_01432	MUY_03463
MUY_01432	MUY_03477
MUY_01432	MUY_03478
MUY_01432	MUY_03483
MUY_01432	MUY_03609
MUY_01432	MUY_03619
MUY_01432	MUY_03647

MUY_01432	MUY_03648
MUY_01432	MUY_03924
MUY_01432	MUY_04096
MUY_01432	MUY_04146
MUY_01432	MUY_04177
MUY_01432	MUY_04211
MUY_01432	MUY_04212
MUY_01432	MUY_04293
MUY_01432	MUY_04294
MUY_01432	MUY_04358
MUY_01432	MUY_04359
MUY_01432	MUY_04379
MUY_01432	MUY_04417
MUY_01432	MUY_04418
MUY_01432	MUY_04447
MUY_01432	MUY_04448
MUY_01432	MUY_04471
MUY_01432	MUY_00418
MUY_01432	MUY_00436
MUY_01432	MUY_00575
MUY_01432	MUY_00576
MUY_01432	MUY_00790
MUY_01432	MUY_00961
MUY_00123	MUY_00123
MUY_01436	MUY_01436
MUY_01437	MUY_01437
MUY_01437	MUY_01605
MUY_01437	MUY_01712
MUY_01437	MUY_02198
MUY_01437	MUY_02240
MUY_01437	MUY_00255
MUY_01437	MUY_02780
MUY_01437	MUY_02797
MUY_01437	MUY_03069
MUY_01437	MUY_03314
MUY_01437	MUY_03856
MUY_01437	MUY_03980
MUY_01437	MUY_04043
MUY_01437	MUY_04086
MUY_01437	MUY_00385
MUY_01437	MUY_04334
MUY_01437	MUY_04343
MUY_01437	MUY_00435
MUY_01437	MUY_00722
MUY_01437	MUY_00780
MUY_01438	MUY_01438
MUY_01438	MUY_01443

MUY_01438	MUY_02371
MUY_01438	MUY_04249
MUY_01438	MUY_00481
MUY_01438	MUY_00573
MUY_01439	MUY_01439
MUY_00124	MUY_02348
MUY_00124	MUY_03089
MUY_01443	MUY_01443
MUY_01443	MUY_02371
MUY_01443	MUY_04249
MUY_01443	MUY_00481
MUY_01443	MUY_00573
MUY_00125	MUY_00125
MUY_00125	MUY_03267
MUY_01445	MUY_01445
MUY_01445	MUY_02582
MUY_01445	MUY_02970
MUY_01445	MUY_03658
MUY_01445	MUY_04399
MUY_01446	MUY_01446
MUY_01446	MUY_02837
MUY_01447	MUY_01447
MUY_01447	MUY_04424
MUY_01447	MUY_00424
MUY_01448	MUY_00935
MUY_00126	MUY_00126
MUY_00126	MUY_01832
MUY_00126	MUY_01909
MUY_00126	MUY_03010
MUY_00126	MUY_00979
MUY_00127	MUY_01645
MUY_00127	MUY_01803
MUY_00127	MUY_01980
MUY_00127	MUY_02538
MUY_00127	MUY_03023
MUY_00127	MUY_03135
MUY_00127	MUY_03213
MUY_00127	MUY_03774
MUY_00127	MUY_03900
MUY_00127	MUY_00496
MUY_00127	MUY_00998
MUY_00128	MUY_01754
MUY_00128	MUY_02320
MUY_00128	MUY_03267
MUY_00128	MUY_04476
MUY_01456	MUY_01456
MUY_01456	MUY_01730

MUY_01456	MUY_01888
MUY_01456	MUY_02338
MUY_01456	MUY_02500
MUY_01456	MUY_02665
MUY_01456	MUY_00312
MUY_01456	MUY_03405
MUY_01456	MUY_03608
MUY_01456	MUY_03612
MUY_01456	MUY_03715
MUY_01456	MUY_03896
MUY_01456	MUY_04466
MUY_01457	MUY_01457
MUY_01457	MUY_02188
MUY_01457	MUY_02617
MUY_01457	MUY_02826
MUY_01458	MUY_01458
MUY_01461	MUY_01461
MUY_01461	MUY_01466
MUY_01461	MUY_00134
MUY_01461	MUY_00152
MUY_01461	MUY_00153
MUY_01461	MUY_01652
MUY_01461	MUY_01653
MUY_01461	MUY_01656
MUY_01461	MUY_01664
MUY_01461	MUY_01762
MUY_01461	MUY_00189
MUY_01461	MUY_00194
MUY_01461	MUY_02151
MUY_01461	MUY_02208
MUY_01461	MUY_02209
MUY_01461	MUY_02225
MUY_01461	MUY_02597
MUY_01461	MUY_02598
MUY_01461	MUY_02609
MUY_01461	MUY_02643
MUY_01461	MUY_02700
MUY_01461	MUY_02744
MUY_01461	MUY_02745
MUY_01461	MUY_02760
MUY_01461	MUY_00265
MUY_01461	MUY_02894
MUY_01461	MUY_02929
MUY_01461	MUY_02945
MUY_01461	MUY_02946
MUY_01461	MUY_03002
MUY_01461	MUY_00285

MUY_01461	MUY_03154
MUY_01461	MUY_03330
MUY_01461	MUY_03332
MUY_01461	MUY_03354
MUY_01461	MUY_00322
MUY_01461	MUY_03575
MUY_01461	MUY_03576
MUY_01461	MUY_03582
MUY_01461	MUY_03598
MUY_01461	MUY_03634
MUY_01461	MUY_03649
MUY_01461	MUY_03676
MUY_01461	MUY_03785
MUY_01461	MUY_03805
MUY_01461	MUY_03900
MUY_01461	MUY_03946
MUY_01461	MUY_00372
MUY_01461	MUY_03978
MUY_01461	MUY_04144
MUY_01461	MUY_04172
MUY_01461	MUY_04248
MUY_01461	MUY_04254
MUY_01461	MUY_04266
MUY_01461	MUY_04269
MUY_01461	MUY_04282
MUY_01461	MUY_04296
MUY_01461	MUY_00388
MUY_01461	MUY_04348
MUY_01461	MUY_04357
MUY_01461	MUY_00394
MUY_01461	MUY_04403
MUY_01461	MUY_04416
MUY_01461	MUY_04445
MUY_01461	MUY_04453
MUY_01461	MUY_00420
MUY_01461	MUY_00434
MUY_01461	MUY_00442
MUY_01461	MUY_00516
MUY_01461	MUY_00585
MUY_01461	MUY_00800
MUY_01461	MUY_00815
MUY_01461	MUY_00937
MUY_01461	MUY_00938
MUY_01461	MUY_00939
MUY_01461	MUY_00968
MUY_01461	MUY_00975
MUY_01461	MUY_00996

MUY_01461	MUY_01000
MUY_00129	MUY_00800
MUY_01463	MUY_01463
MUY_01464	MUY_01464
MUY_01464	MUY_02196
MUY_01464	MUY_03372
MUY_01464	MUY_04216
MUY_01465	MUY_01465
MUY_01465	MUY_00320
MUY_01465	MUY_03970
MUY_01466	MUY_01466
MUY_01466	MUY_00134
MUY_01466	MUY_00152
MUY_01466	MUY_00153
MUY_01466	MUY_01652
MUY_01466	MUY_01653
MUY_01466	MUY_01656
MUY_01466	MUY_01664
MUY_01466	MUY_01762
MUY_01466	MUY_00189
MUY_01466	MUY_00194
MUY_01466	MUY_02151
MUY_01466	MUY_02208
MUY_01466	MUY_02209
MUY_01466	MUY_02225
MUY_01466	MUY_02597
MUY_01466	MUY_02598
MUY_01466	MUY_02609
MUY_01466	MUY_02643
MUY_01466	MUY_02700
MUY_01466	MUY_02744
MUY_01466	MUY_02745
MUY_01466	MUY_02760
MUY_01466	MUY_00265
MUY_01466	MUY_02894
MUY_01466	MUY_02929
MUY_01466	MUY_02945
MUY_01466	MUY_02946
MUY_01466	MUY_00285
MUY_01466	MUY_03154
MUY_01466	MUY_03330
MUY_01466	MUY_03332
MUY_01466	MUY_03354
MUY_01466	MUY_00322
MUY_01466	MUY_03575
MUY_01466	MUY_03576
MUY_01466	MUY_03582

MUY_01466	MUY_03598
MUY_01466	MUY_03634
MUY_01466	MUY_03649
MUY_01466	MUY_00344
MUY_01466	MUY_03676
MUY_01466	MUY_03785
MUY_01466	MUY_03805
MUY_01466	MUY_03900
MUY_01466	MUY_03946
MUY_01466	MUY_00372
MUY_01466	MUY_03978
MUY_01466	MUY_04085
MUY_01466	MUY_04144
MUY_01466	MUY_04172
MUY_01466	MUY_04248
MUY_01466	MUY_04254
MUY_01466	MUY_04266
MUY_01466	MUY_04269
MUY_01466	MUY_04282
MUY_01466	MUY_04296
MUY_01466	MUY_00388
MUY_01466	MUY_04348
MUY_01466	MUY_04357
MUY_01466	MUY_00394
MUY_01466	MUY_04403
MUY_01466	MUY_04416
MUY_01466	MUY_04445
MUY_01466	MUY_04453
MUY_01466	MUY_00420
MUY_01466	MUY_00434
MUY_01466	MUY_00442
MUY_01466	MUY_00516
MUY_01466	MUY_00585
MUY_01466	MUY_00800
MUY_01466	MUY_00815
MUY_01466	MUY_00937
MUY_01466	MUY_00938
MUY_01466	MUY_00939
MUY_01466	MUY_00968
MUY_01466	MUY_00975
MUY_01466	MUY_00996
MUY_01466	MUY_01000
MUY_01467	MUY_01467
MUY_01467	MUY_04314
MUY_00131	MUY_03865
MUY_01468	MUY_01468
MUY_01468	MUY_02081

MUY_01468	MUY_03129
MUY_01468	MUY_03476
MUY_01469	MUY_01469
MUY_01469	MUY_01660
MUY_01469	MUY_02190
MUY_01469	MUY_02320
MUY_01469	MUY_00242
MUY_01469	MUY_02797
MUY_01469	MUY_03031
MUY_01469	MUY_03440
MUY_01469	MUY_03498
MUY_01469	MUY_04086
MUY_01469	MUY_04334
MUY_01469	MUY_00002
MUY_01469	MUY_00780
MUY_00132	MUY_00132
MUY_00132	MUY_00156
MUY_00132	MUY_01876
MUY_00132	MUY_01980
MUY_00132	MUY_02813
MUY_00132	MUY_03023
MUY_00132	MUY_03135
MUY_00132	MUY_03690
MUY_00132	MUY_03774
MUY_00132	MUY_04113
MUY_01471	MUY_01471
MUY_01471	MUY_00279
MUY_01471	MUY_03223
MUY_01471	MUY_00326
MUY_01471	MUY_04247
MUY_01474	MUY_01474
MUY_01474	MUY_00193
MUY_01474	MUY_02234
MUY_01477	MUY_01477
MUY_01477	MUY_01479
MUY_01477	MUY_01804
MUY_01477	MUY_01913
MUY_01477	MUY_02187
MUY_01477	MUY_03056
MUY_01477	MUY_03134
MUY_01477	MUY_04317
MUY_01477	MUY_04385
MUY_01477	MUY_00439
MUY_01477	MUY_00745
MUY_01479	MUY_01479
MUY_01479	MUY_01913
MUY_01479	MUY_02187

MUY_01479	MUY_03134
MUY_01479	MUY_04317
MUY_01479	MUY_00439
MUY_01480	MUY_01480
MUY_01480	MUY_04324
MUY_01481	MUY_01481
MUY_01481	MUY_01975
MUY_01481	MUY_02000
MUY_01481	MUY_00866
MUY_00134	MUY_00134
MUY_00134	MUY_00152
MUY_00134	MUY_00153
MUY_00134	MUY_01652
MUY_00134	MUY_01653
MUY_00134	MUY_01656
MUY_00134	MUY_01664
MUY_00134	MUY_01762
MUY_00134	MUY_00189
MUY_00134	MUY_00194
MUY_00134	MUY_02151
MUY_00134	MUY_02208
MUY_00134	MUY_02209
MUY_00134	MUY_02225
MUY_00134	MUY_02597
MUY_00134	MUY_02598
MUY_00134	MUY_02609
MUY_00134	MUY_02643
MUY_00134	MUY_02700
MUY_00134	MUY_02744
MUY_00134	MUY_02745
MUY_00134	MUY_02760
MUY_00134	MUY_00265
MUY_00134	MUY_02894
MUY_00134	MUY_02929
MUY_00134	MUY_02945
MUY_00134	MUY_02946
MUY_00134	MUY_00285
MUY_00134	MUY_03154
MUY_00134	MUY_03330
MUY_00134	MUY_03332
MUY_00134	MUY_03354
MUY_00134	MUY_03367
MUY_00134	MUY_00322
MUY_00134	MUY_03576
MUY_00134	MUY_03582
MUY_00134	MUY_03598
MUY_00134	MUY_03634

MUY_00134	MUY_03649
MUY_00134	MUY_03676
MUY_00134	MUY_03785
MUY_00134	MUY_03805
MUY_00134	MUY_03900
MUY_00134	MUY_03946
MUY_00134	MUY_00372
MUY_00134	MUY_03978
MUY_00134	MUY_04090
MUY_00134	MUY_04144
MUY_00134	MUY_04172
MUY_00134	MUY_04248
MUY_00134	MUY_04254
MUY_00134	MUY_04266
MUY_00134	MUY_04269
MUY_00134	MUY_04282
MUY_00134	MUY_04296
MUY_00134	MUY_00388
MUY_00134	MUY_04348
MUY_00134	MUY_04357
MUY_00134	MUY_00394
MUY_00134	MUY_04403
MUY_00134	MUY_04416
MUY_00134	MUY_04445
MUY_00134	MUY_04453
MUY_00134	MUY_00420
MUY_00134	MUY_00434
MUY_00134	MUY_00442
MUY_00134	MUY_00516
MUY_00134	MUY_00585
MUY_00134	MUY_00800
MUY_00134	MUY_00815
MUY_00134	MUY_00937
MUY_00134	MUY_00938
MUY_00134	MUY_00939
MUY_00134	MUY_00968
MUY_00134	MUY_00975
MUY_00134	MUY_00996
MUY_00134	MUY_01000
MUY_01482	MUY_01482
MUY_01482	MUY_01497
MUY_01482	MUY_01528
MUY_01482	MUY_01701
MUY_01482	MUY_02050
MUY_01482	MUY_02216
MUY_01482	MUY_02232
MUY_01482	MUY_02364

MUY_01482	MUY_02695
MUY_01482	MUY_00272
MUY_01482	MUY_03126
MUY_01482	MUY_03240
MUY_01482	MUY_03659
MUY_01482	MUY_03701
MUY_01482	MUY_03880
MUY_01482	MUY_04014
MUY_01482	MUY_04194
MUY_01482	MUY_00556
MUY_01482	MUY_00710
MUY_01485	MUY_01485
MUY_01485	MUY_02541
MUY_01485	MUY_02794
MUY_01485	MUY_00023
MUY_01486	MUY_01486
MUY_01486	MUY_02295
MUY_01486	MUY_02578
MUY_01486	MUY_03356
MUY_01486	MUY_03860
MUY_01486	MUY_04473
MUY_01488	MUY_01488
MUY_01488	MUY_03411
MUY_01489	MUY_01489
MUY_01489	MUY_02671
MUY_01489	MUY_03267
MUY_01490	MUY_01490
MUY_01490	MUY_01756
MUY_01490	MUY_02229
MUY_01490	MUY_00275
MUY_01490	MUY_04159
MUY_00136	MUY_00136
MUY_00136	MUY_00139
MUY_00136	MUY_01881
MUY_00136	MUY_03135
MUY_00136	MUY_03253
MUY_00136	MUY_03476
MUY_00136	MUY_03774
MUY_00136	MUY_04205
MUY_00136	MUY_04460
MUY_01492	MUY_01492
MUY_01492	MUY_00010
MUY_01492	MUY_01719
MUY_01492	MUY_01857
MUY_01492	MUY_02376
MUY_01492	MUY_02991
MUY_01492	MUY_03222

MUY_01492	MUY_03263
MUY_01492	MUY_03292
MUY_01492	MUY_03434
MUY_01492	MUY_04020
MUY_01492	MUY_04022
MUY_01494	MUY_01494
MUY_01494	MUY_02015
MUY_01494	MUY_02016
MUY_01494	MUY_02134
MUY_01494	MUY_00241
MUY_01494	MUY_00248
MUY_01494	MUY_02947
MUY_01494	MUY_00277
MUY_01494	MUY_00337
MUY_01494	MUY_04086
MUY_01494	MUY_00433
MUY_01494	MUY_00813
MUY_00137	MUY_03089
MUY_01497	MUY_01497
MUY_01497	MUY_01528
MUY_01497	MUY_01701
MUY_01497	MUY_02050
MUY_01497	MUY_02216
MUY_01497	MUY_02232
MUY_01497	MUY_02364
MUY_01497	MUY_02695
MUY_01497	MUY_00272
MUY_01497	MUY_03006
MUY_01497	MUY_03126
MUY_01497	MUY_03240
MUY_01497	MUY_03659
MUY_01497	MUY_03701
MUY_01497	MUY_03880
MUY_01497	MUY_04014
MUY_01497	MUY_04194
MUY_01497	MUY_00556
MUY_01497	MUY_00710
MUY_01498	MUY_01498
MUY_01498	MUY_02856
MUY_01499	MUY_01499
MUY_01504	MUY_01504
MUY_01504	MUY_01696
MUY_01504	MUY_01760
MUY_01504	MUY_01761
MUY_01504	MUY_01879
MUY_01504	MUY_00181
MUY_01504	MUY_00182

MUY_01504	MUY_02516
MUY_01504	MUY_02560
MUY_01504	MUY_02784
MUY_01504	MUY_02845
MUY_01504	MUY_02852
MUY_01504	MUY_02889
MUY_01504	MUY_02892
MUY_01504	MUY_02900
MUY_01504	MUY_03708
MUY_01504	MUY_03918
MUY_01504	MUY_04026
MUY_01504	MUY_04258
MUY_01504	MUY_04322
MUY_01504	MUY_04504
MUY_01504	MUY_00541
MUY_01504	MUY_00571
MUY_01504	MUY_00628
MUY_00139	MUY_00139
MUY_00139	MUY_00156
MUY_00139	MUY_00157
MUY_00139	MUY_01727
MUY_00139	MUY_00178
MUY_00139	MUY_01896
MUY_00139	MUY_01980
MUY_00139	MUY_02061
MUY_00139	MUY_02391
MUY_00139	MUY_02805
MUY_00139	MUY_02813
MUY_00139	MUY_02820
MUY_00139	MUY_03017
MUY_00139	MUY_03062
MUY_00139	MUY_03135
MUY_00139	MUY_03253
MUY_00139	MUY_03774
MUY_00139	MUY_03865
MUY_00139	MUY_03900
MUY_00139	MUY_03956
MUY_00139	MUY_00496
MUY_00139	MUY_00056
MUY_00139	MUY_00794
MUY_00139	MUY_00979
MUY_00139	MUY_00998
MUY_01506	MUY_01506
MUY_01506	MUY_03242
MUY_01506	MUY_00050
MUY_01510	MUY_01510
MUY_01510	MUY_01741

MUY_01510	MUY_01742
MUY_01510	MUY_00251
MUY_01510	MUY_02996
MUY_01510	MUY_03071
MUY_00140	MUY_00140
MUY_01514	MUY_01514
MUY_01514	MUY_02115
MUY_01514	MUY_02118
MUY_01514	MUY_00229
MUY_01514	MUY_02913
MUY_01514	MUY_03127
MUY_01514	MUY_03612
MUY_01514	MUY_04318
MUY_01514	MUY_00397
MUY_01515	MUY_01515
MUY_01515	MUY_02969
MUY_01517	MUY_01517
MUY_01517	MUY_03267
MUY_01518	MUY_01518
MUY_01518	MUY_00199
MUY_01518	MUY_03267
MUY_01518	MUY_03287
MUY_01519	MUY_01519
MUY_01519	MUY_00344
MUY_01520	MUY_01520
MUY_01520	MUY_01616
MUY_01520	MUY_01919
MUY_01520	MUY_01937
MUY_01520	MUY_01989
MUY_01520	MUY_00190
MUY_01520	MUY_02094
MUY_01520	MUY_02152
MUY_01520	MUY_02439
MUY_01520	MUY_02467
MUY_01520	MUY_02627
MUY_01520	MUY_00261
MUY_01520	MUY_00308
MUY_01520	MUY_03333
MUY_01520	MUY_03386
MUY_01520	MUY_03449
MUY_01520	MUY_03454
MUY_01520	MUY_03637
MUY_01520	MUY_03810
MUY_01520	MUY_04070
MUY_01520	MUY_04242
MUY_01520	MUY_00382
MUY_01520	MUY_04302

MUY_01520	MUY_04383
MUY_01520	MUY_04433
MUY_01520	MUY_04483
MUY_01520	MUY_00450
MUY_01520	MUY_00464
MUY_01520	MUY_00808
MUY_01520	MUY_00840
MUY_01521	MUY_01521
MUY_01521	MUY_03267
MUY_01522	MUY_01522
MUY_01522	MUY_04406
MUY_01523	MUY_01523
MUY_01523	MUY_03267
MUY_01523	MUY_03721
MUY_01523	MUY_00512
MUY_01524	MUY_01524
MUY_01524	MUY_02039
MUY_01524	MUY_02052
MUY_01524	MUY_02124
MUY_01524	MUY_02307
MUY_01524	MUY_02411
MUY_01524	MUY_02574
MUY_01524	MUY_02606
MUY_01524	MUY_00250
MUY_01524	MUY_03442
MUY_01524	MUY_04085
MUY_01524	MUY_04171
MUY_01524	MUY_04410
MUY_01524	MUY_00504
MUY_01524	MUY_00695
MUY_01524	MUY_00782
MUY_01524	MUY_00906
MUY_01524	MUY_00920
MUY_01526	MUY_01526
MUY_01527	MUY_01527
MUY_01527	MUY_01670
MUY_01527	MUY_01874
MUY_01527	MUY_00205
MUY_01527	MUY_00231
MUY_01527	MUY_02520
MUY_01527	MUY_02521
MUY_01527	MUY_02523
MUY_01527	MUY_00249
MUY_01527	MUY_02655
MUY_01527	MUY_02663
MUY_01527	MUY_00263
MUY_01527	MUY_00264

MUY_01527	MUY_02912
MUY_01527	MUY_02939
MUY_01527	MUY_02940
MUY_01527	MUY_00288
MUY_01527	MUY_00289
MUY_01527	MUY_03183
MUY_01527	MUY_03201
MUY_01527	MUY_03202
MUY_01527	MUY_03463
MUY_01527	MUY_03477
MUY_01527	MUY_03478
MUY_01527	MUY_03609
MUY_01527	MUY_03610
MUY_01527	MUY_03619
MUY_01527	MUY_03620
MUY_01527	MUY_03647
MUY_01527	MUY_03648
MUY_01527	MUY_03924
MUY_01527	MUY_04096
MUY_01527	MUY_04146
MUY_01527	MUY_04177
MUY_01527	MUY_04211
MUY_01527	MUY_04212
MUY_01527	MUY_04293
MUY_01527	MUY_04294
MUY_01527	MUY_04358
MUY_01527	MUY_04359
MUY_01527	MUY_00393
MUY_01527	MUY_04417
MUY_01527	MUY_04418
MUY_01527	MUY_04420
MUY_01527	MUY_04447
MUY_01527	MUY_04448
MUY_01527	MUY_04470
MUY_01527	MUY_04471
MUY_01527	MUY_00418
MUY_01527	MUY_00436
MUY_01527	MUY_00438
MUY_01527	MUY_00575
MUY_01527	MUY_00576
MUY_01528	MUY_01528
MUY_01528	MUY_01701
MUY_01528	MUY_02050
MUY_01528	MUY_02216
MUY_01528	MUY_02232
MUY_01528	MUY_02364
MUY_01528	MUY_02695

MUY_01528	MUY_00272
MUY_01528	MUY_03126
MUY_01528	MUY_03240
MUY_01528	MUY_03659
MUY_01528	MUY_03701
MUY_01528	MUY_03880
MUY_01528	MUY_04014
MUY_01528	MUY_04194
MUY_01528	MUY_00556
MUY_01528	MUY_00710
MUY_01529	MUY_01529
MUY_01529	MUY_02273
MUY_01529	MUY_03532
MUY_01530	MUY_02273
MUY_01531	MUY_01531
MUY_01531	MUY_01846
MUY_01531	MUY_01847
MUY_01531	MUY_02012
MUY_01531	MUY_03091
MUY_01531	MUY_03093
MUY_01531	MUY_00024
MUY_01531	MUY_00036
MUY_00143	MUY_00143
MUY_00143	MUY_03267
MUY_01533	MUY_01533
MUY_01534	MUY_01534
MUY_01535	MUY_01535
MUY_01535	MUY_01806
MUY_01535	MUY_01938
MUY_01535	MUY_02353
MUY_01535	MUY_02808
MUY_01535	MUY_02815
MUY_01535	MUY_03543
MUY_01535	MUY_04049
MUY_01535	MUY_00810
MUY_01535	MUY_00869
MUY_01536	MUY_01536
MUY_01536	MUY_01578
MUY_01538	MUY_01538
MUY_01538	MUY_01539
MUY_01538	MUY_01562
MUY_01538	MUY_01845
MUY_01538	MUY_02566
MUY_00144	MUY_00144
MUY_00144	MUY_01598
MUY_00144	MUY_02688
MUY_00144	MUY_00833

MUY_01539	MUY_01539
MUY_01539	MUY_01562
MUY_01539	MUY_01845
MUY_01539	MUY_02566
MUY_01550	MUY_01550
MUY_01550	MUY_03771
MUY_01550	MUY_00598
MUY_01554	MUY_01554
MUY_01554	MUY_01585
MUY_00146	MUY_00146
MUY_00147	MUY_01846
MUY_01562	MUY_01562
MUY_01562	MUY_01845
MUY_01562	MUY_02566
MUY_00149	MUY_00149
MUY_00149	MUY_03267
MUY_00149	MUY_04494
MUY_00150	MUY_00150
MUY_00150	MUY_01848
MUY_00150	MUY_03267
MUY_00151	MUY_00151
MUY_00151	MUY_03267
MUY_01572	MUY_01572
MUY_01572	MUY_02500
MUY_01572	MUY_03715
MUY_01572	MUY_00656
MUY_01575	MUY_01575
MUY_01575	MUY_01669
MUY_01575	MUY_03777
MUY_01575	MUY_00042
MUY_01575	MUY_00534
MUY_01577	MUY_01577
MUY_00152	MUY_00152
MUY_00152	MUY_00153
MUY_00152	MUY_01652
MUY_00152	MUY_01653
MUY_00152	MUY_01656
MUY_00152	MUY_01664
MUY_00152	MUY_01762
MUY_00152	MUY_00189
MUY_00152	MUY_00194
MUY_00152	MUY_02151
MUY_00152	MUY_02208
MUY_00152	MUY_02209
MUY_00152	MUY_02225
MUY_00152	MUY_02597
MUY_00152	MUY_02598

MUY_00152	MUY_02609
MUY_00152	MUY_02643
MUY_00152	MUY_02700
MUY_00152	MUY_02744
MUY_00152	MUY_02745
MUY_00152	MUY_02760
MUY_00152	MUY_00265
MUY_00152	MUY_02894
MUY_00152	MUY_02929
MUY_00152	MUY_02945
MUY_00152	MUY_02946
MUY_00152	MUY_00285
MUY_00152	MUY_03154
MUY_00152	MUY_03330
MUY_00152	MUY_03332
MUY_00152	MUY_03354
MUY_00152	MUY_00322
MUY_00152	MUY_03575
MUY_00152	MUY_03576
MUY_00152	MUY_03582
MUY_00152	MUY_03598
MUY_00152	MUY_03634
MUY_00152	MUY_03649
MUY_00152	MUY_03676
MUY_00152	MUY_03785
MUY_00152	MUY_03805
MUY_00152	MUY_03900
MUY_00152	MUY_03946
MUY_00152	MUY_00372
MUY_00152	MUY_03978
MUY_00152	MUY_04144
MUY_00152	MUY_04172
MUY_00152	MUY_04248
MUY_00152	MUY_04254
MUY_00152	MUY_04266
MUY_00152	MUY_04269
MUY_00152	MUY_04282
MUY_00152	MUY_04296
MUY_00152	MUY_00388
MUY_00152	MUY_04348
MUY_00152	MUY_04357
MUY_00152	MUY_00394
MUY_00152	MUY_04403
MUY_00152	MUY_04416
MUY_00152	MUY_04445
MUY_00152	MUY_04453
MUY_00152	MUY_00420

MUY_00152	MUY_00434
MUY_00152	MUY_00442
MUY_00152	MUY_00516
MUY_00152	MUY_00585
MUY_00152	MUY_00800
MUY_00152	MUY_00815
MUY_00152	MUY_00937
MUY_00152	MUY_00938
MUY_00152	MUY_00939
MUY_00152	MUY_00968
MUY_00152	MUY_00975
MUY_00152	MUY_00996
MUY_00152	MUY_01000
MUY_01578	MUY_01578
MUY_01582	MUY_01582
MUY_01582	MUY_01874
MUY_01582	MUY_00205
MUY_01582	MUY_02521
MUY_01582	MUY_02523
MUY_01582	MUY_00249
MUY_01582	MUY_02663
MUY_01582	MUY_00263
MUY_01582	MUY_02912
MUY_01582	MUY_02940
MUY_01582	MUY_03012
MUY_01582	MUY_00288
MUY_01582	MUY_03117
MUY_01582	MUY_03183
MUY_01582	MUY_03202
MUY_01582	MUY_03306
MUY_01582	MUY_03463
MUY_01582	MUY_03477
MUY_01582	MUY_03609
MUY_01582	MUY_03619
MUY_01582	MUY_03648
MUY_01582	MUY_03863
MUY_01582	MUY_03924
MUY_01582	MUY_04096
MUY_01582	MUY_04146
MUY_01582	MUY_04177
MUY_01582	MUY_04212
MUY_01582	MUY_04293
MUY_01582	MUY_04359
MUY_01582	MUY_04418
MUY_01582	MUY_04448
MUY_01582	MUY_04471
MUY_01582	MUY_00418

MUY_01582	MUY_00436
MUY_01582	MUY_00576
MUY_01582	MUY_00790
MUY_01582	MUY_00961
MUY_01585	MUY_01585
MUY_00153	MUY_00153
MUY_00153	MUY_01652
MUY_00153	MUY_01653
MUY_00153	MUY_01656
MUY_00153	MUY_01664
MUY_00153	MUY_01762
MUY_00153	MUY_00189
MUY_00153	MUY_00194
MUY_00153	MUY_02151
MUY_00153	MUY_02208
MUY_00153	MUY_02209
MUY_00153	MUY_02225
MUY_00153	MUY_02597
MUY_00153	MUY_02598
MUY_00153	MUY_02609
MUY_00153	MUY_02643
MUY_00153	MUY_02700
MUY_00153	MUY_02744
MUY_00153	MUY_02745
MUY_00153	MUY_02760
MUY_00153	MUY_00265
MUY_00153	MUY_02894
MUY_00153	MUY_02929
MUY_00153	MUY_02945
MUY_00153	MUY_02946
MUY_00153	MUY_00285
MUY_00153	MUY_03154
MUY_00153	MUY_03330
MUY_00153	MUY_03332
MUY_00153	MUY_03354
MUY_00153	MUY_00322
MUY_00153	MUY_03575
MUY_00153	MUY_03576
MUY_00153	MUY_03582
MUY_00153	MUY_03598
MUY_00153	MUY_03634
MUY_00153	MUY_03649
MUY_00153	MUY_03676
MUY_00153	MUY_03785
MUY_00153	MUY_03805
MUY_00153	MUY_03900
MUY_00153	MUY_03946

MUY_00153	MUY_00372
MUY_00153	MUY_03978
MUY_00153	MUY_04144
MUY_00153	MUY_04172
MUY_00153	MUY_04248
MUY_00153	MUY_04254
MUY_00153	MUY_04266
MUY_00153	MUY_04269
MUY_00153	MUY_04282
MUY_00153	MUY_04296
MUY_00153	MUY_00388
MUY_00153	MUY_04348
MUY_00153	MUY_04357
MUY_00153	MUY_00394
MUY_00153	MUY_04403
MUY_00153	MUY_04416
MUY_00153	MUY_04445
MUY_00153	MUY_04453
MUY_00153	MUY_00420
MUY_00153	MUY_00434
MUY_00153	MUY_00442
MUY_00153	MUY_00516
MUY_00153	MUY_00585
MUY_00153	MUY_00800
MUY_00153	MUY_00815
MUY_00153	MUY_00937
MUY_00153	MUY_00938
MUY_00153	MUY_00939
MUY_00153	MUY_00968
MUY_00153	MUY_00975
MUY_00153	MUY_00996
MUY_00153	MUY_01000
MUY_01594	MUY_01594
MUY_01594	MUY_00317
MUY_01596	MUY_01596
MUY_01596	MUY_01641
MUY_01596	MUY_01642
MUY_01596	MUY_02043
MUY_01596	MUY_02051
MUY_01596	MUY_02077
MUY_01596	MUY_02333
MUY_01596	MUY_02526
MUY_01596	MUY_02896
MUY_01596	MUY_04163
MUY_01596	MUY_04439
MUY_01596	MUY_04501
MUY_01596	MUY_04505

MUY_01596	MUY_00447
MUY_01596	MUY_00856
MUY_01596	MUY_00917
MUY_01596	MUY_00945
MUY_01597	MUY_01597
MUY_01597	MUY_01796
MUY_01597	MUY_03670
MUY_01597	MUY_03686
MUY_01598	MUY_01598
MUY_01598	MUY_02688
MUY_01598	MUY_03267
MUY_01598	MUY_00833
MUY_01602	MUY_01602
MUY_01602	MUY_02159
MUY_01602	MUY_03267
MUY_01602	MUY_03644
MUY_01602	MUY_03962
MUY_01602	MUY_03975
MUY_01602	MUY_04082
MUY_01602	MUY_04328
MUY_01602	MUY_04428
MUY_01602	MUY_00700
MUY_01603	MUY_01603
MUY_01603	MUY_04157
MUY_01603	MUY_04346
MUY_01603	MUY_00789
MUY_01604	MUY_01604
MUY_01604	MUY_00176
MUY_01604	MUY_00240
MUY_01604	MUY_03659
MUY_01604	MUY_04156
MUY_01604	MUY_04194
MUY_01604	MUY_04345
MUY_01604	MUY_04361
MUY_01604	MUY_04485
MUY_01604	MUY_00787
MUY_01604	MUY_00838
MUY_01604	MUY_00886
MUY_01605	MUY_01605
MUY_01605	MUY_01606
MUY_01605	MUY_01660
MUY_01605	MUY_02135
MUY_01605	MUY_02963
MUY_01605	MUY_03267
MUY_01605	MUY_03683
MUY_01605	MUY_03856
MUY_01605	MUY_03879

MUY_01605	MUY_03980
MUY_01605	MUY_00483
MUY_01606	MUY_01606
MUY_01606	MUY_01771
MUY_01606	MUY_02135
MUY_01606	MUY_03267
MUY_01606	MUY_03856
MUY_01606	MUY_04470
MUY_00155	MUY_00155
MUY_00155	MUY_01000
MUY_01609	MUY_01609
MUY_01609	MUY_01693
MUY_01609	MUY_01735
MUY_01609	MUY_02030
MUY_01609	MUY_02361
MUY_01609	MUY_02588
MUY_01609	MUY_00297
MUY_01609	MUY_03518
MUY_01609	MUY_03667
MUY_01609	MUY_04463
MUY_01609	MUY_00557
MUY_01609	MUY_00583
MUY_01609	MUY_00674
MUY_01610	MUY_01610
MUY_01610	MUY_03425
MUY_01611	MUY_01611
MUY_01611	MUY_01625
MUY_01611	MUY_01923
MUY_01611	MUY_02100
MUY_01611	MUY_02153
MUY_01611	MUY_02211
MUY_01611	MUY_02331
MUY_01611	MUY_02615
MUY_01611	MUY_02633
MUY_01611	MUY_02950
MUY_01611	MUY_02958
MUY_01611	MUY_00292
MUY_01611	MUY_03128
MUY_01611	MUY_03225
MUY_01611	MUY_00313
MUY_01611	MUY_03494
MUY_01611	MUY_03606
MUY_01611	MUY_03627
MUY_01611	MUY_03638
MUY_01611	MUY_03964
MUY_01611	MUY_03998
MUY_01611	MUY_04038

MUY_01611	MUY_00486
MUY_01611	MUY_00681
MUY_01611	MUY_00933
MUY_01612	MUY_01612
MUY_01612	MUY_02141
MUY_01612	MUY_02634
MUY_01612	MUY_03267
MUY_01612	MUY_04436
MUY_01613	MUY_01613
MUY_01613	MUY_01720
MUY_01614	MUY_03071
MUY_01615	MUY_01615
MUY_01615	MUY_02835
MUY_01615	MUY_04096
MUY_00156	MUY_00156
MUY_00156	MUY_00157
MUY_00156	MUY_01980
MUY_00156	MUY_02380
MUY_00156	MUY_02538
MUY_00156	MUY_03135
MUY_00156	MUY_03476
MUY_00156	MUY_03774
MUY_00156	MUY_04205
MUY_00156	MUY_00496
MUY_00156	MUY_00680
MUY_00156	MUY_00998
MUY_01616	MUY_01616
MUY_01616	MUY_01919
MUY_01616	MUY_01937
MUY_01616	MUY_01989
MUY_01616	MUY_00190
MUY_01616	MUY_02094
MUY_01616	MUY_02152
MUY_01616	MUY_02439
MUY_01616	MUY_02467
MUY_01616	MUY_02627
MUY_01616	MUY_00261
MUY_01616	MUY_00308
MUY_01616	MUY_03333
MUY_01616	MUY_03386
MUY_01616	MUY_03449
MUY_01616	MUY_03454
MUY_01616	MUY_03637
MUY_01616	MUY_03810
MUY_01616	MUY_04070
MUY_01616	MUY_04242
MUY_01616	MUY_00382

MUY_01616	MUY_04302
MUY_01616	MUY_04383
MUY_01616	MUY_04433
MUY_01616	MUY_04483
MUY_01616	MUY_00450
MUY_01616	MUY_00464
MUY_01616	MUY_00808
MUY_01616	MUY_00840
MUY_00157	MUY_00157
MUY_00157	MUY_01767
MUY_00157	MUY_01804
MUY_00157	MUY_02813
MUY_00157	MUY_03062
MUY_00157	MUY_03299
MUY_00157	MUY_03774
MUY_00157	MUY_03900
MUY_00157	MUY_04460
MUY_00157	MUY_04494
MUY_00157	MUY_00056
MUY_00157	MUY_00794
MUY_00157	MUY_00931
MUY_00157	MUY_00998
MUY_01620	MUY_01620
MUY_01620	MUY_01876
MUY_01620	MUY_02939
MUY_01620	MUY_03267
MUY_01620	MUY_03478
MUY_01620	MUY_04211
MUY_01620	MUY_04294
MUY_01620	MUY_04358
MUY_01620	MUY_04447
MUY_01623	MUY_01623
MUY_01623	MUY_01969
MUY_01623	MUY_02062
MUY_01623	MUY_02258
MUY_01623	MUY_02321
MUY_01623	MUY_02508
MUY_01623	MUY_02515
MUY_01623	MUY_00021
MUY_01623	MUY_03050
MUY_01624	MUY_01624
MUY_01624	MUY_02506
MUY_01624	MUY_03121
MUY_01624	MUY_03418
MUY_00158	MUY_00158
MUY_00158	MUY_02300
MUY_00158	MUY_03044

MUY_01625	MUY_01625
MUY_01625	MUY_01923
MUY_01625	MUY_02100
MUY_01625	MUY_02153
MUY_01625	MUY_02211
MUY_01625	MUY_02331
MUY_01625	MUY_02615
MUY_01625	MUY_02633
MUY_01625	MUY_02950
MUY_01625	MUY_02958
MUY_01625	MUY_00292
MUY_01625	MUY_03128
MUY_01625	MUY_03225
MUY_01625	MUY_00313
MUY_01625	MUY_03494
MUY_01625	MUY_03606
MUY_01625	MUY_03627
MUY_01625	MUY_03638
MUY_01625	MUY_03964
MUY_01625	MUY_03998
MUY_01625	MUY_04038
MUY_01625	MUY_00486
MUY_01625	MUY_00681
MUY_01625	MUY_00933
MUY_01626	MUY_01626
MUY_01626	MUY_01874
MUY_01626	MUY_00205
MUY_01626	MUY_02366
MUY_01626	MUY_02521
MUY_01626	MUY_02523
MUY_01626	MUY_00249
MUY_01626	MUY_02663
MUY_01626	MUY_00263
MUY_01626	MUY_02912
MUY_01626	MUY_02940
MUY_01626	MUY_00288
MUY_01626	MUY_03183
MUY_01626	MUY_03202
MUY_01626	MUY_03252
MUY_01626	MUY_03463
MUY_01626	MUY_03477
MUY_01626	MUY_03483
MUY_01626	MUY_03609
MUY_01626	MUY_03619
MUY_01626	MUY_03648
MUY_01626	MUY_03924
MUY_01626	MUY_04096

MUY_01626	MUY_04146
MUY_01626	MUY_04177
MUY_01626	MUY_04212
MUY_01626	MUY_04293
MUY_01626	MUY_04359
MUY_01626	MUY_04379
MUY_01626	MUY_04418
MUY_01626	MUY_04448
MUY_01626	MUY_04471
MUY_01626	MUY_00418
MUY_01626	MUY_00436
MUY_01626	MUY_00576
MUY_01629	MUY_01629
MUY_01629	MUY_03659
MUY_01632	MUY_00042
MUY_01634	MUY_01634
MUY_01634	MUY_00207
MUY_01634	MUY_02203
MUY_01634	MUY_02978
MUY_01634	MUY_00290
MUY_01634	MUY_03320
MUY_01634	MUY_03640
MUY_01634	MUY_03984
MUY_01634	MUY_04198
MUY_01634	MUY_00401
MUY_01634	MUY_00563
MUY_01635	MUY_01635
MUY_01635	MUY_01637
MUY_01635	MUY_01783
MUY_01635	MUY_01915
MUY_01635	MUY_02210
MUY_01635	MUY_02616
MUY_01635	MUY_02725
MUY_01635	MUY_02910
MUY_01635	MUY_02981
MUY_01635	MUY_03284
MUY_01635	MUY_03699
MUY_01635	MUY_04467
MUY_01635	MUY_00750
MUY_01635	MUY_00811
MUY_01635	MUY_00824
MUY_01637	MUY_01637
MUY_01637	MUY_01783
MUY_01637	MUY_01915
MUY_01637	MUY_02210
MUY_01637	MUY_02616
MUY_01637	MUY_02725

MUY_01637	MUY_02910
MUY_01637	MUY_02981
MUY_01637	MUY_03284
MUY_01637	MUY_03699
MUY_01637	MUY_04467
MUY_01637	MUY_00750
MUY_01637	MUY_00811
MUY_01637	MUY_00824
MUY_01638	MUY_01638
MUY_01638	MUY_00243
MUY_01638	MUY_03267
MUY_01638	MUY_03342
MUY_01639	MUY_01639
MUY_01639	MUY_02355
MUY_01639	MUY_02618
MUY_01639	MUY_03173
MUY_01639	MUY_03557
MUY_00160	MUY_00160
MUY_00160	MUY_01993
MUY_00160	MUY_02659
MUY_01641	MUY_01641
MUY_01641	MUY_01642
MUY_01641	MUY_02043
MUY_01641	MUY_02051
MUY_01641	MUY_02077
MUY_01641	MUY_02333
MUY_01641	MUY_02526
MUY_01641	MUY_02896
MUY_01641	MUY_04163
MUY_01641	MUY_04439
MUY_01641	MUY_04501
MUY_01641	MUY_04505
MUY_01641	MUY_00447
MUY_01641	MUY_00856
MUY_01641	MUY_00917
MUY_01641	MUY_00945
MUY_01642	MUY_01642
MUY_01642	MUY_02043
MUY_01642	MUY_02051
MUY_01642	MUY_02077
MUY_01642	MUY_02333
MUY_01642	MUY_02526
MUY_01642	MUY_02896
MUY_01642	MUY_04163
MUY_01642	MUY_04439
MUY_01642	MUY_04501
MUY_01642	MUY_04505

MUY_01642	MUY_00447
MUY_01642	MUY_00856
MUY_01642	MUY_00917
MUY_01642	MUY_00945
MUY_01645	MUY_01645
MUY_01645	MUY_01648
MUY_01645	MUY_02108
MUY_01645	MUY_02168
MUY_01645	MUY_00243
MUY_01645	MUY_03342
MUY_01646	MUY_01646
MUY_01646	MUY_01650
MUY_01646	MUY_03017
MUY_01647	MUY_01647
MUY_01647	MUY_01929
MUY_01647	MUY_03229
MUY_01648	MUY_01648
MUY_01648	MUY_01649
MUY_01648	MUY_02108
MUY_01649	MUY_01649
MUY_01649	MUY_01650
MUY_01649	MUY_02595
MUY_01649	MUY_00344
MUY_01649	MUY_04481
MUY_01650	MUY_01650
MUY_01650	MUY_00012
MUY_01650	MUY_02376
MUY_01650	MUY_02991
MUY_01650	MUY_03017
MUY_01650	MUY_03292
MUY_01650	MUY_00344
MUY_01650	MUY_04304
MUY_00161	MUY_00161
MUY_00161	MUY_03067
MUY_00161	MUY_03267
MUY_00161	MUY_03828
MUY_00161	MUY_04502
MUY_01652	MUY_01652
MUY_01652	MUY_01653
MUY_01652	MUY_01656
MUY_01652	MUY_01664
MUY_01652	MUY_01762
MUY_01652	MUY_00189
MUY_01652	MUY_00194
MUY_01652	MUY_02151
MUY_01652	MUY_02208
MUY_01652	MUY_02209

MUY_01652	MUY_02225
MUY_01652	MUY_02597
MUY_01652	MUY_02598
MUY_01652	MUY_02609
MUY_01652	MUY_02643
MUY_01652	MUY_02700
MUY_01652	MUY_02744
MUY_01652	MUY_02745
MUY_01652	MUY_02760
MUY_01652	MUY_00265
MUY_01652	MUY_02894
MUY_01652	MUY_02929
MUY_01652	MUY_02945
MUY_01652	MUY_02946
MUY_01652	MUY_00285
MUY_01652	MUY_03154
MUY_01652	MUY_03330
MUY_01652	MUY_03332
MUY_01652	MUY_03354
MUY_01652	MUY_00322
MUY_01652	MUY_03575
MUY_01652	MUY_03576
MUY_01652	MUY_03582
MUY_01652	MUY_03598
MUY_01652	MUY_03634
MUY_01652	MUY_03649
MUY_01652	MUY_03676
MUY_01652	MUY_03785
MUY_01652	MUY_03805
MUY_01652	MUY_03900
MUY_01652	MUY_03946
MUY_01652	MUY_00372
MUY_01652	MUY_03978
MUY_01652	MUY_04144
MUY_01652	MUY_04172
MUY_01652	MUY_04248
MUY_01652	MUY_04254
MUY_01652	MUY_04266
MUY_01652	MUY_04269
MUY_01652	MUY_04282
MUY_01652	MUY_04296
MUY_01652	MUY_00388
MUY_01652	MUY_04348
MUY_01652	MUY_04357
MUY_01652	MUY_00394
MUY_01652	MUY_04403
MUY_01652	MUY_04416

MUY_01652	MUY_04445
MUY_01652	MUY_04453
MUY_01652	MUY_00420
MUY_01652	MUY_00434
MUY_01652	MUY_00442
MUY_01652	MUY_00516
MUY_01652	MUY_00585
MUY_01652	MUY_00800
MUY_01652	MUY_00815
MUY_01652	MUY_00937
MUY_01652	MUY_00938
MUY_01652	MUY_00939
MUY_01652	MUY_00968
MUY_01652	MUY_00975
MUY_01652	MUY_00996
MUY_01652	MUY_01000
MUY_01653	MUY_01653
MUY_01653	MUY_01656
MUY_01653	MUY_01664
MUY_01653	MUY_01762
MUY_01653	MUY_00189
MUY_01653	MUY_00194
MUY_01653	MUY_02151
MUY_01653	MUY_02208
MUY_01653	MUY_02209
MUY_01653	MUY_02225
MUY_01653	MUY_02597
MUY_01653	MUY_02598
MUY_01653	MUY_02609
MUY_01653	MUY_02643
MUY_01653	MUY_02700
MUY_01653	MUY_02744
MUY_01653	MUY_02745
MUY_01653	MUY_02760
MUY_01653	MUY_00265
MUY_01653	MUY_02894
MUY_01653	MUY_02929
MUY_01653	MUY_02945
MUY_01653	MUY_02946
MUY_01653	MUY_00285
MUY_01653	MUY_03154
MUY_01653	MUY_03330
MUY_01653	MUY_03332
MUY_01653	MUY_03354
MUY_01653	MUY_00322
MUY_01653	MUY_03575
MUY_01653	MUY_03576

MUY_01653	MUY_03582
MUY_01653	MUY_03598
MUY_01653	MUY_03634
MUY_01653	MUY_03649
MUY_01653	MUY_03676
MUY_01653	MUY_03785
MUY_01653	MUY_03805
MUY_01653	MUY_03900
MUY_01653	MUY_03946
MUY_01653	MUY_00372
MUY_01653	MUY_03978
MUY_01653	MUY_04144
MUY_01653	MUY_04172
MUY_01653	MUY_04248
MUY_01653	MUY_04254
MUY_01653	MUY_04266
MUY_01653	MUY_04269
MUY_01653	MUY_04282
MUY_01653	MUY_04296
MUY_01653	MUY_00388
MUY_01653	MUY_04348
MUY_01653	MUY_04357
MUY_01653	MUY_00394
MUY_01653	MUY_04403
MUY_01653	MUY_04416
MUY_01653	MUY_04445
MUY_01653	MUY_04453
MUY_01653	MUY_00420
MUY_01653	MUY_00434
MUY_01653	MUY_00442
MUY_01653	MUY_00516
MUY_01653	MUY_00585
MUY_01653	MUY_00800
MUY_01653	MUY_00815
MUY_01653	MUY_00937
MUY_01653	MUY_00938
MUY_01653	MUY_00939
MUY_01653	MUY_00968
MUY_01653	MUY_00975
MUY_01653	MUY_00996
MUY_01653	MUY_01000
MUY_00010	MUY_00010
MUY_00010	MUY_01719
MUY_00010	MUY_01857
MUY_00010	MUY_02376
MUY_00010	MUY_02991
MUY_00010	MUY_03222

MUY_00010	MUY_03263
MUY_00010	MUY_03267
MUY_00010	MUY_03292
MUY_00010	MUY_03434
MUY_00010	MUY_04020
MUY_00010	MUY_04022
MUY_01654	MUY_01654
MUY_01655	MUY_01655
MUY_01656	MUY_01656
MUY_01656	MUY_01664
MUY_01656	MUY_01762
MUY_01656	MUY_00189
MUY_01656	MUY_00194
MUY_01656	MUY_02151
MUY_01656	MUY_02208
MUY_01656	MUY_02209
MUY_01656	MUY_02225
MUY_01656	MUY_02597
MUY_01656	MUY_02598
MUY_01656	MUY_02609
MUY_01656	MUY_02643
MUY_01656	MUY_02700
MUY_01656	MUY_02744
MUY_01656	MUY_02745
MUY_01656	MUY_02760
MUY_01656	MUY_00265
MUY_01656	MUY_02894
MUY_01656	MUY_02929
MUY_01656	MUY_02945
MUY_01656	MUY_02946
MUY_01656	MUY_00285
MUY_01656	MUY_03154
MUY_01656	MUY_03330
MUY_01656	MUY_03332
MUY_01656	MUY_03354
MUY_01656	MUY_00322
MUY_01656	MUY_03575
MUY_01656	MUY_03576
MUY_01656	MUY_03582
MUY_01656	MUY_03598
MUY_01656	MUY_03634
MUY_01656	MUY_03649
MUY_01656	MUY_03676
MUY_01656	MUY_03785
MUY_01656	MUY_03805
MUY_01656	MUY_03900
MUY_01656	MUY_03946

MUY_01656	MUY_00372
MUY_01656	MUY_03978
MUY_01656	MUY_04144
MUY_01656	MUY_04172
MUY_01656	MUY_04248
MUY_01656	MUY_04254
MUY_01656	MUY_04266
MUY_01656	MUY_04269
MUY_01656	MUY_04282
MUY_01656	MUY_04296
MUY_01656	MUY_00388
MUY_01656	MUY_04348
MUY_01656	MUY_04357
MUY_01656	MUY_00394
MUY_01656	MUY_04403
MUY_01656	MUY_04416
MUY_01656	MUY_04445
MUY_01656	MUY_04453
MUY_01656	MUY_00420
MUY_01656	MUY_00434
MUY_01656	MUY_00442
MUY_01656	MUY_00516
MUY_01656	MUY_00585
MUY_01656	MUY_00800
MUY_01656	MUY_00815
MUY_01656	MUY_00937
MUY_01656	MUY_00938
MUY_01656	MUY_00939
MUY_01656	MUY_00968
MUY_01656	MUY_00975
MUY_01656	MUY_00996
MUY_01656	MUY_01000
MUY_01657	MUY_01657
MUY_01659	MUY_01659
MUY_01659	MUY_03681
MUY_01659	MUY_03961
MUY_01659	MUY_03976
MUY_01659	MUY_04153
MUY_01659	MUY_04218
MUY_01659	MUY_04395
MUY_01660	MUY_01660
MUY_01660	MUY_01709
MUY_01660	MUY_01712
MUY_01660	MUY_01740
MUY_01660	MUY_01741
MUY_01660	MUY_01750
MUY_01660	MUY_01754

MUY_01660	MUY_01825
MUY_01660	MUY_01866
MUY_01660	MUY_02135
MUY_01660	MUY_02145
MUY_01660	MUY_00206
MUY_01660	MUY_02198
MUY_01660	MUY_02571
MUY_01660	MUY_02631
MUY_01660	MUY_02671
MUY_01660	MUY_00256
MUY_01660	MUY_02780
MUY_01660	MUY_02797
MUY_01660	MUY_03069
MUY_01660	MUY_00304
MUY_01660	MUY_03296
MUY_01660	MUY_03314
MUY_01660	MUY_03682
MUY_01660	MUY_03683
MUY_01660	MUY_03856
MUY_01660	MUY_03967
MUY_01660	MUY_04238
MUY_01660	MUY_04334
MUY_01660	MUY_04466
MUY_01660	MUY_00435
MUY_01660	MUY_00470
MUY_01660	MUY_00482
MUY_01660	MUY_00483
MUY_01660	MUY_00514
MUY_01660	MUY_00590
MUY_01660	MUY_00068
MUY_01660	MUY_00780
MUY_01661	MUY_01661
MUY_01661	MUY_02711
MUY_01661	MUY_00719
MUY_01664	MUY_01664
MUY_01664	MUY_01762
MUY_01664	MUY_00189
MUY_01664	MUY_00194
MUY_01664	MUY_02151
MUY_01664	MUY_02208
MUY_01664	MUY_02209
MUY_01664	MUY_02225
MUY_01664	MUY_02597
MUY_01664	MUY_02598
MUY_01664	MUY_02609
MUY_01664	MUY_02643
MUY_01664	MUY_02700

MUY_01664	MUY_02744
MUY_01664	MUY_02745
MUY_01664	MUY_02760
MUY_01664	MUY_00265
MUY_01664	MUY_02894
MUY_01664	MUY_02929
MUY_01664	MUY_02945
MUY_01664	MUY_02946
MUY_01664	MUY_00285
MUY_01664	MUY_03154
MUY_01664	MUY_03267
MUY_01664	MUY_03330
MUY_01664	MUY_03332
MUY_01664	MUY_03354
MUY_01664	MUY_00322
MUY_01664	MUY_03575
MUY_01664	MUY_03576
MUY_01664	MUY_03582
MUY_01664	MUY_03598
MUY_01664	MUY_03634
MUY_01664	MUY_03649
MUY_01664	MUY_03676
MUY_01664	MUY_03785
MUY_01664	MUY_03805
MUY_01664	MUY_03900
MUY_01664	MUY_03946
MUY_01664	MUY_00372
MUY_01664	MUY_03978
MUY_01664	MUY_04144
MUY_01664	MUY_04172
MUY_01664	MUY_04248
MUY_01664	MUY_04254
MUY_01664	MUY_04266
MUY_01664	MUY_04269
MUY_01664	MUY_04282
MUY_01664	MUY_04296
MUY_01664	MUY_00388
MUY_01664	MUY_04348
MUY_01664	MUY_04357
MUY_01664	MUY_00394
MUY_01664	MUY_04403
MUY_01664	MUY_04416
MUY_01664	MUY_04445
MUY_01664	MUY_04453
MUY_01664	MUY_00420
MUY_01664	MUY_00434
MUY_01664	MUY_00442

MUY_01664	MUY_00516
MUY_01664	MUY_00585
MUY_01664	MUY_00800
MUY_01664	MUY_00815
MUY_01664	MUY_00937
MUY_01664	MUY_00938
MUY_01664	MUY_00939
MUY_01664	MUY_00968
MUY_01664	MUY_00975
MUY_01664	MUY_00996
MUY_01664	MUY_01000
MUY_01665	MUY_01665
MUY_01668	MUY_01668
MUY_01668	MUY_02812
MUY_01668	MUY_03012
MUY_01668	MUY_03863
MUY_01669	MUY_01669
MUY_01669	MUY_03777
MUY_01669	MUY_00042
MUY_01669	MUY_00534
MUY_01670	MUY_01670
MUY_01670	MUY_01874
MUY_01670	MUY_00205
MUY_01670	MUY_02521
MUY_01670	MUY_02523
MUY_01670	MUY_02534
MUY_01670	MUY_00249
MUY_01670	MUY_02655
MUY_01670	MUY_02663
MUY_01670	MUY_00263
MUY_01670	MUY_00264
MUY_01670	MUY_02912
MUY_01670	MUY_02940
MUY_01670	MUY_00288
MUY_01670	MUY_00289
MUY_01670	MUY_03183
MUY_01670	MUY_03201
MUY_01670	MUY_03202
MUY_01670	MUY_03463
MUY_01670	MUY_03477
MUY_01670	MUY_03609
MUY_01670	MUY_03619
MUY_01670	MUY_03647
MUY_01670	MUY_03648
MUY_01670	MUY_03924
MUY_01670	MUY_04096
MUY_01670	MUY_04146

MUY_01670	MUY_04177
MUY_01670	MUY_04212
MUY_01670	MUY_04293
MUY_01670	MUY_04359
MUY_01670	MUY_04417
MUY_01670	MUY_04418
MUY_01670	MUY_04448
MUY_01670	MUY_04471
MUY_01670	MUY_00418
MUY_01670	MUY_00436
MUY_01670	MUY_00575
MUY_01670	MUY_00576
MUY_01671	MUY_01671
MUY_01671	MUY_01955
MUY_01671	MUY_03393
MUY_01671	MUY_03438
MUY_01672	MUY_01672
MUY_01672	MUY_01780
MUY_01672	MUY_02286
MUY_01672	MUY_00344
MUY_01672	MUY_04131
MUY_01672	MUY_04432
MUY_01672	MUY_00754
MUY_01673	MUY_01673
MUY_01673	MUY_01915
MUY_01673	MUY_01932
MUY_01673	MUY_01986
MUY_01673	MUY_02136
MUY_01673	MUY_02622
MUY_01673	MUY_03257
MUY_01673	MUY_03267
MUY_00165	MUY_00165
MUY_00165	MUY_01907
MUY_00165	MUY_02518
MUY_00165	MUY_04301
MUY_00165	MUY_00698
MUY_00165	MUY_00871
MUY_01676	MUY_01676
MUY_01676	MUY_03969
MUY_01676	MUY_03995
MUY_01676	MUY_04080
MUY_01676	MUY_04404
MUY_01676	MUY_00474
MUY_01677	MUY_01677
MUY_01677	MUY_01803
MUY_01677	MUY_03220
MUY_01680	MUY_01680

MUY_01680	MUY_01681
MUY_01680	MUY_02649
MUY_01680	MUY_02650
MUY_01680	MUY_03267
MUY_01680	MUY_00473
MUY_01680	MUY_00893
MUY_01680	MUY_00894
MUY_01681	MUY_01681
MUY_01681	MUY_02065
MUY_01681	MUY_02649
MUY_01681	MUY_02650
MUY_01681	MUY_02669
MUY_01681	MUY_03267
MUY_01681	MUY_00472
MUY_01681	MUY_00473
MUY_01681	MUY_00893
MUY_01681	MUY_00894
MUY_01682	MUY_01682
MUY_01682	MUY_01683
MUY_01682	MUY_02127
MUY_01682	MUY_02171
MUY_01682	MUY_02648
MUY_01682	MUY_02785
MUY_01682	MUY_02941
MUY_01682	MUY_03513
MUY_01682	MUY_03515
MUY_01682	MUY_03524
MUY_01682	MUY_00461
MUY_01682	MUY_00895
MUY_01683	MUY_01683
MUY_01683	MUY_02127
MUY_01683	MUY_02171
MUY_01683	MUY_02201
MUY_01683	MUY_02269
MUY_01683	MUY_02460
MUY_01683	MUY_02619
MUY_01683	MUY_02648
MUY_01683	MUY_02697
MUY_01683	MUY_02841
MUY_01683	MUY_02914
MUY_01683	MUY_02917
MUY_01683	MUY_02941
MUY_01683	MUY_03136
MUY_01683	MUY_03137
MUY_01683	MUY_03138
MUY_01683	MUY_03267
MUY_01683	MUY_03279

MUY_01683	MUY_03304
MUY_01683	MUY_00316
MUY_01683	MUY_03513
MUY_01683	MUY_03515
MUY_01683	MUY_03524
MUY_01683	MUY_03585
MUY_01683	MUY_03706
MUY_01683	MUY_03840
MUY_01683	MUY_00459
MUY_01683	MUY_00461
MUY_01683	MUY_00523
MUY_01683	MUY_00895
MUY_01684	MUY_01684
MUY_01684	MUY_01992
MUY_01684	MUY_03149
MUY_01684	MUY_03246
MUY_01684	MUY_03791
MUY_01684	MUY_04098
MUY_01684	MUY_04304
MUY_01685	MUY_01685
MUY_01685	MUY_01991
MUY_01685	MUY_03150
MUY_01685	MUY_03247
MUY_01685	MUY_03790
MUY_01685	MUY_04099
MUY_01685	MUY_04305
MUY_01685	MUY_00630
MUY_01688	MUY_01688
MUY_01688	MUY_00032
MUY_01692	MUY_01692
MUY_01692	MUY_00344
MUY_01693	MUY_01693
MUY_01693	MUY_01735
MUY_01693	MUY_02030
MUY_01693	MUY_02361
MUY_01693	MUY_02588
MUY_01693	MUY_00297
MUY_01693	MUY_03518
MUY_01693	MUY_03667
MUY_01693	MUY_04463
MUY_01693	MUY_00557
MUY_01693	MUY_00583
MUY_01693	MUY_00674
MUY_01696	MUY_01696
MUY_01696	MUY_01760
MUY_01696	MUY_01761
MUY_01696	MUY_01879

MUY_01696	MUY_00181
MUY_01696	MUY_00182
MUY_01696	MUY_02516
MUY_01696	MUY_02560
MUY_01696	MUY_02784
MUY_01696	MUY_02845
MUY_01696	MUY_02852
MUY_01696	MUY_02889
MUY_01696	MUY_02892
MUY_01696	MUY_02900
MUY_01696	MUY_03708
MUY_01696	MUY_03918
MUY_01696	MUY_04026
MUY_01696	MUY_04258
MUY_01696	MUY_04322
MUY_01696	MUY_04504
MUY_01696	MUY_00541
MUY_01696	MUY_00571
MUY_01696	MUY_00628
MUY_01697	MUY_01825
MUY_01697	MUY_02671
MUY_01699	MUY_01699
MUY_01699	MUY_01788
MUY_01699	MUY_01882
MUY_01699	MUY_02193
MUY_01699	MUY_02816
MUY_01699	MUY_03267
MUY_01701	MUY_01701
MUY_01701	MUY_02050
MUY_01701	MUY_02216
MUY_01701	MUY_02232
MUY_01701	MUY_02364
MUY_01701	MUY_02695
MUY_01701	MUY_00272
MUY_01701	MUY_03126
MUY_01701	MUY_03240
MUY_01701	MUY_03659
MUY_01701	MUY_03701
MUY_01701	MUY_03880
MUY_01701	MUY_04014
MUY_01701	MUY_04194
MUY_01701	MUY_00556
MUY_01701	MUY_00710
MUY_01702	MUY_01702
MUY_01702	MUY_03567
MUY_01702	MUY_04127
MUY_01705	MUY_01705

MUY_01707	MUY_01707
MUY_01707	MUY_00247
MUY_01708	MUY_01708
MUY_01709	MUY_02198
MUY_01709	MUY_00256
MUY_01709	MUY_02797
MUY_01709	MUY_04334
MUY_01709	MUY_00435
MUY_01709	MUY_00590
MUY_01710	MUY_01710
MUY_01710	MUY_01782
MUY_01710	MUY_02181
MUY_01710	MUY_02316
MUY_01710	MUY_02675
MUY_01712	MUY_02198
MUY_01712	MUY_04334
MUY_01712	MUY_00435
MUY_01712	MUY_00780
MUY_01713	MUY_01713
MUY_01713	MUY_02783
MUY_01713	MUY_03901
MUY_01713	MUY_04182
MUY_01718	MUY_01718
MUY_01718	MUY_03439
MUY_01719	MUY_01719
MUY_01719	MUY_01857
MUY_01719	MUY_02376
MUY_01719	MUY_02991
MUY_01719	MUY_03222
MUY_01719	MUY_03263
MUY_01719	MUY_03292
MUY_01719	MUY_03434
MUY_01719	MUY_04020
MUY_01719	MUY_04022
MUY_01720	MUY_01720
MUY_01726	MUY_01726
MUY_01726	MUY_03900
MUY_01727	MUY_01727
MUY_01727	MUY_02295
MUY_01727	MUY_02578
MUY_01727	MUY_02830
MUY_01727	MUY_03356
MUY_01727	MUY_03860
MUY_01727	MUY_03951
MUY_01727	MUY_04473
MUY_01728	MUY_01728
MUY_01728	MUY_02530

MUY_01730	MUY_01730
MUY_01730	MUY_01888
MUY_01730	MUY_02338
MUY_01730	MUY_02500
MUY_01730	MUY_02665
MUY_01730	MUY_02999
MUY_01730	MUY_00312
MUY_01730	MUY_03608
MUY_01730	MUY_03715
MUY_01730	MUY_03896
MUY_01730	MUY_04466
MUY_01731	MUY_00033
MUY_01733	MUY_04011
MUY_01734	MUY_01734
MUY_01734	MUY_02534
MUY_01735	MUY_01735
MUY_01735	MUY_02030
MUY_01735	MUY_02361
MUY_01735	MUY_02588
MUY_01735	MUY_00297
MUY_01735	MUY_03215
MUY_01735	MUY_03518
MUY_01735	MUY_03667
MUY_01735	MUY_04463
MUY_01735	MUY_00557
MUY_01735	MUY_00583
MUY_01735	MUY_00674
MUY_01736	MUY_01736
MUY_01736	MUY_00344
MUY_01738	MUY_01738
MUY_01740	MUY_01740
MUY_01740	MUY_01741
MUY_01740	MUY_02434
MUY_01740	MUY_02797
MUY_01740	MUY_02812
MUY_01740	MUY_03067
MUY_01740	MUY_04086
MUY_01740	MUY_04334
MUY_01740	MUY_00068
MUY_01740	MUY_00780
MUY_01741	MUY_01741
MUY_01741	MUY_01742
MUY_01741	MUY_01750
MUY_01741	MUY_00251
MUY_01741	MUY_02797
MUY_01741	MUY_02996
MUY_01741	MUY_03071

MUY_01741	MUY_04086
MUY_01741	MUY_04334
MUY_01741	MUY_00780
MUY_01742	MUY_01742
MUY_01742	MUY_00251
MUY_01742	MUY_02996
MUY_01744	MUY_01744
MUY_01744	MUY_03076
MUY_01744	MUY_03267
MUY_01744	MUY_03274
MUY_01744	MUY_03974
MUY_01744	MUY_00525
MUY_01747	MUY_02632
MUY_01747	MUY_02820
MUY_00167	MUY_00167
MUY_00167	MUY_00174
MUY_00167	MUY_01964
MUY_00167	MUY_00301
MUY_01748	MUY_01748
MUY_01748	MUY_02348
MUY_01749	MUY_01749
MUY_01749	MUY_00303
MUY_01749	MUY_03267
MUY_01750	MUY_01750
MUY_01750	MUY_02797
MUY_01750	MUY_04086
MUY_01750	MUY_04334
MUY_01750	MUY_00780
MUY_01751	MUY_01751
MUY_01751	MUY_01752
MUY_01752	MUY_01752
MUY_01754	MUY_01754
MUY_01754	MUY_01755
MUY_01754	MUY_01768
MUY_01754	MUY_02065
MUY_01754	MUY_02671
MUY_01754	MUY_02797
MUY_01754	MUY_03146
MUY_01754	MUY_03248
MUY_01754	MUY_03345
MUY_01754	MUY_04012
MUY_01754	MUY_04334
MUY_01754	MUY_00541
MUY_01754	MUY_00780
MUY_01755	MUY_01755
MUY_01755	MUY_01768
MUY_01755	MUY_02394

MUY_01755	MUY_03146
MUY_01755	MUY_03248
MUY_01755	MUY_03267
MUY_01755	MUY_00784
MUY_01756	MUY_01756
MUY_01756	MUY_02229
MUY_01756	MUY_00275
MUY_01756	MUY_04159
MUY_01760	MUY_01760
MUY_01760	MUY_01761
MUY_01760	MUY_01879
MUY_01760	MUY_00181
MUY_01760	MUY_00182
MUY_01760	MUY_02516
MUY_01760	MUY_02560
MUY_01760	MUY_02784
MUY_01760	MUY_02845
MUY_01760	MUY_02852
MUY_01760	MUY_02889
MUY_01760	MUY_02892
MUY_01760	MUY_02900
MUY_01760	MUY_03708
MUY_01760	MUY_03918
MUY_01760	MUY_04026
MUY_01760	MUY_04258
MUY_01760	MUY_04322
MUY_01760	MUY_04504
MUY_01760	MUY_00541
MUY_01760	MUY_00571
MUY_01760	MUY_00628
MUY_01761	MUY_01761
MUY_01761	MUY_01879
MUY_01761	MUY_00181
MUY_01761	MUY_00182
MUY_01761	MUY_02516
MUY_01761	MUY_02560
MUY_01761	MUY_02784
MUY_01761	MUY_02845
MUY_01761	MUY_02852
MUY_01761	MUY_02889
MUY_01761	MUY_02892
MUY_01761	MUY_02900
MUY_01761	MUY_03708
MUY_01761	MUY_03918
MUY_01761	MUY_04026
MUY_01761	MUY_04258
MUY_01761	MUY_04322

MUY_01761	MUY_04504
MUY_01761	MUY_00541
MUY_01761	MUY_00571
MUY_01761	MUY_00628
MUY_01762	MUY_01762
MUY_01762	MUY_00189
MUY_01762	MUY_00194
MUY_01762	MUY_02151
MUY_01762	MUY_02208
MUY_01762	MUY_02209
MUY_01762	MUY_02225
MUY_01762	MUY_02597
MUY_01762	MUY_02598
MUY_01762	MUY_02609
MUY_01762	MUY_02643
MUY_01762	MUY_02700
MUY_01762	MUY_02744
MUY_01762	MUY_02745
MUY_01762	MUY_02760
MUY_01762	MUY_00265
MUY_01762	MUY_02894
MUY_01762	MUY_02929
MUY_01762	MUY_02945
MUY_01762	MUY_02946
MUY_01762	MUY_00285
MUY_01762	MUY_03154
MUY_01762	MUY_03330
MUY_01762	MUY_03332
MUY_01762	MUY_03354
MUY_01762	MUY_00322
MUY_01762	MUY_03575
MUY_01762	MUY_03576
MUY_01762	MUY_03582
MUY_01762	MUY_03598
MUY_01762	MUY_03634
MUY_01762	MUY_03649
MUY_01762	MUY_03676
MUY_01762	MUY_03785
MUY_01762	MUY_03805
MUY_01762	MUY_03900
MUY_01762	MUY_03946
MUY_01762	MUY_00372
MUY_01762	MUY_03978
MUY_01762	MUY_04144
MUY_01762	MUY_04172
MUY_01762	MUY_04248
MUY_01762	MUY_04254

MUY_01762	MUY_04266
MUY_01762	MUY_04269
MUY_01762	MUY_04282
MUY_01762	MUY_04296
MUY_01762	MUY_00388
MUY_01762	MUY_04348
MUY_01762	MUY_04357
MUY_01762	MUY_00394
MUY_01762	MUY_04403
MUY_01762	MUY_04416
MUY_01762	MUY_04445
MUY_01762	MUY_04453
MUY_01762	MUY_00420
MUY_01762	MUY_00434
MUY_01762	MUY_00442
MUY_01762	MUY_00516
MUY_01762	MUY_00585
MUY_01762	MUY_00800
MUY_01762	MUY_00815
MUY_01762	MUY_00937
MUY_01762	MUY_00938
MUY_01762	MUY_00939
MUY_01762	MUY_00968
MUY_01762	MUY_00975
MUY_01762	MUY_00996
MUY_01762	MUY_01000
MUY_01764	MUY_01764
MUY_01764	MUY_00215
MUY_01764	MUY_02339
MUY_01764	MUY_02984
MUY_01764	MUY_02993
MUY_01764	MUY_00792
MUY_01766	MUY_01766
MUY_01766	MUY_02813
MUY_01767	MUY_01767
MUY_01767	MUY_02998
MUY_01767	MUY_03404
MUY_01767	MUY_00532
MUY_01767	MUY_00768
MUY_01768	MUY_01768
MUY_01768	MUY_03146
MUY_01768	MUY_03248
MUY_01771	MUY_01771
MUY_01771	MUY_01802
MUY_01771	MUY_02671
MUY_01771	MUY_02785
MUY_01771	MUY_03029

MUY_01771	MUY_03146
MUY_01771	MUY_03267
MUY_01771	MUY_04400
MUY_01772	MUY_01772
MUY_01772	MUY_03077
MUY_01772	MUY_03267
MUY_01772	MUY_00043
MUY_01774	MUY_00074
MUY_01775	MUY_01775
MUY_01775	MUY_02528
MUY_01775	MUY_02668
MUY_01775	MUY_03253
MUY_01775	MUY_03299
MUY_01775	MUY_00065
MUY_01776	MUY_01776
MUY_01776	MUY_01885
MUY_01776	MUY_03025
MUY_01776	MUY_04069
MUY_01776	MUY_00773
MUY_01779	MUY_01779
MUY_01779	MUY_04315
MUY_00171	MUY_00171
MUY_00171	MUY_00916
MUY_01780	MUY_01780
MUY_01780	MUY_02286
MUY_01780	MUY_03267
MUY_01780	MUY_00754
MUY_01781	MUY_01781
MUY_01781	MUY_03868
MUY_01781	MUY_00730
MUY_01781	MUY_00081
MUY_01782	MUY_01782
MUY_01782	MUY_02181
MUY_01782	MUY_02316
MUY_01782	MUY_02675
MUY_01782	MUY_00935
MUY_01783	MUY_01783
MUY_01783	MUY_01784
MUY_01784	MUY_01784
MUY_01784	MUY_02201
MUY_01785	MUY_01785
MUY_01785	MUY_03267
MUY_01786	MUY_01786
MUY_01786	MUY_02202
MUY_01786	MUY_02413
MUY_01786	MUY_03025
MUY_01786	MUY_03345

MUY_01786	MUY_04174
MUY_01786	MUY_00053
MUY_01786	MUY_00057
MUY_01786	MUY_00743
MUY_01786	MUY_00773
MUY_01786	MUY_00074
MUY_00172	MUY_00172
MUY_01788	MUY_01788
MUY_01788	MUY_02193
MUY_01788	MUY_02816
MUY_01788	MUY_02914
MUY_01788	MUY_03136
MUY_01788	MUY_03279
MUY_01788	MUY_03585
MUY_01788	MUY_00523
MUY_01790	MUY_01790
MUY_01790	MUY_01791
MUY_01790	MUY_03267
MUY_01791	MUY_01791
MUY_01791	MUY_03267
MUY_01792	MUY_01792
MUY_01792	MUY_01794
MUY_01792	MUY_00041
MUY_01792	MUY_00463
MUY_01792	MUY_00064
MUY_01793	MUY_01793
MUY_01794	MUY_01794
MUY_01794	MUY_00041
MUY_01794	MUY_00463
MUY_01794	MUY_00064
MUY_01796	MUY_01796
MUY_01796	MUY_01809
MUY_01797	MUY_02671
MUY_01799	MUY_01799
MUY_00173	MUY_00173
MUY_00173	MUY_03675
MUY_01800	MUY_01800
MUY_01801	MUY_01801
MUY_01803	MUY_01803
MUY_01804	MUY_01804
MUY_01804	MUY_03134
MUY_01804	MUY_00745
MUY_01805	MUY_01805
MUY_01805	MUY_02673
MUY_01806	MUY_01806
MUY_01806	MUY_01938
MUY_01806	MUY_02353

MUY_01806	MUY_02808
MUY_01806	MUY_02815
MUY_01806	MUY_03543
MUY_01806	MUY_04049
MUY_01806	MUY_00810
MUY_01806	MUY_00869
MUY_01807	MUY_01807
MUY_01807	MUY_01808
MUY_01807	MUY_02269
MUY_01807	MUY_03041
MUY_01807	MUY_00366
MUY_01807	MUY_00542
MUY_01807	MUY_00070
MUY_01807	MUY_00072
MUY_01808	MUY_01808
MUY_01808	MUY_01897
MUY_01808	MUY_02115
MUY_01808	MUY_02118
MUY_01808	MUY_02697
MUY_01808	MUY_02917
MUY_01808	MUY_03041
MUY_01808	MUY_03094
MUY_01808	MUY_00310
MUY_01808	MUY_03304
MUY_01808	MUY_00316
MUY_01808	MUY_03706
MUY_01808	MUY_00366
MUY_01808	MUY_04318
MUY_01808	MUY_00582
MUY_01808	MUY_00584
MUY_01808	MUY_00589
MUY_01808	MUY_00648
MUY_01808	MUY_00072
MUY_01809	MUY_03670
MUY_01810	MUY_01810
MUY_01811	MUY_01811
MUY_01813	MUY_03862
MUY_01816	MUY_01816
MUY_00174	MUY_00174
MUY_00174	MUY_01964
MUY_00174	MUY_00301
MUY_01819	MUY_01819
MUY_01819	MUY_02198
MUY_01819	MUY_03228
MUY_01819	MUY_00376
MUY_01819	MUY_00377
MUY_01819	MUY_00935

MUY_01820	MUY_00548
MUY_00011	MUY_00011
MUY_00011	MUY_02531
MUY_00011	MUY_02563
MUY_00011	MUY_00253
MUY_00011	MUY_03605
MUY_01821	MUY_01821
MUY_01821	MUY_03267
MUY_01822	MUY_03267
MUY_01824	MUY_01824
MUY_01825	MUY_01825
MUY_01825	MUY_02198
MUY_01825	MUY_02533
MUY_01825	MUY_02534
MUY_01825	MUY_00255
MUY_01825	MUY_00256
MUY_01825	MUY_02797
MUY_01825	MUY_03146
MUY_01825	MUY_03248
MUY_01825	MUY_03496
MUY_01825	MUY_04334
MUY_01825	MUY_04501
MUY_01825	MUY_00435
MUY_01825	MUY_00589
MUY_01825	MUY_00590
MUY_01825	MUY_00780
MUY_01826	MUY_01826
MUY_01826	MUY_01828
MUY_01826	MUY_03277
MUY_01827	MUY_02671
MUY_01828	MUY_01828
MUY_01828	MUY_03267
MUY_01829	MUY_00581
MUY_01830	MUY_01830
MUY_01832	MUY_01832
MUY_01832	MUY_01909
MUY_01832	MUY_03010
MUY_01833	MUY_01833
MUY_01833	MUY_02348
MUY_01836	MUY_01836
MUY_01836	MUY_01947
MUY_01836	MUY_02489
MUY_01836	MUY_02793
MUY_01836	MUY_03058
MUY_01836	MUY_03089
MUY_01836	MUY_04497
MUY_01836	MUY_04508

MUY_01836	MUY_00804
MUY_01836	MUY_00963
MUY_00176	MUY_00176
MUY_00176	MUY_00240
MUY_00176	MUY_03659
MUY_00176	MUY_04156
MUY_00176	MUY_04194
MUY_00176	MUY_04345
MUY_00176	MUY_04361
MUY_00176	MUY_04485
MUY_00176	MUY_00787
MUY_00176	MUY_00838
MUY_00176	MUY_00886
MUY_01837	MUY_01837
MUY_01837	MUY_02391
MUY_01839	MUY_01839
MUY_01840	MUY_02169
MUY_01840	MUY_02316
MUY_01840	MUY_04069
MUY_01840	MUY_04482
MUY_01840	MUY_04494
MUY_01841	MUY_01841
MUY_01841	MUY_03267
MUY_01842	MUY_01842
MUY_01842	MUY_01930
MUY_01843	MUY_01843
MUY_01843	MUY_02259
MUY_01843	MUY_02424
MUY_01843	MUY_02510
MUY_01843	MUY_02765
MUY_01843	MUY_02869
MUY_01843	MUY_03586
MUY_01843	MUY_03904
MUY_01843	MUY_03922
MUY_01843	MUY_04367
MUY_01843	MUY_00496
MUY_01843	MUY_00046
MUY_01843	MUY_00527
MUY_01843	MUY_00008
MUY_01843	MUY_00816
MUY_01844	MUY_01844
MUY_01844	MUY_04507
MUY_01844	MUY_00896
MUY_01845	MUY_01845
MUY_01845	MUY_02566
MUY_01846	MUY_01846
MUY_01846	MUY_01847

MUY_01846	MUY_02012
MUY_01846	MUY_03091
MUY_01846	MUY_03093
MUY_01846	MUY_00303
MUY_01846	MUY_03342
MUY_01846	MUY_03606
MUY_01846	MUY_04196
MUY_01847	MUY_01847
MUY_01847	MUY_02012
MUY_01847	MUY_03091
MUY_01847	MUY_03093
MUY_01847	MUY_03267
MUY_01847	MUY_00024
MUY_01847	MUY_00036
MUY_01848	MUY_01848
MUY_01848	MUY_03267
MUY_00177	MUY_00177
MUY_00177	MUY_00178
MUY_00177	MUY_02935
MUY_00177	MUY_03680
MUY_00177	MUY_00900
MUY_01850	MUY_01860
MUY_01852	MUY_01852
MUY_01853	MUY_01853
MUY_01855	MUY_01855
MUY_01856	MUY_01871
MUY_01857	MUY_03263
MUY_01857	MUY_03434
MUY_01857	MUY_04020
MUY_01857	MUY_04022
MUY_00178	MUY_00178
MUY_00178	MUY_01936
MUY_00178	MUY_02935
MUY_00178	MUY_03680
MUY_00178	MUY_00743
MUY_00178	MUY_00900
MUY_01860	MUY_01860
MUY_01860	MUY_00342
MUY_01860	MUY_04011
MUY_01863	MUY_01863
MUY_01864	MUY_01864
MUY_01864	MUY_01877
MUY_01866	MUY_02198
MUY_01866	MUY_00255
MUY_01866	MUY_00256
MUY_01866	MUY_02797
MUY_01866	MUY_04334

MUY_01866	MUY_00589
MUY_01866	MUY_00590
MUY_01866	MUY_00780
MUY_01867	MUY_01867
MUY_01870	MUY_03034
MUY_01871	MUY_03907
MUY_01871	MUY_03908
MUY_01871	MUY_03909
MUY_01871	MUY_03911
MUY_01872	MUY_01872
MUY_01872	MUY_01873
MUY_01873	MUY_01873
MUY_01874	MUY_01874
MUY_01874	MUY_01875
MUY_01874	MUY_00205
MUY_01874	MUY_02178
MUY_01874	MUY_02366
MUY_01874	MUY_00229
MUY_01874	MUY_02521
MUY_01874	MUY_02523
MUY_01874	MUY_00249
MUY_01874	MUY_02655
MUY_01874	MUY_02663
MUY_01874	MUY_00263
MUY_01874	MUY_00264
MUY_01874	MUY_02912
MUY_01874	MUY_02939
MUY_01874	MUY_02940
MUY_01874	MUY_02964
MUY_01874	MUY_00288
MUY_01874	MUY_00289
MUY_01874	MUY_03059
MUY_01874	MUY_03117
MUY_01874	MUY_03127
MUY_01874	MUY_03139
MUY_01874	MUY_03183
MUY_01874	MUY_03201
MUY_01874	MUY_03202
MUY_01874	MUY_03252
MUY_01874	MUY_03306
MUY_01874	MUY_03374
MUY_01874	MUY_03463
MUY_01874	MUY_03477
MUY_01874	MUY_03478
MUY_01874	MUY_03483
MUY_01874	MUY_03609
MUY_01874	MUY_03619

MUY_01874	MUY_03647
MUY_01874	MUY_03648
MUY_01874	MUY_00344
MUY_01874	MUY_03924
MUY_01874	MUY_04096
MUY_01874	MUY_04146
MUY_01874	MUY_04177
MUY_01874	MUY_04211
MUY_01874	MUY_04212
MUY_01874	MUY_04293
MUY_01874	MUY_04294
MUY_01874	MUY_04358
MUY_01874	MUY_04359
MUY_01874	MUY_04379
MUY_01874	MUY_04417
MUY_01874	MUY_04418
MUY_01874	MUY_04447
MUY_01874	MUY_04448
MUY_01874	MUY_04471
MUY_01874	MUY_00418
MUY_01874	MUY_00436
MUY_01874	MUY_00575
MUY_01874	MUY_00576
MUY_01874	MUY_00790
MUY_01874	MUY_00961
MUY_01875	MUY_01875
MUY_01875	MUY_01877
MUY_01875	MUY_00205
MUY_01875	MUY_02521
MUY_01875	MUY_02523
MUY_01875	MUY_00249
MUY_01875	MUY_02663
MUY_01875	MUY_00263
MUY_01875	MUY_02912
MUY_01875	MUY_02940
MUY_01875	MUY_00288
MUY_01875	MUY_03183
MUY_01875	MUY_03202
MUY_01875	MUY_03425
MUY_01875	MUY_03463
MUY_01875	MUY_03477
MUY_01875	MUY_03609
MUY_01875	MUY_03619
MUY_01875	MUY_03648
MUY_01875	MUY_03924
MUY_01875	MUY_04096
MUY_01875	MUY_04146

MUY_01875	MUY_04177
MUY_01875	MUY_04212
MUY_01875	MUY_04293
MUY_01875	MUY_04359
MUY_01875	MUY_04418
MUY_01875	MUY_04448
MUY_01875	MUY_04471
MUY_01875	MUY_00418
MUY_01875	MUY_00435
MUY_01875	MUY_00436
MUY_01875	MUY_00576
MUY_01876	MUY_01876
MUY_01876	MUY_02939
MUY_01876	MUY_03478
MUY_01876	MUY_00344
MUY_01876	MUY_04211
MUY_01876	MUY_04294
MUY_01876	MUY_04358
MUY_01876	MUY_04447
MUY_01876	MUY_00543
MUY_01877	MUY_01877
MUY_01877	MUY_01878
MUY_01878	MUY_01878
MUY_01879	MUY_01879
MUY_01879	MUY_00181
MUY_01879	MUY_00182
MUY_01879	MUY_02516
MUY_01879	MUY_02560
MUY_01879	MUY_02784
MUY_01879	MUY_02845
MUY_01879	MUY_02852
MUY_01879	MUY_02889
MUY_01879	MUY_02892
MUY_01879	MUY_02900
MUY_01879	MUY_03708
MUY_01879	MUY_03918
MUY_01879	MUY_04026
MUY_01879	MUY_04258
MUY_01879	MUY_04322
MUY_01879	MUY_04504
MUY_01879	MUY_00541
MUY_01879	MUY_00571
MUY_01879	MUY_00628
MUY_01881	MUY_01881
MUY_01882	MUY_01882
MUY_01882	MUY_02816
MUY_01882	MUY_03267

MUY_01884	MUY_03267
MUY_01885	MUY_01885
MUY_01885	MUY_03025
MUY_01885	MUY_03860
MUY_01885	MUY_00588
MUY_01885	MUY_00773
MUY_01887	MUY_01887
MUY_00180	MUY_00180
MUY_00180	MUY_04112
MUY_00180	MUY_00400
MUY_00180	MUY_00559
MUY_01888	MUY_01888
MUY_01888	MUY_02665
MUY_01888	MUY_00310
MUY_01888	MUY_00312
MUY_01888	MUY_03608
MUY_01888	MUY_03896
MUY_01888	MUY_04466
MUY_01888	MUY_00648
MUY_01888	MUY_00857
MUY_01889	MUY_01889
MUY_01889	MUY_00014
MUY_01889	MUY_03020
MUY_01889	MUY_03267
MUY_01889	MUY_03872
MUY_01889	MUY_00378
MUY_01889	MUY_04230
MUY_01890	MUY_00003
MUY_01890	MUY_03200
MUY_01890	MUY_00024
MUY_01891	MUY_01891
MUY_01892	MUY_01892
MUY_00181	MUY_00181
MUY_00181	MUY_00182
MUY_00181	MUY_02516
MUY_00181	MUY_02560
MUY_00181	MUY_02784
MUY_00181	MUY_02845
MUY_00181	MUY_02852
MUY_00181	MUY_02889
MUY_00181	MUY_02892
MUY_00181	MUY_02900
MUY_00181	MUY_03708
MUY_00181	MUY_03918
MUY_00181	MUY_04026
MUY_00181	MUY_04258
MUY_00181	MUY_04322

MUY_00181	MUY_04504
MUY_00181	MUY_00541
MUY_00181	MUY_00571
MUY_00181	MUY_00628
MUY_01897	MUY_01897
MUY_01897	MUY_01906
MUY_01897	MUY_01932
MUY_01897	MUY_03041
MUY_01897	MUY_03123
MUY_01897	MUY_03267
MUY_01897	MUY_00366
MUY_01897	MUY_00072
MUY_00182	MUY_00182
MUY_00182	MUY_02516
MUY_00182	MUY_02560
MUY_00182	MUY_02845
MUY_00182	MUY_02852
MUY_00182	MUY_02889
MUY_00182	MUY_02892
MUY_00182	MUY_02900
MUY_00182	MUY_03708
MUY_00182	MUY_04026
MUY_00182	MUY_04258
MUY_00182	MUY_04322
MUY_00182	MUY_04504
MUY_00182	MUY_00541
MUY_00182	MUY_00571
MUY_00182	MUY_00628
MUY_01899	MUY_01899
MUY_01900	MUY_03267
MUY_01902	MUY_01902
MUY_01902	MUY_03344
MUY_01903	MUY_01903
MUY_01903	MUY_02831
MUY_01904	MUY_00344
MUY_01904	MUY_04438
MUY_01905	MUY_03135
MUY_01905	MUY_03169
MUY_01905	MUY_03476
MUY_01905	MUY_00344
MUY_01905	MUY_03774
MUY_01906	MUY_01906
MUY_01906	MUY_01932
MUY_01906	MUY_02320
MUY_01906	MUY_03123
MUY_01906	MUY_03267
MUY_00183	MUY_00183

MUY_00183	MUY_00927
MUY_01907	MUY_01907
MUY_01907	MUY_02518
MUY_01907	MUY_04301
MUY_01907	MUY_00698
MUY_01907	MUY_00871
MUY_01908	MUY_01908
MUY_01908	MUY_01922
MUY_01909	MUY_01909
MUY_01909	MUY_03010
MUY_01911	MUY_01911
MUY_01911	MUY_00402
MUY_01912	MUY_01912
MUY_01912	MUY_02179
MUY_01912	MUY_03099
MUY_01912	MUY_03267
MUY_01913	MUY_01913
MUY_01913	MUY_02187
MUY_01913	MUY_03134
MUY_01913	MUY_03267
MUY_01913	MUY_04317
MUY_01913	MUY_00439
MUY_01914	MUY_01914
MUY_01914	MUY_00257
MUY_01914	MUY_03633
MUY_01914	MUY_03837
MUY_01915	MUY_01915
MUY_01915	MUY_01932
MUY_01915	MUY_02616
MUY_01915	MUY_02697
MUY_01915	MUY_02725
MUY_01915	MUY_02910
MUY_01915	MUY_02917
MUY_01915	MUY_03267
MUY_01915	MUY_03284
MUY_01915	MUY_03304
MUY_01915	MUY_00316
MUY_01915	MUY_03706
MUY_01915	MUY_04467
MUY_01915	MUY_04511
MUY_01915	MUY_00824
MUY_01916	MUY_01916
MUY_01916	MUY_02803
MUY_01916	MUY_03742
MUY_01916	MUY_03844
MUY_01916	MUY_04025
MUY_01918	MUY_02348

MUY_01919	MUY_01919
MUY_01919	MUY_01937
MUY_01919	MUY_01989
MUY_01919	MUY_00190
MUY_01919	MUY_02094
MUY_01919	MUY_02152
MUY_01919	MUY_02439
MUY_01919	MUY_02467
MUY_01919	MUY_02627
MUY_01919	MUY_00261
MUY_01919	MUY_00308
MUY_01919	MUY_03333
MUY_01919	MUY_03386
MUY_01919	MUY_03449
MUY_01919	MUY_03454
MUY_01919	MUY_03637
MUY_01919	MUY_03810
MUY_01919	MUY_04070
MUY_01919	MUY_04242
MUY_01919	MUY_00382
MUY_01919	MUY_04302
MUY_01919	MUY_04383
MUY_01919	MUY_04433
MUY_01919	MUY_04483
MUY_01919	MUY_00450
MUY_01919	MUY_00464
MUY_01919	MUY_00808
MUY_01919	MUY_00840
MUY_01922	MUY_01922
MUY_01923	MUY_01923
MUY_01923	MUY_02100
MUY_01923	MUY_02153
MUY_01923	MUY_02211
MUY_01923	MUY_02331
MUY_01923	MUY_02615
MUY_01923	MUY_02633
MUY_01923	MUY_02950
MUY_01923	MUY_02958
MUY_01923	MUY_00292
MUY_01923	MUY_03128
MUY_01923	MUY_03225
MUY_01923	MUY_00313
MUY_01923	MUY_03494
MUY_01923	MUY_03606
MUY_01923	MUY_03627
MUY_01923	MUY_03638
MUY_01923	MUY_03964

MUY_01923	MUY_03998
MUY_01923	MUY_04038
MUY_01923	MUY_00486
MUY_01923	MUY_00681
MUY_01923	MUY_00933
MUY_00185	MUY_00185
MUY_00185	MUY_03267
MUY_01929	MUY_01929
MUY_01929	MUY_03229
MUY_01930	MUY_01930
MUY_01930	MUY_03267
MUY_01932	MUY_01932
MUY_01932	MUY_03123
MUY_01932	MUY_03211
MUY_01932	MUY_03795
MUY_01932	MUY_00527
MUY_01933	MUY_01933
MUY_01935	MUY_01935
MUY_01936	MUY_01936
MUY_01936	MUY_01937
MUY_01936	MUY_02132
MUY_01936	MUY_00344
MUY_01936	MUY_00554
MUY_01937	MUY_01937
MUY_01937	MUY_01989
MUY_01937	MUY_00190
MUY_01937	MUY_02094
MUY_01937	MUY_02152
MUY_01937	MUY_02439
MUY_01937	MUY_02467
MUY_01937	MUY_02627
MUY_01937	MUY_00261
MUY_01937	MUY_00308
MUY_01937	MUY_03333
MUY_01937	MUY_03386
MUY_01937	MUY_03449
MUY_01937	MUY_03454
MUY_01937	MUY_03637
MUY_01937	MUY_03810
MUY_01937	MUY_04070
MUY_01937	MUY_04242
MUY_01937	MUY_00382
MUY_01937	MUY_04302
MUY_01937	MUY_04383
MUY_01937	MUY_04433
MUY_01937	MUY_04483
MUY_01937	MUY_00450

MUY_01937	MUY_00464
MUY_01937	MUY_00808
MUY_01937	MUY_00840
MUY_01938	MUY_01938
MUY_01938	MUY_02353
MUY_01938	MUY_02808
MUY_01938	MUY_02815
MUY_01938	MUY_03543
MUY_01938	MUY_04049
MUY_01938	MUY_00810
MUY_01938	MUY_00869
MUY_01940	MUY_01940
MUY_01941	MUY_01941
MUY_01942	MUY_01942
MUY_01942	MUY_00003
MUY_01942	MUY_02939
MUY_01942	MUY_03478
MUY_01942	MUY_04211
MUY_01942	MUY_04294
MUY_01942	MUY_04358
MUY_01942	MUY_04402
MUY_01942	MUY_04447
MUY_01943	MUY_01943
MUY_01943	MUY_02589
MUY_00186	MUY_03267
MUY_01947	MUY_01947
MUY_01947	MUY_02489
MUY_01947	MUY_02793
MUY_01947	MUY_03058
MUY_01947	MUY_03089
MUY_01947	MUY_04497
MUY_01947	MUY_04508
MUY_01947	MUY_00804
MUY_01947	MUY_00963
MUY_01948	MUY_01948
MUY_01948	MUY_02825
MUY_01949	MUY_01949
MUY_01949	MUY_02047
MUY_01949	MUY_04028
MUY_01952	MUY_01952
MUY_01952	MUY_01953
MUY_01952	MUY_03567
MUY_01953	MUY_01953
MUY_01953	MUY_03567
MUY_01955	MUY_01955
MUY_01955	MUY_00218
MUY_01955	MUY_03393

MUY_01955	MUY_03438
MUY_01959	MUY_01959
MUY_01959	MUY_02025
MUY_01959	MUY_02289
MUY_01959	MUY_02620
MUY_01959	MUY_03341
MUY_01959	MUY_03375
MUY_01959	MUY_03451
MUY_01959	MUY_03775
MUY_01959	MUY_00378
MUY_01959	MUY_04230
MUY_01959	MUY_00526
MUY_01959	MUY_00857
MUY_01964	MUY_01964
MUY_01964	MUY_00301
MUY_01969	MUY_01969
MUY_01969	MUY_02062
MUY_01969	MUY_02258
MUY_01969	MUY_02321
MUY_01969	MUY_02508
MUY_01969	MUY_02515
MUY_01969	MUY_00021
MUY_01969	MUY_03050
MUY_01970	MUY_01970
MUY_01970	MUY_01999
MUY_01970	MUY_02036
MUY_01970	MUY_02049
MUY_01970	MUY_02231
MUY_01970	MUY_02238
MUY_01970	MUY_02297
MUY_01970	MUY_02580
MUY_01970	MUY_02982
MUY_01970	MUY_03140
MUY_01970	MUY_03250
MUY_01970	MUY_03431
MUY_01970	MUY_00323
MUY_01970	MUY_03553
MUY_01970	MUY_03612
MUY_01970	MUY_00445
MUY_01970	MUY_00677
MUY_01970	MUY_00749
MUY_01970	MUY_00779
MUY_01970	MUY_00882
MUY_01972	MUY_01972
MUY_01973	MUY_01973
MUY_01973	MUY_02615
MUY_01973	MUY_02633

MUY_01973	MUY_02950
MUY_01973	MUY_02958
MUY_01973	MUY_03128
MUY_01973	MUY_00313
MUY_01973	MUY_03494
MUY_01973	MUY_03606
MUY_01973	MUY_03638
MUY_01973	MUY_03964
MUY_01973	MUY_03998
MUY_01973	MUY_04045
MUY_01973	MUY_00486
MUY_01973	MUY_00681
MUY_01973	MUY_00721
MUY_01975	MUY_01975
MUY_01975	MUY_02000
MUY_01975	MUY_00866
MUY_01976	MUY_01976
MUY_01976	MUY_02820
MUY_01978	MUY_02821
MUY_01978	MUY_00780
MUY_01979	MUY_01979
MUY_01979	MUY_02497
MUY_01979	MUY_00020
MUY_01980	MUY_01980
MUY_01980	MUY_03062
MUY_01980	MUY_03774
MUY_01980	MUY_04091
MUY_01980	MUY_04260
MUY_01980	MUY_04262
MUY_01980	MUY_04494
MUY_01980	MUY_00998
MUY_00012	MUY_00012
MUY_00012	MUY_03267
MUY_01983	MUY_01983
MUY_01983	MUY_04113
MUY_01983	MUY_00794
MUY_00189	MUY_00189
MUY_00189	MUY_00194
MUY_00189	MUY_02151
MUY_00189	MUY_02208
MUY_00189	MUY_02209
MUY_00189	MUY_02225
MUY_00189	MUY_02597
MUY_00189	MUY_02598
MUY_00189	MUY_02609
MUY_00189	MUY_02643
MUY_00189	MUY_02700

MUY_00189	MUY_02744
MUY_00189	MUY_02745
MUY_00189	MUY_02760
MUY_00189	MUY_00265
MUY_00189	MUY_02894
MUY_00189	MUY_02929
MUY_00189	MUY_02945
MUY_00189	MUY_02946
MUY_00189	MUY_00285
MUY_00189	MUY_03154
MUY_00189	MUY_03330
MUY_00189	MUY_03332
MUY_00189	MUY_03354
MUY_00189	MUY_00322
MUY_00189	MUY_03575
MUY_00189	MUY_03576
MUY_00189	MUY_03582
MUY_00189	MUY_03598
MUY_00189	MUY_03634
MUY_00189	MUY_03649
MUY_00189	MUY_03676
MUY_00189	MUY_03785
MUY_00189	MUY_03805
MUY_00189	MUY_03900
MUY_00189	MUY_03946
MUY_00189	MUY_00372
MUY_00189	MUY_03978
MUY_00189	MUY_04144
MUY_00189	MUY_04172
MUY_00189	MUY_04248
MUY_00189	MUY_04254
MUY_00189	MUY_04266
MUY_00189	MUY_04269
MUY_00189	MUY_04282
MUY_00189	MUY_04296
MUY_00189	MUY_00388
MUY_00189	MUY_04348
MUY_00189	MUY_04357
MUY_00189	MUY_00394
MUY_00189	MUY_04403
MUY_00189	MUY_04416
MUY_00189	MUY_04445
MUY_00189	MUY_04453
MUY_00189	MUY_00420
MUY_00189	MUY_00434
MUY_00189	MUY_00442
MUY_00189	MUY_00516

MUY_00189	MUY_00585
MUY_00189	MUY_00800
MUY_00189	MUY_00815
MUY_00189	MUY_00937
MUY_00189	MUY_00938
MUY_00189	MUY_00939
MUY_00189	MUY_00968
MUY_00189	MUY_00975
MUY_00189	MUY_00996
MUY_00189	MUY_01000
MUY_01986	MUY_01986
MUY_01986	MUY_02136
MUY_01986	MUY_02622
MUY_01986	MUY_03257
MUY_01987	MUY_01987
MUY_01987	MUY_03014
MUY_01987	MUY_03054
MUY_01987	MUY_03244
MUY_01987	MUY_03556
MUY_01987	MUY_03573
MUY_01987	MUY_03574
MUY_01987	MUY_03660
MUY_01987	MUY_00766
MUY_01988	MUY_01988
MUY_01988	MUY_02568
MUY_01988	MUY_02758
MUY_01988	MUY_04263
MUY_01988	MUY_00946
MUY_01989	MUY_01989
MUY_01989	MUY_00190
MUY_01989	MUY_02094
MUY_01989	MUY_02152
MUY_01989	MUY_02439
MUY_01989	MUY_02467
MUY_01989	MUY_02627
MUY_01989	MUY_00261
MUY_01989	MUY_00308
MUY_01989	MUY_03333
MUY_01989	MUY_03386
MUY_01989	MUY_03449
MUY_01989	MUY_03454
MUY_01989	MUY_03637
MUY_01989	MUY_03810
MUY_01989	MUY_04070
MUY_01989	MUY_04242
MUY_01989	MUY_00382
MUY_01989	MUY_04302

MUY_01989	MUY_04383
MUY_01989	MUY_04433
MUY_01989	MUY_04483
MUY_01989	MUY_00450
MUY_01989	MUY_00464
MUY_01989	MUY_00808
MUY_01989	MUY_00840
MUY_01991	MUY_01991
MUY_01991	MUY_03150
MUY_01991	MUY_03247
MUY_01991	MUY_03790
MUY_01991	MUY_04099
MUY_01991	MUY_04305
MUY_01991	MUY_00630
MUY_01992	MUY_01992
MUY_01992	MUY_03149
MUY_01992	MUY_03246
MUY_01992	MUY_03791
MUY_01992	MUY_04098
MUY_01992	MUY_04304
MUY_01993	MUY_01993
MUY_01993	MUY_02659
MUY_00190	MUY_00190
MUY_00190	MUY_02094
MUY_00190	MUY_02152
MUY_00190	MUY_02439
MUY_00190	MUY_02467
MUY_00190	MUY_02627
MUY_00190	MUY_00261
MUY_00190	MUY_00308
MUY_00190	MUY_03333
MUY_00190	MUY_03386
MUY_00190	MUY_03449
MUY_00190	MUY_03454
MUY_00190	MUY_03637
MUY_00190	MUY_03810
MUY_00190	MUY_04070
MUY_00190	MUY_04242
MUY_00190	MUY_00382
MUY_00190	MUY_04302
MUY_00190	MUY_04383
MUY_00190	MUY_04433
MUY_00190	MUY_04483
MUY_00190	MUY_00450
MUY_00190	MUY_00464
MUY_00190	MUY_00808
MUY_00190	MUY_00840

MUY_01999	MUY_01999
MUY_01999	MUY_02036
MUY_01999	MUY_02049
MUY_01999	MUY_02231
MUY_01999	MUY_02238
MUY_01999	MUY_02297
MUY_01999	MUY_02580
MUY_01999	MUY_02982
MUY_01999	MUY_03140
MUY_01999	MUY_03250
MUY_01999	MUY_03431
MUY_01999	MUY_00323
MUY_01999	MUY_03553
MUY_01999	MUY_03612
MUY_01999	MUY_00445
MUY_01999	MUY_00677
MUY_01999	MUY_00749
MUY_01999	MUY_00779
MUY_01999	MUY_00882
MUY_02000	MUY_02000
MUY_02000	MUY_00866
MUY_00191	MUY_00191
MUY_00191	MUY_02245
MUY_00191	MUY_03066
MUY_00191	MUY_00449
MUY_00191	MUY_00828
MUY_02012	MUY_02012
MUY_02012	MUY_03091
MUY_02012	MUY_03093
MUY_02012	MUY_00024
MUY_02012	MUY_00036
MUY_02014	MUY_02014
MUY_02015	MUY_02015
MUY_02015	MUY_02016
MUY_02015	MUY_02134
MUY_02015	MUY_00241
MUY_02015	MUY_02947
MUY_02015	MUY_00277
MUY_02015	MUY_00337
MUY_02015	MUY_04086
MUY_02015	MUY_00433
MUY_02015	MUY_00813
MUY_02016	MUY_02016
MUY_02016	MUY_00248
MUY_02016	MUY_03267
MUY_02025	MUY_02025
MUY_02025	MUY_02289

MUY_02025	MUY_02620
MUY_02025	MUY_03341
MUY_02025	MUY_03375
MUY_02025	MUY_03451
MUY_02025	MUY_03775
MUY_02025	MUY_00378
MUY_02025	MUY_04230
MUY_02025	MUY_00526
MUY_02025	MUY_00857
MUY_00193	MUY_00193
MUY_00193	MUY_02234
MUY_02028	MUY_02028
MUY_02030	MUY_02030
MUY_02030	MUY_02361
MUY_02030	MUY_02588
MUY_02030	MUY_00297
MUY_02030	MUY_03518
MUY_02030	MUY_03667
MUY_02030	MUY_04463
MUY_02030	MUY_00557
MUY_02030	MUY_00583
MUY_02030	MUY_00674
MUY_00194	MUY_00194
MUY_00194	MUY_02151
MUY_00194	MUY_02208
MUY_00194	MUY_02209
MUY_00194	MUY_02225
MUY_00194	MUY_02597
MUY_00194	MUY_02598
MUY_00194	MUY_02609
MUY_00194	MUY_02643
MUY_00194	MUY_02700
MUY_00194	MUY_02744
MUY_00194	MUY_02745
MUY_00194	MUY_02760
MUY_00194	MUY_00265
MUY_00194	MUY_02894
MUY_00194	MUY_02929
MUY_00194	MUY_02945
MUY_00194	MUY_02946
MUY_00194	MUY_00285
MUY_00194	MUY_03154
MUY_00194	MUY_03330
MUY_00194	MUY_03332
MUY_00194	MUY_03354
MUY_00194	MUY_00322
MUY_00194	MUY_03575

MUY_00194	MUY_03576
MUY_00194	MUY_03582
MUY_00194	MUY_03598
MUY_00194	MUY_03634
MUY_00194	MUY_03649
MUY_00194	MUY_03676
MUY_00194	MUY_03785
MUY_00194	MUY_03805
MUY_00194	MUY_03900
MUY_00194	MUY_03946
MUY_00194	MUY_00372
MUY_00194	MUY_03978
MUY_00194	MUY_04144
MUY_00194	MUY_04172
MUY_00194	MUY_04248
MUY_00194	MUY_04254
MUY_00194	MUY_04266
MUY_00194	MUY_04269
MUY_00194	MUY_04282
MUY_00194	MUY_04296
MUY_00194	MUY_00388
MUY_00194	MUY_04348
MUY_00194	MUY_04357
MUY_00194	MUY_00394
MUY_00194	MUY_04403
MUY_00194	MUY_04416
MUY_00194	MUY_04445
MUY_00194	MUY_04453
MUY_00194	MUY_00420
MUY_00194	MUY_00434
MUY_00194	MUY_00442
MUY_00194	MUY_00516
MUY_00194	MUY_00585
MUY_00194	MUY_00800
MUY_00194	MUY_00815
MUY_00194	MUY_00937
MUY_00194	MUY_00938
MUY_00194	MUY_00939
MUY_00194	MUY_00968
MUY_00194	MUY_00975
MUY_00194	MUY_00996
MUY_00194	MUY_01000
MUY_02036	MUY_02036
MUY_02036	MUY_02049
MUY_02036	MUY_02231
MUY_02036	MUY_02238
MUY_02036	MUY_02297

MUY_02036	MUY_02580
MUY_02036	MUY_02982
MUY_02036	MUY_03140
MUY_02036	MUY_03250
MUY_02036	MUY_03431
MUY_02036	MUY_00323
MUY_02036	MUY_03553
MUY_02036	MUY_03612
MUY_02036	MUY_00445
MUY_02036	MUY_00677
MUY_02036	MUY_00749
MUY_02036	MUY_00779
MUY_02036	MUY_00882
MUY_02039	MUY_02039
MUY_02039	MUY_02115
MUY_02039	MUY_02118
MUY_02039	MUY_02124
MUY_02039	MUY_02307
MUY_02039	MUY_02411
MUY_02039	MUY_02574
MUY_02039	MUY_00250
MUY_02039	MUY_03442
MUY_02039	MUY_04171
MUY_02039	MUY_04318
MUY_02039	MUY_04410
MUY_02039	MUY_00504
MUY_02039	MUY_00782
MUY_02039	MUY_00920
MUY_02040	MUY_02040
MUY_02043	MUY_02043
MUY_02043	MUY_02051
MUY_02043	MUY_02077
MUY_02043	MUY_02333
MUY_02043	MUY_02526
MUY_02043	MUY_02896
MUY_02043	MUY_04163
MUY_02043	MUY_04439
MUY_02043	MUY_04501
MUY_02043	MUY_04505
MUY_02043	MUY_00447
MUY_02043	MUY_00856
MUY_02043	MUY_00917
MUY_02043	MUY_00945
MUY_02045	MUY_02045
MUY_02045	MUY_04018
MUY_02047	MUY_02047
MUY_02047	MUY_04028

MUY_02049	MUY_02049
MUY_02049	MUY_02231
MUY_02049	MUY_02238
MUY_02049	MUY_02297
MUY_02049	MUY_02580
MUY_02049	MUY_02982
MUY_02049	MUY_03140
MUY_02049	MUY_03250
MUY_02049	MUY_03431
MUY_02049	MUY_00323
MUY_02049	MUY_03553
MUY_02049	MUY_03612
MUY_02049	MUY_00445
MUY_02049	MUY_00677
MUY_02049	MUY_00749
MUY_02049	MUY_00779
MUY_02049	MUY_00882
MUY_02050	MUY_02050
MUY_02050	MUY_02216
MUY_02050	MUY_02232
MUY_02050	MUY_02364
MUY_02050	MUY_02695
MUY_02050	MUY_00272
MUY_02050	MUY_03126
MUY_02050	MUY_03240
MUY_02050	MUY_03659
MUY_02050	MUY_03701
MUY_02050	MUY_03880
MUY_02050	MUY_04014
MUY_02050	MUY_04194
MUY_02050	MUY_00556
MUY_02050	MUY_00710
MUY_02051	MUY_02051
MUY_02051	MUY_02077
MUY_02051	MUY_02333
MUY_02051	MUY_02526
MUY_02051	MUY_04163
MUY_02051	MUY_04439
MUY_02051	MUY_00447
MUY_02051	MUY_00856
MUY_02051	MUY_00917
MUY_02051	MUY_00945
MUY_02052	MUY_02052
MUY_02052	MUY_02606
MUY_02052	MUY_04085
MUY_02052	MUY_04206
MUY_02052	MUY_04410

MUY_02052	MUY_00695
MUY_02052	MUY_00906
MUY_02053	MUY_02053
MUY_02053	MUY_03851
MUY_02054	MUY_02054
MUY_02054	MUY_04188
MUY_02054	MUY_00993
MUY_02056	MUY_02056
MUY_02058	MUY_00256
MUY_02058	MUY_02797
MUY_02058	MUY_00435
MUY_02061	MUY_02061
MUY_02062	MUY_02062
MUY_02062	MUY_02258
MUY_02062	MUY_02321
MUY_02062	MUY_02508
MUY_02062	MUY_02515
MUY_02062	MUY_00021
MUY_02062	MUY_03050
MUY_02064	MUY_00040
MUY_02065	MUY_02065
MUY_02065	MUY_02649
MUY_02065	MUY_03267
MUY_02065	MUY_04476
MUY_02065	MUY_00472
MUY_02065	MUY_00473
MUY_02065	MUY_00894
MUY_00195	MUY_00195
MUY_02066	MUY_00002
MUY_02076	MUY_03267
MUY_02077	MUY_02077
MUY_02077	MUY_02333
MUY_02077	MUY_02526
MUY_02077	MUY_02896
MUY_02077	MUY_04011
MUY_02077	MUY_04163
MUY_02077	MUY_04439
MUY_02077	MUY_04501
MUY_02077	MUY_04505
MUY_02077	MUY_00447
MUY_02077	MUY_00856
MUY_02077	MUY_00917
MUY_02077	MUY_00945
MUY_02081	MUY_02081
MUY_02081	MUY_03129
MUY_02081	MUY_03476
MUY_00197	MUY_00197

MUY_00197	MUY_03065
MUY_02090	MUY_02090
MUY_02091	MUY_02092
MUY_02092	MUY_02092
MUY_02094	MUY_02094
MUY_02094	MUY_02152
MUY_02094	MUY_02439
MUY_02094	MUY_02467
MUY_02094	MUY_02627
MUY_02094	MUY_00261
MUY_02094	MUY_00308
MUY_02094	MUY_03333
MUY_02094	MUY_03386
MUY_02094	MUY_03449
MUY_02094	MUY_03454
MUY_02094	MUY_03637
MUY_02094	MUY_03810
MUY_02094	MUY_04070
MUY_02094	MUY_04242
MUY_02094	MUY_00382
MUY_02094	MUY_04302
MUY_02094	MUY_04383
MUY_02094	MUY_04433
MUY_02094	MUY_04483
MUY_02094	MUY_00450
MUY_02094	MUY_00464
MUY_02094	MUY_00808
MUY_02094	MUY_00840
MUY_02095	MUY_02095
MUY_02095	MUY_03170
MUY_02095	MUY_03691
MUY_02095	MUY_04192
MUY_02095	MUY_04434
MUY_02096	MUY_02096
MUY_02096	MUY_03169
MUY_02096	MUY_03694
MUY_02096	MUY_04353
MUY_02097	MUY_03171
MUY_00199	MUY_00199
MUY_00199	MUY_03287
MUY_02100	MUY_02100
MUY_02100	MUY_02153
MUY_02100	MUY_02211
MUY_02100	MUY_02331
MUY_02100	MUY_02615
MUY_02100	MUY_02633
MUY_02100	MUY_02950

MUY_02100	MUY_02958
MUY_02100	MUY_00292
MUY_02100	MUY_03128
MUY_02100	MUY_03225
MUY_02100	MUY_00313
MUY_02100	MUY_03494
MUY_02100	MUY_03606
MUY_02100	MUY_03627
MUY_02100	MUY_03638
MUY_02100	MUY_03964
MUY_02100	MUY_03998
MUY_02100	MUY_04038
MUY_02100	MUY_00486
MUY_02100	MUY_00681
MUY_02100	MUY_00933
MUY_00013	MUY_00013
MUY_00013	MUY_03267
MUY_02105	MUY_02108
MUY_02106	MUY_02107
MUY_02106	MUY_02108
MUY_02107	MUY_02107
MUY_02107	MUY_02108
MUY_02107	MUY_00344
MUY_02108	MUY_02108
MUY_02108	MUY_02214
MUY_02108	MUY_02215
MUY_02108	MUY_02268
MUY_02108	MUY_02460
MUY_02108	MUY_02745
MUY_02108	MUY_04052
MUY_02108	MUY_00460
MUY_00200	MUY_00200
MUY_00200	MUY_02834
MUY_00200	MUY_03186
MUY_00200	MUY_03876
MUY_00200	MUY_04337
MUY_00200	MUY_00708
MUY_02110	MUY_02110
MUY_02115	MUY_02115
MUY_02115	MUY_02118
MUY_02115	MUY_02124
MUY_02115	MUY_02307
MUY_02115	MUY_02411
MUY_02115	MUY_02574
MUY_02115	MUY_00250
MUY_02115	MUY_03041
MUY_02115	MUY_03442

MUY_02115	MUY_00366
MUY_02115	MUY_04171
MUY_02115	MUY_04318
MUY_02115	MUY_00397
MUY_02115	MUY_00504
MUY_02115	MUY_00782
MUY_02115	MUY_00072
MUY_02115	MUY_00920
MUY_00003	MUY_00003
MUY_00003	MUY_02635
MUY_00003	MUY_02821
MUY_00003	MUY_03267
MUY_00003	MUY_00038
MUY_02118	MUY_02118
MUY_02118	MUY_02124
MUY_02118	MUY_02307
MUY_02118	MUY_02411
MUY_02118	MUY_02574
MUY_02118	MUY_00250
MUY_02118	MUY_03041
MUY_02118	MUY_03442
MUY_02118	MUY_00366
MUY_02118	MUY_04171
MUY_02118	MUY_04318
MUY_02118	MUY_00397
MUY_02118	MUY_00504
MUY_02118	MUY_00782
MUY_02118	MUY_00072
MUY_02118	MUY_00920
MUY_02120	MUY_03096
MUY_02124	MUY_02124
MUY_02124	MUY_02307
MUY_02124	MUY_02411
MUY_02124	MUY_02574
MUY_02124	MUY_00250
MUY_02124	MUY_03442
MUY_02124	MUY_04171
MUY_02124	MUY_04318
MUY_02124	MUY_04410
MUY_02124	MUY_00504
MUY_02124	MUY_00782
MUY_02124	MUY_00920
MUY_02125	MUY_02125
MUY_02127	MUY_02127
MUY_02127	MUY_02171
MUY_02127	MUY_02201
MUY_02127	MUY_02269

MUY_02127	MUY_02460
MUY_02127	MUY_02619
MUY_02127	MUY_02648
MUY_02127	MUY_02697
MUY_02127	MUY_02841
MUY_02127	MUY_02914
MUY_02127	MUY_02917
MUY_02127	MUY_02941
MUY_02127	MUY_03136
MUY_02127	MUY_03137
MUY_02127	MUY_03138
MUY_02127	MUY_03279
MUY_02127	MUY_03304
MUY_02127	MUY_00316
MUY_02127	MUY_03513
MUY_02127	MUY_03515
MUY_02127	MUY_03524
MUY_02127	MUY_03585
MUY_02127	MUY_03706
MUY_02127	MUY_03840
MUY_02127	MUY_00459
MUY_02127	MUY_00461
MUY_02127	MUY_00523
MUY_02127	MUY_00895
MUY_02128	MUY_02128
MUY_02128	MUY_02827
MUY_02129	MUY_02129
MUY_02129	MUY_03015
MUY_02129	MUY_03490
MUY_02129	MUY_04046
MUY_02132	MUY_02132
MUY_02132	MUY_00344
MUY_02132	MUY_00554
MUY_02134	MUY_02134
MUY_02134	MUY_02534
MUY_02134	MUY_00241
MUY_02134	MUY_02947
MUY_02134	MUY_00277
MUY_02134	MUY_00337
MUY_02134	MUY_04086
MUY_02134	MUY_00433
MUY_02134	MUY_00813
MUY_02135	MUY_02135
MUY_02135	MUY_02963
MUY_02135	MUY_03683
MUY_02135	MUY_03856
MUY_02135	MUY_03980

MUY_02135	MUY_00483
MUY_02136	MUY_02136
MUY_02136	MUY_02622
MUY_02136	MUY_03257
MUY_02138	MUY_02138
MUY_02138	MUY_02139
MUY_02138	MUY_02178
MUY_02138	MUY_03139
MUY_02138	MUY_03374
MUY_02139	MUY_02139
MUY_02139	MUY_02178
MUY_02139	MUY_03139
MUY_02139	MUY_03374
MUY_02140	MUY_02140
MUY_02140	MUY_02305
MUY_02140	MUY_00258
MUY_02140	MUY_03400
MUY_02140	MUY_00347
MUY_02140	MUY_04132
MUY_02140	MUY_04150
MUY_02140	MUY_04397
MUY_02140	MUY_04438
MUY_02140	MUY_00453
MUY_02140	MUY_00797
MUY_02141	MUY_02141
MUY_02141	MUY_02634
MUY_02141	MUY_04436
MUY_02142	MUY_02142
MUY_02142	MUY_02183
MUY_02142	MUY_03137
MUY_02142	MUY_03138
MUY_02142	MUY_03590
MUY_02142	MUY_04103
MUY_02142	MUY_04104
MUY_02142	MUY_00519
MUY_02144	MUY_02144
MUY_02144	MUY_03955
MUY_02145	MUY_03683
MUY_02145	MUY_00483
MUY_02149	MUY_02149
MUY_02149	MUY_02242
MUY_02149	MUY_02447
MUY_02149	MUY_02696
MUY_02149	MUY_02788
MUY_02149	MUY_03055
MUY_02149	MUY_04126
MUY_02149	MUY_04239

MUY_02149	MUY_00469
MUY_02149	MUY_00485
MUY_02149	MUY_00696
MUY_02151	MUY_02151
MUY_02151	MUY_02208
MUY_02151	MUY_02209
MUY_02151	MUY_02225
MUY_02151	MUY_02597
MUY_02151	MUY_02598
MUY_02151	MUY_02609
MUY_02151	MUY_02643
MUY_02151	MUY_02700
MUY_02151	MUY_02744
MUY_02151	MUY_02745
MUY_02151	MUY_02760
MUY_02151	MUY_00265
MUY_02151	MUY_02894
MUY_02151	MUY_02929
MUY_02151	MUY_02945
MUY_02151	MUY_02946
MUY_02151	MUY_00285
MUY_02151	MUY_03154
MUY_02151	MUY_03330
MUY_02151	MUY_03332
MUY_02151	MUY_03354
MUY_02151	MUY_00322
MUY_02151	MUY_03575
MUY_02151	MUY_03576
MUY_02151	MUY_03582
MUY_02151	MUY_03598
MUY_02151	MUY_03634
MUY_02151	MUY_03649
MUY_02151	MUY_03676
MUY_02151	MUY_03785
MUY_02151	MUY_03805
MUY_02151	MUY_03900
MUY_02151	MUY_03946
MUY_02151	MUY_00372
MUY_02151	MUY_03978
MUY_02151	MUY_04144
MUY_02151	MUY_04172
MUY_02151	MUY_04248
MUY_02151	MUY_04254
MUY_02151	MUY_04266
MUY_02151	MUY_04269
MUY_02151	MUY_04282
MUY_02151	MUY_04296

MUY_02151	MUY_00388
MUY_02151	MUY_04348
MUY_02151	MUY_04357
MUY_02151	MUY_00394
MUY_02151	MUY_04403
MUY_02151	MUY_04416
MUY_02151	MUY_04445
MUY_02151	MUY_04453
MUY_02151	MUY_00420
MUY_02151	MUY_00434
MUY_02151	MUY_00442
MUY_02151	MUY_00516
MUY_02151	MUY_00585
MUY_02151	MUY_00800
MUY_02151	MUY_00815
MUY_02151	MUY_00937
MUY_02151	MUY_00938
MUY_02151	MUY_00939
MUY_02151	MUY_00968
MUY_02151	MUY_00975
MUY_02151	MUY_00996
MUY_02151	MUY_01000
MUY_02152	MUY_02152
MUY_02152	MUY_02439
MUY_02152	MUY_02467
MUY_02152	MUY_02627
MUY_02152	MUY_00261
MUY_02152	MUY_00308
MUY_02152	MUY_03333
MUY_02152	MUY_03386
MUY_02152	MUY_03449
MUY_02152	MUY_03454
MUY_02152	MUY_03637
MUY_02152	MUY_03810
MUY_02152	MUY_04070
MUY_02152	MUY_04242
MUY_02152	MUY_00382
MUY_02152	MUY_04302
MUY_02152	MUY_04383
MUY_02152	MUY_04433
MUY_02152	MUY_04483
MUY_02152	MUY_00450
MUY_02152	MUY_00464
MUY_02152	MUY_00808
MUY_02152	MUY_00840
MUY_00204	MUY_00204
MUY_02153	MUY_02153

MUY_02153	MUY_02211
MUY_02153	MUY_02331
MUY_02153	MUY_02615
MUY_02153	MUY_02633
MUY_02153	MUY_02950
MUY_02153	MUY_02958
MUY_02153	MUY_00292
MUY_02153	MUY_03128
MUY_02153	MUY_03225
MUY_02153	MUY_00313
MUY_02153	MUY_03494
MUY_02153	MUY_03606
MUY_02153	MUY_03627
MUY_02153	MUY_03638
MUY_02153	MUY_03964
MUY_02153	MUY_03998
MUY_02153	MUY_04038
MUY_02153	MUY_00486
MUY_02153	MUY_00681
MUY_02153	MUY_00933
MUY_02154	MUY_02154
MUY_02154	MUY_03036
MUY_02154	MUY_00344
MUY_02159	MUY_02159
MUY_02159	MUY_03267
MUY_02159	MUY_03644
MUY_02159	MUY_03962
MUY_02159	MUY_03975
MUY_02159	MUY_04082
MUY_02159	MUY_04328
MUY_02159	MUY_04428
MUY_02159	MUY_00700
MUY_02160	MUY_02160
MUY_02160	MUY_02914
MUY_02160	MUY_03136
MUY_02160	MUY_03279
MUY_02160	MUY_03585
MUY_02160	MUY_04073
MUY_02160	MUY_00523
MUY_02160	MUY_00861
MUY_00205	MUY_00205
MUY_00205	MUY_00206
MUY_00205	MUY_02178
MUY_00205	MUY_02366
MUY_00205	MUY_00229
MUY_00205	MUY_02521
MUY_00205	MUY_02523

MUY_00205	MUY_00249
MUY_00205	MUY_02655
MUY_00205	MUY_02663
MUY_00205	MUY_00263
MUY_00205	MUY_00264
MUY_00205	MUY_02912
MUY_00205	MUY_02939
MUY_00205	MUY_02940
MUY_00205	MUY_02964
MUY_00205	MUY_00288
MUY_00205	MUY_00289
MUY_00205	MUY_03059
MUY_00205	MUY_03117
MUY_00205	MUY_03127
MUY_00205	MUY_03139
MUY_00205	MUY_03183
MUY_00205	MUY_03201
MUY_00205	MUY_03202
MUY_00205	MUY_03252
MUY_00205	MUY_03306
MUY_00205	MUY_03374
MUY_00205	MUY_03463
MUY_00205	MUY_03477
MUY_00205	MUY_03478
MUY_00205	MUY_03483
MUY_00205	MUY_03609
MUY_00205	MUY_03619
MUY_00205	MUY_03647
MUY_00205	MUY_03648
MUY_00205	MUY_03924
MUY_00205	MUY_04096
MUY_00205	MUY_04146
MUY_00205	MUY_04177
MUY_00205	MUY_04211
MUY_00205	MUY_04212
MUY_00205	MUY_04293
MUY_00205	MUY_04294
MUY_00205	MUY_04358
MUY_00205	MUY_04359
MUY_00205	MUY_04379
MUY_00205	MUY_04417
MUY_00205	MUY_04418
MUY_00205	MUY_04447
MUY_00205	MUY_04448
MUY_00205	MUY_04471
MUY_00205	MUY_00418
MUY_00205	MUY_00436

MUY_00205	MUY_00575
MUY_00205	MUY_00576
MUY_00205	MUY_00790
MUY_00205	MUY_00961
MUY_02162	MUY_02162
MUY_02163	MUY_02163
MUY_02163	MUY_03525
MUY_02164	MUY_02164
MUY_02164	MUY_04012
MUY_02167	MUY_00206
MUY_02167	MUY_02671
MUY_00206	MUY_00206
MUY_00206	MUY_02797
MUY_00206	MUY_02821
MUY_00206	MUY_03035
MUY_00206	MUY_03227
MUY_00206	MUY_03462
MUY_00206	MUY_03803
MUY_00206	MUY_03984
MUY_00206	MUY_04086
MUY_00206	MUY_04334
MUY_00206	MUY_00402
MUY_00206	MUY_00780
MUY_02168	MUY_02445
MUY_02168	MUY_00344
MUY_02168	MUY_03684
MUY_02168	MUY_00699
MUY_02169	MUY_02169
MUY_02169	MUY_02703
MUY_02169	MUY_03096
MUY_02169	MUY_00344
MUY_02169	MUY_04182
MUY_02169	MUY_04324
MUY_02169	MUY_04482
MUY_02170	MUY_02170
MUY_02171	MUY_02171
MUY_02171	MUY_02201
MUY_02171	MUY_02269
MUY_02171	MUY_02460
MUY_02171	MUY_02619
MUY_02171	MUY_02648
MUY_02171	MUY_02697
MUY_02171	MUY_02841
MUY_02171	MUY_02914
MUY_02171	MUY_02917
MUY_02171	MUY_02941
MUY_02171	MUY_03136

MUY_02171	MUY_03137
MUY_02171	MUY_03138
MUY_02171	MUY_03279
MUY_02171	MUY_03304
MUY_02171	MUY_00316
MUY_02171	MUY_03513
MUY_02171	MUY_03515
MUY_02171	MUY_03524
MUY_02171	MUY_03585
MUY_02171	MUY_03706
MUY_02171	MUY_03840
MUY_02171	MUY_00459
MUY_02171	MUY_00461
MUY_02171	MUY_00523
MUY_02171	MUY_00895
MUY_02174	MUY_02174
MUY_02174	MUY_00662
MUY_02175	MUY_02175
MUY_02175	MUY_03146
MUY_02175	MUY_03610
MUY_02175	MUY_04508
MUY_02176	MUY_02176
MUY_02176	MUY_02182
MUY_02176	MUY_02410
MUY_02176	MUY_00273
MUY_02176	MUY_03141
MUY_02176	MUY_03241
MUY_02176	MUY_03261
MUY_02176	MUY_03373
MUY_02176	MUY_00376
MUY_02176	MUY_04033
MUY_02176	MUY_04034
MUY_02176	MUY_04036
MUY_02176	MUY_00377
MUY_02176	MUY_00378
MUY_02176	MUY_04228
MUY_02176	MUY_04230
MUY_02176	MUY_00379
MUY_02177	MUY_02177
MUY_02177	MUY_02316
MUY_02177	MUY_02639
MUY_02177	MUY_03213
MUY_02177	MUY_03214
MUY_02178	MUY_02178
MUY_02178	MUY_02521
MUY_02178	MUY_02523
MUY_02178	MUY_00249

MUY_02178	MUY_02663
MUY_02178	MUY_00263
MUY_02178	MUY_02912
MUY_02178	MUY_02940
MUY_02178	MUY_00288
MUY_02178	MUY_03139
MUY_02178	MUY_03183
MUY_02178	MUY_03202
MUY_02178	MUY_03374
MUY_02178	MUY_03463
MUY_02178	MUY_03477
MUY_02178	MUY_03591
MUY_02178	MUY_03592
MUY_02178	MUY_03609
MUY_02178	MUY_03619
MUY_02178	MUY_03648
MUY_02178	MUY_03924
MUY_02178	MUY_04096
MUY_02178	MUY_04105
MUY_02178	MUY_04106
MUY_02178	MUY_04146
MUY_02178	MUY_04177
MUY_02178	MUY_04212
MUY_02178	MUY_04293
MUY_02178	MUY_04359
MUY_02178	MUY_04418
MUY_02178	MUY_04448
MUY_02178	MUY_04471
MUY_02178	MUY_00418
MUY_02178	MUY_00436
MUY_02178	MUY_00576
MUY_02179	MUY_02179
MUY_02179	MUY_03099
MUY_02181	MUY_02181
MUY_02181	MUY_02316
MUY_02181	MUY_02675
MUY_02182	MUY_02182
MUY_02182	MUY_02410
MUY_02182	MUY_00273
MUY_02182	MUY_03141
MUY_02182	MUY_03241
MUY_02182	MUY_03261
MUY_02182	MUY_03373
MUY_02182	MUY_00376
MUY_02182	MUY_04033
MUY_02182	MUY_04034
MUY_02182	MUY_04036

MUY_02182	MUY_00377
MUY_02182	MUY_00378
MUY_02182	MUY_04228
MUY_02182	MUY_04230
MUY_02182	MUY_00379
MUY_02183	MUY_02183
MUY_02183	MUY_03137
MUY_02183	MUY_03138
MUY_02183	MUY_03590
MUY_02183	MUY_04103
MUY_02183	MUY_04104
MUY_02183	MUY_00519
MUY_02184	MUY_02184
MUY_02184	MUY_02301
MUY_02184	MUY_03704
MUY_02184	MUY_03831
MUY_02184	MUY_00360
MUY_02184	MUY_00911
MUY_02185	MUY_02185
MUY_02185	MUY_02332
MUY_02185	MUY_02937
MUY_02185	MUY_02972
MUY_02185	MUY_03839
MUY_02185	MUY_04049
MUY_02185	MUY_00560
MUY_02185	MUY_00855
MUY_02185	MUY_00904
MUY_00207	MUY_00207
MUY_00207	MUY_02203
MUY_00207	MUY_02978
MUY_00207	MUY_00290
MUY_00207	MUY_03320
MUY_00207	MUY_03640
MUY_00207	MUY_03984
MUY_00207	MUY_04198
MUY_00207	MUY_00401
MUY_00207	MUY_00563
MUY_02187	MUY_02187
MUY_02187	MUY_03134
MUY_02187	MUY_04317
MUY_02187	MUY_00439
MUY_02188	MUY_02188
MUY_02188	MUY_02617
MUY_02188	MUY_02826
MUY_02190	MUY_02190
MUY_02190	MUY_00242
MUY_02190	MUY_03031

MUY_02190	MUY_03440
MUY_00014	MUY_00014
MUY_00014	MUY_03020
MUY_00014	MUY_03872
MUY_00014	MUY_00378
MUY_00014	MUY_04230
MUY_02193	MUY_02193
MUY_02193	MUY_02816
MUY_02193	MUY_02914
MUY_02193	MUY_03136
MUY_02193	MUY_03279
MUY_02193	MUY_03585
MUY_02193	MUY_00523
MUY_02194	MUY_02194
MUY_02196	MUY_02196
MUY_02196	MUY_03372
MUY_02196	MUY_04216
MUY_02198	MUY_02198
MUY_02198	MUY_00256
MUY_02198	MUY_02780
MUY_02198	MUY_02797
MUY_02198	MUY_02983
MUY_02198	MUY_03069
MUY_02198	MUY_03314
MUY_02198	MUY_03378
MUY_02198	MUY_03496
MUY_02198	MUY_03610
MUY_02198	MUY_03620
MUY_02198	MUY_03789
MUY_02198	MUY_03819
MUY_02198	MUY_03821
MUY_02198	MUY_03824
MUY_02198	MUY_03931
MUY_02198	MUY_03967
MUY_02198	MUY_04086
MUY_02198	MUY_04152
MUY_02198	MUY_04334
MUY_02198	MUY_04466
MUY_02198	MUY_00435
MUY_02198	MUY_00514
MUY_02198	MUY_00590
MUY_02198	MUY_00780
MUY_02198	MUY_00895
MUY_02198	MUY_00927
MUY_02201	MUY_02201
MUY_02201	MUY_02941
MUY_02201	MUY_03267

MUY_02201	MUY_03513
MUY_02201	MUY_03515
MUY_02201	MUY_03524
MUY_02201	MUY_03567
MUY_02201	MUY_00461
MUY_02202	MUY_02202
MUY_02202	MUY_02413
MUY_02202	MUY_03025
MUY_02202	MUY_03267
MUY_02202	MUY_00743
MUY_02202	MUY_00074
MUY_02203	MUY_02203
MUY_02203	MUY_02978
MUY_02203	MUY_00290
MUY_02203	MUY_03320
MUY_02203	MUY_03640
MUY_02203	MUY_03984
MUY_02203	MUY_04198
MUY_02203	MUY_00401
MUY_02203	MUY_00563
MUY_02206	MUY_02206
MUY_02206	MUY_03415
MUY_02207	MUY_02207
MUY_02207	MUY_03786
MUY_02207	MUY_04432
MUY_02207	MUY_00423
MUY_02208	MUY_02208
MUY_02208	MUY_02209
MUY_02208	MUY_02225
MUY_02208	MUY_02597
MUY_02208	MUY_02598
MUY_02208	MUY_02609
MUY_02208	MUY_02643
MUY_02208	MUY_02700
MUY_02208	MUY_02744
MUY_02208	MUY_02745
MUY_02208	MUY_02760
MUY_02208	MUY_00265
MUY_02208	MUY_02894
MUY_02208	MUY_02929
MUY_02208	MUY_02945
MUY_02208	MUY_02946
MUY_02208	MUY_00285
MUY_02208	MUY_03154
MUY_02208	MUY_03330
MUY_02208	MUY_03332
MUY_02208	MUY_03354

MUY_02208	MUY_00322
MUY_02208	MUY_03575
MUY_02208	MUY_03576
MUY_02208	MUY_03582
MUY_02208	MUY_03598
MUY_02208	MUY_03634
MUY_02208	MUY_03649
MUY_02208	MUY_03676
MUY_02208	MUY_03785
MUY_02208	MUY_03805
MUY_02208	MUY_03900
MUY_02208	MUY_03946
MUY_02208	MUY_00372
MUY_02208	MUY_03978
MUY_02208	MUY_04144
MUY_02208	MUY_04172
MUY_02208	MUY_04248
MUY_02208	MUY_04254
MUY_02208	MUY_04266
MUY_02208	MUY_04269
MUY_02208	MUY_04282
MUY_02208	MUY_04296
MUY_02208	MUY_00388
MUY_02208	MUY_04348
MUY_02208	MUY_04357
MUY_02208	MUY_00394
MUY_02208	MUY_04403
MUY_02208	MUY_04416
MUY_02208	MUY_04445
MUY_02208	MUY_04453
MUY_02208	MUY_00420
MUY_02208	MUY_00434
MUY_02208	MUY_00442
MUY_02208	MUY_00516
MUY_02208	MUY_00585
MUY_02208	MUY_00800
MUY_02208	MUY_00815
MUY_02208	MUY_00937
MUY_02208	MUY_00938
MUY_02208	MUY_00939
MUY_02208	MUY_00968
MUY_02208	MUY_00975
MUY_02208	MUY_00996
MUY_02208	MUY_01000
MUY_02209	MUY_02209
MUY_02209	MUY_02225
MUY_02209	MUY_02597

MUY_02209	MUY_02598
MUY_02209	MUY_02609
MUY_02209	MUY_02643
MUY_02209	MUY_02700
MUY_02209	MUY_02744
MUY_02209	MUY_02745
MUY_02209	MUY_02760
MUY_02209	MUY_00265
MUY_02209	MUY_02894
MUY_02209	MUY_02929
MUY_02209	MUY_02945
MUY_02209	MUY_02946
MUY_02209	MUY_00285
MUY_02209	MUY_03154
MUY_02209	MUY_03330
MUY_02209	MUY_03332
MUY_02209	MUY_03354
MUY_02209	MUY_00322
MUY_02209	MUY_03575
MUY_02209	MUY_03576
MUY_02209	MUY_03582
MUY_02209	MUY_03598
MUY_02209	MUY_03634
MUY_02209	MUY_03649
MUY_02209	MUY_03676
MUY_02209	MUY_03785
MUY_02209	MUY_03805
MUY_02209	MUY_03900
MUY_02209	MUY_03946
MUY_02209	MUY_00372
MUY_02209	MUY_03978
MUY_02209	MUY_04144
MUY_02209	MUY_04172
MUY_02209	MUY_04248
MUY_02209	MUY_04254
MUY_02209	MUY_04266
MUY_02209	MUY_04269
MUY_02209	MUY_04282
MUY_02209	MUY_04296
MUY_02209	MUY_00388
MUY_02209	MUY_04348
MUY_02209	MUY_04357
MUY_02209	MUY_00394
MUY_02209	MUY_04403
MUY_02209	MUY_04416
MUY_02209	MUY_04445
MUY_02209	MUY_04453

MUY_02209	MUY_00420
MUY_02209	MUY_00434
MUY_02209	MUY_00442
MUY_02209	MUY_00516
MUY_02209	MUY_00585
MUY_02209	MUY_00800
MUY_02209	MUY_00815
MUY_02209	MUY_00937
MUY_02209	MUY_00938
MUY_02209	MUY_00939
MUY_02209	MUY_00968
MUY_02209	MUY_00975
MUY_02209	MUY_00996
MUY_02209	MUY_01000
MUY_02210	MUY_02210
MUY_02210	MUY_02981
MUY_02210	MUY_03699
MUY_02210	MUY_00378
MUY_02210	MUY_04230
MUY_02210	MUY_00750
MUY_02210	MUY_00811
MUY_02211	MUY_02211
MUY_02211	MUY_02331
MUY_02211	MUY_02615
MUY_02211	MUY_02633
MUY_02211	MUY_02950
MUY_02211	MUY_02958
MUY_02211	MUY_00292
MUY_02211	MUY_03128
MUY_02211	MUY_03225
MUY_02211	MUY_00313
MUY_02211	MUY_03494
MUY_02211	MUY_03606
MUY_02211	MUY_03627
MUY_02211	MUY_03638
MUY_02211	MUY_03964
MUY_02211	MUY_03998
MUY_02211	MUY_04038
MUY_02211	MUY_00486
MUY_02211	MUY_00681
MUY_02211	MUY_00933
MUY_02212	MUY_02212
MUY_02212	MUY_02296
MUY_02212	MUY_00678
MUY_02214	MUY_02214
MUY_02214	MUY_02215
MUY_02214	MUY_02268

MUY_02214	MUY_02269
MUY_02214	MUY_02841
MUY_02214	MUY_04052
MUY_02214	MUY_00460
MUY_02215	MUY_02215
MUY_02215	MUY_02268
MUY_02215	MUY_02460
MUY_02215	MUY_04052
MUY_02215	MUY_00460
MUY_02216	MUY_02216
MUY_02216	MUY_02232
MUY_02216	MUY_02364
MUY_02216	MUY_02695
MUY_02216	MUY_00272
MUY_02216	MUY_03126
MUY_02216	MUY_03240
MUY_02216	MUY_03659
MUY_02216	MUY_03701
MUY_02216	MUY_03880
MUY_02216	MUY_04014
MUY_02216	MUY_04194
MUY_02216	MUY_00556
MUY_02216	MUY_00710
MUY_02225	MUY_02225
MUY_02225	MUY_02597
MUY_02225	MUY_02598
MUY_02225	MUY_02609
MUY_02225	MUY_02643
MUY_02225	MUY_02700
MUY_02225	MUY_02744
MUY_02225	MUY_02745
MUY_02225	MUY_02760
MUY_02225	MUY_00265
MUY_02225	MUY_02894
MUY_02225	MUY_02929
MUY_02225	MUY_02945
MUY_02225	MUY_02946
MUY_02225	MUY_00285
MUY_02225	MUY_03154
MUY_02225	MUY_03330
MUY_02225	MUY_03332
MUY_02225	MUY_03354
MUY_02225	MUY_00322
MUY_02225	MUY_03575
MUY_02225	MUY_03576
MUY_02225	MUY_03582
MUY_02225	MUY_03598

MUY_02225	MUY_03634
MUY_02225	MUY_03649
MUY_02225	MUY_03676
MUY_02225	MUY_03785
MUY_02225	MUY_03805
MUY_02225	MUY_03900
MUY_02225	MUY_03946
MUY_02225	MUY_00372
MUY_02225	MUY_03978
MUY_02225	MUY_04144
MUY_02225	MUY_04172
MUY_02225	MUY_04248
MUY_02225	MUY_04254
MUY_02225	MUY_04266
MUY_02225	MUY_04269
MUY_02225	MUY_04282
MUY_02225	MUY_04296
MUY_02225	MUY_00388
MUY_02225	MUY_04348
MUY_02225	MUY_04357
MUY_02225	MUY_00394
MUY_02225	MUY_04403
MUY_02225	MUY_04416
MUY_02225	MUY_04445
MUY_02225	MUY_04453
MUY_02225	MUY_00420
MUY_02225	MUY_00434
MUY_02225	MUY_00442
MUY_02225	MUY_00516
MUY_02225	MUY_00585
MUY_02225	MUY_00800
MUY_02225	MUY_00815
MUY_02225	MUY_00937
MUY_02225	MUY_00938
MUY_02225	MUY_00939
MUY_02225	MUY_00968
MUY_02225	MUY_00975
MUY_02225	MUY_00996
MUY_02225	MUY_01000
MUY_02227	MUY_02227
MUY_02227	MUY_03424
MUY_02227	MUY_03426
MUY_02228	MUY_02228
MUY_02228	MUY_03157
MUY_02228	MUY_03277
MUY_02229	MUY_02229
MUY_02229	MUY_00275

MUY_02229	MUY_04159
MUY_02231	MUY_02231
MUY_02231	MUY_02238
MUY_02231	MUY_02297
MUY_02231	MUY_02580
MUY_02231	MUY_02982
MUY_02231	MUY_03140
MUY_02231	MUY_03250
MUY_02231	MUY_03431
MUY_02231	MUY_00323
MUY_02231	MUY_03553
MUY_02231	MUY_03612
MUY_02231	MUY_00445
MUY_02231	MUY_00677
MUY_02231	MUY_00749
MUY_02231	MUY_00779
MUY_02231	MUY_00882
MUY_02232	MUY_02232
MUY_02232	MUY_02348
MUY_02232	MUY_02364
MUY_02232	MUY_02695
MUY_02232	MUY_00272
MUY_02232	MUY_03126
MUY_02232	MUY_03240
MUY_02232	MUY_03659
MUY_02232	MUY_03701
MUY_02232	MUY_03880
MUY_02232	MUY_04014
MUY_02232	MUY_04194
MUY_02232	MUY_00556
MUY_02232	MUY_00710
MUY_02233	MUY_02233
MUY_02233	MUY_02448
MUY_02233	MUY_02797
MUY_02233	MUY_02829
MUY_02233	MUY_03251
MUY_02233	MUY_03847
MUY_02233	MUY_04122
MUY_02234	MUY_02234
MUY_02238	MUY_02238
MUY_02238	MUY_02297
MUY_02238	MUY_02580
MUY_02238	MUY_02982
MUY_02238	MUY_03140
MUY_02238	MUY_03250
MUY_02238	MUY_03431
MUY_02238	MUY_00323

MUY_02238	MUY_03553
MUY_02238	MUY_03612
MUY_02238	MUY_00445
MUY_02238	MUY_00677
MUY_02238	MUY_00749
MUY_02238	MUY_00779
MUY_02238	MUY_00882
MUY_02240	MUY_02240
MUY_02240	MUY_03314
MUY_02240	MUY_04043
MUY_02240	MUY_00385
MUY_02240	MUY_04343
MUY_02240	MUY_00722
MUY_02242	MUY_02242
MUY_02242	MUY_02447
MUY_02242	MUY_02696
MUY_02242	MUY_02788
MUY_02242	MUY_03055
MUY_02242	MUY_04126
MUY_02242	MUY_04239
MUY_02242	MUY_00469
MUY_02242	MUY_00485
MUY_02242	MUY_00696
MUY_02245	MUY_02245
MUY_02245	MUY_03066
MUY_02245	MUY_00449
MUY_02245	MUY_00828
MUY_02250	MUY_02250
MUY_02253	MUY_02253
MUY_02253	MUY_02394
MUY_02253	MUY_02740
MUY_02253	MUY_03823
MUY_02253	MUY_03936
MUY_02253	MUY_03949
MUY_02253	MUY_00849
MUY_02257	MUY_03519
MUY_02258	MUY_02258
MUY_02258	MUY_02321
MUY_02258	MUY_02508
MUY_02258	MUY_02515
MUY_02258	MUY_00021
MUY_02258	MUY_03050
MUY_02259	MUY_02259
MUY_02259	MUY_02424
MUY_02259	MUY_02510
MUY_02259	MUY_02765
MUY_02259	MUY_02869

MUY_02259	MUY_03012
MUY_02259	MUY_03586
MUY_02259	MUY_03863
MUY_02259	MUY_03904
MUY_02259	MUY_03922
MUY_02259	MUY_04367
MUY_02259	MUY_00496
MUY_02259	MUY_00046
MUY_02259	MUY_00527
MUY_02259	MUY_00816
MUY_02259	MUY_00979
MUY_02263	MUY_02263
MUY_02268	MUY_02268
MUY_02268	MUY_02460
MUY_02268	MUY_04052
MUY_02268	MUY_00460
MUY_02269	MUY_02269
MUY_02269	MUY_02651
MUY_02269	MUY_02841
MUY_02269	MUY_02941
MUY_02269	MUY_03513
MUY_02269	MUY_03515
MUY_02269	MUY_03524
MUY_02269	MUY_03862
MUY_02269	MUY_00461
MUY_00215	MUY_00215
MUY_00215	MUY_02339
MUY_00215	MUY_02984
MUY_00215	MUY_02993
MUY_00215	MUY_00792
MUY_02273	MUY_02273
MUY_02273	MUY_00376
MUY_02273	MUY_00377
MUY_02273	MUY_00379
MUY_02285	MUY_02285
MUY_02285	MUY_03113
MUY_02285	MUY_03114
MUY_00216	MUY_00216
MUY_00216	MUY_00344
MUY_00216	MUY_00817
MUY_02286	MUY_02286
MUY_02286	MUY_00754
MUY_02289	MUY_02289
MUY_02289	MUY_02620
MUY_02289	MUY_03341
MUY_02289	MUY_03375
MUY_02289	MUY_03451

MUY_02289	MUY_03775
MUY_02289	MUY_00378
MUY_02289	MUY_04230
MUY_02289	MUY_00526
MUY_02289	MUY_00857
MUY_02295	MUY_02295
MUY_02295	MUY_02578
MUY_02295	MUY_02830
MUY_02295	MUY_03356
MUY_02295	MUY_03860
MUY_02295	MUY_03951
MUY_02295	MUY_04473
MUY_02295	MUY_00912
MUY_02296	MUY_02296
MUY_02296	MUY_00678
MUY_02297	MUY_02297
MUY_02297	MUY_02580
MUY_02297	MUY_02982
MUY_02297	MUY_03140
MUY_02297	MUY_03250
MUY_02297	MUY_03431
MUY_02297	MUY_00323
MUY_02297	MUY_03553
MUY_02297	MUY_03612
MUY_02297	MUY_00445
MUY_02297	MUY_00677
MUY_02297	MUY_00749
MUY_02297	MUY_00779
MUY_02297	MUY_00882
MUY_02300	MUY_02300
MUY_02300	MUY_03044
MUY_02301	MUY_02301
MUY_02301	MUY_03704
MUY_02301	MUY_03831
MUY_02301	MUY_00360
MUY_02301	MUY_00911
MUY_02302	MUY_02302
MUY_02302	MUY_03379
MUY_02303	MUY_02303
MUY_02305	MUY_02305
MUY_02305	MUY_02538
MUY_02305	MUY_02540
MUY_02305	MUY_00258
MUY_02305	MUY_03400
MUY_02305	MUY_00347
MUY_02305	MUY_04132
MUY_02305	MUY_04150

MUY_02305	MUY_04397
MUY_02305	MUY_04438
MUY_02305	MUY_00453
MUY_02305	MUY_00797
MUY_02307	MUY_02307
MUY_02307	MUY_02411
MUY_02307	MUY_02574
MUY_02307	MUY_00250
MUY_02307	MUY_03442
MUY_02307	MUY_04171
MUY_02307	MUY_04318
MUY_02307	MUY_04410
MUY_02307	MUY_00504
MUY_02307	MUY_00782
MUY_02307	MUY_00920
MUY_02309	MUY_02309
MUY_00218	MUY_00218
MUY_02310	MUY_02310
MUY_02310	MUY_02751
MUY_02310	MUY_00784
MUY_02316	MUY_02316
MUY_02316	MUY_02317
MUY_02316	MUY_02639
MUY_02316	MUY_02675
MUY_02316	MUY_02696
MUY_02316	MUY_03135
MUY_02316	MUY_03214
MUY_02316	MUY_03508
MUY_02316	MUY_03543
MUY_02316	MUY_03690
MUY_02316	MUY_04126
MUY_02316	MUY_00478
MUY_02316	MUY_00479
MUY_02317	MUY_02317
MUY_02317	MUY_02725
MUY_02317	MUY_03135
MUY_02317	MUY_03543
MUY_02318	MUY_02318
MUY_02318	MUY_04053
MUY_02318	MUY_04054
MUY_02318	MUY_00071
MUY_02319	MUY_02319
MUY_02320	MUY_02320
MUY_02320	MUY_03064
MUY_02320	MUY_03183
MUY_02320	MUY_03670
MUY_02320	MUY_03686

MUY_02320	MUY_03721
MUY_02320	MUY_03800
MUY_02320	MUY_04382
MUY_02320	MUY_04399
MUY_02320	MUY_00043
MUY_02320	MUY_00706
MUY_02320	MUY_00759
MUY_02320	MUY_00874
MUY_02320	MUY_00916
MUY_02321	MUY_02321
MUY_02321	MUY_02508
MUY_02321	MUY_02515
MUY_02321	MUY_00021
MUY_02321	MUY_03050
MUY_02324	MUY_02324
MUY_02324	MUY_02450
MUY_00015	MUY_00015
MUY_02329	MUY_02329
MUY_02329	MUY_00713
MUY_02331	MUY_02331
MUY_02331	MUY_02615
MUY_02331	MUY_02633
MUY_02331	MUY_02950
MUY_02331	MUY_02958
MUY_02331	MUY_00292
MUY_02331	MUY_03128
MUY_02331	MUY_03225
MUY_02331	MUY_00313
MUY_02331	MUY_03494
MUY_02331	MUY_03606
MUY_02331	MUY_03627
MUY_02331	MUY_03638
MUY_02331	MUY_03964
MUY_02331	MUY_03998
MUY_02331	MUY_04038
MUY_02331	MUY_00486
MUY_02331	MUY_00681
MUY_02331	MUY_00933
MUY_02332	MUY_02332
MUY_02332	MUY_02937
MUY_02332	MUY_02972
MUY_02332	MUY_03839
MUY_02332	MUY_00560
MUY_02332	MUY_00855
MUY_02332	MUY_00904
MUY_02333	MUY_02333
MUY_02333	MUY_02526

MUY_02333	MUY_04163
MUY_02333	MUY_04439
MUY_02333	MUY_00447
MUY_02333	MUY_00856
MUY_02333	MUY_00917
MUY_02333	MUY_00945
MUY_02334	MUY_02334
MUY_02334	MUY_03318
MUY_02334	MUY_03491
MUY_02334	MUY_00854
MUY_02338	MUY_02338
MUY_02338	MUY_02665
MUY_02338	MUY_03532
MUY_02338	MUY_03896
MUY_02339	MUY_02339
MUY_02339	MUY_02984
MUY_02339	MUY_02993
MUY_02339	MUY_00792
MUY_02342	MUY_02342
MUY_02342	MUY_02564
MUY_02342	MUY_02992
MUY_02342	MUY_03267
MUY_02348	MUY_02348
MUY_02348	MUY_03098
MUY_02348	MUY_03283
MUY_02348	MUY_00366
MUY_02348	MUY_04034
MUY_02348	MUY_04086
MUY_02348	MUY_00073
MUY_02348	MUY_00895
MUY_02353	MUY_02353
MUY_02353	MUY_02808
MUY_02353	MUY_02815
MUY_02353	MUY_03543
MUY_02353	MUY_04049
MUY_02353	MUY_00810
MUY_02353	MUY_00869
MUY_00224	MUY_00224
MUY_02355	MUY_02355
MUY_02355	MUY_02618
MUY_02355	MUY_03173
MUY_02355	MUY_03557
MUY_02356	MUY_02356
MUY_02358	MUY_02358
MUY_02358	MUY_03082
MUY_02358	MUY_00398
MUY_02358	MUY_00451

MUY_02358	MUY_00807
MUY_02358	MUY_00809
MUY_02358	MUY_00944
MUY_02361	MUY_02361
MUY_02361	MUY_02588
MUY_02361	MUY_00297
MUY_02361	MUY_03518
MUY_02361	MUY_03667
MUY_02361	MUY_04463
MUY_02361	MUY_00557
MUY_02361	MUY_00583
MUY_02361	MUY_00674
MUY_02362	MUY_02362
MUY_02362	MUY_02363
MUY_02363	MUY_02363
MUY_02363	MUY_02914
MUY_02363	MUY_03136
MUY_02363	MUY_03279
MUY_02363	MUY_03585
MUY_02363	MUY_00523
MUY_02364	MUY_02364
MUY_02364	MUY_02695
MUY_02364	MUY_00272
MUY_02364	MUY_03126
MUY_02364	MUY_03240
MUY_02364	MUY_03659
MUY_02364	MUY_03701
MUY_02364	MUY_03880
MUY_02364	MUY_04014
MUY_02364	MUY_04194
MUY_02364	MUY_00556
MUY_02364	MUY_00710
MUY_02366	MUY_02366
MUY_02366	MUY_02521
MUY_02366	MUY_02523
MUY_02366	MUY_00249
MUY_02366	MUY_02663
MUY_02366	MUY_00263
MUY_02366	MUY_02912
MUY_02366	MUY_02940
MUY_02366	MUY_00288
MUY_02366	MUY_03183
MUY_02366	MUY_03202
MUY_02366	MUY_03252
MUY_02366	MUY_03463
MUY_02366	MUY_03477
MUY_02366	MUY_03483

MUY_02366	MUY_03609
MUY_02366	MUY_03619
MUY_02366	MUY_03648
MUY_02366	MUY_03924
MUY_02366	MUY_04096
MUY_02366	MUY_04146
MUY_02366	MUY_04177
MUY_02366	MUY_04212
MUY_02366	MUY_04293
MUY_02366	MUY_04359
MUY_02366	MUY_04379
MUY_02366	MUY_04418
MUY_02366	MUY_04448
MUY_02366	MUY_04471
MUY_02366	MUY_00418
MUY_02366	MUY_00436
MUY_02366	MUY_00576
MUY_02371	MUY_02371
MUY_02371	MUY_04249
MUY_02371	MUY_00481
MUY_02371	MUY_00573
MUY_00226	MUY_00226
MUY_00226	MUY_00909
MUY_02372	MUY_02372
MUY_02373	MUY_02373
MUY_02373	MUY_03923
MUY_02376	MUY_02376
MUY_02376	MUY_02468
MUY_02376	MUY_02991
MUY_02376	MUY_03263
MUY_02376	MUY_03267
MUY_02376	MUY_03292
MUY_02376	MUY_03434
MUY_02376	MUY_04020
MUY_02376	MUY_04022
MUY_02377	MUY_02377
MUY_02380	MUY_02380
MUY_02380	MUY_04032
MUY_02380	MUY_00971
MUY_02380	MUY_00989
MUY_02382	MUY_02382
MUY_02382	MUY_03538
MUY_02385	MUY_02385
MUY_02386	MUY_02386
MUY_02386	MUY_02646
MUY_02387	MUY_02387
MUY_02387	MUY_03267

MUY_02391	MUY_02391
MUY_02391	MUY_00344
MUY_00228	MUY_00228
MUY_00228	MUY_03779
MUY_00228	MUY_03780
MUY_02392	MUY_02392
MUY_02394	MUY_02394
MUY_02394	MUY_02449
MUY_02394	MUY_02740
MUY_02394	MUY_03248
MUY_02394	MUY_03344
MUY_02394	MUY_03381
MUY_02394	MUY_03823
MUY_02394	MUY_03936
MUY_02394	MUY_03949
MUY_02394	MUY_00849
MUY_02395	MUY_02395
MUY_00229	MUY_00229
MUY_00229	MUY_02521
MUY_00229	MUY_02523
MUY_00229	MUY_00249
MUY_00229	MUY_02663
MUY_00229	MUY_00263
MUY_00229	MUY_02912
MUY_00229	MUY_02940
MUY_00229	MUY_00288
MUY_00229	MUY_03127
MUY_00229	MUY_03183
MUY_00229	MUY_03202
MUY_00229	MUY_03463
MUY_00229	MUY_03477
MUY_00229	MUY_03609
MUY_00229	MUY_03619
MUY_00229	MUY_03648
MUY_00229	MUY_03924
MUY_00229	MUY_04096
MUY_00229	MUY_04146
MUY_00229	MUY_04177
MUY_00229	MUY_04212
MUY_00229	MUY_04293
MUY_00229	MUY_04359
MUY_00229	MUY_00397
MUY_00229	MUY_04418
MUY_00229	MUY_04448
MUY_00229	MUY_04471
MUY_00229	MUY_00418
MUY_00229	MUY_00436

MUY_00229	MUY_00576
MUY_02400	MUY_02400
MUY_02400	MUY_02402
MUY_02402	MUY_02402
MUY_02404	MUY_02404
MUY_02404	MUY_03200
MUY_02407	MUY_02407
MUY_00230	MUY_00230
MUY_00230	MUY_03446
MUY_00230	MUY_03632
MUY_00230	MUY_00344
MUY_00230	MUY_03836
MUY_00230	MUY_00872
MUY_02410	MUY_02410
MUY_02410	MUY_00273
MUY_02410	MUY_03141
MUY_02410	MUY_03241
MUY_02410	MUY_03261
MUY_02410	MUY_03373
MUY_02410	MUY_04036
MUY_02410	MUY_04228
MUY_02411	MUY_02411
MUY_02411	MUY_02574
MUY_02411	MUY_00250
MUY_02411	MUY_03442
MUY_02411	MUY_04171
MUY_02411	MUY_04318
MUY_02411	MUY_04410
MUY_02411	MUY_00504
MUY_02411	MUY_00782
MUY_02411	MUY_00920
MUY_02412	MUY_00979
MUY_02413	MUY_02413
MUY_02413	MUY_03025
MUY_02413	MUY_03345
MUY_02413	MUY_04174
MUY_02413	MUY_00053
MUY_02413	MUY_00057
MUY_02413	MUY_00743
MUY_02413	MUY_00074
MUY_02418	MUY_02418
MUY_02418	MUY_03243
MUY_02419	MUY_02434
MUY_02419	MUY_02534
MUY_02419	MUY_03070
MUY_02419	MUY_03248
MUY_00231	MUY_00231

MUY_00231	MUY_02520
MUY_00231	MUY_00264
MUY_00231	MUY_02939
MUY_00231	MUY_00289
MUY_00231	MUY_03191
MUY_00231	MUY_03425
MUY_00231	MUY_03478
MUY_00231	MUY_03610
MUY_00231	MUY_03620
MUY_00231	MUY_03647
MUY_00231	MUY_04211
MUY_00231	MUY_04294
MUY_00231	MUY_04358
MUY_00231	MUY_00393
MUY_00231	MUY_04417
MUY_00231	MUY_04420
MUY_00231	MUY_04447
MUY_00231	MUY_04470
MUY_00231	MUY_00438
MUY_00231	MUY_00575
MUY_00231	MUY_00780
MUY_02423	MUY_02423
MUY_02424	MUY_02424
MUY_02424	MUY_02510
MUY_02424	MUY_02765
MUY_02424	MUY_02869
MUY_02424	MUY_03012
MUY_02424	MUY_03586
MUY_02424	MUY_03863
MUY_02424	MUY_03904
MUY_02424	MUY_03922
MUY_02424	MUY_04367
MUY_02424	MUY_00496
MUY_02424	MUY_00046
MUY_02424	MUY_00527
MUY_02424	MUY_00816
MUY_02430	MUY_03248
MUY_02434	MUY_02434
MUY_02434	MUY_00251
MUY_02434	MUY_03070
MUY_02434	MUY_03071
MUY_02434	MUY_03248
MUY_02434	MUY_03459
MUY_02434	MUY_04116
MUY_02436	MUY_02436
MUY_02436	MUY_00875
MUY_02436	MUY_00931

MUY_02437	MUY_02437
MUY_02437	MUY_03191
MUY_02437	MUY_00002
MUY_02437	MUY_00609
MUY_02438	MUY_02438
MUY_02438	MUY_03019
MUY_02438	MUY_03267
MUY_02439	MUY_02439
MUY_02439	MUY_02467
MUY_02439	MUY_02627
MUY_02439	MUY_00261
MUY_02439	MUY_03267
MUY_02439	MUY_00308
MUY_02439	MUY_03333
MUY_02439	MUY_03386
MUY_02439	MUY_03449
MUY_02439	MUY_03454
MUY_02439	MUY_03637
MUY_02439	MUY_03810
MUY_02439	MUY_04070
MUY_02439	MUY_04242
MUY_02439	MUY_00382
MUY_02439	MUY_04302
MUY_02439	MUY_04383
MUY_02439	MUY_04433
MUY_02439	MUY_04483
MUY_02439	MUY_00450
MUY_02439	MUY_00464
MUY_02439	MUY_00808
MUY_02439	MUY_00840
MUY_02442	MUY_02442
MUY_02442	MUY_02785
MUY_02442	MUY_03457
MUY_02443	MUY_02443
MUY_02443	MUY_03606
MUY_02444	MUY_02444
MUY_02445	MUY_02445
MUY_02445	MUY_02820
MUY_02445	MUY_00446
MUY_02445	MUY_00998
MUY_02447	MUY_02447
MUY_02447	MUY_02696
MUY_02447	MUY_02788
MUY_02447	MUY_03055
MUY_02447	MUY_04126
MUY_02447	MUY_04239
MUY_02447	MUY_00469

MUY_02447	MUY_00485
MUY_02447	MUY_00696
MUY_02448	MUY_02448
MUY_02448	MUY_02797
MUY_02448	MUY_02829
MUY_02448	MUY_03847
MUY_02448	MUY_04122
MUY_02449	MUY_02449
MUY_02449	MUY_03344
MUY_02449	MUY_03949
MUY_02449	MUY_00849
MUY_02450	MUY_02450
MUY_02451	MUY_02451
MUY_02451	MUY_00746
MUY_02452	MUY_02452
MUY_02453	MUY_02453
MUY_02453	MUY_03846
MUY_02454	MUY_02454
MUY_02454	MUY_02762
MUY_02454	MUY_03902
MUY_02454	MUY_04312
MUY_02454	MUY_00561
MUY_02454	MUY_00734
MUY_02458	MUY_02458
MUY_02458	MUY_02460
MUY_02460	MUY_02460
MUY_02460	MUY_02941
MUY_02460	MUY_03513
MUY_02460	MUY_03515
MUY_02460	MUY_03524
MUY_02460	MUY_04052
MUY_02460	MUY_00460
MUY_02460	MUY_00461
MUY_02463	MUY_02463
MUY_02463	MUY_02736
MUY_02463	MUY_03012
MUY_02463	MUY_03095
MUY_02463	MUY_03863
MUY_02465	MUY_02465
MUY_02465	MUY_03267
MUY_02466	MUY_02466
MUY_02467	MUY_02467
MUY_02467	MUY_02627
MUY_02467	MUY_00261
MUY_02467	MUY_03267
MUY_02467	MUY_00308
MUY_02467	MUY_03333

MUY_02467	MUY_03386
MUY_02467	MUY_03449
MUY_02467	MUY_03454
MUY_02467	MUY_03637
MUY_02467	MUY_03810
MUY_02467	MUY_04070
MUY_02467	MUY_04242
MUY_02467	MUY_00382
MUY_02467	MUY_04302
MUY_02467	MUY_04383
MUY_02467	MUY_04433
MUY_02467	MUY_04483
MUY_02467	MUY_00450
MUY_02467	MUY_00464
MUY_02467	MUY_00808
MUY_02467	MUY_00840
MUY_02468	MUY_02468
MUY_02468	MUY_02469
MUY_02468	MUY_02991
MUY_02468	MUY_03292
MUY_02470	MUY_02470
MUY_02470	MUY_02471
MUY_02471	MUY_02471
MUY_02472	MUY_02472
MUY_02472	MUY_03561
MUY_02472	MUY_03562
MUY_02472	MUY_04373
MUY_02473	MUY_02473
MUY_02473	MUY_03868
MUY_02473	MUY_04037
MUY_02473	MUY_00730
MUY_02473	MUY_00080
MUY_02473	MUY_00081
MUY_02474	MUY_02474
MUY_02475	MUY_02475
MUY_02476	MUY_03267
MUY_02477	MUY_02477
MUY_02477	MUY_04011
MUY_02478	MUY_02478
MUY_02479	MUY_02479
MUY_02479	MUY_02670
MUY_02479	MUY_03480
MUY_02479	MUY_00061
MUY_02480	MUY_02480
MUY_02482	MUY_02482
MUY_02483	MUY_02483
MUY_02484	MUY_02484

MUY_02484	MUY_03267
MUY_02488	MUY_02488
MUY_02488	MUY_03935
MUY_02489	MUY_02489
MUY_02489	MUY_02793
MUY_02489	MUY_03058
MUY_02489	MUY_03089
MUY_02489	MUY_04497
MUY_02489	MUY_04508
MUY_02489	MUY_00804
MUY_02489	MUY_00963
MUY_02495	MUY_02495
MUY_02496	MUY_03267
MUY_02497	MUY_02497
MUY_02497	MUY_00020
MUY_02500	MUY_02500
MUY_02500	MUY_02665
MUY_02500	MUY_03715
MUY_02500	MUY_03896
MUY_02500	MUY_00656
MUY_02502	MUY_02502
MUY_02502	MUY_04290
MUY_02502	MUY_00775
MUY_02503	MUY_02503
MUY_02503	MUY_00803
MUY_02504	MUY_02504
MUY_02504	MUY_02653
MUY_02504	MUY_03267
MUY_02506	MUY_02506
MUY_02506	MUY_03121
MUY_02506	MUY_03418
MUY_02508	MUY_02508
MUY_02508	MUY_02515
MUY_02508	MUY_00021
MUY_02508	MUY_03050
MUY_02510	MUY_02510
MUY_02510	MUY_02765
MUY_02510	MUY_02869
MUY_02510	MUY_03012
MUY_02510	MUY_03586
MUY_02510	MUY_03863
MUY_02510	MUY_03904
MUY_02510	MUY_03922
MUY_02510	MUY_04367
MUY_02510	MUY_00496
MUY_02510	MUY_00046
MUY_02510	MUY_00527

MUY_02510	MUY_00816
MUY_00238	MUY_00238
MUY_00238	MUY_00344
MUY_00238	MUY_00083
MUY_02514	MUY_02514
MUY_02514	MUY_03100
MUY_02514	MUY_03536
MUY_02514	MUY_03833
MUY_02514	MUY_03850
MUY_02514	MUY_04207
MUY_02515	MUY_02515
MUY_02515	MUY_00021
MUY_02515	MUY_03050
MUY_02516	MUY_02516
MUY_02516	MUY_02560
MUY_02516	MUY_02784
MUY_02516	MUY_02845
MUY_02516	MUY_02852
MUY_02516	MUY_02889
MUY_02516	MUY_02892
MUY_02516	MUY_02900
MUY_02516	MUY_03708
MUY_02516	MUY_03918
MUY_02516	MUY_04026
MUY_02516	MUY_04258
MUY_02516	MUY_04322
MUY_02516	MUY_04504
MUY_02516	MUY_00541
MUY_02516	MUY_00571
MUY_02516	MUY_00628
MUY_02517	MUY_02517
MUY_02517	MUY_03927
MUY_02517	MUY_03942
MUY_02517	MUY_03968
MUY_02517	MUY_00492
MUY_02518	MUY_02518
MUY_02518	MUY_04301
MUY_02518	MUY_00698
MUY_02518	MUY_00871
MUY_02520	MUY_02520
MUY_02520	MUY_00264
MUY_02520	MUY_02939
MUY_02520	MUY_00289
MUY_02520	MUY_03478
MUY_02520	MUY_03610
MUY_02520	MUY_03620
MUY_02520	MUY_03647

MUY_02520	MUY_04211
MUY_02520	MUY_04294
MUY_02520	MUY_04358
MUY_02520	MUY_00393
MUY_02520	MUY_04417
MUY_02520	MUY_04420
MUY_02520	MUY_04447
MUY_02520	MUY_04470
MUY_02520	MUY_00438
MUY_02520	MUY_00575
MUY_00239	MUY_00239
MUY_00239	MUY_00309
MUY_02521	MUY_02521
MUY_02521	MUY_02523
MUY_02521	MUY_00249
MUY_02521	MUY_02655
MUY_02521	MUY_02663
MUY_02521	MUY_00263
MUY_02521	MUY_00264
MUY_02521	MUY_02912
MUY_02521	MUY_02939
MUY_02521	MUY_02940
MUY_02521	MUY_02964
MUY_02521	MUY_00288
MUY_02521	MUY_00289
MUY_02521	MUY_03059
MUY_02521	MUY_03117
MUY_02521	MUY_03127
MUY_02521	MUY_03139
MUY_02521	MUY_03183
MUY_02521	MUY_03201
MUY_02521	MUY_03202
MUY_02521	MUY_03252
MUY_02521	MUY_03306
MUY_02521	MUY_03374
MUY_02521	MUY_03463
MUY_02521	MUY_03477
MUY_02521	MUY_03478
MUY_02521	MUY_03483
MUY_02521	MUY_03609
MUY_02521	MUY_03619
MUY_02521	MUY_03647
MUY_02521	MUY_03648
MUY_02521	MUY_03924
MUY_02521	MUY_04096
MUY_02521	MUY_04146
MUY_02521	MUY_04177

MUY_02521	MUY_04211
MUY_02521	MUY_04212
MUY_02521	MUY_04293
MUY_02521	MUY_04294
MUY_02521	MUY_04358
MUY_02521	MUY_04359
MUY_02521	MUY_04379
MUY_02521	MUY_04417
MUY_02521	MUY_04418
MUY_02521	MUY_04447
MUY_02521	MUY_04448
MUY_02521	MUY_04471
MUY_02521	MUY_00418
MUY_02521	MUY_00436
MUY_02521	MUY_00575
MUY_02521	MUY_00576
MUY_02521	MUY_00790
MUY_02521	MUY_00961
MUY_02523	MUY_02523
MUY_02523	MUY_00249
MUY_02523	MUY_02655
MUY_02523	MUY_02663
MUY_02523	MUY_00263
MUY_02523	MUY_00264
MUY_02523	MUY_02912
MUY_02523	MUY_02939
MUY_02523	MUY_02940
MUY_02523	MUY_02964
MUY_02523	MUY_00288
MUY_02523	MUY_00289
MUY_02523	MUY_03059
MUY_02523	MUY_03117
MUY_02523	MUY_03127
MUY_02523	MUY_03139
MUY_02523	MUY_03183
MUY_02523	MUY_03201
MUY_02523	MUY_03202
MUY_02523	MUY_03252
MUY_02523	MUY_03306
MUY_02523	MUY_03374
MUY_02523	MUY_03463
MUY_02523	MUY_03477
MUY_02523	MUY_03478
MUY_02523	MUY_03483
MUY_02523	MUY_03609
MUY_02523	MUY_03619
MUY_02523	MUY_03647

MUY_02523	MUY_03648
MUY_02523	MUY_03924
MUY_02523	MUY_04096
MUY_02523	MUY_04146
MUY_02523	MUY_04177
MUY_02523	MUY_04211
MUY_02523	MUY_04212
MUY_02523	MUY_04293
MUY_02523	MUY_04294
MUY_02523	MUY_04358
MUY_02523	MUY_04359
MUY_02523	MUY_04379
MUY_02523	MUY_04417
MUY_02523	MUY_04418
MUY_02523	MUY_04447
MUY_02523	MUY_04448
MUY_02523	MUY_04471
MUY_02523	MUY_00418
MUY_02523	MUY_00436
MUY_02523	MUY_00575
MUY_02523	MUY_00576
MUY_02523	MUY_00790
MUY_02523	MUY_00961
MUY_02525	MUY_03496
MUY_02525	MUY_00780
MUY_02526	MUY_02526
MUY_02526	MUY_02896
MUY_02526	MUY_04163
MUY_02526	MUY_04439
MUY_02526	MUY_04501
MUY_02526	MUY_04505
MUY_02526	MUY_00447
MUY_02526	MUY_00856
MUY_02526	MUY_00917
MUY_02526	MUY_00945
MUY_00240	MUY_00240
MUY_00240	MUY_03659
MUY_00240	MUY_04156
MUY_00240	MUY_04194
MUY_00240	MUY_04345
MUY_00240	MUY_04361
MUY_00240	MUY_04485
MUY_00240	MUY_00787
MUY_00240	MUY_00838
MUY_00240	MUY_00886
MUY_02528	MUY_02528
MUY_02528	MUY_02668

MUY_02528	MUY_03072
MUY_02528	MUY_03253
MUY_02528	MUY_03299
MUY_02528	MUY_00065
MUY_02530	MUY_02530
MUY_02531	MUY_02531
MUY_02531	MUY_02563
MUY_02531	MUY_00253
MUY_02531	MUY_03605
MUY_02533	MUY_02533
MUY_02533	MUY_02534
MUY_02534	MUY_02534
MUY_02534	MUY_03240
MUY_02534	MUY_03863
MUY_02534	MUY_03925
MUY_02534	MUY_00043
MUY_02537	MUY_02537
MUY_02537	MUY_02576
MUY_02538	MUY_02538
MUY_02538	MUY_02541
MUY_02538	MUY_03004
MUY_02538	MUY_03062
MUY_02539	MUY_02539
MUY_02539	MUY_03267
MUY_02540	MUY_02540
MUY_02540	MUY_02541
MUY_02541	MUY_02541
MUY_02541	MUY_02667
MUY_02541	MUY_02794
MUY_02541	MUY_03062
MUY_02541	MUY_00023
MUY_02541	MUY_00344
MUY_02544	MUY_02544
MUY_02545	MUY_02545
MUY_02547	MUY_03477
MUY_02547	MUY_04096
MUY_00241	MUY_00241
MUY_00241	MUY_02947
MUY_00241	MUY_00277
MUY_00241	MUY_00337
MUY_00241	MUY_04086
MUY_00241	MUY_00433
MUY_00241	MUY_00813
MUY_02548	MUY_02548
MUY_02548	MUY_00857
MUY_02550	MUY_02550
MUY_02550	MUY_02614

MUY_02551	MUY_02551
MUY_02556	MUY_02556
MUY_00242	MUY_00242
MUY_00242	MUY_03031
MUY_00242	MUY_03440
MUY_02559	MUY_02559
MUY_02560	MUY_02560
MUY_02560	MUY_02561
MUY_02560	MUY_02784
MUY_02560	MUY_02845
MUY_02560	MUY_02852
MUY_02560	MUY_02889
MUY_02560	MUY_02892
MUY_02560	MUY_02900
MUY_02560	MUY_03708
MUY_02560	MUY_03918
MUY_02560	MUY_04026
MUY_02560	MUY_04258
MUY_02560	MUY_04322
MUY_02560	MUY_04504
MUY_02560	MUY_00541
MUY_02560	MUY_00571
MUY_02560	MUY_00628
MUY_02561	MUY_02561
MUY_02561	MUY_02562
MUY_02561	MUY_02722
MUY_02561	MUY_03848
MUY_02561	MUY_04492
MUY_02561	MUY_00535
MUY_02561	MUY_00536
MUY_02561	MUY_00537
MUY_02561	MUY_00539
MUY_02561	MUY_00540
MUY_02561	MUY_00542
MUY_02561	MUY_00070
MUY_02562	MUY_02562
MUY_02562	MUY_02722
MUY_02562	MUY_03848
MUY_02562	MUY_00378
MUY_02562	MUY_04230
MUY_02562	MUY_04492
MUY_02562	MUY_00535
MUY_02562	MUY_00536
MUY_02562	MUY_00537
MUY_02562	MUY_00539
MUY_02562	MUY_00540
MUY_02563	MUY_02563

MUY_02563	MUY_00253
MUY_02563	MUY_03605
MUY_02564	MUY_02564
MUY_02564	MUY_02992
MUY_02564	MUY_00344
MUY_02565	MUY_02565
MUY_02565	MUY_00767
MUY_00243	MUY_00243
MUY_00243	MUY_03342
MUY_00243	MUY_03382
MUY_00243	MUY_03383
MUY_00243	MUY_03945
MUY_00243	MUY_00056
MUY_02566	MUY_02566
MUY_02568	MUY_02568
MUY_02568	MUY_02758
MUY_02568	MUY_04263
MUY_02568	MUY_00946
MUY_02571	MUY_03683
MUY_02571	MUY_00483
MUY_02574	MUY_02574
MUY_02574	MUY_00250
MUY_02574	MUY_03442
MUY_02574	MUY_04171
MUY_02574	MUY_04318
MUY_02574	MUY_04410
MUY_02574	MUY_00504
MUY_02574	MUY_00782
MUY_02574	MUY_00920
MUY_02576	MUY_02576
MUY_02578	MUY_02578
MUY_02578	MUY_02830
MUY_02578	MUY_03356
MUY_02578	MUY_03860
MUY_02578	MUY_03951
MUY_02578	MUY_04473
MUY_02578	MUY_00912
MUY_02580	MUY_02580
MUY_02580	MUY_02982
MUY_02580	MUY_03140
MUY_02580	MUY_03250
MUY_02580	MUY_03431
MUY_02580	MUY_00323
MUY_02580	MUY_03553
MUY_02580	MUY_03612
MUY_02580	MUY_00445
MUY_02580	MUY_00677

MUY_02580	MUY_00749
MUY_02580	MUY_00779
MUY_02580	MUY_00882
MUY_02582	MUY_02582
MUY_02582	MUY_02970
MUY_02582	MUY_03658
MUY_02582	MUY_04399
MUY_02586	MUY_02586
MUY_02586	MUY_03519
MUY_02587	MUY_02587
MUY_02588	MUY_02588
MUY_02588	MUY_00297
MUY_02588	MUY_03518
MUY_02588	MUY_03667
MUY_02588	MUY_04463
MUY_02588	MUY_00557
MUY_02588	MUY_00583
MUY_02588	MUY_00674
MUY_02589	MUY_02589
MUY_02597	MUY_02597
MUY_02597	MUY_02598
MUY_02597	MUY_02609
MUY_02597	MUY_02643
MUY_02597	MUY_02700
MUY_02597	MUY_02744
MUY_02597	MUY_02745
MUY_02597	MUY_02760
MUY_02597	MUY_00265
MUY_02597	MUY_02894
MUY_02597	MUY_02929
MUY_02597	MUY_02945
MUY_02597	MUY_02946
MUY_02597	MUY_00285
MUY_02597	MUY_03154
MUY_02597	MUY_03330
MUY_02597	MUY_03332
MUY_02597	MUY_03354
MUY_02597	MUY_00322
MUY_02597	MUY_03575
MUY_02597	MUY_03576
MUY_02597	MUY_03582
MUY_02597	MUY_03598
MUY_02597	MUY_03634
MUY_02597	MUY_03649
MUY_02597	MUY_03676
MUY_02597	MUY_03785
MUY_02597	MUY_03805

MUY_02597	MUY_03900
MUY_02597	MUY_03946
MUY_02597	MUY_00372
MUY_02597	MUY_03978
MUY_02597	MUY_04144
MUY_02597	MUY_04172
MUY_02597	MUY_04248
MUY_02597	MUY_04254
MUY_02597	MUY_04266
MUY_02597	MUY_04269
MUY_02597	MUY_04282
MUY_02597	MUY_04296
MUY_02597	MUY_00388
MUY_02597	MUY_04348
MUY_02597	MUY_04357
MUY_02597	MUY_00394
MUY_02597	MUY_04403
MUY_02597	MUY_04416
MUY_02597	MUY_04445
MUY_02597	MUY_04453
MUY_02597	MUY_00420
MUY_02597	MUY_00434
MUY_02597	MUY_00442
MUY_02597	MUY_00516
MUY_02597	MUY_00585
MUY_02597	MUY_00800
MUY_02597	MUY_00815
MUY_02597	MUY_00937
MUY_02597	MUY_00938
MUY_02597	MUY_00939
MUY_02597	MUY_00968
MUY_02597	MUY_00975
MUY_02597	MUY_00996
MUY_02597	MUY_01000
MUY_02598	MUY_02598
MUY_02598	MUY_02609
MUY_02598	MUY_02643
MUY_02598	MUY_02700
MUY_02598	MUY_02744
MUY_02598	MUY_02745
MUY_02598	MUY_02760
MUY_02598	MUY_00265
MUY_02598	MUY_02894
MUY_02598	MUY_02929
MUY_02598	MUY_02945
MUY_02598	MUY_02946
MUY_02598	MUY_00285

MUY_02598	MUY_03154
MUY_02598	MUY_03330
MUY_02598	MUY_03332
MUY_02598	MUY_03354
MUY_02598	MUY_00322
MUY_02598	MUY_03575
MUY_02598	MUY_03576
MUY_02598	MUY_03582
MUY_02598	MUY_03598
MUY_02598	MUY_03634
MUY_02598	MUY_03649
MUY_02598	MUY_03676
MUY_02598	MUY_03785
MUY_02598	MUY_03805
MUY_02598	MUY_03900
MUY_02598	MUY_03946
MUY_02598	MUY_00372
MUY_02598	MUY_03978
MUY_02598	MUY_04144
MUY_02598	MUY_04172
MUY_02598	MUY_04248
MUY_02598	MUY_04254
MUY_02598	MUY_04266
MUY_02598	MUY_04269
MUY_02598	MUY_04282
MUY_02598	MUY_04296
MUY_02598	MUY_00388
MUY_02598	MUY_04348
MUY_02598	MUY_04357
MUY_02598	MUY_00394
MUY_02598	MUY_04403
MUY_02598	MUY_04416
MUY_02598	MUY_04445
MUY_02598	MUY_04453
MUY_02598	MUY_00420
MUY_02598	MUY_00434
MUY_02598	MUY_00442
MUY_02598	MUY_00516
MUY_02598	MUY_00585
MUY_02598	MUY_00800
MUY_02598	MUY_00815
MUY_02598	MUY_00937
MUY_02598	MUY_00938
MUY_02598	MUY_00939
MUY_02598	MUY_00968
MUY_02598	MUY_00975
MUY_02598	MUY_00996

MUY_02598	MUY_01000
MUY_02602	MUY_02602
MUY_02602	MUY_02635
MUY_02602	MUY_03200
MUY_02606	MUY_02606
MUY_02606	MUY_04085
MUY_02606	MUY_04206
MUY_02606	MUY_04410
MUY_02606	MUY_00695
MUY_02606	MUY_00906
MUY_02609	MUY_02609
MUY_02609	MUY_02643
MUY_02609	MUY_02700
MUY_02609	MUY_02744
MUY_02609	MUY_02745
MUY_02609	MUY_02760
MUY_02609	MUY_00265
MUY_02609	MUY_02894
MUY_02609	MUY_02929
MUY_02609	MUY_02945
MUY_02609	MUY_02946
MUY_02609	MUY_00285
MUY_02609	MUY_03154
MUY_02609	MUY_03330
MUY_02609	MUY_03332
MUY_02609	MUY_03354
MUY_02609	MUY_00322
MUY_02609	MUY_03575
MUY_02609	MUY_03576
MUY_02609	MUY_03582
MUY_02609	MUY_03598
MUY_02609	MUY_03634
MUY_02609	MUY_03649
MUY_02609	MUY_03676
MUY_02609	MUY_03785
MUY_02609	MUY_03805
MUY_02609	MUY_03900
MUY_02609	MUY_03946
MUY_02609	MUY_00372
MUY_02609	MUY_03978
MUY_02609	MUY_04144
MUY_02609	MUY_04172
MUY_02609	MUY_04248
MUY_02609	MUY_04254
MUY_02609	MUY_04266
MUY_02609	MUY_04269
MUY_02609	MUY_04282

MUY_02609	MUY_04296
MUY_02609	MUY_00388
MUY_02609	MUY_04348
MUY_02609	MUY_04357
MUY_02609	MUY_00394
MUY_02609	MUY_04403
MUY_02609	MUY_04416
MUY_02609	MUY_04445
MUY_02609	MUY_04453
MUY_02609	MUY_00420
MUY_02609	MUY_00434
MUY_02609	MUY_00442
MUY_02609	MUY_00516
MUY_02609	MUY_00585
MUY_02609	MUY_00800
MUY_02609	MUY_00815
MUY_02609	MUY_00937
MUY_02609	MUY_00938
MUY_02609	MUY_00939
MUY_02609	MUY_00968
MUY_02609	MUY_00975
MUY_02609	MUY_00996
MUY_02609	MUY_01000
MUY_00247	MUY_00247
MUY_02614	MUY_02614
MUY_02615	MUY_02615
MUY_02615	MUY_02633
MUY_02615	MUY_02950
MUY_02615	MUY_02958
MUY_02615	MUY_00292
MUY_02615	MUY_03128
MUY_02615	MUY_03225
MUY_02615	MUY_00313
MUY_02615	MUY_03494
MUY_02615	MUY_03606
MUY_02615	MUY_03627
MUY_02615	MUY_03638
MUY_02615	MUY_00344
MUY_02615	MUY_03964
MUY_02615	MUY_03998
MUY_02615	MUY_04038
MUY_02615	MUY_04045
MUY_02615	MUY_00479
MUY_02615	MUY_00486
MUY_02615	MUY_00681
MUY_02615	MUY_00721
MUY_02615	MUY_00933

MUY_02615	MUY_00979
MUY_02616	MUY_02616
MUY_02616	MUY_02697
MUY_02616	MUY_02725
MUY_02616	MUY_02910
MUY_02616	MUY_02917
MUY_02616	MUY_03284
MUY_02616	MUY_03304
MUY_02616	MUY_00316
MUY_02616	MUY_03706
MUY_02616	MUY_04467
MUY_02616	MUY_00824
MUY_02617	MUY_02617
MUY_02617	MUY_02826
MUY_02618	MUY_02618
MUY_02618	MUY_03173
MUY_02618	MUY_03557
MUY_02619	MUY_02619
MUY_02619	MUY_02941
MUY_02619	MUY_03513
MUY_02619	MUY_03515
MUY_02619	MUY_03524
MUY_02619	MUY_03567
MUY_02619	MUY_03840
MUY_02619	MUY_00461
MUY_02620	MUY_02620
MUY_02620	MUY_03341
MUY_02620	MUY_03375
MUY_02620	MUY_03451
MUY_02620	MUY_03775
MUY_02620	MUY_00378
MUY_02620	MUY_04230
MUY_02620	MUY_00526
MUY_02620	MUY_00857
MUY_02622	MUY_02622
MUY_02622	MUY_03257
MUY_02623	MUY_02623
MUY_02623	MUY_03267
MUY_02626	MUY_02626
MUY_02626	MUY_02645
MUY_02627	MUY_02627
MUY_02627	MUY_00261
MUY_02627	MUY_00308
MUY_02627	MUY_03333
MUY_02627	MUY_03386
MUY_02627	MUY_03449
MUY_02627	MUY_03454

MUY_02627	MUY_03637
MUY_02627	MUY_03810
MUY_02627	MUY_04070
MUY_02627	MUY_04242
MUY_02627	MUY_00382
MUY_02627	MUY_04302
MUY_02627	MUY_04383
MUY_02627	MUY_04433
MUY_02627	MUY_04483
MUY_02627	MUY_00450
MUY_02627	MUY_00464
MUY_02627	MUY_00808
MUY_02627	MUY_00840
MUY_00017	MUY_00017
MUY_02631	MUY_03683
MUY_02631	MUY_00483
MUY_02632	MUY_04482
MUY_02632	MUY_00583
MUY_02633	MUY_02633
MUY_02633	MUY_02950
MUY_02633	MUY_02958
MUY_02633	MUY_00292
MUY_02633	MUY_03128
MUY_02633	MUY_03225
MUY_02633	MUY_00313
MUY_02633	MUY_03494
MUY_02633	MUY_03606
MUY_02633	MUY_03627
MUY_02633	MUY_03638
MUY_02633	MUY_03964
MUY_02633	MUY_03998
MUY_02633	MUY_04038
MUY_02633	MUY_04045
MUY_02633	MUY_00486
MUY_02633	MUY_00681
MUY_02633	MUY_00721
MUY_02633	MUY_00933
MUY_02634	MUY_02634
MUY_02634	MUY_03267
MUY_02634	MUY_04436
MUY_02635	MUY_02635
MUY_02635	MUY_03200
MUY_02637	MUY_02637
MUY_02637	MUY_02642
MUY_02637	MUY_02680
MUY_02637	MUY_04060
MUY_02637	MUY_04061

MUY_02637	MUY_04062
MUY_02637	MUY_04063
MUY_02637	MUY_04064
MUY_02637	MUY_04065
MUY_02637	MUY_04066
MUY_02637	MUY_04067
MUY_02638	MUY_02638
MUY_02638	MUY_03281
MUY_00249	MUY_00249
MUY_00249	MUY_02655
MUY_00249	MUY_02663
MUY_00249	MUY_00263
MUY_00249	MUY_00264
MUY_00249	MUY_02912
MUY_00249	MUY_02939
MUY_00249	MUY_02940
MUY_00249	MUY_02964
MUY_00249	MUY_00288
MUY_00249	MUY_00289
MUY_00249	MUY_03059
MUY_00249	MUY_03117
MUY_00249	MUY_03127
MUY_00249	MUY_03139
MUY_00249	MUY_03183
MUY_00249	MUY_03201
MUY_00249	MUY_03202
MUY_00249	MUY_03252
MUY_00249	MUY_03306
MUY_00249	MUY_03374
MUY_00249	MUY_03463
MUY_00249	MUY_03477
MUY_00249	MUY_03478
MUY_00249	MUY_03483
MUY_00249	MUY_03609
MUY_00249	MUY_03619
MUY_00249	MUY_03647
MUY_00249	MUY_03648
MUY_00249	MUY_03924
MUY_00249	MUY_04096
MUY_00249	MUY_04146
MUY_00249	MUY_04177
MUY_00249	MUY_04211
MUY_00249	MUY_04212
MUY_00249	MUY_04293
MUY_00249	MUY_04294
MUY_00249	MUY_04358
MUY_00249	MUY_04359

MUY_00249	MUY_04379
MUY_00249	MUY_04417
MUY_00249	MUY_04418
MUY_00249	MUY_04447
MUY_00249	MUY_04448
MUY_00249	MUY_04471
MUY_00249	MUY_00418
MUY_00249	MUY_00436
MUY_00249	MUY_00575
MUY_00249	MUY_00576
MUY_00249	MUY_00790
MUY_00249	MUY_00961
MUY_02639	MUY_02639
MUY_02639	MUY_03213
MUY_02639	MUY_03214
MUY_02642	MUY_04510
MUY_02643	MUY_02643
MUY_02643	MUY_02700
MUY_02643	MUY_02744
MUY_02643	MUY_02745
MUY_02643	MUY_02760
MUY_02643	MUY_00265
MUY_02643	MUY_02894
MUY_02643	MUY_02929
MUY_02643	MUY_02945
MUY_02643	MUY_02946
MUY_02643	MUY_00285
MUY_02643	MUY_03154
MUY_02643	MUY_03330
MUY_02643	MUY_03332
MUY_02643	MUY_03354
MUY_02643	MUY_00322
MUY_02643	MUY_03575
MUY_02643	MUY_03576
MUY_02643	MUY_03582
MUY_02643	MUY_03598
MUY_02643	MUY_03634
MUY_02643	MUY_03649
MUY_02643	MUY_03676
MUY_02643	MUY_03785
MUY_02643	MUY_03805
MUY_02643	MUY_03900
MUY_02643	MUY_03946
MUY_02643	MUY_00372
MUY_02643	MUY_03978
MUY_02643	MUY_04144
MUY_02643	MUY_04172

MUY_02643	MUY_04248
MUY_02643	MUY_04254
MUY_02643	MUY_04266
MUY_02643	MUY_04269
MUY_02643	MUY_04282
MUY_02643	MUY_04296
MUY_02643	MUY_00388
MUY_02643	MUY_04348
MUY_02643	MUY_04357
MUY_02643	MUY_00394
MUY_02643	MUY_04403
MUY_02643	MUY_04416
MUY_02643	MUY_04445
MUY_02643	MUY_04453
MUY_02643	MUY_00420
MUY_02643	MUY_00434
MUY_02643	MUY_00442
MUY_02643	MUY_00516
MUY_02643	MUY_00585
MUY_02643	MUY_00800
MUY_02643	MUY_00074
MUY_02643	MUY_00815
MUY_02643	MUY_00937
MUY_02643	MUY_00938
MUY_02643	MUY_00939
MUY_02643	MUY_00968
MUY_02643	MUY_00975
MUY_02643	MUY_00996
MUY_02643	MUY_01000
MUY_02645	MUY_02645
MUY_02645	MUY_03034
MUY_02645	MUY_00772
MUY_02646	MUY_02646
MUY_02647	MUY_02647
MUY_02647	MUY_03289
MUY_02647	MUY_00784
MUY_02648	MUY_02648
MUY_02648	MUY_02941
MUY_02648	MUY_03513
MUY_02648	MUY_03515
MUY_02648	MUY_03524
MUY_02648	MUY_00461
MUY_02648	MUY_00895
MUY_02649	MUY_02649
MUY_02649	MUY_02650
MUY_02649	MUY_02669
MUY_02649	MUY_02785

MUY_02649	MUY_04476
MUY_02649	MUY_00472
MUY_02649	MUY_00473
MUY_02649	MUY_00893
MUY_02649	MUY_00894
MUY_00250	MUY_00250
MUY_00250	MUY_03442
MUY_00250	MUY_04171
MUY_00250	MUY_04318
MUY_00250	MUY_04410
MUY_00250	MUY_00504
MUY_00250	MUY_00782
MUY_00250	MUY_00920
MUY_02650	MUY_02650
MUY_02650	MUY_00473
MUY_02650	MUY_00893
MUY_02650	MUY_00894
MUY_02651	MUY_02651
MUY_02651	MUY_02841
MUY_02651	MUY_03862
MUY_02652	MUY_02652
MUY_02652	MUY_03230
MUY_02653	MUY_02653
MUY_02653	MUY_03267
MUY_02654	MUY_02654
MUY_02654	MUY_04136
MUY_02655	MUY_02655
MUY_02655	MUY_02663
MUY_02655	MUY_00263
MUY_02655	MUY_00264
MUY_02655	MUY_02912
MUY_02655	MUY_02940
MUY_02655	MUY_00288
MUY_02655	MUY_00289
MUY_02655	MUY_03183
MUY_02655	MUY_03201
MUY_02655	MUY_03202
MUY_02655	MUY_03463
MUY_02655	MUY_03477
MUY_02655	MUY_03609
MUY_02655	MUY_03619
MUY_02655	MUY_03647
MUY_02655	MUY_03648
MUY_02655	MUY_03924
MUY_02655	MUY_04096
MUY_02655	MUY_04146
MUY_02655	MUY_04177

MUY_02655	MUY_04212
MUY_02655	MUY_04293
MUY_02655	MUY_04359
MUY_02655	MUY_04417
MUY_02655	MUY_04418
MUY_02655	MUY_04448
MUY_02655	MUY_04471
MUY_02655	MUY_00418
MUY_02655	MUY_00436
MUY_02655	MUY_00575
MUY_02655	MUY_00576
MUY_00251	MUY_00251
MUY_00251	MUY_02996
MUY_00251	MUY_03459
MUY_00251	MUY_04116
MUY_02658	MUY_02658
MUY_02658	MUY_04307
MUY_02658	MUY_04400
MUY_02659	MUY_02659
MUY_02663	MUY_02663
MUY_02663	MUY_00263
MUY_02663	MUY_00264
MUY_02663	MUY_02912
MUY_02663	MUY_02939
MUY_02663	MUY_02940
MUY_02663	MUY_02964
MUY_02663	MUY_00288
MUY_02663	MUY_00289
MUY_02663	MUY_03059
MUY_02663	MUY_03117
MUY_02663	MUY_03127
MUY_02663	MUY_03139
MUY_02663	MUY_03183
MUY_02663	MUY_03201
MUY_02663	MUY_03202
MUY_02663	MUY_03252
MUY_02663	MUY_03306
MUY_02663	MUY_03374
MUY_02663	MUY_03463
MUY_02663	MUY_03477
MUY_02663	MUY_03478
MUY_02663	MUY_03483
MUY_02663	MUY_03609
MUY_02663	MUY_03619
MUY_02663	MUY_03647
MUY_02663	MUY_03648
MUY_02663	MUY_03924

MUY_02663	MUY_04096
MUY_02663	MUY_04146
MUY_02663	MUY_04177
MUY_02663	MUY_04211
MUY_02663	MUY_04212
MUY_02663	MUY_04293
MUY_02663	MUY_04294
MUY_02663	MUY_04358
MUY_02663	MUY_04359
MUY_02663	MUY_04379
MUY_02663	MUY_04417
MUY_02663	MUY_04418
MUY_02663	MUY_04447
MUY_02663	MUY_04448
MUY_02663	MUY_04471
MUY_02663	MUY_00418
MUY_02663	MUY_00436
MUY_02663	MUY_00575
MUY_02663	MUY_00576
MUY_02663	MUY_00790
MUY_02663	MUY_00961
MUY_02665	MUY_02665
MUY_02665	MUY_00312
MUY_02665	MUY_03608
MUY_02665	MUY_03715
MUY_02665	MUY_03896
MUY_02665	MUY_04466
MUY_02666	MUY_02666
MUY_02666	MUY_00998
MUY_02667	MUY_02667
MUY_02667	MUY_04313
MUY_02668	MUY_02668
MUY_02668	MUY_03253
MUY_02668	MUY_03299
MUY_02668	MUY_00065
MUY_02669	MUY_02669
MUY_02669	MUY_00473
MUY_02669	MUY_00894
MUY_02670	MUY_02670
MUY_02670	MUY_03480
MUY_02671	MUY_02671
MUY_02671	MUY_02788
MUY_02671	MUY_02798
MUY_02671	MUY_02814
MUY_02671	MUY_03024
MUY_02671	MUY_03190
MUY_02671	MUY_03227

MUY_02671	MUY_03248
MUY_02671	MUY_03425
MUY_02671	MUY_00367
MUY_02671	MUY_03925
MUY_02671	MUY_04086
MUY_02671	MUY_04149
MUY_02671	MUY_04334
MUY_02671	MUY_04476
MUY_02671	MUY_04495
MUY_02671	MUY_00002
MUY_02671	MUY_00536
MUY_02671	MUY_00540
MUY_02671	MUY_00008
MUY_02671	MUY_00984
MUY_02672	MUY_02672
MUY_02672	MUY_03267
MUY_02673	MUY_02673
MUY_00252	MUY_00252
MUY_02675	MUY_02675
MUY_02675	MUY_03267
MUY_02677	MUY_02677
MUY_02677	MUY_04027
MUY_02680	MUY_04510
MUY_02687	MUY_02687
MUY_00253	MUY_00253
MUY_00253	MUY_03605
MUY_02688	MUY_02688
MUY_02688	MUY_00833
MUY_02689	MUY_02689
MUY_02695	MUY_02695
MUY_02695	MUY_02820
MUY_02695	MUY_00272
MUY_02695	MUY_03126
MUY_02695	MUY_03240
MUY_02695	MUY_03659
MUY_02695	MUY_03701
MUY_02695	MUY_03880
MUY_02695	MUY_04014
MUY_02695	MUY_04194
MUY_02695	MUY_00556
MUY_02695	MUY_00710
MUY_02695	MUY_01000
MUY_02696	MUY_02696
MUY_02696	MUY_02788
MUY_02696	MUY_03055
MUY_02696	MUY_04126
MUY_02696	MUY_04239

MUY_02696	MUY_00469
MUY_02696	MUY_00485
MUY_02696	MUY_00696
MUY_02697	MUY_02697
MUY_02697	MUY_02725
MUY_02697	MUY_02910
MUY_02697	MUY_02917
MUY_02697	MUY_02941
MUY_02697	MUY_03041
MUY_02697	MUY_03284
MUY_02697	MUY_03304
MUY_02697	MUY_00316
MUY_02697	MUY_03513
MUY_02697	MUY_03515
MUY_02697	MUY_03524
MUY_02697	MUY_03706
MUY_02697	MUY_00366
MUY_02697	MUY_04467
MUY_02697	MUY_00461
MUY_02697	MUY_00072
MUY_02697	MUY_00824
MUY_02698	MUY_02698
MUY_02698	MUY_03783
MUY_02700	MUY_02700
MUY_02700	MUY_02744
MUY_02700	MUY_02745
MUY_02700	MUY_02760
MUY_02700	MUY_00265
MUY_02700	MUY_02894
MUY_02700	MUY_02929
MUY_02700	MUY_02945
MUY_02700	MUY_02946
MUY_02700	MUY_00285
MUY_02700	MUY_03154
MUY_02700	MUY_03330
MUY_02700	MUY_03332
MUY_02700	MUY_03354
MUY_02700	MUY_00322
MUY_02700	MUY_03575
MUY_02700	MUY_03576
MUY_02700	MUY_03582
MUY_02700	MUY_03598
MUY_02700	MUY_03634
MUY_02700	MUY_03649
MUY_02700	MUY_03676
MUY_02700	MUY_03785
MUY_02700	MUY_03805

MUY_02700	MUY_03900
MUY_02700	MUY_03946
MUY_02700	MUY_00372
MUY_02700	MUY_03978
MUY_02700	MUY_04144
MUY_02700	MUY_04172
MUY_02700	MUY_04248
MUY_02700	MUY_04254
MUY_02700	MUY_04266
MUY_02700	MUY_04269
MUY_02700	MUY_04282
MUY_02700	MUY_04296
MUY_02700	MUY_00388
MUY_02700	MUY_04348
MUY_02700	MUY_04357
MUY_02700	MUY_00394
MUY_02700	MUY_04403
MUY_02700	MUY_04416
MUY_02700	MUY_04445
MUY_02700	MUY_04453
MUY_02700	MUY_00420
MUY_02700	MUY_00434
MUY_02700	MUY_00442
MUY_02700	MUY_00516
MUY_02700	MUY_00585
MUY_02700	MUY_00800
MUY_02700	MUY_00815
MUY_02700	MUY_00937
MUY_02700	MUY_00938
MUY_02700	MUY_00939
MUY_02700	MUY_00968
MUY_02700	MUY_00975
MUY_02700	MUY_00996
MUY_02700	MUY_01000
MUY_02701	MUY_02701
MUY_02701	MUY_02702
MUY_02702	MUY_02702
MUY_00254	MUY_00254
MUY_00254	MUY_03182
MUY_02703	MUY_02703
MUY_02705	MUY_02705
MUY_02705	MUY_03891
MUY_02707	MUY_02707
MUY_02707	MUY_02708
MUY_02707	MUY_02882
MUY_02707	MUY_02883
MUY_02707	MUY_03769

MUY_02707	MUY_03778
MUY_02707	MUY_03830
MUY_02707	MUY_04185
MUY_02707	MUY_04271
MUY_02707	MUY_04291
MUY_02707	MUY_00551
MUY_02707	MUY_00599
MUY_02707	MUY_00602
MUY_02707	MUY_00691
MUY_02707	MUY_00795
MUY_02707	MUY_00086
MUY_02707	MUY_00973
MUY_02708	MUY_02708
MUY_02708	MUY_02882
MUY_02708	MUY_02883
MUY_02708	MUY_02889
MUY_02708	MUY_02892
MUY_02708	MUY_02900
MUY_02708	MUY_03708
MUY_02708	MUY_03769
MUY_02708	MUY_03778
MUY_02708	MUY_03830
MUY_02708	MUY_04185
MUY_02708	MUY_04271
MUY_02708	MUY_04291
MUY_02708	MUY_04501
MUY_02708	MUY_04505
MUY_02708	MUY_00551
MUY_02708	MUY_00599
MUY_02708	MUY_00602
MUY_02708	MUY_00628
MUY_02708	MUY_00691
MUY_02708	MUY_00795
MUY_02708	MUY_00086
MUY_02708	MUY_00973
MUY_02710	MUY_02710
MUY_02711	MUY_02711
MUY_02711	MUY_00719
MUY_02720	MUY_02720
MUY_02721	MUY_02721
MUY_02721	MUY_02874
MUY_02722	MUY_02722
MUY_02722	MUY_03848
MUY_02722	MUY_00378
MUY_02722	MUY_04230
MUY_02722	MUY_04492
MUY_02722	MUY_00535

MUY_02722	MUY_00536
MUY_02722	MUY_00537
MUY_02722	MUY_00539
MUY_02722	MUY_00540
MUY_02723	MUY_02723
MUY_02723	MUY_03589
MUY_02725	MUY_02725
MUY_02725	MUY_02910
MUY_02725	MUY_02917
MUY_02725	MUY_03284
MUY_02725	MUY_03304
MUY_02725	MUY_00316
MUY_02725	MUY_03706
MUY_02725	MUY_04467
MUY_02725	MUY_00824
MUY_02727	MUY_02727
MUY_00255	MUY_00255
MUY_00255	MUY_02780
MUY_00255	MUY_03296
MUY_00255	MUY_03314
MUY_00255	MUY_03967
MUY_00255	MUY_04086
MUY_00255	MUY_00435
MUY_00255	MUY_00514
MUY_00255	MUY_00589
MUY_00255	MUY_00741
MUY_00255	MUY_00780
MUY_02729	MUY_02729
MUY_02729	MUY_03608
MUY_02729	MUY_00825
MUY_02730	MUY_02730
MUY_02730	MUY_02977
MUY_02730	MUY_03601
MUY_02730	MUY_03792
MUY_02730	MUY_04042
MUY_02730	MUY_04408
MUY_02730	MUY_00443
MUY_00256	MUY_02780
MUY_00256	MUY_03211
MUY_00256	MUY_03296
MUY_00256	MUY_03789
MUY_00256	MUY_03967
MUY_00256	MUY_04086
MUY_00256	MUY_00435
MUY_00256	MUY_00514
MUY_00256	MUY_00741
MUY_00256	MUY_00780

MUY_00256	MUY_00857
MUY_02733	MUY_02733
MUY_02736	MUY_02736
MUY_02736	MUY_03012
MUY_02736	MUY_03095
MUY_02736	MUY_03863
MUY_02738	MUY_02738
MUY_02740	MUY_02740
MUY_02740	MUY_03823
MUY_02740	MUY_03936
MUY_02740	MUY_03949
MUY_02740	MUY_00849
MUY_00257	MUY_00257
MUY_00257	MUY_03633
MUY_00257	MUY_03837
MUY_02743	MUY_02743
MUY_02744	MUY_02744
MUY_02744	MUY_02745
MUY_02744	MUY_02760
MUY_02744	MUY_00265
MUY_02744	MUY_02894
MUY_02744	MUY_02929
MUY_02744	MUY_02945
MUY_02744	MUY_02946
MUY_02744	MUY_00285
MUY_02744	MUY_03154
MUY_02744	MUY_03330
MUY_02744	MUY_03332
MUY_02744	MUY_03354
MUY_02744	MUY_00322
MUY_02744	MUY_03575
MUY_02744	MUY_03576
MUY_02744	MUY_03582
MUY_02744	MUY_03598
MUY_02744	MUY_03634
MUY_02744	MUY_03649
MUY_02744	MUY_03676
MUY_02744	MUY_03785
MUY_02744	MUY_03805
MUY_02744	MUY_03900
MUY_02744	MUY_03946
MUY_02744	MUY_00372
MUY_02744	MUY_03978
MUY_02744	MUY_04144
MUY_02744	MUY_04172
MUY_02744	MUY_04248
MUY_02744	MUY_04254

MUY_02744	MUY_04266
MUY_02744	MUY_04269
MUY_02744	MUY_04282
MUY_02744	MUY_04296
MUY_02744	MUY_00388
MUY_02744	MUY_04348
MUY_02744	MUY_04357
MUY_02744	MUY_00394
MUY_02744	MUY_04403
MUY_02744	MUY_04416
MUY_02744	MUY_04445
MUY_02744	MUY_04453
MUY_02744	MUY_00420
MUY_02744	MUY_00434
MUY_02744	MUY_00442
MUY_02744	MUY_00516
MUY_02744	MUY_00585
MUY_02744	MUY_00800
MUY_02744	MUY_00815
MUY_02744	MUY_00937
MUY_02744	MUY_00938
MUY_02744	MUY_00939
MUY_02744	MUY_00968
MUY_02744	MUY_00975
MUY_02744	MUY_00996
MUY_02744	MUY_01000
MUY_02745	MUY_02745
MUY_02745	MUY_02760
MUY_02745	MUY_00265
MUY_02745	MUY_02894
MUY_02745	MUY_02929
MUY_02745	MUY_02945
MUY_02745	MUY_02946
MUY_02745	MUY_00285
MUY_02745	MUY_03154
MUY_02745	MUY_03330
MUY_02745	MUY_03332
MUY_02745	MUY_03354
MUY_02745	MUY_00322
MUY_02745	MUY_03575
MUY_02745	MUY_03576
MUY_02745	MUY_03582
MUY_02745	MUY_03598
MUY_02745	MUY_03634
MUY_02745	MUY_03649
MUY_02745	MUY_03676
MUY_02745	MUY_03785

MUY_02745	MUY_03805
MUY_02745	MUY_03900
MUY_02745	MUY_03946
MUY_02745	MUY_00372
MUY_02745	MUY_03978
MUY_02745	MUY_04144
MUY_02745	MUY_04172
MUY_02745	MUY_04248
MUY_02745	MUY_04254
MUY_02745	MUY_04266
MUY_02745	MUY_04269
MUY_02745	MUY_04282
MUY_02745	MUY_04296
MUY_02745	MUY_00388
MUY_02745	MUY_04348
MUY_02745	MUY_04357
MUY_02745	MUY_00394
MUY_02745	MUY_04403
MUY_02745	MUY_04416
MUY_02745	MUY_04445
MUY_02745	MUY_04453
MUY_02745	MUY_00420
MUY_02745	MUY_00434
MUY_02745	MUY_00442
MUY_02745	MUY_00516
MUY_02745	MUY_00585
MUY_02745	MUY_00800
MUY_02745	MUY_00815
MUY_02745	MUY_00937
MUY_02745	MUY_00938
MUY_02745	MUY_00939
MUY_02745	MUY_00968
MUY_02745	MUY_00975
MUY_02745	MUY_00996
MUY_02745	MUY_01000
MUY_02751	MUY_02751
MUY_02751	MUY_03267
MUY_00258	MUY_00258
MUY_00258	MUY_03400
MUY_00258	MUY_00347
MUY_00258	MUY_04132
MUY_00258	MUY_04150
MUY_00258	MUY_04397
MUY_00258	MUY_04438
MUY_00258	MUY_00453
MUY_00258	MUY_00797
MUY_02755	MUY_03267

MUY_02758	MUY_02758
MUY_02758	MUY_04263
MUY_02758	MUY_00946
MUY_02760	MUY_02760
MUY_02760	MUY_00265
MUY_02760	MUY_02894
MUY_02760	MUY_02929
MUY_02760	MUY_02945
MUY_02760	MUY_02946
MUY_02760	MUY_00285
MUY_02760	MUY_03154
MUY_02760	MUY_03330
MUY_02760	MUY_03332
MUY_02760	MUY_03354
MUY_02760	MUY_00322
MUY_02760	MUY_03575
MUY_02760	MUY_03576
MUY_02760	MUY_03582
MUY_02760	MUY_03598
MUY_02760	MUY_03634
MUY_02760	MUY_03649
MUY_02760	MUY_03676
MUY_02760	MUY_03785
MUY_02760	MUY_03805
MUY_02760	MUY_03900
MUY_02760	MUY_03946
MUY_02760	MUY_00372
MUY_02760	MUY_03978
MUY_02760	MUY_04144
MUY_02760	MUY_04172
MUY_02760	MUY_04248
MUY_02760	MUY_04254
MUY_02760	MUY_04266
MUY_02760	MUY_04269
MUY_02760	MUY_04282
MUY_02760	MUY_04296
MUY_02760	MUY_00388
MUY_02760	MUY_04348
MUY_02760	MUY_04357
MUY_02760	MUY_00394
MUY_02760	MUY_04403
MUY_02760	MUY_04416
MUY_02760	MUY_04445
MUY_02760	MUY_04453
MUY_02760	MUY_00420
MUY_02760	MUY_00434
MUY_02760	MUY_00442

MUY_02760	MUY_00516
MUY_02760	MUY_00585
MUY_02760	MUY_00800
MUY_02760	MUY_00815
MUY_02760	MUY_00937
MUY_02760	MUY_00938
MUY_02760	MUY_00939
MUY_02760	MUY_00968
MUY_02760	MUY_00975
MUY_02760	MUY_00996
MUY_02760	MUY_01000
MUY_02762	MUY_02762
MUY_02762	MUY_03902
MUY_02762	MUY_04312
MUY_02762	MUY_00561
MUY_02762	MUY_00734
MUY_02764	MUY_02764
MUY_02764	MUY_03692
MUY_02764	MUY_04193
MUY_02764	MUY_04389
MUY_02764	MUY_04390
MUY_02764	MUY_04393
MUY_02765	MUY_02765
MUY_02765	MUY_02869
MUY_02765	MUY_02983
MUY_02765	MUY_03012
MUY_02765	MUY_03586
MUY_02765	MUY_03863
MUY_02765	MUY_03904
MUY_02765	MUY_03922
MUY_02765	MUY_04367
MUY_02765	MUY_00496
MUY_02765	MUY_00046
MUY_02765	MUY_00527
MUY_02765	MUY_00816
MUY_02767	MUY_02767
MUY_02768	MUY_02768
MUY_02769	MUY_02769
MUY_02770	MUY_02770
MUY_02770	MUY_02771
MUY_02771	MUY_02771
MUY_02780	MUY_02797
MUY_02780	MUY_03069
MUY_02780	MUY_03314
MUY_02780	MUY_00317
MUY_02780	MUY_03425
MUY_02780	MUY_03576

MUY_02780	MUY_03610
MUY_02780	MUY_04138
MUY_02780	MUY_04334
MUY_02780	MUY_04466
MUY_02780	MUY_00435
MUY_02780	MUY_00589
MUY_02780	MUY_00590
MUY_02780	MUY_00780
MUY_02783	MUY_02783
MUY_02783	MUY_00303
MUY_02783	MUY_03505
MUY_02783	MUY_03901
MUY_02784	MUY_02784
MUY_02784	MUY_02845
MUY_02784	MUY_02852
MUY_02784	MUY_04026
MUY_02784	MUY_04258
MUY_02784	MUY_04322
MUY_02784	MUY_04504
MUY_02784	MUY_00541
MUY_02784	MUY_00571
MUY_02785	MUY_02785
MUY_02785	MUY_02788
MUY_02785	MUY_04476
MUY_02785	MUY_00895
MUY_02788	MUY_02788
MUY_02788	MUY_03055
MUY_02788	MUY_04126
MUY_02788	MUY_04239
MUY_02788	MUY_00469
MUY_02788	MUY_00485
MUY_02788	MUY_00696
MUY_02789	MUY_03267
MUY_00260	MUY_00344
MUY_02790	MUY_02790
MUY_02793	MUY_02793
MUY_02793	MUY_03058
MUY_02793	MUY_03089
MUY_02793	MUY_04497
MUY_02793	MUY_04508
MUY_02793	MUY_00804
MUY_02793	MUY_00963
MUY_02794	MUY_02794
MUY_02794	MUY_00023
MUY_02796	MUY_02796
MUY_02797	MUY_02797
MUY_02797	MUY_02829

MUY_02797	MUY_03069
MUY_02797	MUY_03251
MUY_02797	MUY_03314
MUY_02797	MUY_03535
MUY_02797	MUY_03610
MUY_02797	MUY_03789
MUY_02797	MUY_03847
MUY_02797	MUY_04086
MUY_02797	MUY_04122
MUY_02797	MUY_04334
MUY_02797	MUY_04466
MUY_02797	MUY_00435
MUY_02797	MUY_00514
MUY_02797	MUY_00068
MUY_02797	MUY_00759
MUY_02797	MUY_00780
MUY_02798	MUY_02798
MUY_02803	MUY_02803
MUY_02803	MUY_03742
MUY_02803	MUY_03844
MUY_02803	MUY_04025
MUY_02804	MUY_03267
MUY_02805	MUY_03774
MUY_00261	MUY_00261
MUY_00261	MUY_00308
MUY_00261	MUY_03333
MUY_00261	MUY_03386
MUY_00261	MUY_03449
MUY_00261	MUY_03454
MUY_00261	MUY_03637
MUY_00261	MUY_03810
MUY_00261	MUY_04070
MUY_00261	MUY_04242
MUY_00261	MUY_00382
MUY_00261	MUY_04302
MUY_00261	MUY_04383
MUY_00261	MUY_04433
MUY_00261	MUY_04483
MUY_00261	MUY_00450
MUY_00261	MUY_00464
MUY_00261	MUY_00808
MUY_00261	MUY_00840
MUY_02807	MUY_02807
MUY_02808	MUY_02808
MUY_02808	MUY_02815
MUY_02808	MUY_03543
MUY_02808	MUY_04049

MUY_02808	MUY_00810
MUY_02808	MUY_00869
MUY_02809	MUY_02809
MUY_02810	MUY_02810
MUY_02811	MUY_02811
MUY_02811	MUY_02812
MUY_02811	MUY_00541
MUY_02812	MUY_02812
MUY_02812	MUY_03012
MUY_02812	MUY_03267
MUY_02812	MUY_03863
MUY_02813	MUY_02813
MUY_02813	MUY_00344
MUY_02813	MUY_04091
MUY_02813	MUY_00968
MUY_00262	MUY_00262
MUY_00262	MUY_03642
MUY_00262	MUY_00780
MUY_02814	MUY_04508
MUY_02814	MUY_00436
MUY_02814	MUY_00935
MUY_02815	MUY_02815
MUY_02815	MUY_03543
MUY_02815	MUY_04049
MUY_02815	MUY_00810
MUY_02815	MUY_00869
MUY_02816	MUY_02816
MUY_02816	MUY_02821
MUY_02819	MUY_02819
MUY_02820	MUY_03135
MUY_02820	MUY_03476
MUY_02820	MUY_03557
MUY_02820	MUY_03774
MUY_02820	MUY_04205
MUY_02820	MUY_04236
MUY_02820	MUY_00543
MUY_02820	MUY_00998
MUY_02821	MUY_03191
MUY_02821	MUY_03240
MUY_02821	MUY_00024
MUY_02825	MUY_02825
MUY_02826	MUY_02826
MUY_02827	MUY_02827
MUY_02828	MUY_02828
MUY_02829	MUY_02829
MUY_02829	MUY_03251
MUY_02829	MUY_03847

MUY_02829	MUY_04122
MUY_02829	MUY_00472
MUY_02830	MUY_02830
MUY_02830	MUY_03356
MUY_02830	MUY_03860
MUY_02830	MUY_03951
MUY_02830	MUY_04473
MUY_02831	MUY_02831
MUY_00263	MUY_00263
MUY_00263	MUY_00264
MUY_00263	MUY_02912
MUY_00263	MUY_02939
MUY_00263	MUY_02940
MUY_00263	MUY_02964
MUY_00263	MUY_00288
MUY_00263	MUY_00289
MUY_00263	MUY_03059
MUY_00263	MUY_03117
MUY_00263	MUY_03127
MUY_00263	MUY_03139
MUY_00263	MUY_03183
MUY_00263	MUY_03201
MUY_00263	MUY_03202
MUY_00263	MUY_03252
MUY_00263	MUY_03306
MUY_00263	MUY_03374
MUY_00263	MUY_03463
MUY_00263	MUY_03477
MUY_00263	MUY_03478
MUY_00263	MUY_03483
MUY_00263	MUY_03609
MUY_00263	MUY_03619
MUY_00263	MUY_03647
MUY_00263	MUY_03648
MUY_00263	MUY_03924
MUY_00263	MUY_04096
MUY_00263	MUY_04146
MUY_00263	MUY_04177
MUY_00263	MUY_04211
MUY_00263	MUY_04212
MUY_00263	MUY_04293
MUY_00263	MUY_04294
MUY_00263	MUY_04358
MUY_00263	MUY_04359
MUY_00263	MUY_04379
MUY_00263	MUY_04417
MUY_00263	MUY_04418

MUY_00263	MUY_04447
MUY_00263	MUY_04448
MUY_00263	MUY_04471
MUY_00263	MUY_00418
MUY_00263	MUY_00436
MUY_00263	MUY_00575
MUY_00263	MUY_00576
MUY_00263	MUY_00790
MUY_00263	MUY_00961
MUY_02832	MUY_02832
MUY_02832	MUY_00965
MUY_02834	MUY_02834
MUY_02834	MUY_03186
MUY_02834	MUY_03876
MUY_02834	MUY_04337
MUY_02834	MUY_00708
MUY_02837	MUY_02837
MUY_02838	MUY_02838
MUY_02841	MUY_02841
MUY_02841	MUY_02941
MUY_02841	MUY_03513
MUY_02841	MUY_03515
MUY_02841	MUY_03524
MUY_02841	MUY_03862
MUY_02841	MUY_00461
MUY_00264	MUY_00264
MUY_00264	MUY_02912
MUY_00264	MUY_02939
MUY_00264	MUY_02940
MUY_00264	MUY_00288
MUY_00264	MUY_00289
MUY_00264	MUY_03183
MUY_00264	MUY_03201
MUY_00264	MUY_03202
MUY_00264	MUY_03463
MUY_00264	MUY_03477
MUY_00264	MUY_03478
MUY_00264	MUY_03609
MUY_00264	MUY_03610
MUY_00264	MUY_03619
MUY_00264	MUY_03620
MUY_00264	MUY_03647
MUY_00264	MUY_03648
MUY_00264	MUY_03924
MUY_00264	MUY_04096
MUY_00264	MUY_04146
MUY_00264	MUY_04177

MUY_00264	MUY_04211
MUY_00264	MUY_04212
MUY_00264	MUY_04293
MUY_00264	MUY_04294
MUY_00264	MUY_04358
MUY_00264	MUY_04359
MUY_00264	MUY_00393
MUY_00264	MUY_04417
MUY_00264	MUY_04418
MUY_00264	MUY_04420
MUY_00264	MUY_04447
MUY_00264	MUY_04448
MUY_00264	MUY_04470
MUY_00264	MUY_04471
MUY_00264	MUY_00418
MUY_00264	MUY_00436
MUY_00264	MUY_00438
MUY_00264	MUY_00575
MUY_00264	MUY_00576
MUY_02845	MUY_02845
MUY_02845	MUY_02852
MUY_02845	MUY_02889
MUY_02845	MUY_02892
MUY_02845	MUY_02900
MUY_02845	MUY_03708
MUY_02845	MUY_03918
MUY_02845	MUY_04026
MUY_02845	MUY_04258
MUY_02845	MUY_04322
MUY_02845	MUY_04504
MUY_02845	MUY_00541
MUY_02845	MUY_00571
MUY_02845	MUY_00628
MUY_02852	MUY_02852
MUY_02852	MUY_02889
MUY_02852	MUY_02892
MUY_02852	MUY_02900
MUY_02852	MUY_03063
MUY_02852	MUY_03708
MUY_02852	MUY_03918
MUY_02852	MUY_04026
MUY_02852	MUY_04258
MUY_02852	MUY_04322
MUY_02852	MUY_04504
MUY_02852	MUY_00541
MUY_02852	MUY_00571
MUY_02852	MUY_00628

MUY_00265	MUY_00265
MUY_00265	MUY_02894
MUY_00265	MUY_02929
MUY_00265	MUY_02945
MUY_00265	MUY_02946
MUY_00265	MUY_00285
MUY_00265	MUY_03154
MUY_00265	MUY_03330
MUY_00265	MUY_03332
MUY_00265	MUY_03354
MUY_00265	MUY_00322
MUY_00265	MUY_03575
MUY_00265	MUY_03576
MUY_00265	MUY_03582
MUY_00265	MUY_03598
MUY_00265	MUY_03634
MUY_00265	MUY_03649
MUY_00265	MUY_03676
MUY_00265	MUY_03785
MUY_00265	MUY_03805
MUY_00265	MUY_03900
MUY_00265	MUY_03946
MUY_00265	MUY_00372
MUY_00265	MUY_03978
MUY_00265	MUY_04144
MUY_00265	MUY_04172
MUY_00265	MUY_04248
MUY_00265	MUY_04254
MUY_00265	MUY_04266
MUY_00265	MUY_04269
MUY_00265	MUY_04282
MUY_00265	MUY_04296
MUY_00265	MUY_00388
MUY_00265	MUY_04348
MUY_00265	MUY_04357
MUY_00265	MUY_00394
MUY_00265	MUY_04403
MUY_00265	MUY_04416
MUY_00265	MUY_04445
MUY_00265	MUY_04453
MUY_00265	MUY_00420
MUY_00265	MUY_00434
MUY_00265	MUY_00442
MUY_00265	MUY_00516
MUY_00265	MUY_00585
MUY_00265	MUY_00800
MUY_00265	MUY_00815

MUY_00265	MUY_00937
MUY_00265	MUY_00938
MUY_00265	MUY_00939
MUY_00265	MUY_00968
MUY_00265	MUY_00975
MUY_00265	MUY_00996
MUY_00265	MUY_01000
MUY_02856	MUY_02856
MUY_00019	MUY_00019
MUY_02869	MUY_02869
MUY_02869	MUY_03586
MUY_02869	MUY_03904
MUY_02869	MUY_03922
MUY_02869	MUY_04367
MUY_02869	MUY_00496
MUY_02869	MUY_00046
MUY_02869	MUY_00527
MUY_02869	MUY_00008
MUY_02869	MUY_00816
MUY_02870	MUY_02870
MUY_02870	MUY_04004
MUY_02870	MUY_04495
MUY_02874	MUY_02874
MUY_02881	MUY_02881
MUY_02881	MUY_03112
MUY_02882	MUY_02882
MUY_02882	MUY_02883
MUY_02882	MUY_02889
MUY_02882	MUY_02892
MUY_02882	MUY_02900
MUY_02882	MUY_03708
MUY_02882	MUY_03769
MUY_02882	MUY_03778
MUY_02882	MUY_03830
MUY_02882	MUY_04185
MUY_02882	MUY_04271
MUY_02882	MUY_04291
MUY_02882	MUY_04501
MUY_02882	MUY_04505
MUY_02882	MUY_00551
MUY_02882	MUY_00599
MUY_02882	MUY_00602
MUY_02882	MUY_00628
MUY_02882	MUY_00691
MUY_02882	MUY_00795
MUY_02882	MUY_00086
MUY_02882	MUY_00973

MUY_02883	MUY_02883
MUY_02883	MUY_02889
MUY_02883	MUY_02892
MUY_02883	MUY_02900
MUY_02883	MUY_03708
MUY_02883	MUY_03769
MUY_02883	MUY_03778
MUY_02883	MUY_03830
MUY_02883	MUY_04185
MUY_02883	MUY_04271
MUY_02883	MUY_04291
MUY_02883	MUY_04501
MUY_02883	MUY_04505
MUY_02883	MUY_00551
MUY_02883	MUY_00599
MUY_02883	MUY_00602
MUY_02883	MUY_00628
MUY_02883	MUY_00691
MUY_02883	MUY_00795
MUY_02883	MUY_00086
MUY_02883	MUY_00973
MUY_02889	MUY_02889
MUY_02889	MUY_02892
MUY_02889	MUY_02900
MUY_02889	MUY_03708
MUY_02889	MUY_03769
MUY_02889	MUY_03778
MUY_02889	MUY_03830
MUY_02889	MUY_03918
MUY_02889	MUY_04026
MUY_02889	MUY_04258
MUY_02889	MUY_04271
MUY_02889	MUY_04291
MUY_02889	MUY_04322
MUY_02889	MUY_04504
MUY_02889	MUY_00541
MUY_02889	MUY_00551
MUY_02889	MUY_00571
MUY_02889	MUY_00599
MUY_02889	MUY_00602
MUY_02889	MUY_00628
MUY_02889	MUY_00691
MUY_02889	MUY_00795
MUY_02889	MUY_00086
MUY_02889	MUY_00973
MUY_00272	MUY_00272
MUY_00272	MUY_03126

MUY_00272	MUY_03240
MUY_00272	MUY_03659
MUY_00272	MUY_03701
MUY_00272	MUY_03880
MUY_00272	MUY_04014
MUY_00272	MUY_04194
MUY_00272	MUY_00556
MUY_00272	MUY_00710
MUY_02892	MUY_02892
MUY_02892	MUY_02900
MUY_02892	MUY_03708
MUY_02892	MUY_03769
MUY_02892	MUY_03778
MUY_02892	MUY_03830
MUY_02892	MUY_03918
MUY_02892	MUY_04026
MUY_02892	MUY_04258
MUY_02892	MUY_04271
MUY_02892	MUY_04291
MUY_02892	MUY_04322
MUY_02892	MUY_04504
MUY_02892	MUY_00541
MUY_02892	MUY_00551
MUY_02892	MUY_00571
MUY_02892	MUY_00599
MUY_02892	MUY_00602
MUY_02892	MUY_00628
MUY_02892	MUY_00691
MUY_02892	MUY_00795
MUY_02892	MUY_00086
MUY_02892	MUY_00973
MUY_00273	MUY_00273
MUY_00273	MUY_03141
MUY_00273	MUY_03241
MUY_00273	MUY_03261
MUY_00273	MUY_03373
MUY_00273	MUY_00376
MUY_00273	MUY_04033
MUY_00273	MUY_04034
MUY_00273	MUY_04036
MUY_00273	MUY_00377
MUY_00273	MUY_00378
MUY_00273	MUY_04228
MUY_00273	MUY_04230
MUY_00273	MUY_00379
MUY_02894	MUY_02894
MUY_02894	MUY_02929

MUY_02894	MUY_02945
MUY_02894	MUY_02946
MUY_02894	MUY_00285
MUY_02894	MUY_03154
MUY_02894	MUY_03330
MUY_02894	MUY_03332
MUY_02894	MUY_03354
MUY_02894	MUY_00322
MUY_02894	MUY_03575
MUY_02894	MUY_03576
MUY_02894	MUY_03582
MUY_02894	MUY_03598
MUY_02894	MUY_03634
MUY_02894	MUY_03649
MUY_02894	MUY_03676
MUY_02894	MUY_03785
MUY_02894	MUY_03805
MUY_02894	MUY_03900
MUY_02894	MUY_03946
MUY_02894	MUY_00372
MUY_02894	MUY_03978
MUY_02894	MUY_04144
MUY_02894	MUY_04172
MUY_02894	MUY_04248
MUY_02894	MUY_04254
MUY_02894	MUY_04266
MUY_02894	MUY_04269
MUY_02894	MUY_04282
MUY_02894	MUY_04296
MUY_02894	MUY_00388
MUY_02894	MUY_04348
MUY_02894	MUY_04357
MUY_02894	MUY_00394
MUY_02894	MUY_04403
MUY_02894	MUY_04416
MUY_02894	MUY_04445
MUY_02894	MUY_04453
MUY_02894	MUY_00420
MUY_02894	MUY_00434
MUY_02894	MUY_00442
MUY_02894	MUY_00516
MUY_02894	MUY_00585
MUY_02894	MUY_00800
MUY_02894	MUY_00815
MUY_02894	MUY_00937
MUY_02894	MUY_00938
MUY_02894	MUY_00939

MUY_02894	MUY_00968
MUY_02894	MUY_00975
MUY_02894	MUY_00996
MUY_02894	MUY_01000
MUY_00020	MUY_00020
MUY_02896	MUY_02896
MUY_02896	MUY_04439
MUY_02896	MUY_00945
MUY_02900	MUY_02900
MUY_02900	MUY_03708
MUY_02900	MUY_03769
MUY_02900	MUY_03778
MUY_02900	MUY_03830
MUY_02900	MUY_03918
MUY_02900	MUY_04026
MUY_02900	MUY_04258
MUY_02900	MUY_04271
MUY_02900	MUY_04291
MUY_02900	MUY_04322
MUY_02900	MUY_04504
MUY_02900	MUY_00541
MUY_02900	MUY_00551
MUY_02900	MUY_00571
MUY_02900	MUY_00599
MUY_02900	MUY_00602
MUY_02900	MUY_00628
MUY_02900	MUY_00691
MUY_02900	MUY_00795
MUY_02900	MUY_00086
MUY_02900	MUY_00973
MUY_02910	MUY_02910
MUY_02910	MUY_02917
MUY_02910	MUY_03284
MUY_02910	MUY_03304
MUY_02910	MUY_00316
MUY_02910	MUY_03706
MUY_02910	MUY_04467
MUY_02910	MUY_00824
MUY_02912	MUY_02912
MUY_02912	MUY_02939
MUY_02912	MUY_02940
MUY_02912	MUY_02964
MUY_02912	MUY_00288
MUY_02912	MUY_00289
MUY_02912	MUY_03059
MUY_02912	MUY_03117
MUY_02912	MUY_03127

MUY_02912	MUY_03139
MUY_02912	MUY_03183
MUY_02912	MUY_03201
MUY_02912	MUY_03202
MUY_02912	MUY_03252
MUY_02912	MUY_03306
MUY_02912	MUY_03374
MUY_02912	MUY_03463
MUY_02912	MUY_03477
MUY_02912	MUY_03478
MUY_02912	MUY_03483
MUY_02912	MUY_03609
MUY_02912	MUY_03619
MUY_02912	MUY_03647
MUY_02912	MUY_03648
MUY_02912	MUY_03924
MUY_02912	MUY_04096
MUY_02912	MUY_04146
MUY_02912	MUY_04177
MUY_02912	MUY_04211
MUY_02912	MUY_04212
MUY_02912	MUY_04293
MUY_02912	MUY_04294
MUY_02912	MUY_04358
MUY_02912	MUY_04359
MUY_02912	MUY_04379
MUY_02912	MUY_04417
MUY_02912	MUY_04418
MUY_02912	MUY_04447
MUY_02912	MUY_04448
MUY_02912	MUY_04471
MUY_02912	MUY_00418
MUY_02912	MUY_00436
MUY_02912	MUY_00575
MUY_02912	MUY_00576
MUY_02912	MUY_00790
MUY_02912	MUY_00961
MUY_02913	MUY_02913
MUY_02913	MUY_00397
MUY_02914	MUY_02914
MUY_02914	MUY_02941
MUY_02914	MUY_03136
MUY_02914	MUY_03232
MUY_02914	MUY_03279
MUY_02914	MUY_03513
MUY_02914	MUY_03515
MUY_02914	MUY_03524

MUY_02914	MUY_03585
MUY_02914	MUY_04073
MUY_02914	MUY_00461
MUY_02914	MUY_00523
MUY_02914	MUY_00861
MUY_02916	MUY_03014
MUY_02916	MUY_03054
MUY_02916	MUY_03244
MUY_02916	MUY_03556
MUY_02916	MUY_03574
MUY_02916	MUY_03660
MUY_02916	MUY_00766
MUY_02917	MUY_02917
MUY_02917	MUY_02941
MUY_02917	MUY_03041
MUY_02917	MUY_03284
MUY_02917	MUY_03304
MUY_02917	MUY_00316
MUY_02917	MUY_03513
MUY_02917	MUY_03515
MUY_02917	MUY_03524
MUY_02917	MUY_03706
MUY_02917	MUY_00366
MUY_02917	MUY_04467
MUY_02917	MUY_00461
MUY_02917	MUY_00072
MUY_02917	MUY_00824
MUY_02918	MUY_03014
MUY_02918	MUY_03054
MUY_02918	MUY_03244
MUY_02918	MUY_03556
MUY_02918	MUY_03574
MUY_02918	MUY_03660
MUY_02918	MUY_00766
MUY_02919	MUY_02919
MUY_02921	MUY_02921
MUY_02922	MUY_02922
MUY_02925	MUY_02925
MUY_02927	MUY_02927
MUY_02927	MUY_03083
MUY_00275	MUY_00275
MUY_02929	MUY_02929
MUY_02929	MUY_02945
MUY_02929	MUY_02946
MUY_02929	MUY_00285
MUY_02929	MUY_03154
MUY_02929	MUY_03330

MUY_02929	MUY_03332
MUY_02929	MUY_03354
MUY_02929	MUY_00322
MUY_02929	MUY_03575
MUY_02929	MUY_03576
MUY_02929	MUY_03582
MUY_02929	MUY_03598
MUY_02929	MUY_03634
MUY_02929	MUY_03649
MUY_02929	MUY_03676
MUY_02929	MUY_03785
MUY_02929	MUY_03805
MUY_02929	MUY_03900
MUY_02929	MUY_03946
MUY_02929	MUY_00372
MUY_02929	MUY_03978
MUY_02929	MUY_04144
MUY_02929	MUY_04172
MUY_02929	MUY_04248
MUY_02929	MUY_04254
MUY_02929	MUY_04266
MUY_02929	MUY_04269
MUY_02929	MUY_04282
MUY_02929	MUY_04296
MUY_02929	MUY_00388
MUY_02929	MUY_04348
MUY_02929	MUY_04357
MUY_02929	MUY_00394
MUY_02929	MUY_04403
MUY_02929	MUY_04416
MUY_02929	MUY_04445
MUY_02929	MUY_04453
MUY_02929	MUY_00420
MUY_02929	MUY_00434
MUY_02929	MUY_00442
MUY_02929	MUY_00516
MUY_02929	MUY_00585
MUY_02929	MUY_00800
MUY_02929	MUY_00815
MUY_02929	MUY_00937
MUY_02929	MUY_00938
MUY_02929	MUY_00939
MUY_02929	MUY_00968
MUY_02929	MUY_00975
MUY_02929	MUY_00996
MUY_02929	MUY_01000
MUY_02931	MUY_02931

MUY_02931	MUY_00047
MUY_02932	MUY_02932
MUY_02932	MUY_00291
MUY_02935	MUY_02935
MUY_02935	MUY_03680
MUY_02935	MUY_00743
MUY_02935	MUY_00900
MUY_02936	MUY_02936
MUY_02937	MUY_02937
MUY_02937	MUY_02972
MUY_02937	MUY_03839
MUY_02937	MUY_00560
MUY_02937	MUY_00855
MUY_02937	MUY_00904
MUY_02939	MUY_02939
MUY_02939	MUY_02940
MUY_02939	MUY_00288
MUY_02939	MUY_00289
MUY_02939	MUY_03183
MUY_02939	MUY_03202
MUY_02939	MUY_03463
MUY_02939	MUY_03477
MUY_02939	MUY_03478
MUY_02939	MUY_03609
MUY_02939	MUY_03610
MUY_02939	MUY_03619
MUY_02939	MUY_03620
MUY_02939	MUY_03647
MUY_02939	MUY_03648
MUY_02939	MUY_03924
MUY_02939	MUY_03925
MUY_02939	MUY_04096
MUY_02939	MUY_04145
MUY_02939	MUY_04146
MUY_02939	MUY_04177
MUY_02939	MUY_04211
MUY_02939	MUY_04212
MUY_02939	MUY_04293
MUY_02939	MUY_04294
MUY_02939	MUY_04358
MUY_02939	MUY_04359
MUY_02939	MUY_00393
MUY_02939	MUY_04402
MUY_02939	MUY_04417
MUY_02939	MUY_04418
MUY_02939	MUY_04420
MUY_02939	MUY_04447

MUY_02939	MUY_04448
MUY_02939	MUY_04470
MUY_02939	MUY_04471
MUY_02939	MUY_00418
MUY_02939	MUY_00436
MUY_02939	MUY_00438
MUY_02939	MUY_00575
MUY_02939	MUY_00576
MUY_02940	MUY_02940
MUY_02940	MUY_02964
MUY_02940	MUY_00288
MUY_02940	MUY_00289
MUY_02940	MUY_03059
MUY_02940	MUY_03117
MUY_02940	MUY_03127
MUY_02940	MUY_03139
MUY_02940	MUY_03183
MUY_02940	MUY_03201
MUY_02940	MUY_03202
MUY_02940	MUY_03252
MUY_02940	MUY_03306
MUY_02940	MUY_03374
MUY_02940	MUY_03463
MUY_02940	MUY_03477
MUY_02940	MUY_03478
MUY_02940	MUY_03483
MUY_02940	MUY_03609
MUY_02940	MUY_03619
MUY_02940	MUY_03647
MUY_02940	MUY_03648
MUY_02940	MUY_03924
MUY_02940	MUY_04096
MUY_02940	MUY_04146
MUY_02940	MUY_04177
MUY_02940	MUY_04211
MUY_02940	MUY_04212
MUY_02940	MUY_04293
MUY_02940	MUY_04294
MUY_02940	MUY_04358
MUY_02940	MUY_04359
MUY_02940	MUY_04379
MUY_02940	MUY_04417
MUY_02940	MUY_04418
MUY_02940	MUY_04447
MUY_02940	MUY_04448
MUY_02940	MUY_04471
MUY_02940	MUY_00418

MUY_02940	MUY_00436
MUY_02940	MUY_00575
MUY_02940	MUY_00576
MUY_02940	MUY_00790
MUY_02940	MUY_00961
MUY_02941	MUY_02941
MUY_02941	MUY_03136
MUY_02941	MUY_03137
MUY_02941	MUY_03138
MUY_02941	MUY_03279
MUY_02941	MUY_03304
MUY_02941	MUY_00316
MUY_02941	MUY_03513
MUY_02941	MUY_03515
MUY_02941	MUY_03524
MUY_02941	MUY_03585
MUY_02941	MUY_03706
MUY_02941	MUY_03840
MUY_02941	MUY_00459
MUY_02941	MUY_00461
MUY_02941	MUY_00523
MUY_02941	MUY_00895
MUY_02942	MUY_02942
MUY_02945	MUY_02945
MUY_02945	MUY_02946
MUY_02945	MUY_00285
MUY_02945	MUY_03154
MUY_02945	MUY_03330
MUY_02945	MUY_03332
MUY_02945	MUY_03354
MUY_02945	MUY_00322
MUY_02945	MUY_03575
MUY_02945	MUY_03576
MUY_02945	MUY_03582
MUY_02945	MUY_03598
MUY_02945	MUY_03634
MUY_02945	MUY_03649
MUY_02945	MUY_03676
MUY_02945	MUY_03785
MUY_02945	MUY_03805
MUY_02945	MUY_03900
MUY_02945	MUY_03946
MUY_02945	MUY_00372
MUY_02945	MUY_03978
MUY_02945	MUY_04144
MUY_02945	MUY_04172
MUY_02945	MUY_04248

MUY_02945	MUY_04254
MUY_02945	MUY_04266
MUY_02945	MUY_04269
MUY_02945	MUY_04282
MUY_02945	MUY_04296
MUY_02945	MUY_00388
MUY_02945	MUY_04348
MUY_02945	MUY_04357
MUY_02945	MUY_00394
MUY_02945	MUY_04403
MUY_02945	MUY_04416
MUY_02945	MUY_04445
MUY_02945	MUY_04453
MUY_02945	MUY_00420
MUY_02945	MUY_00434
MUY_02945	MUY_00442
MUY_02945	MUY_00516
MUY_02945	MUY_00585
MUY_02945	MUY_00800
MUY_02945	MUY_00815
MUY_02945	MUY_00937
MUY_02945	MUY_00938
MUY_02945	MUY_00939
MUY_02945	MUY_00968
MUY_02945	MUY_00975
MUY_02945	MUY_00996
MUY_02945	MUY_01000
MUY_02946	MUY_02946
MUY_02946	MUY_00285
MUY_02946	MUY_03154
MUY_02946	MUY_03330
MUY_02946	MUY_03332
MUY_02946	MUY_03354
MUY_02946	MUY_00322
MUY_02946	MUY_03575
MUY_02946	MUY_03576
MUY_02946	MUY_03582
MUY_02946	MUY_03598
MUY_02946	MUY_03634
MUY_02946	MUY_03649
MUY_02946	MUY_03676
MUY_02946	MUY_03785
MUY_02946	MUY_03805
MUY_02946	MUY_03900
MUY_02946	MUY_03946
MUY_02946	MUY_00372
MUY_02946	MUY_03978

MUY_02946	MUY_04144
MUY_02946	MUY_04172
MUY_02946	MUY_04248
MUY_02946	MUY_04254
MUY_02946	MUY_04266
MUY_02946	MUY_04269
MUY_02946	MUY_04282
MUY_02946	MUY_04296
MUY_02946	MUY_00388
MUY_02946	MUY_04348
MUY_02946	MUY_04357
MUY_02946	MUY_00394
MUY_02946	MUY_04403
MUY_02946	MUY_04416
MUY_02946	MUY_04445
MUY_02946	MUY_04453
MUY_02946	MUY_00420
MUY_02946	MUY_00434
MUY_02946	MUY_00442
MUY_02946	MUY_00516
MUY_02946	MUY_00585
MUY_02946	MUY_00800
MUY_02946	MUY_00815
MUY_02946	MUY_00937
MUY_02946	MUY_00938
MUY_02946	MUY_00939
MUY_02946	MUY_00968
MUY_02946	MUY_00975
MUY_02946	MUY_00996
MUY_02946	MUY_01000
MUY_02947	MUY_02947
MUY_02947	MUY_00277
MUY_02947	MUY_00337
MUY_02947	MUY_04086
MUY_02947	MUY_00433
MUY_02947	MUY_00813
MUY_00277	MUY_00277
MUY_00277	MUY_00337
MUY_00277	MUY_04086
MUY_00277	MUY_00433
MUY_00277	MUY_00813
MUY_02950	MUY_02950
MUY_02950	MUY_02958
MUY_02950	MUY_00292
MUY_02950	MUY_03128
MUY_02950	MUY_03225
MUY_02950	MUY_00313

MUY_02950	MUY_03494
MUY_02950	MUY_03606
MUY_02950	MUY_03627
MUY_02950	MUY_03638
MUY_02950	MUY_03964
MUY_02950	MUY_03998
MUY_02950	MUY_04038
MUY_02950	MUY_04045
MUY_02950	MUY_00486
MUY_02950	MUY_00681
MUY_02950	MUY_00721
MUY_02950	MUY_00933
MUY_02951	MUY_02951
MUY_02952	MUY_02952
MUY_02952	MUY_03352
MUY_02958	MUY_02958
MUY_02958	MUY_00292
MUY_02958	MUY_03128
MUY_02958	MUY_03225
MUY_02958	MUY_00313
MUY_02958	MUY_03494
MUY_02958	MUY_03606
MUY_02958	MUY_03627
MUY_02958	MUY_03638
MUY_02958	MUY_03964
MUY_02958	MUY_03998
MUY_02958	MUY_04038
MUY_02958	MUY_04045
MUY_02958	MUY_00486
MUY_02958	MUY_00681
MUY_02958	MUY_00721
MUY_02958	MUY_00933
MUY_02959	MUY_02959
MUY_02959	MUY_03842
MUY_02959	MUY_04155
MUY_02959	MUY_04326
MUY_00279	MUY_00279
MUY_00279	MUY_03223
MUY_00279	MUY_00326
MUY_00279	MUY_04247
MUY_02962	MUY_02962
MUY_02962	MUY_02963
MUY_02963	MUY_02963
MUY_02963	MUY_03856
MUY_02964	MUY_02964
MUY_02964	MUY_00288
MUY_02964	MUY_03183

MUY_02964	MUY_03202
MUY_02964	MUY_03463
MUY_02964	MUY_03477
MUY_02964	MUY_03609
MUY_02964	MUY_03619
MUY_02964	MUY_03648
MUY_02964	MUY_03924
MUY_02964	MUY_04096
MUY_02964	MUY_04146
MUY_02964	MUY_04177
MUY_02964	MUY_04212
MUY_02964	MUY_04293
MUY_02964	MUY_04359
MUY_02964	MUY_04418
MUY_02964	MUY_04448
MUY_02964	MUY_04471
MUY_02964	MUY_00418
MUY_02964	MUY_00436
MUY_02964	MUY_00576
MUY_02969	MUY_02969
MUY_02970	MUY_02970
MUY_02970	MUY_03658
MUY_02970	MUY_04399
MUY_02971	MUY_04136
MUY_00281	MUY_00281
MUY_00021	MUY_00021
MUY_00021	MUY_03050
MUY_02972	MUY_02972
MUY_02972	MUY_03839
MUY_02972	MUY_00560
MUY_02972	MUY_00855
MUY_02972	MUY_00904
MUY_02973	MUY_02973
MUY_02975	MUY_02975
MUY_02975	MUY_03084
MUY_02977	MUY_02977
MUY_02977	MUY_03601
MUY_02977	MUY_03792
MUY_02977	MUY_04042
MUY_02977	MUY_04408
MUY_02977	MUY_00443
MUY_02978	MUY_02978
MUY_02978	MUY_00290
MUY_02978	MUY_03320
MUY_02978	MUY_03640
MUY_02978	MUY_03984
MUY_02978	MUY_04198

MUY_02978	MUY_00401
MUY_02978	MUY_00563
MUY_02981	MUY_02981
MUY_02981	MUY_03699
MUY_02981	MUY_00378
MUY_02981	MUY_04230
MUY_02981	MUY_00750
MUY_02981	MUY_00811
MUY_02982	MUY_02982
MUY_02982	MUY_03140
MUY_02982	MUY_03250
MUY_02982	MUY_03431
MUY_02982	MUY_00323
MUY_02982	MUY_03553
MUY_02982	MUY_03612
MUY_02982	MUY_00445
MUY_02982	MUY_00677
MUY_02982	MUY_00749
MUY_02982	MUY_00779
MUY_02982	MUY_00882
MUY_02983	MUY_02983
MUY_02983	MUY_03518
MUY_02983	MUY_03612
MUY_02983	MUY_04508
MUY_02983	MUY_00589
MUY_02983	MUY_00590
MUY_02983	MUY_00780
MUY_02983	MUY_00075
MUY_00283	MUY_03496
MUY_02984	MUY_02984
MUY_02984	MUY_02993
MUY_02984	MUY_00792
MUY_02990	MUY_02990
MUY_02991	MUY_02991
MUY_02991	MUY_03263
MUY_02991	MUY_03292
MUY_02991	MUY_03434
MUY_02991	MUY_04020
MUY_02991	MUY_04022
MUY_02992	MUY_02992
MUY_02992	MUY_03383
MUY_02993	MUY_02993
MUY_02993	MUY_00792
MUY_02996	MUY_02996
MUY_02996	MUY_03071
MUY_02997	MUY_02997
MUY_02997	MUY_03267

MUY_02998	MUY_02998
MUY_03001	MUY_03001
MUY_03004	MUY_03004
MUY_00285	MUY_00285
MUY_00285	MUY_03154
MUY_00285	MUY_03330
MUY_00285	MUY_03332
MUY_00285	MUY_03354
MUY_00285	MUY_00322
MUY_00285	MUY_03575
MUY_00285	MUY_03576
MUY_00285	MUY_03582
MUY_00285	MUY_03598
MUY_00285	MUY_03634
MUY_00285	MUY_03649
MUY_00285	MUY_03676
MUY_00285	MUY_03785
MUY_00285	MUY_03805
MUY_00285	MUY_03900
MUY_00285	MUY_03946
MUY_00285	MUY_00372
MUY_00285	MUY_03978
MUY_00285	MUY_04144
MUY_00285	MUY_04172
MUY_00285	MUY_04248
MUY_00285	MUY_04254
MUY_00285	MUY_04266
MUY_00285	MUY_04269
MUY_00285	MUY_04282
MUY_00285	MUY_04296
MUY_00285	MUY_00388
MUY_00285	MUY_04348
MUY_00285	MUY_04357
MUY_00285	MUY_00394
MUY_00285	MUY_04403
MUY_00285	MUY_04416
MUY_00285	MUY_04445
MUY_00285	MUY_04453
MUY_00285	MUY_00420
MUY_00285	MUY_00434
MUY_00285	MUY_00442
MUY_00285	MUY_00516
MUY_00285	MUY_00585
MUY_00285	MUY_00800
MUY_00285	MUY_00815
MUY_00285	MUY_00937
MUY_00285	MUY_00938

MUY_00285	MUY_00939
MUY_00285	MUY_00968
MUY_00285	MUY_00975
MUY_00285	MUY_00996
MUY_00285	MUY_01000
MUY_03006	MUY_03006
MUY_03006	MUY_04216
MUY_03010	MUY_03010
MUY_03012	MUY_03012
MUY_03012	MUY_03094
MUY_03012	MUY_03095
MUY_03012	MUY_03117
MUY_03012	MUY_03306
MUY_03012	MUY_03863
MUY_03012	MUY_03904
MUY_03012	MUY_03922
MUY_03012	MUY_04367
MUY_03012	MUY_00527
MUY_03012	MUY_00790
MUY_03012	MUY_00816
MUY_03012	MUY_00857
MUY_03012	MUY_00961
MUY_03013	MUY_03013
MUY_03013	MUY_00344
MUY_03014	MUY_03014
MUY_03014	MUY_03054
MUY_03014	MUY_03244
MUY_03014	MUY_03556
MUY_03014	MUY_03573
MUY_03014	MUY_03574
MUY_03014	MUY_03660
MUY_03014	MUY_00766
MUY_03015	MUY_03015
MUY_03015	MUY_03490
MUY_03015	MUY_04046
MUY_03016	MUY_03016
MUY_03016	MUY_03038
MUY_03017	MUY_03017
MUY_03019	MUY_03019
MUY_03019	MUY_03267
MUY_03020	MUY_03020
MUY_03020	MUY_03267
MUY_03020	MUY_03871
MUY_03020	MUY_03872
MUY_03023	MUY_03023
MUY_03024	MUY_03024
MUY_00288	MUY_00288

MUY_00288	MUY_00289
MUY_00288	MUY_03059
MUY_00288	MUY_03117
MUY_00288	MUY_03127
MUY_00288	MUY_03139
MUY_00288	MUY_03183
MUY_00288	MUY_03201
MUY_00288	MUY_03202
MUY_00288	MUY_03252
MUY_00288	MUY_03306
MUY_00288	MUY_03374
MUY_00288	MUY_03463
MUY_00288	MUY_03477
MUY_00288	MUY_03478
MUY_00288	MUY_03483
MUY_00288	MUY_03609
MUY_00288	MUY_03619
MUY_00288	MUY_03647
MUY_00288	MUY_03648
MUY_00288	MUY_03924
MUY_00288	MUY_04096
MUY_00288	MUY_04146
MUY_00288	MUY_04177
MUY_00288	MUY_04211
MUY_00288	MUY_04212
MUY_00288	MUY_04293
MUY_00288	MUY_04294
MUY_00288	MUY_04358
MUY_00288	MUY_04359
MUY_00288	MUY_04379
MUY_00288	MUY_04417
MUY_00288	MUY_04418
MUY_00288	MUY_04447
MUY_00288	MUY_04448
MUY_00288	MUY_04471
MUY_00288	MUY_00418
MUY_00288	MUY_00436
MUY_00288	MUY_00575
MUY_00288	MUY_00576
MUY_00288	MUY_00790
MUY_00288	MUY_00961
MUY_03025	MUY_03025
MUY_03025	MUY_03345
MUY_03025	MUY_04174
MUY_03025	MUY_00053
MUY_03025	MUY_00057
MUY_03025	MUY_00743

MUY_03025	MUY_00773
MUY_03025	MUY_00074
MUY_03026	MUY_03026
MUY_03026	MUY_03220
MUY_03026	MUY_04017
MUY_03029	MUY_03029
MUY_00289	MUY_00289
MUY_00289	MUY_03183
MUY_00289	MUY_03201
MUY_00289	MUY_03202
MUY_00289	MUY_03463
MUY_00289	MUY_03477
MUY_00289	MUY_03478
MUY_00289	MUY_03609
MUY_00289	MUY_03610
MUY_00289	MUY_03619
MUY_00289	MUY_03620
MUY_00289	MUY_03647
MUY_00289	MUY_03648
MUY_00289	MUY_03924
MUY_00289	MUY_04096
MUY_00289	MUY_04146
MUY_00289	MUY_04177
MUY_00289	MUY_04211
MUY_00289	MUY_04212
MUY_00289	MUY_04293
MUY_00289	MUY_04294
MUY_00289	MUY_04358
MUY_00289	MUY_04359
MUY_00289	MUY_00393
MUY_00289	MUY_04417
MUY_00289	MUY_04418
MUY_00289	MUY_04420
MUY_00289	MUY_04447
MUY_00289	MUY_04448
MUY_00289	MUY_04470
MUY_00289	MUY_04471
MUY_00289	MUY_00418
MUY_00289	MUY_00436
MUY_00289	MUY_00438
MUY_00289	MUY_00575
MUY_00289	MUY_00576
MUY_03031	MUY_03031
MUY_03031	MUY_03440
MUY_03034	MUY_03034
MUY_03034	MUY_00303
MUY_03034	MUY_04136

MUY_03034	MUY_00443
MUY_03034	MUY_00709
MUY_03034	MUY_00851
MUY_03035	MUY_03035
MUY_03036	MUY_03036
MUY_03036	MUY_00344
MUY_03036	MUY_03963
MUY_03036	MUY_00420
MUY_03038	MUY_03038
MUY_03039	MUY_03039
MUY_03039	MUY_00344
MUY_03040	MUY_03040
MUY_03041	MUY_03041
MUY_03041	MUY_03094
MUY_03041	MUY_00310
MUY_03041	MUY_03304
MUY_03041	MUY_00316
MUY_03041	MUY_03706
MUY_03041	MUY_00366
MUY_03041	MUY_04318
MUY_03041	MUY_00582
MUY_03041	MUY_00584
MUY_03041	MUY_00648
MUY_03041	MUY_00072
MUY_03044	MUY_03044
MUY_03050	MUY_03050
MUY_03051	MUY_03267
MUY_00290	MUY_00290
MUY_00290	MUY_03320
MUY_00290	MUY_03640
MUY_00290	MUY_03984
MUY_00290	MUY_04198
MUY_00290	MUY_00401
MUY_00290	MUY_00563
MUY_03052	MUY_03052
MUY_03053	MUY_03053
MUY_03053	MUY_03130
MUY_03053	MUY_03131
MUY_03054	MUY_03054
MUY_03054	MUY_03244
MUY_03054	MUY_03556
MUY_03054	MUY_03573
MUY_03054	MUY_03574
MUY_03054	MUY_03660
MUY_03054	MUY_00766
MUY_03055	MUY_03055
MUY_03055	MUY_04126

MUY_03055	MUY_04239
MUY_03055	MUY_00469
MUY_03055	MUY_00485
MUY_03055	MUY_00696
MUY_03056	MUY_03056
MUY_03056	MUY_03134
MUY_03056	MUY_04385
MUY_03058	MUY_03058
MUY_03058	MUY_03089
MUY_03058	MUY_03267
MUY_03058	MUY_04497
MUY_03058	MUY_04508
MUY_03058	MUY_00804
MUY_03058	MUY_00963
MUY_03059	MUY_03059
MUY_03059	MUY_03183
MUY_03059	MUY_03202
MUY_03059	MUY_03463
MUY_03059	MUY_03477
MUY_03059	MUY_03609
MUY_03059	MUY_03619
MUY_03059	MUY_03648
MUY_03059	MUY_03924
MUY_03059	MUY_04096
MUY_03059	MUY_04146
MUY_03059	MUY_04177
MUY_03059	MUY_04212
MUY_03059	MUY_04293
MUY_03059	MUY_04359
MUY_03059	MUY_04418
MUY_03059	MUY_04448
MUY_03059	MUY_04471
MUY_03059	MUY_00418
MUY_03059	MUY_00436
MUY_03059	MUY_00576
MUY_03060	MUY_03089
MUY_03061	MUY_03061
MUY_03062	MUY_03135
MUY_03062	MUY_03192
MUY_03062	MUY_03476
MUY_03062	MUY_03774
MUY_03062	MUY_03900
MUY_03062	MUY_04205
MUY_03062	MUY_00496
MUY_03062	MUY_00998
MUY_00291	MUY_00291
MUY_03063	MUY_03063

MUY_03064	MUY_03064
MUY_03064	MUY_03544
MUY_03065	MUY_03065
MUY_03066	MUY_03066
MUY_03066	MUY_00449
MUY_03066	MUY_00828
MUY_03067	MUY_03067
MUY_03067	MUY_03267
MUY_03067	MUY_03828
MUY_03067	MUY_04502
MUY_03068	MUY_03068
MUY_00292	MUY_00292
MUY_00292	MUY_03128
MUY_00292	MUY_03225
MUY_00292	MUY_00313
MUY_00292	MUY_03494
MUY_00292	MUY_03606
MUY_00292	MUY_03627
MUY_00292	MUY_03638
MUY_00292	MUY_03964
MUY_00292	MUY_03998
MUY_00292	MUY_04038
MUY_00292	MUY_00486
MUY_00292	MUY_00681
MUY_00292	MUY_00933
MUY_03069	MUY_03496
MUY_03069	MUY_03929
MUY_03069	MUY_03950
MUY_03069	MUY_03952
MUY_03069	MUY_03953
MUY_03069	MUY_04086
MUY_03069	MUY_04334
MUY_03069	MUY_00435
MUY_03069	MUY_00505
MUY_03069	MUY_00780
MUY_03070	MUY_03071
MUY_03070	MUY_03929
MUY_03070	MUY_03950
MUY_03070	MUY_03952
MUY_03070	MUY_03953
MUY_03070	MUY_04334
MUY_03071	MUY_03071
MUY_03071	MUY_03459
MUY_03071	MUY_04116
MUY_03071	MUY_00594
MUY_03073	MUY_03073
MUY_03076	MUY_03076

MUY_03076	MUY_03274
MUY_03076	MUY_03974
MUY_03076	MUY_00525
MUY_03077	MUY_03077
MUY_03077	MUY_00043
MUY_03082	MUY_03082
MUY_03082	MUY_00398
MUY_03082	MUY_00451
MUY_03082	MUY_00807
MUY_03082	MUY_00809
MUY_03082	MUY_00944
MUY_03083	MUY_03083
MUY_03084	MUY_03084
MUY_03085	MUY_03085
MUY_03087	MUY_03087
MUY_03087	MUY_04103
MUY_03089	MUY_03089
MUY_03089	MUY_04497
MUY_03089	MUY_04508
MUY_03089	MUY_00804
MUY_03089	MUY_00963
MUY_03090	MUY_03267
MUY_03090	MUY_00780
MUY_03091	MUY_03091
MUY_03091	MUY_03093
MUY_03091	MUY_00024
MUY_03091	MUY_00036
MUY_03093	MUY_03093
MUY_03093	MUY_03267
MUY_03094	MUY_03094
MUY_03094	MUY_03267
MUY_03094	MUY_03863
MUY_03094	MUY_00366
MUY_03094	MUY_00072
MUY_03095	MUY_03095
MUY_03095	MUY_03863
MUY_03096	MUY_03096
MUY_03096	MUY_03097
MUY_03097	MUY_03097
MUY_03097	MUY_03267
MUY_03098	MUY_03098
MUY_03098	MUY_03205
MUY_03098	MUY_03897
MUY_03099	MUY_03099
MUY_03100	MUY_03100
MUY_03100	MUY_03267
MUY_03100	MUY_03536

MUY_03100	MUY_03833
MUY_03100	MUY_03850
MUY_03100	MUY_04207
MUY_03101	MUY_03101
MUY_03102	MUY_03102
MUY_03102	MUY_03376
MUY_03102	MUY_03983
MUY_03102	MUY_04394
MUY_03102	MUY_00501
MUY_03103	MUY_03103
MUY_03103	MUY_00303
MUY_03103	MUY_00082
MUY_03104	MUY_03104
MUY_03109	MUY_03109
MUY_03109	MUY_00631
MUY_03112	MUY_03112
MUY_03113	MUY_03113
MUY_03113	MUY_03114
MUY_03114	MUY_03114
MUY_00022	MUY_00022
MUY_00022	MUY_03485
MUY_00022	MUY_04035
MUY_03117	MUY_03117
MUY_03117	MUY_03183
MUY_03117	MUY_03202
MUY_03117	MUY_03306
MUY_03117	MUY_03463
MUY_03117	MUY_03477
MUY_03117	MUY_03609
MUY_03117	MUY_03619
MUY_03117	MUY_03648
MUY_03117	MUY_03863
MUY_03117	MUY_03924
MUY_03117	MUY_04096
MUY_03117	MUY_04146
MUY_03117	MUY_04177
MUY_03117	MUY_04212
MUY_03117	MUY_04293
MUY_03117	MUY_04359
MUY_03117	MUY_04418
MUY_03117	MUY_04448
MUY_03117	MUY_04471
MUY_03117	MUY_00418
MUY_03117	MUY_00436
MUY_03117	MUY_00576
MUY_03117	MUY_00790
MUY_03117	MUY_00961

MUY_03121	MUY_03121
MUY_03121	MUY_03418
MUY_03121	MUY_04506
MUY_03122	MUY_03122
MUY_03123	MUY_03123
MUY_03125	MUY_03125
MUY_03126	MUY_03126
MUY_03126	MUY_03240
MUY_03126	MUY_03659
MUY_03126	MUY_03701
MUY_03126	MUY_03880
MUY_03126	MUY_04014
MUY_03126	MUY_04194
MUY_03126	MUY_00556
MUY_03126	MUY_00710
MUY_03127	MUY_03127
MUY_03127	MUY_03183
MUY_03127	MUY_03202
MUY_03127	MUY_03463
MUY_03127	MUY_03477
MUY_03127	MUY_03609
MUY_03127	MUY_03619
MUY_03127	MUY_03648
MUY_03127	MUY_03924
MUY_03127	MUY_04096
MUY_03127	MUY_04146
MUY_03127	MUY_04177
MUY_03127	MUY_04212
MUY_03127	MUY_04293
MUY_03127	MUY_04359
MUY_03127	MUY_00397
MUY_03127	MUY_04418
MUY_03127	MUY_04448
MUY_03127	MUY_04471
MUY_03127	MUY_00418
MUY_03127	MUY_00436
MUY_03127	MUY_00576
MUY_03128	MUY_03128
MUY_03128	MUY_03225
MUY_03128	MUY_00313
MUY_03128	MUY_03494
MUY_03128	MUY_03606
MUY_03128	MUY_03627
MUY_03128	MUY_03638
MUY_03128	MUY_03964
MUY_03128	MUY_03998
MUY_03128	MUY_04038

MUY_03128	MUY_04045
MUY_03128	MUY_00486
MUY_03128	MUY_00681
MUY_03128	MUY_00721
MUY_03128	MUY_00933
MUY_03129	MUY_03129
MUY_03129	MUY_03476
MUY_03130	MUY_03130
MUY_03130	MUY_03131
MUY_03131	MUY_03131
MUY_03131	MUY_03267
MUY_00296	MUY_00296
MUY_03133	MUY_03133
MUY_03134	MUY_03134
MUY_03134	MUY_03267
MUY_03134	MUY_04317
MUY_03134	MUY_04385
MUY_03134	MUY_00439
MUY_03134	MUY_00745
MUY_03135	MUY_03135
MUY_03135	MUY_03503
MUY_03135	MUY_04494
MUY_03135	MUY_00979
MUY_03135	MUY_00998
MUY_03136	MUY_03136
MUY_03136	MUY_03232
MUY_03136	MUY_03267
MUY_03136	MUY_03279
MUY_03136	MUY_03513
MUY_03136	MUY_03515
MUY_03136	MUY_03524
MUY_03136	MUY_03585
MUY_03136	MUY_04073
MUY_03136	MUY_00461
MUY_03136	MUY_00523
MUY_03136	MUY_00861
MUY_03137	MUY_03137
MUY_03137	MUY_03138
MUY_03137	MUY_03513
MUY_03137	MUY_03515
MUY_03137	MUY_03524
MUY_03137	MUY_03590
MUY_03137	MUY_04103
MUY_03137	MUY_04104
MUY_03137	MUY_00461
MUY_03137	MUY_00519
MUY_03138	MUY_03138

MUY_03138	MUY_03513
MUY_03138	MUY_03515
MUY_03138	MUY_03524
MUY_03138	MUY_03590
MUY_03138	MUY_04103
MUY_03138	MUY_04104
MUY_03138	MUY_00461
MUY_03138	MUY_00519
MUY_03139	MUY_03139
MUY_03139	MUY_03183
MUY_03139	MUY_03202
MUY_03139	MUY_03374
MUY_03139	MUY_03463
MUY_03139	MUY_03477
MUY_03139	MUY_03591
MUY_03139	MUY_03592
MUY_03139	MUY_03609
MUY_03139	MUY_03619
MUY_03139	MUY_03648
MUY_03139	MUY_03924
MUY_03139	MUY_04096
MUY_03139	MUY_04105
MUY_03139	MUY_04106
MUY_03139	MUY_04146
MUY_03139	MUY_04177
MUY_03139	MUY_04212
MUY_03139	MUY_04293
MUY_03139	MUY_04359
MUY_03139	MUY_04418
MUY_03139	MUY_04448
MUY_03139	MUY_04471
MUY_03139	MUY_00418
MUY_03139	MUY_00436
MUY_03139	MUY_00576
MUY_03140	MUY_03140
MUY_03140	MUY_03250
MUY_03140	MUY_03431
MUY_03140	MUY_00323
MUY_03140	MUY_03553
MUY_03140	MUY_03612
MUY_03140	MUY_00445
MUY_03140	MUY_00677
MUY_03140	MUY_00749
MUY_03140	MUY_00779
MUY_03140	MUY_00882
MUY_03141	MUY_03141
MUY_03141	MUY_03241

MUY_03141	MUY_03261
MUY_03141	MUY_03373
MUY_03141	MUY_00376
MUY_03141	MUY_04033
MUY_03141	MUY_04034
MUY_03141	MUY_04036
MUY_03141	MUY_00377
MUY_03141	MUY_00378
MUY_03141	MUY_04228
MUY_03141	MUY_04230
MUY_03141	MUY_00379
MUY_00297	MUY_00297
MUY_00297	MUY_03518
MUY_00297	MUY_03667
MUY_00297	MUY_04463
MUY_00297	MUY_00557
MUY_00297	MUY_00583
MUY_00297	MUY_00674
MUY_03144	MUY_03144
MUY_03144	MUY_03216
MUY_03144	MUY_03249
MUY_03144	MUY_03989
MUY_03146	MUY_03146
MUY_03146	MUY_03248
MUY_00298	MUY_00298
MUY_00298	MUY_03702
MUY_00298	MUY_00774
MUY_00298	MUY_00775
MUY_03149	MUY_03149
MUY_03149	MUY_03246
MUY_03149	MUY_03791
MUY_03149	MUY_04098
MUY_03149	MUY_04304
MUY_03150	MUY_03150
MUY_03150	MUY_03247
MUY_03150	MUY_03790
MUY_03150	MUY_04099
MUY_03150	MUY_04305
MUY_03150	MUY_00630
MUY_03154	MUY_03154
MUY_03154	MUY_03330
MUY_03154	MUY_03332
MUY_03154	MUY_03354
MUY_03154	MUY_00322
MUY_03154	MUY_03575
MUY_03154	MUY_03576
MUY_03154	MUY_03582

MUY_03154	MUY_03598
MUY_03154	MUY_03634
MUY_03154	MUY_03649
MUY_03154	MUY_03676
MUY_03154	MUY_03785
MUY_03154	MUY_03805
MUY_03154	MUY_03900
MUY_03154	MUY_03946
MUY_03154	MUY_00372
MUY_03154	MUY_03978
MUY_03154	MUY_04144
MUY_03154	MUY_04172
MUY_03154	MUY_04248
MUY_03154	MUY_04254
MUY_03154	MUY_04266
MUY_03154	MUY_04269
MUY_03154	MUY_04282
MUY_03154	MUY_04296
MUY_03154	MUY_00388
MUY_03154	MUY_04348
MUY_03154	MUY_04357
MUY_03154	MUY_00394
MUY_03154	MUY_04403
MUY_03154	MUY_04416
MUY_03154	MUY_04445
MUY_03154	MUY_04453
MUY_03154	MUY_00420
MUY_03154	MUY_00434
MUY_03154	MUY_00442
MUY_03154	MUY_00516
MUY_03154	MUY_00585
MUY_03154	MUY_00800
MUY_03154	MUY_00815
MUY_03154	MUY_00937
MUY_03154	MUY_00938
MUY_03154	MUY_00939
MUY_03154	MUY_00968
MUY_03154	MUY_00975
MUY_03154	MUY_00996
MUY_03154	MUY_01000
MUY_03157	MUY_03157
MUY_03157	MUY_03267
MUY_03157	MUY_03277
MUY_03158	MUY_03158
MUY_03160	MUY_03160
MUY_03160	MUY_00983
MUY_03168	MUY_03168

MUY_03168	MUY_03399
MUY_03168	MUY_04405
MUY_03169	MUY_03169
MUY_03169	MUY_03694
MUY_03169	MUY_00056
MUY_03170	MUY_03170
MUY_03170	MUY_00344
MUY_03170	MUY_03691
MUY_03170	MUY_04192
MUY_03170	MUY_04434
MUY_03171	MUY_03171
MUY_03173	MUY_03173
MUY_03173	MUY_03557
MUY_03179	MUY_03267
MUY_03182	MUY_03182
MUY_00023	MUY_00023
MUY_03183	MUY_03183
MUY_03183	MUY_03201
MUY_03183	MUY_03202
MUY_03183	MUY_03252
MUY_03183	MUY_03306
MUY_03183	MUY_03374
MUY_03183	MUY_03463
MUY_03183	MUY_03477
MUY_03183	MUY_03478
MUY_03183	MUY_03483
MUY_03183	MUY_03609
MUY_03183	MUY_03619
MUY_03183	MUY_03647
MUY_03183	MUY_03648
MUY_03183	MUY_03924
MUY_03183	MUY_04096
MUY_03183	MUY_04146
MUY_03183	MUY_04177
MUY_03183	MUY_04211
MUY_03183	MUY_04212
MUY_03183	MUY_04293
MUY_03183	MUY_04294
MUY_03183	MUY_04358
MUY_03183	MUY_04359
MUY_03183	MUY_04379
MUY_03183	MUY_04417
MUY_03183	MUY_04418
MUY_03183	MUY_04447
MUY_03183	MUY_04448
MUY_03183	MUY_04471
MUY_03183	MUY_00418

MUY_03183	MUY_00436
MUY_03183	MUY_00575
MUY_03183	MUY_00576
MUY_03183	MUY_00790
MUY_03183	MUY_00961
MUY_03186	MUY_03186
MUY_03186	MUY_03876
MUY_03186	MUY_04337
MUY_03186	MUY_00708
MUY_03187	MUY_03267
MUY_03190	MUY_03190
MUY_03190	MUY_00005
MUY_03190	MUY_04334
MUY_03190	MUY_04476
MUY_03190	MUY_00610
MUY_00301	MUY_00301
MUY_03191	MUY_03191
MUY_03191	MUY_03425
MUY_03191	MUY_00609
MUY_03194	MUY_03194
MUY_03195	MUY_03195
MUY_03195	MUY_03800
MUY_03196	MUY_03196
MUY_03196	MUY_00907
MUY_03196	MUY_00908
MUY_03197	MUY_03197
MUY_03199	MUY_03199
MUY_03199	MUY_04007
MUY_03199	MUY_04401
MUY_03200	MUY_03200
MUY_03200	MUY_03216
MUY_03200	MUY_00036
MUY_03201	MUY_03201
MUY_03201	MUY_03202
MUY_03201	MUY_03463
MUY_03201	MUY_03477
MUY_03201	MUY_03609
MUY_03201	MUY_03619
MUY_03201	MUY_03647
MUY_03201	MUY_03648
MUY_03201	MUY_03924
MUY_03201	MUY_04096
MUY_03201	MUY_04146
MUY_03201	MUY_04177
MUY_03201	MUY_04212
MUY_03201	MUY_04293
MUY_03201	MUY_04359

MUY_03201	MUY_04417
MUY_03201	MUY_04418
MUY_03201	MUY_04448
MUY_03201	MUY_04471
MUY_03201	MUY_00418
MUY_03201	MUY_00436
MUY_03201	MUY_00575
MUY_03201	MUY_00576
MUY_03202	MUY_03202
MUY_03202	MUY_03252
MUY_03202	MUY_03306
MUY_03202	MUY_03374
MUY_03202	MUY_03463
MUY_03202	MUY_03477
MUY_03202	MUY_03478
MUY_03202	MUY_03483
MUY_03202	MUY_03609
MUY_03202	MUY_03619
MUY_03202	MUY_03647
MUY_03202	MUY_03648
MUY_03202	MUY_03924
MUY_03202	MUY_04096
MUY_03202	MUY_04146
MUY_03202	MUY_04177
MUY_03202	MUY_04211
MUY_03202	MUY_04212
MUY_03202	MUY_04293
MUY_03202	MUY_04294
MUY_03202	MUY_04358
MUY_03202	MUY_04359
MUY_03202	MUY_04379
MUY_03202	MUY_04417
MUY_03202	MUY_04418
MUY_03202	MUY_04447
MUY_03202	MUY_04448
MUY_03202	MUY_04471
MUY_03202	MUY_00418
MUY_03202	MUY_00436
MUY_03202	MUY_00575
MUY_03202	MUY_00576
MUY_03202	MUY_00790
MUY_03202	MUY_00961
MUY_03204	MUY_03204
MUY_03204	MUY_03267
MUY_03204	MUY_00340
MUY_03205	MUY_03205
MUY_03205	MUY_03267

MUY_03205	MUY_03897
MUY_03206	MUY_03206
MUY_03206	MUY_03267
MUY_03206	MUY_04243
MUY_03210	MUY_03267
MUY_03211	MUY_03267
MUY_03213	MUY_03213
MUY_03213	MUY_03214
MUY_03214	MUY_03214
MUY_03215	MUY_03215
MUY_03215	MUY_03267
MUY_03215	MUY_04287
MUY_03216	MUY_03216
MUY_03216	MUY_03249
MUY_03216	MUY_03296
MUY_03216	MUY_03989
MUY_03216	MUY_00008
MUY_03216	MUY_00782
MUY_00303	MUY_00303
MUY_00303	MUY_03901
MUY_03220	MUY_03220
MUY_03220	MUY_04017
MUY_03222	MUY_03222
MUY_03222	MUY_03263
MUY_03222	MUY_03434
MUY_03222	MUY_04020
MUY_03222	MUY_04022
MUY_03223	MUY_03223
MUY_03223	MUY_00326
MUY_03223	MUY_04247
MUY_03225	MUY_03225
MUY_03225	MUY_00313
MUY_03225	MUY_03494
MUY_03225	MUY_03606
MUY_03225	MUY_03627
MUY_03225	MUY_03638
MUY_03225	MUY_03964
MUY_03225	MUY_03998
MUY_03225	MUY_04038
MUY_03225	MUY_00486
MUY_03225	MUY_00681
MUY_03225	MUY_00933
MUY_03227	MUY_03227
MUY_03227	MUY_03425
MUY_03227	MUY_03613
MUY_03227	MUY_04289
MUY_03227	MUY_00507

MUY_03227	MUY_00738
MUY_03228	MUY_03228
MUY_03228	MUY_03267
MUY_03229	MUY_03229
MUY_03230	MUY_03230
MUY_03230	MUY_03267
MUY_03231	MUY_03231
MUY_03232	MUY_03232
MUY_03232	MUY_03267
MUY_03232	MUY_03279
MUY_03232	MUY_03585
MUY_03232	MUY_00523
MUY_00304	MUY_03683
MUY_00304	MUY_00483
MUY_03237	MUY_03237
MUY_03238	MUY_03238
MUY_03238	MUY_00991
MUY_03240	MUY_03240
MUY_03240	MUY_03659
MUY_03240	MUY_03701
MUY_03240	MUY_03880
MUY_03240	MUY_04014
MUY_03240	MUY_04194
MUY_03240	MUY_00556
MUY_03240	MUY_00710
MUY_03241	MUY_03241
MUY_03241	MUY_03261
MUY_03241	MUY_03267
MUY_03241	MUY_03373
MUY_03241	MUY_00376
MUY_03241	MUY_04033
MUY_03241	MUY_04034
MUY_03241	MUY_04036
MUY_03241	MUY_00377
MUY_03241	MUY_00378
MUY_03241	MUY_04228
MUY_03241	MUY_04230
MUY_03241	MUY_00379
MUY_03242	MUY_03242
MUY_03242	MUY_00050
MUY_03243	MUY_03243
MUY_03244	MUY_03244
MUY_03244	MUY_03556
MUY_03244	MUY_03573
MUY_03244	MUY_03574
MUY_03244	MUY_03660
MUY_03244	MUY_00766

MUY_03246	MUY_03246
MUY_03246	MUY_03791
MUY_03246	MUY_04098
MUY_03246	MUY_04304
MUY_03247	MUY_03247
MUY_03247	MUY_03790
MUY_03247	MUY_04099
MUY_03247	MUY_04305
MUY_03247	MUY_00630
MUY_03249	MUY_03249
MUY_03249	MUY_03989
MUY_03250	MUY_03250
MUY_03250	MUY_03431
MUY_03250	MUY_00323
MUY_03250	MUY_03553
MUY_03250	MUY_03612
MUY_03250	MUY_00445
MUY_03250	MUY_00677
MUY_03250	MUY_00749
MUY_03250	MUY_00779
MUY_03250	MUY_00882
MUY_03251	MUY_03251
MUY_03251	MUY_03847
MUY_03251	MUY_04122
MUY_03251	MUY_04219
MUY_03251	MUY_04402
MUY_03252	MUY_03252
MUY_03252	MUY_03463
MUY_03252	MUY_03477
MUY_03252	MUY_03483
MUY_03252	MUY_03609
MUY_03252	MUY_03619
MUY_03252	MUY_03648
MUY_03252	MUY_03924
MUY_03252	MUY_04096
MUY_03252	MUY_04146
MUY_03252	MUY_04177
MUY_03252	MUY_04212
MUY_03252	MUY_04293
MUY_03252	MUY_04359
MUY_03252	MUY_04379
MUY_03252	MUY_04418
MUY_03252	MUY_04448
MUY_03252	MUY_04471
MUY_03252	MUY_00418
MUY_03252	MUY_00436
MUY_03252	MUY_00576

MUY_03253	MUY_03253
MUY_03253	MUY_03267
MUY_03253	MUY_03299
MUY_03253	MUY_00065
MUY_00307	MUY_00307
MUY_00307	MUY_04235
MUY_00307	MUY_00899
MUY_03257	MUY_03257
MUY_03258	MUY_03258
MUY_03258	MUY_03986
MUY_03258	MUY_04376
MUY_03258	MUY_00495
MUY_03260	MUY_03260
MUY_03260	MUY_00709
MUY_03261	MUY_03261
MUY_03261	MUY_03373
MUY_03261	MUY_00376
MUY_03261	MUY_04033
MUY_03261	MUY_04034
MUY_03261	MUY_04036
MUY_03261	MUY_00377
MUY_03261	MUY_00378
MUY_03261	MUY_04228
MUY_03261	MUY_04230
MUY_03261	MUY_00379
MUY_03263	MUY_03263
MUY_03263	MUY_03292
MUY_03263	MUY_03434
MUY_03263	MUY_04020
MUY_03263	MUY_04022
MUY_03263	MUY_00038
MUY_03264	MUY_03264
MUY_03267	MUY_03267
MUY_03267	MUY_03323
MUY_03267	MUY_03345
MUY_03267	MUY_03347
MUY_03267	MUY_03367
MUY_03267	MUY_03443
MUY_03267	MUY_03446
MUY_03267	MUY_03484
MUY_03267	MUY_00327
MUY_03267	MUY_00328
MUY_03267	MUY_00329
MUY_03267	MUY_03515
MUY_03267	MUY_03534
MUY_03267	MUY_00334
MUY_03267	MUY_03572

MUY_03267	MUY_03574
MUY_03267	MUY_03575
MUY_03267	MUY_03576
MUY_03267	MUY_03582
MUY_03267	MUY_03613
MUY_03267	MUY_03644
MUY_03267	MUY_03658
MUY_03267	MUY_03795
MUY_03267	MUY_03796
MUY_03267	MUY_03800
MUY_03267	MUY_03807
MUY_03267	MUY_03808
MUY_03267	MUY_03890
MUY_03267	MUY_03904
MUY_03267	MUY_03905
MUY_03267	MUY_03911
MUY_03267	MUY_03945
MUY_03267	MUY_03962
MUY_03267	MUY_03975
MUY_03267	MUY_00376
MUY_03267	MUY_04017
MUY_03267	MUY_04034
MUY_03267	MUY_04035
MUY_03267	MUY_04036
MUY_03267	MUY_04037
MUY_03267	MUY_04038
MUY_03267	MUY_04061
MUY_03267	MUY_04063
MUY_03267	MUY_04070
MUY_03267	MUY_04082
MUY_03267	MUY_04094
MUY_03267	MUY_04095
MUY_03267	MUY_04110
MUY_03267	MUY_00377
MUY_03267	MUY_04183
MUY_03267	MUY_04228
MUY_03267	MUY_00379
MUY_03267	MUY_04292
MUY_03267	MUY_04328
MUY_03267	MUY_04332
MUY_03267	MUY_04428
MUY_03267	MUY_04439
MUY_03267	MUY_04440
MUY_03267	MUY_04474
MUY_03267	MUY_04497
MUY_03267	MUY_04507
MUY_03267	MUY_00043

MUY_03267	MUY_00527
MUY_03267	MUY_00055
MUY_03267	MUY_00057
MUY_03267	MUY_00684
MUY_03267	MUY_00687
MUY_03267	MUY_00700
MUY_03267	MUY_00008
MUY_03267	MUY_00730
MUY_03267	MUY_00736
MUY_03267	MUY_00738
MUY_03267	MUY_00739
MUY_03267	MUY_00742
MUY_03267	MUY_00743
MUY_03267	MUY_00746
MUY_03267	MUY_00774
MUY_03267	MUY_00775
MUY_03267	MUY_00797
MUY_03267	MUY_00802
MUY_03267	MUY_00805
MUY_03267	MUY_00807
MUY_03267	MUY_00810
MUY_03267	MUY_00075
MUY_03267	MUY_00079
MUY_03267	MUY_00088
MUY_03267	MUY_00933
MUY_03268	MUY_03268
MUY_03274	MUY_03274
MUY_03274	MUY_03974
MUY_03274	MUY_00525
MUY_00308	MUY_00308
MUY_00308	MUY_03333
MUY_00308	MUY_03386
MUY_00308	MUY_03449
MUY_00308	MUY_03454
MUY_00308	MUY_03637
MUY_00308	MUY_03810
MUY_00308	MUY_04070
MUY_00308	MUY_04242
MUY_00308	MUY_00382
MUY_00308	MUY_04302
MUY_00308	MUY_04383
MUY_00308	MUY_04433
MUY_00308	MUY_04483
MUY_00308	MUY_00450
MUY_00308	MUY_00464
MUY_00308	MUY_00808
MUY_00308	MUY_00840

MUY_03276	MUY_03276
MUY_03277	MUY_03277
MUY_03279	MUY_03279
MUY_03279	MUY_03513
MUY_03279	MUY_03515
MUY_03279	MUY_03524
MUY_03279	MUY_03585
MUY_03279	MUY_04073
MUY_03279	MUY_00461
MUY_03279	MUY_00523
MUY_03279	MUY_00861
MUY_03281	MUY_03281
MUY_00309	MUY_00309
MUY_00309	MUY_03785
MUY_03282	MUY_03282
MUY_00024	MUY_00024
MUY_00024	MUY_04476
MUY_00024	MUY_00036
MUY_03284	MUY_03284
MUY_03284	MUY_03304
MUY_03284	MUY_00316
MUY_03284	MUY_03706
MUY_03284	MUY_04467
MUY_03284	MUY_00824
MUY_03285	MUY_03285
MUY_03287	MUY_03287
MUY_03288	MUY_03288
MUY_03288	MUY_03384
MUY_03288	MUY_03385
MUY_03289	MUY_03289
MUY_03289	MUY_00784
MUY_03291	MUY_03291
MUY_03292	MUY_03292
MUY_03292	MUY_03434
MUY_03292	MUY_04020
MUY_03292	MUY_04022
MUY_00310	MUY_00310
MUY_00310	MUY_00366
MUY_00310	MUY_00648
MUY_00310	MUY_00072
MUY_03296	MUY_03496
MUY_03296	MUY_04334
MUY_03296	MUY_00435
MUY_03296	MUY_00590
MUY_03296	MUY_00780
MUY_03299	MUY_03299
MUY_03299	MUY_00065

MUY_03302	MUY_03302
MUY_03304	MUY_03304
MUY_03304	MUY_00316
MUY_03304	MUY_03513
MUY_03304	MUY_03515
MUY_03304	MUY_03524
MUY_03304	MUY_03706
MUY_03304	MUY_00366
MUY_03304	MUY_04467
MUY_03304	MUY_00461
MUY_03304	MUY_00072
MUY_03304	MUY_00824
MUY_03306	MUY_03306
MUY_03306	MUY_03463
MUY_03306	MUY_03477
MUY_03306	MUY_03609
MUY_03306	MUY_03619
MUY_03306	MUY_03648
MUY_03306	MUY_03863
MUY_03306	MUY_03924
MUY_03306	MUY_04096
MUY_03306	MUY_04146
MUY_03306	MUY_04177
MUY_03306	MUY_04212
MUY_03306	MUY_04293
MUY_03306	MUY_04359
MUY_03306	MUY_04418
MUY_03306	MUY_04448
MUY_03306	MUY_04471
MUY_03306	MUY_00418
MUY_03306	MUY_00436
MUY_03306	MUY_00576
MUY_03306	MUY_00790
MUY_03306	MUY_00961
MUY_03310	MUY_03310
MUY_03310	MUY_03422
MUY_03310	MUY_03703
MUY_03310	MUY_04079
MUY_03310	MUY_04391
MUY_03310	MUY_00852
MUY_03314	MUY_03314
MUY_03314	MUY_03496
MUY_03314	MUY_03980
MUY_03314	MUY_04043
MUY_03314	MUY_04086
MUY_03314	MUY_00385
MUY_03314	MUY_04334

MUY_03314	MUY_04343
MUY_03314	MUY_00435
MUY_03314	MUY_00589
MUY_03314	MUY_00722
MUY_03314	MUY_00780
MUY_03317	MUY_03317
MUY_03317	MUY_03934
MUY_03317	MUY_04183
MUY_03317	MUY_04335
MUY_03317	MUY_04430
MUY_03317	MUY_00847
MUY_03317	MUY_00921
MUY_03318	MUY_03318
MUY_03318	MUY_03491
MUY_03318	MUY_00854
MUY_03320	MUY_03320
MUY_03320	MUY_03640
MUY_03320	MUY_03984
MUY_03320	MUY_04198
MUY_03320	MUY_00401
MUY_03320	MUY_00563
MUY_03330	MUY_03330
MUY_03330	MUY_03332
MUY_03330	MUY_03354
MUY_03330	MUY_00322
MUY_03330	MUY_03575
MUY_03330	MUY_03576
MUY_03330	MUY_03582
MUY_03330	MUY_03598
MUY_03330	MUY_03634
MUY_03330	MUY_03649
MUY_03330	MUY_03676
MUY_03330	MUY_03785
MUY_03330	MUY_03805
MUY_03330	MUY_03900
MUY_03330	MUY_03946
MUY_03330	MUY_00372
MUY_03330	MUY_03978
MUY_03330	MUY_04144
MUY_03330	MUY_04172
MUY_03330	MUY_04248
MUY_03330	MUY_04254
MUY_03330	MUY_04266
MUY_03330	MUY_04269
MUY_03330	MUY_04282
MUY_03330	MUY_04296
MUY_03330	MUY_00388

MUY_03330	MUY_04348
MUY_03330	MUY_04357
MUY_03330	MUY_00394
MUY_03330	MUY_04403
MUY_03330	MUY_04416
MUY_03330	MUY_04445
MUY_03330	MUY_04453
MUY_03330	MUY_00420
MUY_03330	MUY_00434
MUY_03330	MUY_00442
MUY_03330	MUY_00516
MUY_03330	MUY_00585
MUY_03330	MUY_00800
MUY_03330	MUY_00815
MUY_03330	MUY_00937
MUY_03330	MUY_00938
MUY_03330	MUY_00939
MUY_03330	MUY_00968
MUY_03330	MUY_00975
MUY_03330	MUY_00996
MUY_03330	MUY_01000
MUY_03332	MUY_03332
MUY_03332	MUY_03354
MUY_03332	MUY_00322
MUY_03332	MUY_03575
MUY_03332	MUY_03576
MUY_03332	MUY_03582
MUY_03332	MUY_03598
MUY_03332	MUY_03634
MUY_03332	MUY_03649
MUY_03332	MUY_03676
MUY_03332	MUY_03785
MUY_03332	MUY_03805
MUY_03332	MUY_03900
MUY_03332	MUY_03946
MUY_03332	MUY_00372
MUY_03332	MUY_03978
MUY_03332	MUY_04144
MUY_03332	MUY_04172
MUY_03332	MUY_04248
MUY_03332	MUY_04254
MUY_03332	MUY_04266
MUY_03332	MUY_04269
MUY_03332	MUY_04282
MUY_03332	MUY_04296
MUY_03332	MUY_00388
MUY_03332	MUY_04348

MUY_03332	MUY_04357
MUY_03332	MUY_00394
MUY_03332	MUY_04403
MUY_03332	MUY_04416
MUY_03332	MUY_04445
MUY_03332	MUY_04453
MUY_03332	MUY_00420
MUY_03332	MUY_00434
MUY_03332	MUY_00442
MUY_03332	MUY_00516
MUY_03332	MUY_00585
MUY_03332	MUY_00800
MUY_03332	MUY_00815
MUY_03332	MUY_00937
MUY_03332	MUY_00938
MUY_03332	MUY_00939
MUY_03332	MUY_00968
MUY_03332	MUY_00975
MUY_03332	MUY_00996
MUY_03332	MUY_01000
MUY_03333	MUY_03333
MUY_03333	MUY_03386
MUY_03333	MUY_03449
MUY_03333	MUY_03454
MUY_03333	MUY_03637
MUY_03333	MUY_03810
MUY_03333	MUY_04070
MUY_03333	MUY_04242
MUY_03333	MUY_00382
MUY_03333	MUY_04302
MUY_03333	MUY_04383
MUY_03333	MUY_04433
MUY_03333	MUY_04483
MUY_03333	MUY_00450
MUY_03333	MUY_00464
MUY_03333	MUY_00808
MUY_03333	MUY_00840
MUY_03337	MUY_00344
MUY_03338	MUY_03338
MUY_00312	MUY_00312
MUY_00312	MUY_03608
MUY_00312	MUY_03896
MUY_00312	MUY_04466
MUY_03341	MUY_03341
MUY_03341	MUY_03375
MUY_03341	MUY_03451
MUY_03341	MUY_03775

MUY_03341	MUY_00378
MUY_03341	MUY_04230
MUY_03341	MUY_00526
MUY_03341	MUY_00857
MUY_03342	MUY_03342
MUY_03342	MUY_03382
MUY_03342	MUY_03383
MUY_03342	MUY_03945
MUY_03342	MUY_04196
MUY_03342	MUY_00056
MUY_03344	MUY_03344
MUY_03344	MUY_03949
MUY_03344	MUY_04150
MUY_03344	MUY_00849
MUY_03345	MUY_03345
MUY_03345	MUY_04174
MUY_03345	MUY_04476
MUY_03345	MUY_00074
MUY_03347	MUY_03347
MUY_03349	MUY_03349
MUY_00313	MUY_00313
MUY_00313	MUY_03494
MUY_00313	MUY_03606
MUY_00313	MUY_03627
MUY_00313	MUY_03638
MUY_00313	MUY_03964
MUY_00313	MUY_03998
MUY_00313	MUY_04038
MUY_00313	MUY_04045
MUY_00313	MUY_00486
MUY_00313	MUY_00681
MUY_00313	MUY_00721
MUY_00313	MUY_00933
MUY_03351	MUY_03351
MUY_03352	MUY_03352
MUY_03353	MUY_03353
MUY_03354	MUY_03354
MUY_03354	MUY_00322
MUY_03354	MUY_03575
MUY_03354	MUY_03576
MUY_03354	MUY_03582
MUY_03354	MUY_03598
MUY_03354	MUY_03634
MUY_03354	MUY_03649
MUY_03354	MUY_03676
MUY_03354	MUY_03785
MUY_03354	MUY_03805

MUY_03354	MUY_03900
MUY_03354	MUY_03946
MUY_03354	MUY_00372
MUY_03354	MUY_03978
MUY_03354	MUY_04144
MUY_03354	MUY_04172
MUY_03354	MUY_04248
MUY_03354	MUY_04254
MUY_03354	MUY_04266
MUY_03354	MUY_04269
MUY_03354	MUY_04282
MUY_03354	MUY_04296
MUY_03354	MUY_00388
MUY_03354	MUY_04348
MUY_03354	MUY_04357
MUY_03354	MUY_00394
MUY_03354	MUY_04403
MUY_03354	MUY_04416
MUY_03354	MUY_04445
MUY_03354	MUY_04453
MUY_03354	MUY_00420
MUY_03354	MUY_00434
MUY_03354	MUY_00442
MUY_03354	MUY_00516
MUY_03354	MUY_00585
MUY_03354	MUY_00800
MUY_03354	MUY_00815
MUY_03354	MUY_00937
MUY_03354	MUY_00938
MUY_03354	MUY_00939
MUY_03354	MUY_00968
MUY_03354	MUY_00975
MUY_03354	MUY_00996
MUY_03354	MUY_01000
MUY_03356	MUY_03356
MUY_03356	MUY_03860
MUY_03356	MUY_03951
MUY_03356	MUY_04473
MUY_03356	MUY_00912
MUY_03362	MUY_03362
MUY_03364	MUY_03364
MUY_03364	MUY_03849
MUY_03364	MUY_00888
MUY_03365	MUY_03365
MUY_03365	MUY_00805
MUY_03367	MUY_03367
MUY_03367	MUY_04090

MUY_03367	MUY_04486
MUY_03372	MUY_03372
MUY_03372	MUY_04216
MUY_03373	MUY_03373
MUY_03373	MUY_00376
MUY_03373	MUY_04033
MUY_03373	MUY_04034
MUY_03373	MUY_04036
MUY_03373	MUY_00377
MUY_03373	MUY_00378
MUY_03373	MUY_04228
MUY_03373	MUY_04230
MUY_03373	MUY_00379
MUY_03374	MUY_03374
MUY_03374	MUY_03463
MUY_03374	MUY_03477
MUY_03374	MUY_03591
MUY_03374	MUY_03592
MUY_03374	MUY_03609
MUY_03374	MUY_03619
MUY_03374	MUY_03648
MUY_03374	MUY_00344
MUY_03374	MUY_03924
MUY_03374	MUY_04096
MUY_03374	MUY_04105
MUY_03374	MUY_04106
MUY_03374	MUY_04146
MUY_03374	MUY_04177
MUY_03374	MUY_04212
MUY_03374	MUY_04293
MUY_03374	MUY_04359
MUY_03374	MUY_04418
MUY_03374	MUY_04448
MUY_03374	MUY_04471
MUY_03374	MUY_00418
MUY_03374	MUY_00436
MUY_03374	MUY_00576
MUY_03374	MUY_00979
MUY_03375	MUY_03375
MUY_03375	MUY_03451
MUY_03375	MUY_03775
MUY_03375	MUY_00378
MUY_03375	MUY_04230
MUY_03375	MUY_00526
MUY_03375	MUY_00857
MUY_03376	MUY_03376
MUY_03376	MUY_03983

MUY_03376	MUY_04394
MUY_03376	MUY_04395
MUY_03376	MUY_00501
MUY_03379	MUY_03379
MUY_03381	MUY_03381
MUY_03381	MUY_03949
MUY_03381	MUY_00849
MUY_03382	MUY_03382
MUY_03382	MUY_03383
MUY_03382	MUY_03945
MUY_03382	MUY_00056
MUY_03383	MUY_03383
MUY_03383	MUY_03945
MUY_03383	MUY_00056
MUY_03384	MUY_03384
MUY_03384	MUY_03385
MUY_03385	MUY_03385
MUY_00316	MUY_00316
MUY_00316	MUY_03513
MUY_00316	MUY_03515
MUY_00316	MUY_03524
MUY_00316	MUY_03706
MUY_00316	MUY_00366
MUY_00316	MUY_04467
MUY_00316	MUY_00461
MUY_00316	MUY_00072
MUY_00316	MUY_00824
MUY_03386	MUY_03386
MUY_03386	MUY_03449
MUY_03386	MUY_03454
MUY_03386	MUY_03637
MUY_03386	MUY_03810
MUY_03386	MUY_04070
MUY_03386	MUY_04242
MUY_03386	MUY_00382
MUY_03386	MUY_04302
MUY_03386	MUY_04383
MUY_03386	MUY_04433
MUY_03386	MUY_04483
MUY_03386	MUY_00450
MUY_03386	MUY_00464
MUY_03386	MUY_00808
MUY_03386	MUY_00840
MUY_00317	MUY_00317
MUY_00317	MUY_03496
MUY_00317	MUY_00780
MUY_03388	MUY_03388

MUY_03388	MUY_00578
MUY_03392	MUY_03392
MUY_03393	MUY_03393
MUY_03393	MUY_03438
MUY_00318	MUY_00318
MUY_00318	MUY_03802
MUY_00318	MUY_04161
MUY_00318	MUY_00688
MUY_00318	MUY_00971
MUY_03398	MUY_03398
MUY_03398	MUY_03639
MUY_03398	MUY_00830
MUY_03399	MUY_03399
MUY_03399	MUY_04405
MUY_03400	MUY_03400
MUY_03400	MUY_00347
MUY_03400	MUY_04132
MUY_03400	MUY_04150
MUY_03400	MUY_04397
MUY_03400	MUY_04438
MUY_03400	MUY_00453
MUY_03400	MUY_00797
MUY_03403	MUY_03403
MUY_03403	MUY_00857
MUY_03404	MUY_03404
MUY_03404	MUY_00532
MUY_03404	MUY_00768
MUY_03405	MUY_03405
MUY_03405	MUY_03710
MUY_00320	MUY_00320
MUY_00320	MUY_03970
MUY_03406	MUY_03406
MUY_03411	MUY_03411
MUY_03415	MUY_03415
MUY_03418	MUY_03418
MUY_03422	MUY_03422
MUY_03422	MUY_03703
MUY_03422	MUY_04079
MUY_03422	MUY_04391
MUY_03422	MUY_00852
MUY_03423	MUY_03423
MUY_03423	MUY_03689
MUY_03423	MUY_03794
MUY_03423	MUY_04260
MUY_03423	MUY_04262
MUY_03423	MUY_00892
MUY_03424	MUY_03424

MUY_03424	MUY_03426
MUY_03425	MUY_03425
MUY_03425	MUY_03496
MUY_03425	MUY_03613
MUY_03425	MUY_03656
MUY_03425	MUY_00367
MUY_03425	MUY_03896
MUY_03425	MUY_03925
MUY_03425	MUY_04043
MUY_03425	MUY_04289
MUY_03425	MUY_00038
MUY_03425	MUY_00501
MUY_03425	MUY_00008
MUY_03425	MUY_00738
MUY_03426	MUY_03426
MUY_03428	MUY_03428
MUY_00322	MUY_00322
MUY_00322	MUY_03575
MUY_00322	MUY_03576
MUY_00322	MUY_03582
MUY_00322	MUY_03598
MUY_00322	MUY_03634
MUY_00322	MUY_03649
MUY_00322	MUY_03676
MUY_00322	MUY_03785
MUY_00322	MUY_03805
MUY_00322	MUY_03900
MUY_00322	MUY_03946
MUY_00322	MUY_00372
MUY_00322	MUY_03978
MUY_00322	MUY_04144
MUY_00322	MUY_04172
MUY_00322	MUY_04248
MUY_00322	MUY_04254
MUY_00322	MUY_04266
MUY_00322	MUY_04269
MUY_00322	MUY_04282
MUY_00322	MUY_04296
MUY_00322	MUY_00388
MUY_00322	MUY_04348
MUY_00322	MUY_04357
MUY_00322	MUY_00394
MUY_00322	MUY_04403
MUY_00322	MUY_04416
MUY_00322	MUY_04445
MUY_00322	MUY_04453
MUY_00322	MUY_00420

MUY_00322	MUY_00434
MUY_00322	MUY_00442
MUY_00322	MUY_00516
MUY_00322	MUY_00585
MUY_00322	MUY_00800
MUY_00322	MUY_00815
MUY_00322	MUY_00937
MUY_00322	MUY_00938
MUY_00322	MUY_00939
MUY_00322	MUY_00968
MUY_00322	MUY_00975
MUY_00322	MUY_00996
MUY_00322	MUY_01000
MUY_03429	MUY_03429
MUY_03431	MUY_03431
MUY_03431	MUY_00323
MUY_03431	MUY_03553
MUY_03431	MUY_03612
MUY_03431	MUY_00445
MUY_03431	MUY_00677
MUY_03431	MUY_00749
MUY_03431	MUY_00779
MUY_03431	MUY_00882
MUY_03434	MUY_03434
MUY_03434	MUY_04020
MUY_03434	MUY_04022
MUY_03438	MUY_03438
MUY_03439	MUY_03439
MUY_03440	MUY_03440
MUY_00323	MUY_00323
MUY_00323	MUY_03553
MUY_00323	MUY_03612
MUY_00323	MUY_00445
MUY_00323	MUY_00677
MUY_00323	MUY_00749
MUY_00323	MUY_00779
MUY_00323	MUY_00882
MUY_03442	MUY_03442
MUY_03442	MUY_04171
MUY_03442	MUY_04318
MUY_03442	MUY_04410
MUY_03442	MUY_00504
MUY_03442	MUY_00782
MUY_03442	MUY_00920
MUY_03443	MUY_03443
MUY_03446	MUY_03446
MUY_03446	MUY_03632

MUY_03446	MUY_03836
MUY_03446	MUY_00872
MUY_03449	MUY_03449
MUY_03449	MUY_03454
MUY_03449	MUY_03637
MUY_03449	MUY_03810
MUY_03449	MUY_04070
MUY_03449	MUY_04242
MUY_03449	MUY_00382
MUY_03449	MUY_04302
MUY_03449	MUY_04383
MUY_03449	MUY_04433
MUY_03449	MUY_04483
MUY_03449	MUY_00450
MUY_03449	MUY_00464
MUY_03449	MUY_00808
MUY_03449	MUY_00840
MUY_03450	MUY_03450
MUY_03451	MUY_03451
MUY_03451	MUY_03775
MUY_03451	MUY_00378
MUY_03451	MUY_04230
MUY_03451	MUY_00526
MUY_03451	MUY_00857
MUY_03454	MUY_03454
MUY_03454	MUY_03637
MUY_03454	MUY_03810
MUY_03454	MUY_04070
MUY_03454	MUY_04242
MUY_03454	MUY_00382
MUY_03454	MUY_04302
MUY_03454	MUY_04383
MUY_03454	MUY_04433
MUY_03454	MUY_04483
MUY_03454	MUY_00450
MUY_03454	MUY_00464
MUY_03454	MUY_00808
MUY_03454	MUY_00840
MUY_03456	MUY_03456
MUY_03457	MUY_03457
MUY_03459	MUY_03459
MUY_03459	MUY_04116
MUY_03462	MUY_03462
MUY_03462	MUY_03463
MUY_00025	MUY_00344
MUY_00025	MUY_03937
MUY_03463	MUY_03463

MUY_03463	MUY_03477
MUY_03463	MUY_03478
MUY_03463	MUY_03483
MUY_03463	MUY_03609
MUY_03463	MUY_03619
MUY_03463	MUY_03647
MUY_03463	MUY_03648
MUY_03463	MUY_03924
MUY_03463	MUY_04096
MUY_03463	MUY_04146
MUY_03463	MUY_04177
MUY_03463	MUY_04211
MUY_03463	MUY_04212
MUY_03463	MUY_04293
MUY_03463	MUY_04294
MUY_03463	MUY_04358
MUY_03463	MUY_04359
MUY_03463	MUY_04379
MUY_03463	MUY_04417
MUY_03463	MUY_04418
MUY_03463	MUY_04447
MUY_03463	MUY_04448
MUY_03463	MUY_04471
MUY_03463	MUY_00418
MUY_03463	MUY_00436
MUY_03463	MUY_00575
MUY_03463	MUY_00576
MUY_03463	MUY_00790
MUY_03463	MUY_00961
MUY_03469	MUY_03469
MUY_03469	MUY_03470
MUY_03469	MUY_03471
MUY_03469	MUY_03472
MUY_03469	MUY_03473
MUY_03469	MUY_03474
MUY_03469	MUY_03475
MUY_03469	MUY_00890
MUY_03470	MUY_03473
MUY_03471	MUY_03473
MUY_03472	MUY_03473
MUY_03473	MUY_03473
MUY_03473	MUY_03474
MUY_03473	MUY_03475
MUY_03476	MUY_03476
MUY_03476	MUY_03520
MUY_03476	MUY_00979
MUY_03477	MUY_03477

MUY_03477	MUY_03478
MUY_03477	MUY_03483
MUY_03477	MUY_03609
MUY_03477	MUY_03619
MUY_03477	MUY_03647
MUY_03477	MUY_03648
MUY_03477	MUY_03924
MUY_03477	MUY_04096
MUY_03477	MUY_04146
MUY_03477	MUY_04177
MUY_03477	MUY_04211
MUY_03477	MUY_04212
MUY_03477	MUY_04293
MUY_03477	MUY_04294
MUY_03477	MUY_04358
MUY_03477	MUY_04359
MUY_03477	MUY_04379
MUY_03477	MUY_04417
MUY_03477	MUY_04418
MUY_03477	MUY_04447
MUY_03477	MUY_04448
MUY_03477	MUY_04471
MUY_03477	MUY_00418
MUY_03477	MUY_00436
MUY_03477	MUY_00575
MUY_03477	MUY_00576
MUY_03477	MUY_00790
MUY_03477	MUY_00961
MUY_03478	MUY_03478
MUY_03478	MUY_03609
MUY_03478	MUY_03610
MUY_03478	MUY_03619
MUY_03478	MUY_03620
MUY_03478	MUY_03647
MUY_03478	MUY_03648
MUY_03478	MUY_03924
MUY_03478	MUY_03925
MUY_03478	MUY_04096
MUY_03478	MUY_04145
MUY_03478	MUY_04146
MUY_03478	MUY_04177
MUY_03478	MUY_04211
MUY_03478	MUY_04212
MUY_03478	MUY_04293
MUY_03478	MUY_04294
MUY_03478	MUY_04358
MUY_03478	MUY_04359

MUY_03478	MUY_00393
MUY_03478	MUY_04402
MUY_03478	MUY_04417
MUY_03478	MUY_04418
MUY_03478	MUY_04420
MUY_03478	MUY_04447
MUY_03478	MUY_04448
MUY_03478	MUY_04470
MUY_03478	MUY_04471
MUY_03478	MUY_00418
MUY_03478	MUY_00436
MUY_03478	MUY_00438
MUY_03478	MUY_00575
MUY_03478	MUY_00576
MUY_00326	MUY_00326
MUY_00326	MUY_04247
MUY_03480	MUY_03480
MUY_03483	MUY_03483
MUY_03483	MUY_03609
MUY_03483	MUY_03619
MUY_03483	MUY_03648
MUY_03483	MUY_03924
MUY_03483	MUY_04096
MUY_03483	MUY_04146
MUY_03483	MUY_04177
MUY_03483	MUY_04212
MUY_03483	MUY_04293
MUY_03483	MUY_04359
MUY_03483	MUY_04379
MUY_03483	MUY_04418
MUY_03483	MUY_04448
MUY_03483	MUY_04471
MUY_03483	MUY_00418
MUY_03483	MUY_00436
MUY_03483	MUY_00576
MUY_03485	MUY_03485
MUY_03485	MUY_04035
MUY_03486	MUY_03486
MUY_00327	MUY_00327
MUY_00327	MUY_00328
MUY_00327	MUY_00329
MUY_03490	MUY_03490
MUY_03490	MUY_04046
MUY_03491	MUY_03491
MUY_03491	MUY_00854
MUY_03494	MUY_03494
MUY_03494	MUY_03606

MUY_03494	MUY_03627
MUY_03494	MUY_03638
MUY_03494	MUY_03964
MUY_03494	MUY_03998
MUY_03494	MUY_04038
MUY_03494	MUY_04045
MUY_03494	MUY_00486
MUY_03494	MUY_00681
MUY_03494	MUY_00721
MUY_03494	MUY_00933
MUY_03496	MUY_03535
MUY_03496	MUY_03610
MUY_03496	MUY_04086
MUY_03496	MUY_04466
MUY_03496	MUY_00514
MUY_03496	MUY_00780
MUY_00328	MUY_00328
MUY_00328	MUY_00329
MUY_00026	MUY_00026
MUY_00329	MUY_00329
MUY_03499	MUY_03499
MUY_03503	MUY_03503
MUY_03503	MUY_04422
MUY_03505	MUY_03505
MUY_03505	MUY_03901
MUY_03506	MUY_03506
MUY_03507	MUY_00342
MUY_03508	MUY_03508
MUY_03508	MUY_04315
MUY_03513	MUY_03513
MUY_03513	MUY_03515
MUY_03513	MUY_03524
MUY_03513	MUY_03585
MUY_03513	MUY_03706
MUY_03513	MUY_03840
MUY_03513	MUY_00459
MUY_03513	MUY_00461
MUY_03513	MUY_00523
MUY_03513	MUY_00895
MUY_03515	MUY_03515
MUY_03515	MUY_03524
MUY_03515	MUY_03585
MUY_03515	MUY_03706
MUY_03515	MUY_03840
MUY_03515	MUY_00459
MUY_03515	MUY_00461
MUY_03515	MUY_00523

MUY_03515	MUY_00895
MUY_03518	MUY_03518
MUY_03518	MUY_03667
MUY_03518	MUY_04463
MUY_03518	MUY_00557
MUY_03518	MUY_00583
MUY_03518	MUY_00674
MUY_03519	MUY_03519
MUY_03520	MUY_03520
MUY_03522	MUY_03522
MUY_03524	MUY_03524
MUY_03524	MUY_03585
MUY_03524	MUY_03706
MUY_03524	MUY_03840
MUY_03524	MUY_00459
MUY_03524	MUY_00461
MUY_03524	MUY_00523
MUY_03524	MUY_00895
MUY_03525	MUY_03525
MUY_03532	MUY_03532
MUY_03535	MUY_03535
MUY_03536	MUY_03536
MUY_03536	MUY_03833
MUY_03536	MUY_03850
MUY_03536	MUY_04207
MUY_03538	MUY_03538
MUY_03539	MUY_03539
MUY_03540	MUY_03540
MUY_03543	MUY_03543
MUY_03543	MUY_00344
MUY_03543	MUY_04049
MUY_03543	MUY_04219
MUY_03543	MUY_00810
MUY_03543	MUY_00869
MUY_03544	MUY_03544
MUY_03551	MUY_03551
MUY_03553	MUY_03553
MUY_03553	MUY_03612
MUY_03553	MUY_00445
MUY_03553	MUY_00677
MUY_03553	MUY_00749
MUY_03553	MUY_00779
MUY_03553	MUY_00882
MUY_03556	MUY_03556
MUY_03556	MUY_03573
MUY_03556	MUY_03574
MUY_03556	MUY_03660

MUY_03556	MUY_00766
MUY_03557	MUY_03557
MUY_03557	MUY_00344
MUY_03561	MUY_03561
MUY_03561	MUY_03562
MUY_03561	MUY_04373
MUY_03562	MUY_03562
MUY_03562	MUY_04373
MUY_03563	MUY_03563
MUY_03566	MUY_03566
MUY_03567	MUY_03567
MUY_03567	MUY_03840
MUY_03567	MUY_04127
MUY_03573	MUY_03573
MUY_03573	MUY_03574
MUY_03573	MUY_03660
MUY_03573	MUY_00766
MUY_03574	MUY_03574
MUY_03574	MUY_03660
MUY_03574	MUY_00766
MUY_03575	MUY_03575
MUY_03575	MUY_03576
MUY_03575	MUY_03582
MUY_03575	MUY_03598
MUY_03575	MUY_03634
MUY_03575	MUY_03649
MUY_03575	MUY_03676
MUY_03575	MUY_03785
MUY_03575	MUY_03805
MUY_03575	MUY_03900
MUY_03575	MUY_03946
MUY_03575	MUY_00372
MUY_03575	MUY_03978
MUY_03575	MUY_04144
MUY_03575	MUY_04172
MUY_03575	MUY_04248
MUY_03575	MUY_04254
MUY_03575	MUY_04266
MUY_03575	MUY_04269
MUY_03575	MUY_04282
MUY_03575	MUY_04296
MUY_03575	MUY_00388
MUY_03575	MUY_04348
MUY_03575	MUY_04357
MUY_03575	MUY_00394
MUY_03575	MUY_04403
MUY_03575	MUY_04416

MUY_03575	MUY_04445
MUY_03575	MUY_04453
MUY_03575	MUY_00420
MUY_03575	MUY_00434
MUY_03575	MUY_00442
MUY_03575	MUY_00516
MUY_03575	MUY_00585
MUY_03575	MUY_00073
MUY_03575	MUY_00800
MUY_03575	MUY_00815
MUY_03575	MUY_00937
MUY_03575	MUY_00938
MUY_03575	MUY_00939
MUY_03575	MUY_00968
MUY_03575	MUY_00975
MUY_03575	MUY_00996
MUY_03575	MUY_01000
MUY_03576	MUY_03576
MUY_03576	MUY_03582
MUY_03576	MUY_03598
MUY_03576	MUY_03634
MUY_03576	MUY_03649
MUY_03576	MUY_03676
MUY_03576	MUY_03785
MUY_03576	MUY_03805
MUY_03576	MUY_03900
MUY_03576	MUY_03946
MUY_03576	MUY_00372
MUY_03576	MUY_03978
MUY_03576	MUY_04144
MUY_03576	MUY_04172
MUY_03576	MUY_04248
MUY_03576	MUY_04254
MUY_03576	MUY_04266
MUY_03576	MUY_04269
MUY_03576	MUY_04282
MUY_03576	MUY_04296
MUY_03576	MUY_00388
MUY_03576	MUY_04348
MUY_03576	MUY_04357
MUY_03576	MUY_00394
MUY_03576	MUY_04403
MUY_03576	MUY_04416
MUY_03576	MUY_04445
MUY_03576	MUY_04453
MUY_03576	MUY_00420
MUY_03576	MUY_00434

MUY_03576	MUY_00442
MUY_03576	MUY_00516
MUY_03576	MUY_00585
MUY_03576	MUY_00800
MUY_03576	MUY_00815
MUY_03576	MUY_00937
MUY_03576	MUY_00938
MUY_03576	MUY_00939
MUY_03576	MUY_00968
MUY_03576	MUY_00975
MUY_03576	MUY_00996
MUY_03576	MUY_01000
MUY_03579	MUY_03579
MUY_03579	MUY_04503
MUY_03580	MUY_03580
MUY_03580	MUY_00371
MUY_03580	MUY_00702
MUY_03582	MUY_03582
MUY_03582	MUY_03598
MUY_03582	MUY_03634
MUY_03582	MUY_03649
MUY_03582	MUY_03676
MUY_03582	MUY_03785
MUY_03582	MUY_03805
MUY_03582	MUY_03900
MUY_03582	MUY_03946
MUY_03582	MUY_00372
MUY_03582	MUY_03978
MUY_03582	MUY_04144
MUY_03582	MUY_04172
MUY_03582	MUY_04248
MUY_03582	MUY_04254
MUY_03582	MUY_04266
MUY_03582	MUY_04269
MUY_03582	MUY_04282
MUY_03582	MUY_04296
MUY_03582	MUY_00388
MUY_03582	MUY_04348
MUY_03582	MUY_04357
MUY_03582	MUY_00394
MUY_03582	MUY_04403
MUY_03582	MUY_04416
MUY_03582	MUY_04445
MUY_03582	MUY_04453
MUY_03582	MUY_00420
MUY_03582	MUY_00434
MUY_03582	MUY_00442

MUY_03582	MUY_00516
MUY_03582	MUY_00585
MUY_03582	MUY_00800
MUY_03582	MUY_00815
MUY_03582	MUY_00937
MUY_03582	MUY_00938
MUY_03582	MUY_00939
MUY_03582	MUY_00968
MUY_03582	MUY_00975
MUY_03582	MUY_00996
MUY_03582	MUY_01000
MUY_03584	MUY_03584
MUY_03585	MUY_03585
MUY_03585	MUY_04073
MUY_03585	MUY_00461
MUY_03585	MUY_00523
MUY_03585	MUY_00861
MUY_03586	MUY_03586
MUY_03586	MUY_03904
MUY_03586	MUY_03922
MUY_03586	MUY_04367
MUY_03586	MUY_00496
MUY_03586	MUY_00046
MUY_03586	MUY_00527
MUY_03586	MUY_00008
MUY_03586	MUY_00816
MUY_00337	MUY_00337
MUY_00337	MUY_04086
MUY_00337	MUY_00433
MUY_00337	MUY_00813
MUY_03589	MUY_03589
MUY_03590	MUY_03590
MUY_03590	MUY_04103
MUY_03590	MUY_04104
MUY_03590	MUY_00519
MUY_03591	MUY_04106
MUY_03592	MUY_03592
MUY_03592	MUY_04105
MUY_03598	MUY_03598
MUY_03598	MUY_03634
MUY_03598	MUY_03649
MUY_03598	MUY_00344
MUY_03598	MUY_03676
MUY_03598	MUY_03785
MUY_03598	MUY_03805
MUY_03598	MUY_03900
MUY_03598	MUY_03946

MUY_03598	MUY_00372
MUY_03598	MUY_03978
MUY_03598	MUY_04144
MUY_03598	MUY_04172
MUY_03598	MUY_04248
MUY_03598	MUY_04254
MUY_03598	MUY_04266
MUY_03598	MUY_04269
MUY_03598	MUY_04282
MUY_03598	MUY_04296
MUY_03598	MUY_00388
MUY_03598	MUY_04348
MUY_03598	MUY_04357
MUY_03598	MUY_00394
MUY_03598	MUY_04403
MUY_03598	MUY_04416
MUY_03598	MUY_04445
MUY_03598	MUY_04453
MUY_03598	MUY_00420
MUY_03598	MUY_00434
MUY_03598	MUY_00442
MUY_03598	MUY_00516
MUY_03598	MUY_00585
MUY_03598	MUY_00800
MUY_03598	MUY_00815
MUY_03598	MUY_00937
MUY_03598	MUY_00938
MUY_03598	MUY_00939
MUY_03598	MUY_00968
MUY_03598	MUY_00975
MUY_03598	MUY_00996
MUY_03598	MUY_01000
MUY_03601	MUY_03601
MUY_03601	MUY_03792
MUY_03601	MUY_04042
MUY_03601	MUY_04408
MUY_03601	MUY_00443
MUY_03603	MUY_03603
MUY_03605	MUY_03605
MUY_03606	MUY_03606
MUY_03606	MUY_03627
MUY_03606	MUY_03638
MUY_03606	MUY_03964
MUY_03606	MUY_03998
MUY_03606	MUY_04038
MUY_03606	MUY_04045
MUY_03606	MUY_00486

MUY_03606	MUY_00681
MUY_03606	MUY_00721
MUY_03606	MUY_00933
MUY_03608	MUY_03608
MUY_03608	MUY_03612
MUY_03608	MUY_03896
MUY_03608	MUY_03980
MUY_03608	MUY_04224
MUY_03608	MUY_04466
MUY_03608	MUY_00825
MUY_03609	MUY_03609
MUY_03609	MUY_03619
MUY_03609	MUY_03647
MUY_03609	MUY_03648
MUY_03609	MUY_03924
MUY_03609	MUY_04096
MUY_03609	MUY_04146
MUY_03609	MUY_04177
MUY_03609	MUY_04211
MUY_03609	MUY_04212
MUY_03609	MUY_04293
MUY_03609	MUY_04294
MUY_03609	MUY_04358
MUY_03609	MUY_04359
MUY_03609	MUY_04379
MUY_03609	MUY_04417
MUY_03609	MUY_04418
MUY_03609	MUY_04447
MUY_03609	MUY_04448
MUY_03609	MUY_04471
MUY_03609	MUY_00418
MUY_03609	MUY_00436
MUY_03609	MUY_00575
MUY_03609	MUY_00576
MUY_03609	MUY_00790
MUY_03609	MUY_00961
MUY_03610	MUY_03610
MUY_03610	MUY_03620
MUY_03610	MUY_03647
MUY_03610	MUY_04086
MUY_03610	MUY_04211
MUY_03610	MUY_04294
MUY_03610	MUY_04334
MUY_03610	MUY_04358
MUY_03610	MUY_00393
MUY_03610	MUY_04417
MUY_03610	MUY_04420

MUY_03610	MUY_04447
MUY_03610	MUY_04470
MUY_03610	MUY_00435
MUY_03610	MUY_00438
MUY_03610	MUY_00575
MUY_03610	MUY_00603
MUY_03610	MUY_00738
MUY_03610	MUY_00780
MUY_03612	MUY_03612
MUY_03612	MUY_03863
MUY_03612	MUY_04467
MUY_03612	MUY_00445
MUY_03612	MUY_00677
MUY_03612	MUY_00749
MUY_03612	MUY_00779
MUY_03612	MUY_00882
MUY_03613	MUY_03613
MUY_03613	MUY_04289
MUY_03613	MUY_00507
MUY_03613	MUY_00738
MUY_00340	MUY_00340
MUY_03619	MUY_03619
MUY_03619	MUY_03647
MUY_03619	MUY_03648
MUY_03619	MUY_03924
MUY_03619	MUY_04096
MUY_03619	MUY_04146
MUY_03619	MUY_04177
MUY_03619	MUY_04211
MUY_03619	MUY_04212
MUY_03619	MUY_04293
MUY_03619	MUY_04294
MUY_03619	MUY_04358
MUY_03619	MUY_04359
MUY_03619	MUY_04379
MUY_03619	MUY_04417
MUY_03619	MUY_04418
MUY_03619	MUY_04447
MUY_03619	MUY_04448
MUY_03619	MUY_04471
MUY_03619	MUY_00418
MUY_03619	MUY_00436
MUY_03619	MUY_00575
MUY_03619	MUY_00576
MUY_03619	MUY_00790
MUY_03619	MUY_00961
MUY_03620	MUY_03620

MUY_03620	MUY_03647
MUY_03620	MUY_04211
MUY_03620	MUY_04294
MUY_03620	MUY_04334
MUY_03620	MUY_04358
MUY_03620	MUY_00393
MUY_03620	MUY_04417
MUY_03620	MUY_04420
MUY_03620	MUY_04447
MUY_03620	MUY_04470
MUY_03620	MUY_00435
MUY_03620	MUY_00438
MUY_03620	MUY_00575
MUY_03625	MUY_03625
MUY_03626	MUY_03626
MUY_03627	MUY_03627
MUY_03627	MUY_03638
MUY_03627	MUY_03964
MUY_03627	MUY_03998
MUY_03627	MUY_04038
MUY_03627	MUY_00486
MUY_03627	MUY_00681
MUY_03627	MUY_00933
MUY_03628	MUY_03628
MUY_03628	MUY_03834
MUY_03632	MUY_03632
MUY_03632	MUY_03836
MUY_03632	MUY_00872
MUY_03633	MUY_03633
MUY_03633	MUY_03837
MUY_03634	MUY_03634
MUY_03634	MUY_03649
MUY_03634	MUY_03676
MUY_03634	MUY_03785
MUY_03634	MUY_03805
MUY_03634	MUY_03900
MUY_03634	MUY_03946
MUY_03634	MUY_00372
MUY_03634	MUY_03978
MUY_03634	MUY_04144
MUY_03634	MUY_04172
MUY_03634	MUY_04248
MUY_03634	MUY_04254
MUY_03634	MUY_04266
MUY_03634	MUY_04269
MUY_03634	MUY_04282
MUY_03634	MUY_04296

MUY_03634	MUY_00388
MUY_03634	MUY_04348
MUY_03634	MUY_04357
MUY_03634	MUY_00394
MUY_03634	MUY_04403
MUY_03634	MUY_04416
MUY_03634	MUY_04445
MUY_03634	MUY_04453
MUY_03634	MUY_00420
MUY_03634	MUY_00434
MUY_03634	MUY_00442
MUY_03634	MUY_00516
MUY_03634	MUY_00585
MUY_03634	MUY_00800
MUY_03634	MUY_00815
MUY_03634	MUY_00937
MUY_03634	MUY_00938
MUY_03634	MUY_00939
MUY_03634	MUY_00968
MUY_03634	MUY_00975
MUY_03634	MUY_00996
MUY_03634	MUY_01000
MUY_03637	MUY_03637
MUY_03637	MUY_03810
MUY_03637	MUY_04070
MUY_03637	MUY_04242
MUY_03637	MUY_00382
MUY_03637	MUY_04302
MUY_03637	MUY_04383
MUY_03637	MUY_04433
MUY_03637	MUY_04483
MUY_03637	MUY_00450
MUY_03637	MUY_00464
MUY_03637	MUY_00808
MUY_03637	MUY_00840
MUY_03638	MUY_03638
MUY_03638	MUY_03964
MUY_03638	MUY_03998
MUY_03638	MUY_04038
MUY_03638	MUY_04045
MUY_03638	MUY_00486
MUY_03638	MUY_00681
MUY_03638	MUY_00721
MUY_03638	MUY_00933
MUY_03639	MUY_03639
MUY_03639	MUY_00830
MUY_03640	MUY_03640

MUY_03640	MUY_03984
MUY_03640	MUY_04198
MUY_03640	MUY_00401
MUY_03640	MUY_00563
MUY_03642	MUY_03642
MUY_03643	MUY_03643
MUY_03643	MUY_00344
MUY_03643	MUY_00484
MUY_03644	MUY_03644
MUY_03644	MUY_03962
MUY_03644	MUY_03975
MUY_03644	MUY_04082
MUY_03644	MUY_04328
MUY_03644	MUY_04428
MUY_03644	MUY_00700
MUY_03645	MUY_00780
MUY_03646	MUY_03646
MUY_03646	MUY_00344
MUY_03647	MUY_03647
MUY_03647	MUY_03648
MUY_03647	MUY_03924
MUY_03647	MUY_04096
MUY_03647	MUY_04146
MUY_03647	MUY_04177
MUY_03647	MUY_04211
MUY_03647	MUY_04212
MUY_03647	MUY_04293
MUY_03647	MUY_04294
MUY_03647	MUY_04358
MUY_03647	MUY_04359
MUY_03647	MUY_00393
MUY_03647	MUY_04417
MUY_03647	MUY_04418
MUY_03647	MUY_04420
MUY_03647	MUY_04447
MUY_03647	MUY_04448
MUY_03647	MUY_04470
MUY_03647	MUY_04471
MUY_03647	MUY_00418
MUY_03647	MUY_00436
MUY_03647	MUY_00438
MUY_03647	MUY_00575
MUY_03647	MUY_00576
MUY_03648	MUY_03648
MUY_03648	MUY_03924
MUY_03648	MUY_04096
MUY_03648	MUY_04146

MUY_03648	MUY_04177
MUY_03648	MUY_04211
MUY_03648	MUY_04212
MUY_03648	MUY_04293
MUY_03648	MUY_04294
MUY_03648	MUY_04358
MUY_03648	MUY_04359
MUY_03648	MUY_04379
MUY_03648	MUY_04417
MUY_03648	MUY_04418
MUY_03648	MUY_04447
MUY_03648	MUY_04448
MUY_03648	MUY_04471
MUY_03648	MUY_00418
MUY_03648	MUY_00436
MUY_03648	MUY_00575
MUY_03648	MUY_00576
MUY_03648	MUY_00790
MUY_03648	MUY_00961
MUY_03649	MUY_03649
MUY_03649	MUY_03676
MUY_03649	MUY_03785
MUY_03649	MUY_03805
MUY_03649	MUY_03900
MUY_03649	MUY_03946
MUY_03649	MUY_00372
MUY_03649	MUY_03978
MUY_03649	MUY_04144
MUY_03649	MUY_04172
MUY_03649	MUY_04248
MUY_03649	MUY_04254
MUY_03649	MUY_04266
MUY_03649	MUY_04269
MUY_03649	MUY_04282
MUY_03649	MUY_04296
MUY_03649	MUY_00388
MUY_03649	MUY_04348
MUY_03649	MUY_04357
MUY_03649	MUY_00394
MUY_03649	MUY_04403
MUY_03649	MUY_04416
MUY_03649	MUY_04445
MUY_03649	MUY_04453
MUY_03649	MUY_00420
MUY_03649	MUY_00434
MUY_03649	MUY_00442
MUY_03649	MUY_00516

MUY_03649	MUY_00585
MUY_03649	MUY_00800
MUY_03649	MUY_00815
MUY_03649	MUY_00937
MUY_03649	MUY_00938
MUY_03649	MUY_00939
MUY_03649	MUY_00968
MUY_03649	MUY_00975
MUY_03649	MUY_00996
MUY_03649	MUY_01000
MUY_03658	MUY_03658
MUY_03658	MUY_04399
MUY_03659	MUY_03659
MUY_03659	MUY_03701
MUY_03659	MUY_03880
MUY_03659	MUY_04014
MUY_03659	MUY_04156
MUY_03659	MUY_04194
MUY_03659	MUY_04345
MUY_03659	MUY_04361
MUY_03659	MUY_04485
MUY_03659	MUY_00556
MUY_03659	MUY_00710
MUY_03659	MUY_00787
MUY_03659	MUY_00838
MUY_03659	MUY_00886
MUY_03660	MUY_03660
MUY_03660	MUY_00766
MUY_00344	MUY_00344
MUY_00344	MUY_03684
MUY_00344	MUY_03868
MUY_00344	MUY_03957
MUY_00344	MUY_03963
MUY_00344	MUY_04024
MUY_00344	MUY_04092
MUY_00344	MUY_04112
MUY_00344	MUY_04131
MUY_00344	MUY_04244
MUY_00344	MUY_04245
MUY_00344	MUY_04315
MUY_00344	MUY_04324
MUY_00344	MUY_04438
MUY_00344	MUY_04481
MUY_00344	MUY_04482
MUY_00344	MUY_00044
MUY_00344	MUY_00580
MUY_00344	MUY_00680

MUY_00344	MUY_00730
MUY_00344	MUY_00737
MUY_00344	MUY_00766
MUY_00344	MUY_00839
MUY_00344	MUY_00081
MUY_00344	MUY_00971
MUY_00344	MUY_00989
MUY_00344	MUY_00998
MUY_03667	MUY_03667
MUY_03667	MUY_04463
MUY_03667	MUY_00557
MUY_03667	MUY_00583
MUY_03667	MUY_00674
MUY_03669	MUY_03669
MUY_03670	MUY_03670
MUY_03670	MUY_03686
MUY_03674	MUY_03674
MUY_00345	MUY_00345
MUY_03675	MUY_03675
MUY_03676	MUY_03676
MUY_03676	MUY_03785
MUY_03676	MUY_03805
MUY_03676	MUY_03900
MUY_03676	MUY_03946
MUY_03676	MUY_00372
MUY_03676	MUY_03978
MUY_03676	MUY_04144
MUY_03676	MUY_04172
MUY_03676	MUY_04248
MUY_03676	MUY_04254
MUY_03676	MUY_04266
MUY_03676	MUY_04269
MUY_03676	MUY_04282
MUY_03676	MUY_04296
MUY_03676	MUY_00388
MUY_03676	MUY_04348
MUY_03676	MUY_04357
MUY_03676	MUY_00394
MUY_03676	MUY_04403
MUY_03676	MUY_04416
MUY_03676	MUY_04445
MUY_03676	MUY_04453
MUY_03676	MUY_00420
MUY_03676	MUY_00434
MUY_03676	MUY_00442
MUY_03676	MUY_00516
MUY_03676	MUY_00585

MUY_03676	MUY_00800
MUY_03676	MUY_00815
MUY_03676	MUY_00937
MUY_03676	MUY_00938
MUY_03676	MUY_00939
MUY_03676	MUY_00968
MUY_03676	MUY_00975
MUY_03676	MUY_00996
MUY_03676	MUY_01000
MUY_03678	MUY_03678
MUY_03680	MUY_03680
MUY_03680	MUY_00900
MUY_03681	MUY_03681
MUY_03681	MUY_03961
MUY_03681	MUY_03976
MUY_03681	MUY_04153
MUY_03681	MUY_04218
MUY_03681	MUY_04395
MUY_03682	MUY_03683
MUY_03682	MUY_00483
MUY_03683	MUY_03683
MUY_03683	MUY_03856
MUY_03683	MUY_04238
MUY_03683	MUY_00470
MUY_03683	MUY_00482
MUY_03683	MUY_00483
MUY_03686	MUY_03686
MUY_03686	MUY_03687
MUY_03689	MUY_03689
MUY_03689	MUY_03794
MUY_03689	MUY_04260
MUY_03689	MUY_04262
MUY_03689	MUY_00892
MUY_03690	MUY_03690
MUY_03691	MUY_03691
MUY_03691	MUY_04192
MUY_03691	MUY_04434
MUY_03692	MUY_03692
MUY_03692	MUY_04193
MUY_03692	MUY_04389
MUY_03692	MUY_04390
MUY_03692	MUY_04393
MUY_03693	MUY_03693
MUY_03694	MUY_03694
MUY_00347	MUY_00347
MUY_00347	MUY_04132
MUY_00347	MUY_04150

MUY_00347	MUY_04397
MUY_00347	MUY_04438
MUY_00347	MUY_00453
MUY_00347	MUY_00797
MUY_03699	MUY_03699
MUY_03699	MUY_00378
MUY_03699	MUY_04230
MUY_03699	MUY_00750
MUY_03699	MUY_00811
MUY_03701	MUY_03701
MUY_03701	MUY_03880
MUY_03701	MUY_04014
MUY_03701	MUY_04194
MUY_03701	MUY_00556
MUY_03701	MUY_00710
MUY_03702	MUY_03702
MUY_03702	MUY_00774
MUY_03702	MUY_00775
MUY_03703	MUY_03703
MUY_03703	MUY_04079
MUY_03703	MUY_04391
MUY_03703	MUY_00852
MUY_03704	MUY_03704
MUY_03704	MUY_03831
MUY_03704	MUY_00360
MUY_03704	MUY_00911
MUY_03706	MUY_03706
MUY_03706	MUY_00366
MUY_03706	MUY_04467
MUY_03706	MUY_00461
MUY_03706	MUY_00072
MUY_03706	MUY_00824
MUY_03707	MUY_03708
MUY_03708	MUY_03708
MUY_03708	MUY_03769
MUY_03708	MUY_03778
MUY_03708	MUY_03830
MUY_03708	MUY_03918
MUY_03708	MUY_04026
MUY_03708	MUY_04258
MUY_03708	MUY_04271
MUY_03708	MUY_04291
MUY_03708	MUY_04322
MUY_03708	MUY_04504
MUY_03708	MUY_00541
MUY_03708	MUY_00551
MUY_03708	MUY_00571

MUY_03708	MUY_00599
MUY_03708	MUY_00602
MUY_03708	MUY_00628
MUY_03708	MUY_00691
MUY_03708	MUY_00795
MUY_03708	MUY_00086
MUY_03708	MUY_00973
MUY_03710	MUY_03710
MUY_03715	MUY_03715
MUY_03715	MUY_03896
MUY_03715	MUY_00656
MUY_03720	MUY_03720
MUY_03721	MUY_03721
MUY_03721	MUY_00512
MUY_03722	MUY_03722
MUY_03727	MUY_03727
MUY_03739	MUY_03739
MUY_03739	MUY_00643
MUY_03742	MUY_03742
MUY_03742	MUY_03844
MUY_03742	MUY_04025
MUY_03743	MUY_03743
MUY_03747	MUY_03747
MUY_03754	MUY_03754
MUY_03769	MUY_03769
MUY_03769	MUY_03778
MUY_03769	MUY_03830
MUY_03769	MUY_04185
MUY_03769	MUY_04271
MUY_03769	MUY_04291
MUY_03769	MUY_04501
MUY_03769	MUY_04505
MUY_03769	MUY_00551
MUY_03769	MUY_00599
MUY_03769	MUY_00602
MUY_03769	MUY_00628
MUY_03769	MUY_00691
MUY_03769	MUY_00795
MUY_03769	MUY_00086
MUY_03769	MUY_00973
MUY_03771	MUY_03771
MUY_03771	MUY_00598
MUY_03773	MUY_03773
MUY_03774	MUY_03774
MUY_03774	MUY_04205
MUY_03774	MUY_04494
MUY_03774	MUY_00979

MUY_03774	MUY_00998
MUY_03775	MUY_03775
MUY_03775	MUY_00378
MUY_03775	MUY_04230
MUY_03775	MUY_00526
MUY_03775	MUY_00857
MUY_03777	MUY_03777
MUY_03777	MUY_00042
MUY_03777	MUY_00534
MUY_03778	MUY_03778
MUY_03778	MUY_03830
MUY_03778	MUY_04185
MUY_03778	MUY_04271
MUY_03778	MUY_04291
MUY_03778	MUY_04501
MUY_03778	MUY_04505
MUY_03778	MUY_00551
MUY_03778	MUY_00599
MUY_03778	MUY_00602
MUY_03778	MUY_00628
MUY_03778	MUY_00691
MUY_03778	MUY_00795
MUY_03778	MUY_00086
MUY_03778	MUY_00973
MUY_03779	MUY_03779
MUY_03779	MUY_03780
MUY_03780	MUY_03780
MUY_00005	MUY_00005
MUY_00005	MUY_00857
MUY_03783	MUY_03783
MUY_03785	MUY_03785
MUY_03785	MUY_03805
MUY_03785	MUY_03900
MUY_03785	MUY_03946
MUY_03785	MUY_00372
MUY_03785	MUY_03978
MUY_03785	MUY_04144
MUY_03785	MUY_04172
MUY_03785	MUY_04248
MUY_03785	MUY_04254
MUY_03785	MUY_04266
MUY_03785	MUY_04269
MUY_03785	MUY_04282
MUY_03785	MUY_04296
MUY_03785	MUY_00388
MUY_03785	MUY_04348
MUY_03785	MUY_04357

MUY_03785	MUY_00394
MUY_03785	MUY_04403
MUY_03785	MUY_04416
MUY_03785	MUY_04445
MUY_03785	MUY_04453
MUY_03785	MUY_00420
MUY_03785	MUY_00434
MUY_03785	MUY_00442
MUY_03785	MUY_00516
MUY_03785	MUY_00585
MUY_03785	MUY_00800
MUY_03785	MUY_00815
MUY_03785	MUY_00937
MUY_03785	MUY_00938
MUY_03785	MUY_00939
MUY_03785	MUY_00968
MUY_03785	MUY_00975
MUY_03785	MUY_00996
MUY_03785	MUY_01000
MUY_03786	MUY_03786
MUY_03786	MUY_04432
MUY_03786	MUY_00423
MUY_03789	MUY_00435
MUY_03789	MUY_00780
MUY_00353	MUY_00353
MUY_03790	MUY_03790
MUY_03790	MUY_04099
MUY_03790	MUY_04305
MUY_03790	MUY_00630
MUY_03791	MUY_03791
MUY_03791	MUY_04098
MUY_03791	MUY_04304
MUY_03792	MUY_03792
MUY_03792	MUY_04042
MUY_03792	MUY_04408
MUY_03792	MUY_00443
MUY_03794	MUY_03794
MUY_03794	MUY_04260
MUY_03794	MUY_04262
MUY_03794	MUY_00892
MUY_03795	MUY_03795
MUY_03796	MUY_03796
MUY_03798	MUY_03798
MUY_00354	MUY_00354
MUY_03800	MUY_03800
MUY_03802	MUY_03802
MUY_03802	MUY_04161

MUY_03802	MUY_00688
MUY_03802	MUY_00971
MUY_03805	MUY_03805
MUY_03805	MUY_03900
MUY_03805	MUY_03946
MUY_03805	MUY_00372
MUY_03805	MUY_03978
MUY_03805	MUY_04144
MUY_03805	MUY_04172
MUY_03805	MUY_04248
MUY_03805	MUY_04254
MUY_03805	MUY_04266
MUY_03805	MUY_04269
MUY_03805	MUY_04282
MUY_03805	MUY_04296
MUY_03805	MUY_00388
MUY_03805	MUY_04348
MUY_03805	MUY_04357
MUY_03805	MUY_00394
MUY_03805	MUY_04403
MUY_03805	MUY_04416
MUY_03805	MUY_04445
MUY_03805	MUY_04453
MUY_03805	MUY_00420
MUY_03805	MUY_00434
MUY_03805	MUY_00442
MUY_03805	MUY_00516
MUY_03805	MUY_00585
MUY_03805	MUY_00800
MUY_03805	MUY_00815
MUY_03805	MUY_00937
MUY_03805	MUY_00938
MUY_03805	MUY_00939
MUY_03805	MUY_00968
MUY_03805	MUY_00975
MUY_03805	MUY_00996
MUY_03805	MUY_01000
MUY_03810	MUY_03810
MUY_03810	MUY_04070
MUY_03810	MUY_04242
MUY_03810	MUY_00382
MUY_03810	MUY_04302
MUY_03810	MUY_04383
MUY_03810	MUY_04433
MUY_03810	MUY_04483
MUY_03810	MUY_00450
MUY_03810	MUY_00464

MUY_03810	MUY_00808
MUY_03810	MUY_00840
MUY_03812	MUY_03812
MUY_03815	MUY_03815
MUY_03815	MUY_04081
MUY_03815	MUY_00845
MUY_03815	MUY_00851
MUY_03819	MUY_03819
MUY_03819	MUY_03821
MUY_03819	MUY_03824
MUY_03819	MUY_03931
MUY_03819	MUY_04152
MUY_03819	MUY_04334
MUY_03819	MUY_04402
MUY_03819	MUY_00927
MUY_03821	MUY_03821
MUY_03821	MUY_03824
MUY_03821	MUY_03931
MUY_03821	MUY_04152
MUY_03821	MUY_04334
MUY_03821	MUY_00927
MUY_03823	MUY_03823
MUY_03823	MUY_03936
MUY_03823	MUY_03949
MUY_03823	MUY_00849
MUY_03824	MUY_03824
MUY_03824	MUY_03931
MUY_03824	MUY_04152
MUY_03824	MUY_04334
MUY_03824	MUY_00927
MUY_03826	MUY_03826
MUY_03828	MUY_03828
MUY_03828	MUY_04502
MUY_03830	MUY_03830
MUY_03830	MUY_04185
MUY_03830	MUY_04271
MUY_03830	MUY_04291
MUY_03830	MUY_04501
MUY_03830	MUY_04505
MUY_03830	MUY_00551
MUY_03830	MUY_00599
MUY_03830	MUY_00602
MUY_03830	MUY_00628
MUY_03830	MUY_00691
MUY_03830	MUY_00795
MUY_03830	MUY_00086
MUY_03830	MUY_00973

MUY_03831	MUY_03831
MUY_03831	MUY_00360
MUY_03831	MUY_00911
MUY_03833	MUY_03833
MUY_03833	MUY_03850
MUY_03833	MUY_04207
MUY_00360	MUY_00360
MUY_00360	MUY_00911
MUY_03834	MUY_03834
MUY_03835	MUY_03835
MUY_03836	MUY_03836
MUY_03836	MUY_00872
MUY_03837	MUY_03837
MUY_03838	MUY_03838
MUY_03839	MUY_03839
MUY_03839	MUY_00560
MUY_03839	MUY_00855
MUY_03839	MUY_00904
MUY_03840	MUY_03840
MUY_03840	MUY_00461
MUY_03842	MUY_03842
MUY_03842	MUY_04155
MUY_03842	MUY_04326
MUY_03844	MUY_03844
MUY_03844	MUY_04025
MUY_03845	MUY_03845
MUY_00362	MUY_00362
MUY_03846	MUY_03846
MUY_03847	MUY_03847
MUY_03847	MUY_04122
MUY_03848	MUY_03848
MUY_03848	MUY_00378
MUY_03848	MUY_04230
MUY_03848	MUY_04492
MUY_03848	MUY_00535
MUY_03848	MUY_00536
MUY_03848	MUY_00537
MUY_03848	MUY_00539
MUY_03848	MUY_00540
MUY_03849	MUY_03849
MUY_03849	MUY_00888
MUY_03850	MUY_03850
MUY_03850	MUY_04207
MUY_03851	MUY_03851
MUY_03853	MUY_03853
MUY_03853	MUY_03854
MUY_03854	MUY_03854

MUY_03855	MUY_03855
MUY_03856	MUY_03856
MUY_03856	MUY_03879
MUY_03856	MUY_03980
MUY_03856	MUY_00483
MUY_03858	MUY_03858
MUY_03859	MUY_04466
MUY_03860	MUY_03860
MUY_03860	MUY_03951
MUY_03860	MUY_04473
MUY_03860	MUY_00773
MUY_03860	MUY_00912
MUY_03862	MUY_03862
MUY_03862	MUY_04011
MUY_03863	MUY_03863
MUY_03863	MUY_03904
MUY_03863	MUY_03922
MUY_03863	MUY_04367
MUY_03863	MUY_00435
MUY_03863	MUY_00527
MUY_03863	MUY_00790
MUY_03863	MUY_00816
MUY_03863	MUY_00857
MUY_03863	MUY_00961
MUY_00363	MUY_00363
MUY_03864	MUY_03864
MUY_03865	MUY_03865
MUY_03866	MUY_03866
MUY_03866	MUY_03868
MUY_03866	MUY_00730
MUY_03866	MUY_00081
MUY_03867	MUY_00081
MUY_03868	MUY_03868
MUY_03868	MUY_04037
MUY_03868	MUY_04100
MUY_03868	MUY_00730
MUY_03868	MUY_00080
MUY_03868	MUY_00081
MUY_03869	MUY_03869
MUY_03871	MUY_03871
MUY_03871	MUY_03872
MUY_03872	MUY_03872
MUY_03876	MUY_03876
MUY_03876	MUY_04337
MUY_03876	MUY_00708
MUY_03879	MUY_03879
MUY_03880	MUY_03880

MUY_03880	MUY_04014
MUY_03880	MUY_04194
MUY_03880	MUY_00556
MUY_03880	MUY_00710
MUY_00366	MUY_00366
MUY_00366	MUY_04318
MUY_00366	MUY_00582
MUY_00366	MUY_00584
MUY_00366	MUY_00648
MUY_00366	MUY_00072
MUY_03891	MUY_03891
MUY_03896	MUY_03896
MUY_03896	MUY_04466
MUY_03896	MUY_00935
MUY_03897	MUY_03897
MUY_03900	MUY_03900
MUY_03900	MUY_03946
MUY_03900	MUY_00372
MUY_03900	MUY_03978
MUY_03900	MUY_04144
MUY_03900	MUY_04172
MUY_03900	MUY_04248
MUY_03900	MUY_04254
MUY_03900	MUY_04266
MUY_03900	MUY_04269
MUY_03900	MUY_04282
MUY_03900	MUY_04296
MUY_03900	MUY_00388
MUY_03900	MUY_04348
MUY_03900	MUY_04357
MUY_03900	MUY_00394
MUY_03900	MUY_04403
MUY_03900	MUY_04416
MUY_03900	MUY_04445
MUY_03900	MUY_04453
MUY_03900	MUY_00420
MUY_03900	MUY_00434
MUY_03900	MUY_00442
MUY_03900	MUY_00516
MUY_03900	MUY_00585
MUY_03900	MUY_00800
MUY_03900	MUY_00815
MUY_03900	MUY_00937
MUY_03900	MUY_00938
MUY_03900	MUY_00939
MUY_03900	MUY_00968
MUY_03900	MUY_00975

MUY_03900	MUY_00996
MUY_03900	MUY_01000
MUY_03901	MUY_03901
MUY_03902	MUY_03902
MUY_03902	MUY_04312
MUY_03902	MUY_00561
MUY_03902	MUY_00734
MUY_03904	MUY_03904
MUY_03904	MUY_03922
MUY_03904	MUY_04367
MUY_03904	MUY_00496
MUY_03904	MUY_00046
MUY_03904	MUY_00527
MUY_03904	MUY_00816
MUY_03907	MUY_03909
MUY_03913	MUY_03913
MUY_03918	MUY_03918
MUY_03918	MUY_04026
MUY_03918	MUY_04258
MUY_03918	MUY_04322
MUY_03918	MUY_04504
MUY_03918	MUY_00541
MUY_03918	MUY_00571
MUY_03918	MUY_00628
MUY_03922	MUY_03922
MUY_03922	MUY_04367
MUY_03922	MUY_00496
MUY_03922	MUY_00046
MUY_03922	MUY_00527
MUY_03922	MUY_00816
MUY_03923	MUY_03923
MUY_03924	MUY_03924
MUY_03924	MUY_04096
MUY_03924	MUY_04146
MUY_03924	MUY_04177
MUY_03924	MUY_04211
MUY_03924	MUY_04212
MUY_03924	MUY_04293
MUY_03924	MUY_04294
MUY_03924	MUY_04358
MUY_03924	MUY_04359
MUY_03924	MUY_04379
MUY_03924	MUY_04417
MUY_03924	MUY_04418
MUY_03924	MUY_04447
MUY_03924	MUY_04448
MUY_03924	MUY_04471

MUY_03924	MUY_00418
MUY_03924	MUY_00436
MUY_03924	MUY_00575
MUY_03924	MUY_00576
MUY_03924	MUY_00790
MUY_03924	MUY_00961
MUY_03925	MUY_03925
MUY_03925	MUY_04145
MUY_03925	MUY_04211
MUY_03925	MUY_04294
MUY_03925	MUY_04358
MUY_03925	MUY_04447
MUY_03925	MUY_00782
MUY_03927	MUY_03927
MUY_03927	MUY_03942
MUY_03927	MUY_03968
MUY_03927	MUY_00492
MUY_03929	MUY_03950
MUY_03929	MUY_03952
MUY_03929	MUY_03953
MUY_03930	MUY_03930
MUY_03931	MUY_03931
MUY_03931	MUY_04152
MUY_03931	MUY_04334
MUY_03931	MUY_00927
MUY_03934	MUY_03934
MUY_03934	MUY_04183
MUY_03934	MUY_04335
MUY_03934	MUY_04430
MUY_03934	MUY_00847
MUY_03934	MUY_00921
MUY_03935	MUY_03935
MUY_03936	MUY_03936
MUY_03936	MUY_03949
MUY_03936	MUY_00849
MUY_03942	MUY_03942
MUY_03942	MUY_03968
MUY_03942	MUY_00492
MUY_03943	MUY_03943
MUY_03945	MUY_03945
MUY_03945	MUY_00056
MUY_03946	MUY_03946
MUY_03946	MUY_00372
MUY_03946	MUY_03978
MUY_03946	MUY_04144
MUY_03946	MUY_04172
MUY_03946	MUY_04248

MUY_03946	MUY_04254
MUY_03946	MUY_04266
MUY_03946	MUY_04269
MUY_03946	MUY_04282
MUY_03946	MUY_04296
MUY_03946	MUY_00388
MUY_03946	MUY_04348
MUY_03946	MUY_04357
MUY_03946	MUY_00394
MUY_03946	MUY_04403
MUY_03946	MUY_04416
MUY_03946	MUY_04445
MUY_03946	MUY_04453
MUY_03946	MUY_00420
MUY_03946	MUY_00434
MUY_03946	MUY_00442
MUY_03946	MUY_00516
MUY_03946	MUY_00585
MUY_03946	MUY_00800
MUY_03946	MUY_00815
MUY_03946	MUY_00937
MUY_03946	MUY_00938
MUY_03946	MUY_00939
MUY_03946	MUY_00968
MUY_03946	MUY_00975
MUY_03946	MUY_00996
MUY_03946	MUY_01000
MUY_03949	MUY_03949
MUY_03949	MUY_00849
MUY_03950	MUY_03950
MUY_03950	MUY_03952
MUY_03950	MUY_03953
MUY_03951	MUY_03951
MUY_03951	MUY_04473
MUY_03952	MUY_03952
MUY_03952	MUY_03953
MUY_03953	MUY_03953
MUY_00371	MUY_00371
MUY_00371	MUY_00702
MUY_03955	MUY_03955
MUY_03956	MUY_03956
MUY_03956	MUY_03957
MUY_03960	MUY_03960
MUY_03960	MUY_04217
MUY_03961	MUY_03961
MUY_03961	MUY_03976
MUY_03961	MUY_04153

MUY_03961	MUY_04218
MUY_03961	MUY_04395
MUY_03962	MUY_03962
MUY_03962	MUY_03975
MUY_03962	MUY_04082
MUY_03962	MUY_04328
MUY_03962	MUY_04428
MUY_03962	MUY_00700
MUY_03963	MUY_03963
MUY_03963	MUY_04396
MUY_03964	MUY_03964
MUY_03964	MUY_03998
MUY_03964	MUY_04038
MUY_03964	MUY_04045
MUY_03964	MUY_00486
MUY_03964	MUY_00681
MUY_03964	MUY_00721
MUY_03964	MUY_00933
MUY_03965	MUY_03965
MUY_03965	MUY_00554
MUY_00372	MUY_00372
MUY_00372	MUY_03978
MUY_00372	MUY_04144
MUY_00372	MUY_04172
MUY_00372	MUY_04248
MUY_00372	MUY_04254
MUY_00372	MUY_04266
MUY_00372	MUY_04269
MUY_00372	MUY_04282
MUY_00372	MUY_04296
MUY_00372	MUY_00388
MUY_00372	MUY_04348
MUY_00372	MUY_04357
MUY_00372	MUY_00394
MUY_00372	MUY_04403
MUY_00372	MUY_04416
MUY_00372	MUY_04445
MUY_00372	MUY_04453
MUY_00372	MUY_00420
MUY_00372	MUY_00434
MUY_00372	MUY_00442
MUY_00372	MUY_00516
MUY_00372	MUY_00585
MUY_00372	MUY_00800
MUY_00372	MUY_00815
MUY_00372	MUY_00937
MUY_00372	MUY_00938

MUY_00372	MUY_00939
MUY_00372	MUY_00968
MUY_00372	MUY_00975
MUY_00372	MUY_00996
MUY_00372	MUY_01000
MUY_03967	MUY_04334
MUY_03967	MUY_00435
MUY_03967	MUY_00590
MUY_03967	MUY_00780
MUY_03968	MUY_03968
MUY_03968	MUY_00492
MUY_03969	MUY_03969
MUY_03969	MUY_03995
MUY_03969	MUY_04080
MUY_03969	MUY_04404
MUY_03969	MUY_00474
MUY_03970	MUY_03970
MUY_03974	MUY_03974
MUY_03974	MUY_00525
MUY_03975	MUY_03975
MUY_03975	MUY_04082
MUY_03975	MUY_04328
MUY_03975	MUY_04428
MUY_03975	MUY_00700
MUY_03976	MUY_03976
MUY_03976	MUY_04153
MUY_03976	MUY_04218
MUY_03976	MUY_04395
MUY_03977	MUY_03977
MUY_03978	MUY_03978
MUY_03978	MUY_04144
MUY_03978	MUY_04172
MUY_03978	MUY_04248
MUY_03978	MUY_04254
MUY_03978	MUY_04266
MUY_03978	MUY_04269
MUY_03978	MUY_04282
MUY_03978	MUY_04296
MUY_03978	MUY_00388
MUY_03978	MUY_04348
MUY_03978	MUY_04357
MUY_03978	MUY_00394
MUY_03978	MUY_04403
MUY_03978	MUY_04416
MUY_03978	MUY_04445
MUY_03978	MUY_04453
MUY_03978	MUY_00420

MUY_03978	MUY_00434
MUY_03978	MUY_00442
MUY_03978	MUY_00516
MUY_03978	MUY_00585
MUY_03978	MUY_00800
MUY_03978	MUY_00815
MUY_03978	MUY_00937
MUY_03978	MUY_00938
MUY_03978	MUY_00939
MUY_03978	MUY_00968
MUY_03978	MUY_00975
MUY_03978	MUY_00996
MUY_03978	MUY_01000
MUY_03980	MUY_03980
MUY_03980	MUY_04043
MUY_03980	MUY_00722
MUY_03982	MUY_03982
MUY_03983	MUY_03983
MUY_03983	MUY_04394
MUY_03983	MUY_00501
MUY_03984	MUY_03984
MUY_03984	MUY_04198
MUY_03984	MUY_00401
MUY_03984	MUY_00563
MUY_03985	MUY_03985
MUY_03986	MUY_03986
MUY_03986	MUY_04376
MUY_03986	MUY_00495
MUY_03989	MUY_03989
MUY_03995	MUY_03995
MUY_03995	MUY_04080
MUY_03995	MUY_04404
MUY_03995	MUY_00474
MUY_03996	MUY_00780
MUY_03997	MUY_03997
MUY_03997	MUY_04094
MUY_03998	MUY_03998
MUY_03998	MUY_04038
MUY_03998	MUY_04045
MUY_03998	MUY_00486
MUY_03998	MUY_00681
MUY_03998	MUY_00721
MUY_03998	MUY_00933
MUY_03999	MUY_03999
MUY_00376	MUY_00376
MUY_00376	MUY_04034
MUY_00376	MUY_04036

MUY_00376	MUY_00377
MUY_00376	MUY_00378
MUY_00376	MUY_04228
MUY_00376	MUY_04230
MUY_00376	MUY_00379
MUY_04004	MUY_04004
MUY_04004	MUY_04495
MUY_04007	MUY_04007
MUY_04008	MUY_04008
MUY_04008	MUY_00378
MUY_04008	MUY_04230
MUY_04008	MUY_00466
MUY_04011	MUY_04011
MUY_04012	MUY_04012
MUY_04014	MUY_04014
MUY_04014	MUY_04194
MUY_04014	MUY_00556
MUY_04014	MUY_00710
MUY_04017	MUY_04017
MUY_04018	MUY_04018
MUY_04020	MUY_04020
MUY_04020	MUY_04022
MUY_04022	MUY_04022
MUY_04023	MUY_04023
MUY_04024	MUY_04024
MUY_04024	MUY_04324
MUY_04025	MUY_04025
MUY_04026	MUY_04026
MUY_04026	MUY_04258
MUY_04026	MUY_04322
MUY_04026	MUY_04504
MUY_04026	MUY_00541
MUY_04026	MUY_00571
MUY_04026	MUY_00628
MUY_04027	MUY_04027
MUY_04028	MUY_04028
MUY_04033	MUY_04033
MUY_04033	MUY_04036
MUY_04033	MUY_04228
MUY_04034	MUY_04034
MUY_04034	MUY_04036
MUY_04034	MUY_00377
MUY_04034	MUY_00378
MUY_04034	MUY_04228
MUY_04034	MUY_04230
MUY_04034	MUY_00379
MUY_04034	MUY_00780

MUY_04035	MUY_04035
MUY_04036	MUY_04036
MUY_04036	MUY_00377
MUY_04036	MUY_00378
MUY_04036	MUY_04228
MUY_04036	MUY_04230
MUY_04036	MUY_00379
MUY_04037	MUY_04037
MUY_04037	MUY_00730
MUY_04037	MUY_00080
MUY_04037	MUY_00081
MUY_04038	MUY_04038
MUY_04038	MUY_00486
MUY_04038	MUY_00681
MUY_04038	MUY_00933
MUY_04039	MUY_04039
MUY_04039	MUY_00384
MUY_04042	MUY_04042
MUY_04042	MUY_04408
MUY_04042	MUY_00443
MUY_04043	MUY_04043
MUY_04043	MUY_00385
MUY_04043	MUY_04343
MUY_04043	MUY_00722
MUY_04044	MUY_00435
MUY_04045	MUY_04045
MUY_04045	MUY_00486
MUY_04045	MUY_00681
MUY_04045	MUY_00721
MUY_04046	MUY_04046
MUY_04049	MUY_04049
MUY_04049	MUY_00810
MUY_04049	MUY_00869
MUY_04050	MUY_04050
MUY_04052	MUY_04052
MUY_04052	MUY_00460
MUY_04053	MUY_04053
MUY_04053	MUY_04054
MUY_04053	MUY_00071
MUY_04054	MUY_04054
MUY_04054	MUY_00071
MUY_04057	MUY_00780
MUY_04060	MUY_04060
MUY_04060	MUY_04510
MUY_04061	MUY_04061
MUY_04061	MUY_04064
MUY_04061	MUY_04510

MUY_04062	MUY_04510
MUY_04063	MUY_04063
MUY_04063	MUY_04510
MUY_04064	MUY_04064
MUY_04064	MUY_04510
MUY_04065	MUY_04510
MUY_04066	MUY_04510
MUY_04067	MUY_04510
MUY_04069	MUY_04069
MUY_04070	MUY_04070
MUY_04070	MUY_04242
MUY_04070	MUY_00382
MUY_04070	MUY_04302
MUY_04070	MUY_04383
MUY_04070	MUY_04433
MUY_04070	MUY_04483
MUY_04070	MUY_00450
MUY_04070	MUY_00464
MUY_04070	MUY_00808
MUY_04070	MUY_00840
MUY_04071	MUY_04071
MUY_04072	MUY_04072
MUY_04073	MUY_04073
MUY_04073	MUY_00523
MUY_04073	MUY_00861
MUY_04075	MUY_04075
MUY_04075	MUY_00829
MUY_04079	MUY_04079
MUY_04079	MUY_04391
MUY_04079	MUY_00852
MUY_04080	MUY_04080
MUY_04080	MUY_04404
MUY_04080	MUY_00474
MUY_04081	MUY_04081
MUY_04081	MUY_00845
MUY_04081	MUY_00851
MUY_04082	MUY_04082
MUY_04082	MUY_04328
MUY_04082	MUY_04428
MUY_04082	MUY_00700
MUY_04083	MUY_04083
MUY_04085	MUY_04085
MUY_04085	MUY_04206
MUY_04085	MUY_04410
MUY_04085	MUY_00695
MUY_04085	MUY_00906
MUY_04086	MUY_04086

MUY_04086	MUY_04334
MUY_04086	MUY_04466
MUY_04086	MUY_00433
MUY_04086	MUY_00435
MUY_04086	MUY_00590
MUY_04086	MUY_00068
MUY_04086	MUY_00780
MUY_04086	MUY_00075
MUY_04086	MUY_00813
MUY_04089	MUY_04089
MUY_04090	MUY_04090
MUY_04090	MUY_04486
MUY_04091	MUY_04091
MUY_04091	MUY_00420
MUY_04091	MUY_00830
MUY_04091	MUY_00998
MUY_04094	MUY_04094
MUY_04095	MUY_04095
MUY_04095	MUY_04388
MUY_04096	MUY_04096
MUY_04096	MUY_04146
MUY_04096	MUY_04177
MUY_04096	MUY_04211
MUY_04096	MUY_04212
MUY_04096	MUY_04293
MUY_04096	MUY_04294
MUY_04096	MUY_04358
MUY_04096	MUY_04359
MUY_04096	MUY_04379
MUY_04096	MUY_04417
MUY_04096	MUY_04418
MUY_04096	MUY_04447
MUY_04096	MUY_04448
MUY_04096	MUY_04471
MUY_04096	MUY_00418
MUY_04096	MUY_00436
MUY_04096	MUY_00575
MUY_04096	MUY_00576
MUY_04096	MUY_00790
MUY_04096	MUY_00961
MUY_04097	MUY_04097
MUY_04098	MUY_04098
MUY_04098	MUY_04304
MUY_04099	MUY_04099
MUY_04099	MUY_04305
MUY_04099	MUY_00630
MUY_04100	MUY_04100

MUY_04100	MUY_00730
MUY_04100	MUY_00081
MUY_04103	MUY_04103
MUY_04103	MUY_04104
MUY_04103	MUY_00519
MUY_04104	MUY_04104
MUY_04104	MUY_00519
MUY_04105	MUY_04105
MUY_04106	MUY_04106
MUY_04106	MUY_00998
MUY_04108	MUY_04108
MUY_04110	MUY_04110
MUY_00377	MUY_00377
MUY_00377	MUY_00378
MUY_00377	MUY_04228
MUY_00377	MUY_04230
MUY_00377	MUY_00379
MUY_04112	MUY_04112
MUY_04112	MUY_00400
MUY_04112	MUY_00559
MUY_04113	MUY_04113
MUY_04116	MUY_04116
MUY_04119	MUY_04119
MUY_04119	MUY_04130
MUY_04122	MUY_04122
MUY_04126	MUY_04126
MUY_04126	MUY_04239
MUY_04126	MUY_00469
MUY_04126	MUY_00485
MUY_04126	MUY_00696
MUY_04127	MUY_04127
MUY_04129	MUY_04129
MUY_04130	MUY_04130
MUY_04131	MUY_04131
MUY_04132	MUY_04132
MUY_04132	MUY_04150
MUY_04132	MUY_04397
MUY_04132	MUY_04438
MUY_04132	MUY_00453
MUY_04132	MUY_00797
MUY_04134	MUY_04134
MUY_04134	MUY_04219
MUY_04134	MUY_00480
MUY_04135	MUY_04135
MUY_04136	MUY_04136
MUY_04136	MUY_00475
MUY_04136	MUY_00709

MUY_04137	MUY_04137
MUY_04138	MUY_00435
MUY_04144	MUY_04144
MUY_04144	MUY_04172
MUY_04144	MUY_04248
MUY_04144	MUY_04254
MUY_04144	MUY_04266
MUY_04144	MUY_04269
MUY_04144	MUY_04282
MUY_04144	MUY_04296
MUY_04144	MUY_00388
MUY_04144	MUY_04348
MUY_04144	MUY_04357
MUY_04144	MUY_00394
MUY_04144	MUY_04403
MUY_04144	MUY_04416
MUY_04144	MUY_04445
MUY_04144	MUY_04453
MUY_04144	MUY_00420
MUY_04144	MUY_00434
MUY_04144	MUY_00442
MUY_04144	MUY_00516
MUY_04144	MUY_00585
MUY_04144	MUY_00800
MUY_04144	MUY_00815
MUY_04144	MUY_00937
MUY_04144	MUY_00938
MUY_04144	MUY_00939
MUY_04144	MUY_00968
MUY_04144	MUY_00975
MUY_04144	MUY_00996
MUY_04144	MUY_01000
MUY_04145	MUY_04145
MUY_04145	MUY_04211
MUY_04145	MUY_04294
MUY_04145	MUY_04358
MUY_04145	MUY_04447
MUY_04146	MUY_04146
MUY_04146	MUY_04177
MUY_04146	MUY_04211
MUY_04146	MUY_04212
MUY_04146	MUY_04293
MUY_04146	MUY_04294
MUY_04146	MUY_04358
MUY_04146	MUY_04359
MUY_04146	MUY_04379
MUY_04146	MUY_04417

MUY_04146	MUY_04418
MUY_04146	MUY_04447
MUY_04146	MUY_04448
MUY_04146	MUY_04471
MUY_04146	MUY_00418
MUY_04146	MUY_00436
MUY_04146	MUY_00575
MUY_04146	MUY_00576
MUY_04146	MUY_00790
MUY_04146	MUY_00961
MUY_04150	MUY_04150
MUY_04150	MUY_04397
MUY_04150	MUY_04438
MUY_04150	MUY_00453
MUY_04150	MUY_00797
MUY_04151	MUY_04151
MUY_04152	MUY_04152
MUY_04152	MUY_04334
MUY_04152	MUY_00927
MUY_04153	MUY_04153
MUY_04153	MUY_04218
MUY_04153	MUY_04395
MUY_04155	MUY_04155
MUY_04155	MUY_04326
MUY_04156	MUY_04156
MUY_04156	MUY_04194
MUY_04156	MUY_04345
MUY_04156	MUY_04361
MUY_04156	MUY_04485
MUY_04156	MUY_00787
MUY_04156	MUY_00838
MUY_04156	MUY_00886
MUY_04157	MUY_04157
MUY_04157	MUY_04346
MUY_04157	MUY_00789
MUY_04159	MUY_04159
MUY_04161	MUY_04161
MUY_04161	MUY_00688
MUY_04161	MUY_00971
MUY_04163	MUY_04163
MUY_04163	MUY_04439
MUY_04163	MUY_00447
MUY_04163	MUY_00856
MUY_04163	MUY_00917
MUY_04163	MUY_00945
MUY_04164	MUY_04164
MUY_04171	MUY_04171

MUY_04171	MUY_04318
MUY_04171	MUY_04410
MUY_04171	MUY_00504
MUY_04171	MUY_00782
MUY_04171	MUY_00920
MUY_04172	MUY_04172
MUY_04172	MUY_04248
MUY_04172	MUY_04254
MUY_04172	MUY_04266
MUY_04172	MUY_04269
MUY_04172	MUY_04282
MUY_04172	MUY_04296
MUY_04172	MUY_00388
MUY_04172	MUY_04348
MUY_04172	MUY_04357
MUY_04172	MUY_00394
MUY_04172	MUY_04403
MUY_04172	MUY_04416
MUY_04172	MUY_04445
MUY_04172	MUY_04453
MUY_04172	MUY_00420
MUY_04172	MUY_00434
MUY_04172	MUY_00442
MUY_04172	MUY_00516
MUY_04172	MUY_00585
MUY_04172	MUY_00800
MUY_04172	MUY_00815
MUY_04172	MUY_00937
MUY_04172	MUY_00938
MUY_04172	MUY_00939
MUY_04172	MUY_00968
MUY_04172	MUY_00975
MUY_04172	MUY_00996
MUY_04172	MUY_01000
MUY_04174	MUY_04174
MUY_04174	MUY_00074
MUY_04177	MUY_04177
MUY_04177	MUY_04211
MUY_04177	MUY_04212
MUY_04177	MUY_04293
MUY_04177	MUY_04294
MUY_04177	MUY_04358
MUY_04177	MUY_04359
MUY_04177	MUY_04379
MUY_04177	MUY_04417
MUY_04177	MUY_04418
MUY_04177	MUY_04447

MUY_04177	MUY_04448
MUY_04177	MUY_04471
MUY_04177	MUY_00418
MUY_04177	MUY_00436
MUY_04177	MUY_00575
MUY_04177	MUY_00576
MUY_04177	MUY_00790
MUY_04177	MUY_00961
MUY_04179	MUY_04182
MUY_04180	MUY_04182
MUY_04181	MUY_04182
MUY_04182	MUY_04182
MUY_04182	MUY_04402
MUY_04182	MUY_04432
MUY_04183	MUY_04183
MUY_04183	MUY_04335
MUY_04183	MUY_04430
MUY_04183	MUY_00847
MUY_04183	MUY_00921
MUY_04185	MUY_04185
MUY_04185	MUY_04271
MUY_04185	MUY_04291
MUY_04185	MUY_00551
MUY_04185	MUY_00599
MUY_04185	MUY_00602
MUY_04185	MUY_00691
MUY_04185	MUY_00795
MUY_04185	MUY_00086
MUY_04185	MUY_00973
MUY_04188	MUY_04188
MUY_04188	MUY_00993
MUY_04191	MUY_04191
MUY_04192	MUY_04192
MUY_04192	MUY_04434
MUY_04193	MUY_04193
MUY_04193	MUY_04389
MUY_04193	MUY_04390
MUY_04193	MUY_04393
MUY_04194	MUY_04194
MUY_04194	MUY_04345
MUY_04194	MUY_04361
MUY_04194	MUY_04485
MUY_04194	MUY_00556
MUY_04194	MUY_00710
MUY_04194	MUY_00787
MUY_04194	MUY_00838
MUY_04194	MUY_00886

MUY_04196	MUY_04196
MUY_04196	MUY_00892
MUY_04197	MUY_04197
MUY_04198	MUY_04198
MUY_04198	MUY_00401
MUY_04198	MUY_00563
MUY_04204	MUY_04204
MUY_04205	MUY_04494
MUY_04205	MUY_00979
MUY_04206	MUY_04206
MUY_04206	MUY_00695
MUY_04206	MUY_00906
MUY_04207	MUY_04207
MUY_04211	MUY_04211
MUY_04211	MUY_04212
MUY_04211	MUY_04293
MUY_04211	MUY_04294
MUY_04211	MUY_04358
MUY_04211	MUY_04359
MUY_04211	MUY_00393
MUY_04211	MUY_04402
MUY_04211	MUY_04417
MUY_04211	MUY_04418
MUY_04211	MUY_04420
MUY_04211	MUY_04447
MUY_04211	MUY_04448
MUY_04211	MUY_04470
MUY_04211	MUY_04471
MUY_04211	MUY_00418
MUY_04211	MUY_00436
MUY_04211	MUY_00438
MUY_04211	MUY_00575
MUY_04211	MUY_00576
MUY_04212	MUY_04212
MUY_04212	MUY_04293
MUY_04212	MUY_04294
MUY_04212	MUY_04358
MUY_04212	MUY_04359
MUY_04212	MUY_04379
MUY_04212	MUY_04417
MUY_04212	MUY_04418
MUY_04212	MUY_04447
MUY_04212	MUY_04448
MUY_04212	MUY_04471
MUY_04212	MUY_00418
MUY_04212	MUY_00436
MUY_04212	MUY_00575

MUY_04212	MUY_00576
MUY_04212	MUY_00790
MUY_04212	MUY_00961
MUY_00378	MUY_00378
MUY_00378	MUY_04228
MUY_00378	MUY_04230
MUY_00378	MUY_00379
MUY_00378	MUY_00380
MUY_00378	MUY_04492
MUY_00378	MUY_00466
MUY_00378	MUY_00526
MUY_00378	MUY_00535
MUY_00378	MUY_00536
MUY_00378	MUY_00539
MUY_00378	MUY_00750
MUY_00378	MUY_00811
MUY_04216	MUY_04216
MUY_04216	MUY_04395
MUY_04217	MUY_04217
MUY_04218	MUY_04218
MUY_04218	MUY_04395
MUY_04219	MUY_04219
MUY_04219	MUY_00480
MUY_04220	MUY_04220
MUY_04228	MUY_04228
MUY_04228	MUY_04230
MUY_04228	MUY_00379
MUY_04230	MUY_04230
MUY_04230	MUY_00379
MUY_04230	MUY_00380
MUY_04230	MUY_04492
MUY_04230	MUY_00466
MUY_04230	MUY_00526
MUY_04230	MUY_00535
MUY_04230	MUY_00536
MUY_04230	MUY_00539
MUY_04230	MUY_00750
MUY_04230	MUY_00811
MUY_04231	MUY_04231
MUY_04232	MUY_04232
MUY_00379	MUY_00379
MUY_04235	MUY_04235
MUY_04235	MUY_00899
MUY_04237	MUY_04237
MUY_04238	MUY_00483
MUY_04239	MUY_04239
MUY_04239	MUY_00469

MUY_04239	MUY_00485
MUY_04239	MUY_00696
MUY_04241	MUY_04241
MUY_04242	MUY_04242
MUY_04242	MUY_00382
MUY_04242	MUY_04302
MUY_04242	MUY_04383
MUY_04242	MUY_04433
MUY_04242	MUY_04483
MUY_04242	MUY_00450
MUY_04242	MUY_00464
MUY_04242	MUY_00808
MUY_04242	MUY_00840
MUY_04243	MUY_04243
MUY_04244	MUY_04244
MUY_04244	MUY_00998
MUY_04245	MUY_04245
MUY_04245	MUY_04281
MUY_04245	MUY_04352
MUY_04247	MUY_04247
MUY_04248	MUY_04248
MUY_04248	MUY_04254
MUY_04248	MUY_04266
MUY_04248	MUY_04269
MUY_04248	MUY_04282
MUY_04248	MUY_04296
MUY_04248	MUY_00388
MUY_04248	MUY_04348
MUY_04248	MUY_04357
MUY_04248	MUY_00394
MUY_04248	MUY_04403
MUY_04248	MUY_04416
MUY_04248	MUY_04445
MUY_04248	MUY_04453
MUY_04248	MUY_00420
MUY_04248	MUY_00434
MUY_04248	MUY_00442
MUY_04248	MUY_00516
MUY_04248	MUY_00585
MUY_04248	MUY_00800
MUY_04248	MUY_00815
MUY_04248	MUY_00937
MUY_04248	MUY_00938
MUY_04248	MUY_00939
MUY_04248	MUY_00968
MUY_04248	MUY_00975
MUY_04248	MUY_00996

MUY_04248	MUY_01000
MUY_04249	MUY_04249
MUY_04249	MUY_00481
MUY_04249	MUY_00573
MUY_04254	MUY_04254
MUY_04254	MUY_04266
MUY_04254	MUY_04269
MUY_04254	MUY_04282
MUY_04254	MUY_04296
MUY_04254	MUY_00388
MUY_04254	MUY_04348
MUY_04254	MUY_04357
MUY_04254	MUY_00394
MUY_04254	MUY_04403
MUY_04254	MUY_04416
MUY_04254	MUY_04445
MUY_04254	MUY_04453
MUY_04254	MUY_00420
MUY_04254	MUY_00434
MUY_04254	MUY_00442
MUY_04254	MUY_00516
MUY_04254	MUY_00585
MUY_04254	MUY_00800
MUY_04254	MUY_00815
MUY_04254	MUY_00937
MUY_04254	MUY_00938
MUY_04254	MUY_00939
MUY_04254	MUY_00968
MUY_04254	MUY_00975
MUY_04254	MUY_00996
MUY_04254	MUY_01000
MUY_04257	MUY_04258
MUY_04258	MUY_04258
MUY_04258	MUY_04322
MUY_04258	MUY_04504
MUY_04258	MUY_00541
MUY_04258	MUY_00571
MUY_04258	MUY_00628
MUY_04260	MUY_04260
MUY_04260	MUY_04262
MUY_04260	MUY_04342
MUY_04260	MUY_00892
MUY_04262	MUY_04262
MUY_04262	MUY_04342
MUY_04262	MUY_00892
MUY_04263	MUY_04263
MUY_04263	MUY_00946

MUY_04264	MUY_04264
MUY_04266	MUY_04266
MUY_04266	MUY_04269
MUY_04266	MUY_04282
MUY_04266	MUY_04296
MUY_04266	MUY_00388
MUY_04266	MUY_04348
MUY_04266	MUY_04357
MUY_04266	MUY_00394
MUY_04266	MUY_04403
MUY_04266	MUY_04416
MUY_04266	MUY_04445
MUY_04266	MUY_04453
MUY_04266	MUY_00420
MUY_04266	MUY_00434
MUY_04266	MUY_00442
MUY_04266	MUY_00516
MUY_04266	MUY_00585
MUY_04266	MUY_00800
MUY_04266	MUY_00815
MUY_04266	MUY_00937
MUY_04266	MUY_00938
MUY_04266	MUY_00939
MUY_04266	MUY_00968
MUY_04266	MUY_00975
MUY_04266	MUY_00996
MUY_04266	MUY_01000
MUY_04269	MUY_04269
MUY_04269	MUY_04282
MUY_04269	MUY_04296
MUY_04269	MUY_00388
MUY_04269	MUY_04348
MUY_04269	MUY_04357
MUY_04269	MUY_00394
MUY_04269	MUY_04403
MUY_04269	MUY_04416
MUY_04269	MUY_04445
MUY_04269	MUY_04453
MUY_04269	MUY_00420
MUY_04269	MUY_00434
MUY_04269	MUY_00442
MUY_04269	MUY_00516
MUY_04269	MUY_00585
MUY_04269	MUY_00800
MUY_04269	MUY_00815
MUY_04269	MUY_00937
MUY_04269	MUY_00938

MUY_04269	MUY_00939
MUY_04269	MUY_00968
MUY_04269	MUY_00975
MUY_04269	MUY_00996
MUY_04269	MUY_01000
MUY_04271	MUY_04271
MUY_04271	MUY_04291
MUY_04271	MUY_04501
MUY_04271	MUY_04505
MUY_04271	MUY_00551
MUY_04271	MUY_00599
MUY_04271	MUY_00602
MUY_04271	MUY_00628
MUY_04271	MUY_00691
MUY_04271	MUY_00795
MUY_04271	MUY_00086
MUY_04271	MUY_00973
MUY_00380	MUY_00380
MUY_04280	MUY_04280
MUY_04281	MUY_04282
MUY_00381	MUY_00780
MUY_04282	MUY_04282
MUY_04282	MUY_04296
MUY_04282	MUY_00388
MUY_04282	MUY_04348
MUY_04282	MUY_04357
MUY_04282	MUY_00394
MUY_04282	MUY_04403
MUY_04282	MUY_04416
MUY_04282	MUY_04445
MUY_04282	MUY_04453
MUY_04282	MUY_00420
MUY_04282	MUY_00434
MUY_04282	MUY_00442
MUY_04282	MUY_00516
MUY_04282	MUY_00585
MUY_04282	MUY_00800
MUY_04282	MUY_00815
MUY_04282	MUY_00937
MUY_04282	MUY_00938
MUY_04282	MUY_00939
MUY_04282	MUY_00968
MUY_04282	MUY_00975
MUY_04282	MUY_00996
MUY_04282	MUY_01000
MUY_04285	MUY_04285
MUY_04287	MUY_04287

MUY_04289	MUY_04289
MUY_04289	MUY_00507
MUY_04289	MUY_00738
MUY_00382	MUY_00382
MUY_00382	MUY_04302
MUY_00382	MUY_04383
MUY_00382	MUY_04433
MUY_00382	MUY_04483
MUY_00382	MUY_00450
MUY_00382	MUY_00464
MUY_00382	MUY_00808
MUY_00382	MUY_00840
MUY_04290	MUY_04290
MUY_04290	MUY_00775
MUY_04291	MUY_04291
MUY_04291	MUY_04501
MUY_04291	MUY_04505
MUY_04291	MUY_00551
MUY_04291	MUY_00599
MUY_04291	MUY_00602
MUY_04291	MUY_00628
MUY_04291	MUY_00691
MUY_04291	MUY_00795
MUY_04291	MUY_00086
MUY_04291	MUY_00973
MUY_04292	MUY_04292
MUY_04293	MUY_04293
MUY_04293	MUY_04294
MUY_04293	MUY_04358
MUY_04293	MUY_04359
MUY_04293	MUY_04379
MUY_04293	MUY_04417
MUY_04293	MUY_04418
MUY_04293	MUY_04447
MUY_04293	MUY_04448
MUY_04293	MUY_04471
MUY_04293	MUY_00418
MUY_04293	MUY_00436
MUY_04293	MUY_00575
MUY_04293	MUY_00576
MUY_04293	MUY_00790
MUY_04293	MUY_00961
MUY_04294	MUY_04294
MUY_04294	MUY_04358
MUY_04294	MUY_04359
MUY_04294	MUY_00393
MUY_04294	MUY_04402

MUY_04294	MUY_04417
MUY_04294	MUY_04418
MUY_04294	MUY_04420
MUY_04294	MUY_04447
MUY_04294	MUY_04448
MUY_04294	MUY_04470
MUY_04294	MUY_04471
MUY_04294	MUY_00418
MUY_04294	MUY_00436
MUY_04294	MUY_00438
MUY_04294	MUY_00575
MUY_04294	MUY_00576
MUY_04296	MUY_04296
MUY_04296	MUY_00388
MUY_04296	MUY_04348
MUY_04296	MUY_04357
MUY_04296	MUY_00394
MUY_04296	MUY_04403
MUY_04296	MUY_04416
MUY_04296	MUY_04445
MUY_04296	MUY_04453
MUY_04296	MUY_00420
MUY_04296	MUY_00434
MUY_04296	MUY_00442
MUY_04296	MUY_00516
MUY_04296	MUY_00585
MUY_04296	MUY_00800
MUY_04296	MUY_00815
MUY_04296	MUY_00937
MUY_04296	MUY_00938
MUY_04296	MUY_00939
MUY_04296	MUY_00968
MUY_04296	MUY_00975
MUY_04296	MUY_00996
MUY_04296	MUY_01000
MUY_04301	MUY_04301
MUY_04301	MUY_00698
MUY_04301	MUY_00871
MUY_04302	MUY_04302
MUY_04302	MUY_04383
MUY_04302	MUY_04433
MUY_04302	MUY_04483
MUY_04302	MUY_00450
MUY_04302	MUY_00464
MUY_04302	MUY_00808
MUY_04302	MUY_00840
MUY_04303	MUY_04303

MUY_04303	MUY_04381
MUY_04304	MUY_04304
MUY_04305	MUY_04305
MUY_04305	MUY_00630
MUY_04307	MUY_04307
MUY_04307	MUY_04400
MUY_00384	MUY_00384
MUY_04312	MUY_04312
MUY_04312	MUY_00561
MUY_04312	MUY_00734
MUY_04313	MUY_04313
MUY_04314	MUY_04314
MUY_04315	MUY_04315
MUY_04317	MUY_04317
MUY_04317	MUY_00439
MUY_04318	MUY_04318
MUY_04318	MUY_00397
MUY_04318	MUY_00504
MUY_04318	MUY_00782
MUY_04318	MUY_00072
MUY_04318	MUY_00920
MUY_00385	MUY_00385
MUY_00385	MUY_04343
MUY_00385	MUY_00722
MUY_04322	MUY_04322
MUY_04322	MUY_04504
MUY_04322	MUY_00541
MUY_04322	MUY_00571
MUY_04322	MUY_00628
MUY_04324	MUY_04324
MUY_04326	MUY_04326
MUY_04327	MUY_04327
MUY_04328	MUY_04328
MUY_04328	MUY_04428
MUY_04328	MUY_00700
MUY_04329	MUY_04329
MUY_04330	MUY_00572
MUY_04334	MUY_04334
MUY_04334	MUY_04466
MUY_04334	MUY_00435
MUY_04334	MUY_00514
MUY_04334	MUY_00068
MUY_04334	MUY_00780
MUY_04334	MUY_00927
MUY_04335	MUY_04335
MUY_04335	MUY_04430
MUY_04335	MUY_00847

MUY_04335	MUY_00921
MUY_04337	MUY_04337
MUY_04337	MUY_00708
MUY_00388	MUY_00388
MUY_00388	MUY_04348
MUY_00388	MUY_04357
MUY_00388	MUY_00394
MUY_00388	MUY_04403
MUY_00388	MUY_04416
MUY_00388	MUY_04445
MUY_00388	MUY_04453
MUY_00388	MUY_00420
MUY_00388	MUY_00434
MUY_00388	MUY_00442
MUY_00388	MUY_00516
MUY_00388	MUY_00585
MUY_00388	MUY_00800
MUY_00388	MUY_00815
MUY_00388	MUY_00937
MUY_00388	MUY_00938
MUY_00388	MUY_00939
MUY_00388	MUY_00968
MUY_00388	MUY_00975
MUY_00388	MUY_00996
MUY_00388	MUY_01000
MUY_04342	MUY_04342
MUY_04343	MUY_04343
MUY_04343	MUY_00722
MUY_04345	MUY_04345
MUY_04345	MUY_04346
MUY_04345	MUY_04361
MUY_04345	MUY_04485
MUY_04345	MUY_00787
MUY_04345	MUY_00838
MUY_04345	MUY_00886
MUY_04346	MUY_04346
MUY_04346	MUY_00789
MUY_04348	MUY_04348
MUY_04348	MUY_04357
MUY_04348	MUY_00394
MUY_04348	MUY_04403
MUY_04348	MUY_04416
MUY_04348	MUY_04445
MUY_04348	MUY_04453
MUY_04348	MUY_00420
MUY_04348	MUY_00434
MUY_04348	MUY_00442

MUY_04348	MUY_00516
MUY_04348	MUY_00585
MUY_04348	MUY_00800
MUY_04348	MUY_00815
MUY_04348	MUY_00937
MUY_04348	MUY_00938
MUY_04348	MUY_00939
MUY_04348	MUY_00968
MUY_04348	MUY_00975
MUY_04348	MUY_00996
MUY_04348	MUY_01000
MUY_04350	MUY_04350
MUY_04352	MUY_04352
MUY_04353	MUY_04353
MUY_04357	MUY_04357
MUY_04357	MUY_00394
MUY_04357	MUY_04403
MUY_04357	MUY_04416
MUY_04357	MUY_04445
MUY_04357	MUY_04453
MUY_04357	MUY_00420
MUY_04357	MUY_00434
MUY_04357	MUY_00442
MUY_04357	MUY_00516
MUY_04357	MUY_00585
MUY_04357	MUY_00800
MUY_04357	MUY_00815
MUY_04357	MUY_00937
MUY_04357	MUY_00938
MUY_04357	MUY_00939
MUY_04357	MUY_00968
MUY_04357	MUY_00975
MUY_04357	MUY_00996
MUY_04357	MUY_01000
MUY_04358	MUY_04358
MUY_04358	MUY_04359
MUY_04358	MUY_00393
MUY_04358	MUY_04402
MUY_04358	MUY_04417
MUY_04358	MUY_04418
MUY_04358	MUY_04420
MUY_04358	MUY_04447
MUY_04358	MUY_04448
MUY_04358	MUY_04470
MUY_04358	MUY_04471
MUY_04358	MUY_00418
MUY_04358	MUY_00436

MUY_04358	MUY_00438
MUY_04358	MUY_00575
MUY_04358	MUY_00576
MUY_04359	MUY_04359
MUY_04359	MUY_04379
MUY_04359	MUY_04417
MUY_04359	MUY_04418
MUY_04359	MUY_04447
MUY_04359	MUY_04448
MUY_04359	MUY_04471
MUY_04359	MUY_00418
MUY_04359	MUY_00436
MUY_04359	MUY_00575
MUY_04359	MUY_00576
MUY_04359	MUY_00790
MUY_04359	MUY_00961
MUY_04361	MUY_04361
MUY_04361	MUY_04485
MUY_04361	MUY_00787
MUY_04361	MUY_00838
MUY_04361	MUY_00886
MUY_00392	MUY_00392
MUY_04364	MUY_04364
MUY_04367	MUY_04367
MUY_04367	MUY_00496
MUY_04367	MUY_00046
MUY_04367	MUY_00527
MUY_04367	MUY_00816
MUY_04373	MUY_04373
MUY_04376	MUY_04376
MUY_04376	MUY_00495
MUY_00393	MUY_00393
MUY_00393	MUY_04417
MUY_00393	MUY_04420
MUY_00393	MUY_04447
MUY_00393	MUY_04470
MUY_00393	MUY_00438
MUY_00393	MUY_00575
MUY_04379	MUY_04379
MUY_04379	MUY_04418
MUY_04379	MUY_04448
MUY_04379	MUY_04471
MUY_04379	MUY_00418
MUY_04379	MUY_00436
MUY_04379	MUY_00576
MUY_04381	MUY_04381
MUY_04383	MUY_04383

MUY_04383	MUY_04433
MUY_04383	MUY_04483
MUY_04383	MUY_00450
MUY_04383	MUY_00464
MUY_04383	MUY_00808
MUY_04383	MUY_00840
MUY_04385	MUY_04385
MUY_04388	MUY_04388
MUY_04389	MUY_04389
MUY_04389	MUY_04390
MUY_04389	MUY_04393
MUY_04390	MUY_04390
MUY_04390	MUY_04393
MUY_04391	MUY_04391
MUY_04391	MUY_00852
MUY_00394	MUY_00394
MUY_00394	MUY_04403
MUY_00394	MUY_04416
MUY_00394	MUY_04445
MUY_00394	MUY_04453
MUY_00394	MUY_00420
MUY_00394	MUY_00434
MUY_00394	MUY_00442
MUY_00394	MUY_00516
MUY_00394	MUY_00585
MUY_00394	MUY_00800
MUY_00394	MUY_00815
MUY_00394	MUY_00937
MUY_00394	MUY_00938
MUY_00394	MUY_00939
MUY_00394	MUY_00968
MUY_00394	MUY_00975
MUY_00394	MUY_00996
MUY_00394	MUY_01000
MUY_04393	MUY_04393
MUY_04394	MUY_04394
MUY_04394	MUY_00501
MUY_04395	MUY_04395
MUY_04396	MUY_04396
MUY_04397	MUY_04397
MUY_04397	MUY_04438
MUY_04397	MUY_00453
MUY_04397	MUY_00797
MUY_04399	MUY_04399
MUY_04400	MUY_04400
MUY_04402	MUY_04402
MUY_04402	MUY_04447

MUY_04403	MUY_04403
MUY_04403	MUY_04416
MUY_04403	MUY_04445
MUY_04403	MUY_04453
MUY_04403	MUY_00420
MUY_04403	MUY_00434
MUY_04403	MUY_00442
MUY_04403	MUY_00516
MUY_04403	MUY_00585
MUY_04403	MUY_00800
MUY_04403	MUY_00815
MUY_04403	MUY_00937
MUY_04403	MUY_00938
MUY_04403	MUY_00939
MUY_04403	MUY_00968
MUY_04403	MUY_00975
MUY_04403	MUY_00996
MUY_04403	MUY_01000
MUY_04404	MUY_04404
MUY_04404	MUY_00474
MUY_04405	MUY_04405
MUY_04406	MUY_04406
MUY_04408	MUY_04408
MUY_04408	MUY_00443
MUY_04410	MUY_04410
MUY_04410	MUY_00504
MUY_04410	MUY_00695
MUY_04410	MUY_00782
MUY_04410	MUY_00906
MUY_04410	MUY_00920
MUY_04412	MUY_04412
MUY_04413	MUY_04413
MUY_00397	MUY_00397
MUY_04416	MUY_04416
MUY_04416	MUY_04445
MUY_04416	MUY_04453
MUY_04416	MUY_00420
MUY_04416	MUY_00434
MUY_04416	MUY_00442
MUY_04416	MUY_00516
MUY_04416	MUY_00585
MUY_04416	MUY_00800
MUY_04416	MUY_00815
MUY_04416	MUY_00937
MUY_04416	MUY_00938
MUY_04416	MUY_00939
MUY_04416	MUY_00968

MUY_04416	MUY_00975
MUY_04416	MUY_00996
MUY_04416	MUY_01000
MUY_04417	MUY_04417
MUY_04417	MUY_04418
MUY_04417	MUY_04420
MUY_04417	MUY_04447
MUY_04417	MUY_04448
MUY_04417	MUY_04470
MUY_04417	MUY_04471
MUY_04417	MUY_00418
MUY_04417	MUY_00436
MUY_04417	MUY_00438
MUY_04417	MUY_00575
MUY_04417	MUY_00576
MUY_00032	MUY_00032
MUY_04418	MUY_04418
MUY_04418	MUY_04447
MUY_04418	MUY_04448
MUY_04418	MUY_04471
MUY_04418	MUY_00418
MUY_04418	MUY_00436
MUY_04418	MUY_00575
MUY_04418	MUY_00576
MUY_04418	MUY_00790
MUY_04418	MUY_00961
MUY_04420	MUY_04420
MUY_04420	MUY_04447
MUY_04420	MUY_04470
MUY_04420	MUY_00438
MUY_04420	MUY_00575
MUY_04422	MUY_04422
MUY_04424	MUY_04424
MUY_04424	MUY_00424
MUY_04425	MUY_04427
MUY_00398	MUY_00398
MUY_00398	MUY_00451
MUY_00398	MUY_00807
MUY_00398	MUY_00809
MUY_00398	MUY_00944
MUY_04428	MUY_04428
MUY_04428	MUY_00700
MUY_04429	MUY_04429
MUY_04429	MUY_00052
MUY_04430	MUY_04430
MUY_04430	MUY_00847
MUY_04430	MUY_00921

MUY_04431	MUY_04431
MUY_04432	MUY_04432
MUY_04432	MUY_04482
MUY_04432	MUY_00423
MUY_04433	MUY_04433
MUY_04433	MUY_04483
MUY_04433	MUY_00450
MUY_04433	MUY_00464
MUY_04433	MUY_00808
MUY_04433	MUY_00840
MUY_04434	MUY_04434
MUY_04436	MUY_04436
MUY_04438	MUY_04438
MUY_04438	MUY_04482
MUY_04438	MUY_00453
MUY_04438	MUY_00583
MUY_04438	MUY_00797
MUY_04439	MUY_04439
MUY_04439	MUY_04501
MUY_04439	MUY_04505
MUY_04439	MUY_00447
MUY_04439	MUY_00856
MUY_04439	MUY_00917
MUY_04439	MUY_00945
MUY_04443	MUY_04443
MUY_04445	MUY_04445
MUY_04445	MUY_04453
MUY_04445	MUY_00420
MUY_04445	MUY_00434
MUY_04445	MUY_00442
MUY_04445	MUY_00516
MUY_04445	MUY_00585
MUY_04445	MUY_00800
MUY_04445	MUY_00815
MUY_04445	MUY_00937
MUY_04445	MUY_00938
MUY_04445	MUY_00939
MUY_04445	MUY_00968
MUY_04445	MUY_00975
MUY_04445	MUY_00996
MUY_04445	MUY_01000
MUY_04447	MUY_04447
MUY_04447	MUY_04448
MUY_04447	MUY_04470
MUY_04447	MUY_04471
MUY_04447	MUY_00418
MUY_04447	MUY_00436

MUY_04447	MUY_00438
MUY_04447	MUY_00575
MUY_04447	MUY_00576
MUY_04448	MUY_04448
MUY_04448	MUY_04471
MUY_04448	MUY_00418
MUY_04448	MUY_00436
MUY_04448	MUY_00575
MUY_04448	MUY_00576
MUY_04448	MUY_00790
MUY_04448	MUY_00961
MUY_00400	MUY_00400
MUY_00400	MUY_00559
MUY_04453	MUY_04453
MUY_04453	MUY_00420
MUY_04453	MUY_00434
MUY_04453	MUY_00442
MUY_04453	MUY_00516
MUY_04453	MUY_00585
MUY_04453	MUY_00800
MUY_04453	MUY_00815
MUY_04453	MUY_00937
MUY_04453	MUY_00938
MUY_04453	MUY_00939
MUY_04453	MUY_00968
MUY_04453	MUY_00975
MUY_04453	MUY_00996
MUY_04453	MUY_01000
MUY_04460	MUY_04460
MUY_04463	MUY_04463
MUY_04463	MUY_00557
MUY_04463	MUY_00583
MUY_04463	MUY_00674
MUY_04465	MUY_04465
MUY_04466	MUY_04466
MUY_04466	MUY_00435
MUY_04466	MUY_00780
MUY_00401	MUY_00401
MUY_00401	MUY_00563
MUY_04467	MUY_04467
MUY_04467	MUY_00824
MUY_04470	MUY_04470
MUY_04470	MUY_00438
MUY_04470	MUY_00575
MUY_04470	MUY_00732
MUY_04471	MUY_04471
MUY_04471	MUY_00418

MUY_04471	MUY_00436
MUY_04471	MUY_00575
MUY_04471	MUY_00576
MUY_04471	MUY_00790
MUY_04471	MUY_00961
MUY_04473	MUY_04473
MUY_04473	MUY_00912
MUY_04474	MUY_04474
MUY_04476	MUY_04476
MUY_04476	MUY_00541
MUY_04476	MUY_00610
MUY_00402	MUY_00402
MUY_04482	MUY_04482
MUY_04482	MUY_00769
MUY_04482	MUY_00839
MUY_04483	MUY_04483
MUY_04483	MUY_00450
MUY_04483	MUY_00464
MUY_04483	MUY_00808
MUY_04483	MUY_00840
MUY_00403	MUY_00403
MUY_04484	MUY_04484
MUY_04485	MUY_04485
MUY_04485	MUY_00787
MUY_04485	MUY_00838
MUY_04485	MUY_00886
MUY_04486	MUY_04486
MUY_04492	MUY_04492
MUY_04492	MUY_00535
MUY_04492	MUY_00536
MUY_04492	MUY_00537
MUY_04492	MUY_00539
MUY_04492	MUY_00540
MUY_04494	MUY_04494
MUY_04494	MUY_04496
MUY_04495	MUY_04495
MUY_04496	MUY_04496
MUY_04497	MUY_04497
MUY_04497	MUY_04508
MUY_04497	MUY_00804
MUY_04497	MUY_00963
MUY_04501	MUY_04501
MUY_04501	MUY_04502
MUY_04501	MUY_04505
MUY_04501	MUY_00551
MUY_04501	MUY_00599
MUY_04501	MUY_00602

MUY_04501	MUY_00691
MUY_04501	MUY_00795
MUY_04501	MUY_00086
MUY_04501	MUY_00945
MUY_04501	MUY_00973
MUY_04502	MUY_04502
MUY_04502	MUY_00002
MUY_04503	MUY_04503
MUY_04504	MUY_04504
MUY_04504	MUY_00541
MUY_04504	MUY_00571
MUY_04504	MUY_00628
MUY_04505	MUY_04505
MUY_04505	MUY_00551
MUY_04505	MUY_00599
MUY_04505	MUY_00602
MUY_04505	MUY_00691
MUY_04505	MUY_00795
MUY_04505	MUY_00086
MUY_04505	MUY_00945
MUY_04505	MUY_00973
MUY_04506	MUY_04506
MUY_04507	MUY_04507
MUY_04507	MUY_00896
MUY_04508	MUY_04508
MUY_04508	MUY_00804
MUY_04510	MUY_04510
MUY_04511	MUY_04511
MUY_04511	MUY_00527
MUY_00415	MUY_00415
MUY_00033	MUY_00033
MUY_00416	MUY_00416
MUY_00418	MUY_00418
MUY_00418	MUY_00436
MUY_00418	MUY_00575
MUY_00418	MUY_00576
MUY_00418	MUY_00790
MUY_00418	MUY_00961
MUY_00419	MUY_00419
MUY_00420	MUY_00420
MUY_00420	MUY_00434
MUY_00420	MUY_00442
MUY_00420	MUY_00516
MUY_00420	MUY_00585
MUY_00420	MUY_00800
MUY_00420	MUY_00815
MUY_00420	MUY_00937

MUY_00420	MUY_00938
MUY_00420	MUY_00939
MUY_00420	MUY_00968
MUY_00420	MUY_00975
MUY_00420	MUY_00996
MUY_00420	MUY_01000
MUY_00034	MUY_00034
MUY_00423	MUY_00423
MUY_00424	MUY_00424
MUY_00036	MUY_00036
MUY_00433	MUY_00433
MUY_00433	MUY_00813
MUY_00434	MUY_00434
MUY_00434	MUY_00442
MUY_00434	MUY_00516
MUY_00434	MUY_00585
MUY_00434	MUY_00800
MUY_00434	MUY_00815
MUY_00434	MUY_00937
MUY_00434	MUY_00938
MUY_00434	MUY_00939
MUY_00434	MUY_00968
MUY_00434	MUY_00975
MUY_00434	MUY_00996
MUY_00434	MUY_01000
MUY_00435	MUY_00435
MUY_00435	MUY_00514
MUY_00435	MUY_00590
MUY_00435	MUY_00698
MUY_00435	MUY_00780
MUY_00436	MUY_00436
MUY_00436	MUY_00575
MUY_00436	MUY_00576
MUY_00436	MUY_00790
MUY_00436	MUY_00961
MUY_00438	MUY_00438
MUY_00438	MUY_00575
MUY_00439	MUY_00439
MUY_00442	MUY_00442
MUY_00442	MUY_00516
MUY_00442	MUY_00585
MUY_00442	MUY_00800
MUY_00442	MUY_00815
MUY_00442	MUY_00937
MUY_00442	MUY_00938
MUY_00442	MUY_00939
MUY_00442	MUY_00968

MUY_00442	MUY_00975
MUY_00442	MUY_00996
MUY_00442	MUY_01000
MUY_00038	MUY_00038
MUY_00038	MUY_00002
MUY_00443	MUY_00443
MUY_00445	MUY_00445
MUY_00445	MUY_00677
MUY_00445	MUY_00749
MUY_00445	MUY_00779
MUY_00445	MUY_00882
MUY_00446	MUY_00446
MUY_00447	MUY_00447
MUY_00447	MUY_00856
MUY_00447	MUY_00917
MUY_00447	MUY_00945
MUY_00449	MUY_00449
MUY_00449	MUY_00828
MUY_00450	MUY_00450
MUY_00450	MUY_00464
MUY_00450	MUY_00808
MUY_00450	MUY_00840
MUY_00451	MUY_00451
MUY_00451	MUY_00807
MUY_00451	MUY_00809
MUY_00451	MUY_00944
MUY_00453	MUY_00453
MUY_00453	MUY_00797
MUY_00453	MUY_00872
MUY_00457	MUY_00457
MUY_00041	MUY_00041
MUY_00041	MUY_00463
MUY_00041	MUY_00064
MUY_00459	MUY_00459
MUY_00459	MUY_00461
MUY_00460	MUY_00460
MUY_00461	MUY_00461
MUY_00461	MUY_00523
MUY_00461	MUY_00895
MUY_00042	MUY_00042
MUY_00042	MUY_00534
MUY_00462	MUY_00462
MUY_00463	MUY_00463
MUY_00463	MUY_00064
MUY_00464	MUY_00464
MUY_00464	MUY_00808
MUY_00464	MUY_00840

MUY_00466	MUY_00466
MUY_00468	MUY_00468
MUY_00469	MUY_00469
MUY_00469	MUY_00485
MUY_00469	MUY_00696
MUY_00470	MUY_00483
MUY_00043	MUY_00043
MUY_00472	MUY_00472
MUY_00472	MUY_00473
MUY_00472	MUY_00894
MUY_00473	MUY_00473
MUY_00473	MUY_00893
MUY_00473	MUY_00894
MUY_00474	MUY_00474
MUY_00475	MUY_00475
MUY_00478	MUY_00478
MUY_00478	MUY_00479
MUY_00479	MUY_00479
MUY_00002	MUY_00002
MUY_00002	MUY_00603
MUY_00480	MUY_00480
MUY_00481	MUY_00481
MUY_00481	MUY_00573
MUY_00482	MUY_00482
MUY_00482	MUY_00483
MUY_00483	MUY_00483
MUY_00484	MUY_00484
MUY_00484	MUY_00940
MUY_00485	MUY_00485
MUY_00485	MUY_00696
MUY_00486	MUY_00486
MUY_00486	MUY_00681
MUY_00486	MUY_00721
MUY_00486	MUY_00933
MUY_00487	MUY_00487
MUY_00489	MUY_00489
MUY_00492	MUY_00492
MUY_00044	MUY_00044
MUY_00044	MUY_00765
MUY_00495	MUY_00495
MUY_00496	MUY_00496
MUY_00496	MUY_00046
MUY_00496	MUY_00527
MUY_00496	MUY_00008
MUY_00496	MUY_00731
MUY_00496	MUY_00816
MUY_00496	MUY_00979

MUY_00501	MUY_00501
MUY_00502	MUY_00502
MUY_00504	MUY_00504
MUY_00504	MUY_00782
MUY_00504	MUY_00920
MUY_00507	MUY_00507
MUY_00507	MUY_00738
MUY_00512	MUY_00512
MUY_00514	MUY_00589
MUY_00514	MUY_00590
MUY_00514	MUY_00780
MUY_00516	MUY_00516
MUY_00516	MUY_00585
MUY_00516	MUY_00800
MUY_00516	MUY_00815
MUY_00516	MUY_00937
MUY_00516	MUY_00938
MUY_00516	MUY_00939
MUY_00516	MUY_00968
MUY_00516	MUY_00975
MUY_00516	MUY_00996
MUY_00516	MUY_01000
MUY_00046	MUY_00046
MUY_00046	MUY_00527
MUY_00046	MUY_00008
MUY_00046	MUY_00816
MUY_00519	MUY_00519
MUY_00523	MUY_00523
MUY_00523	MUY_00861
MUY_00524	MUY_00524
MUY_00525	MUY_00525
MUY_00526	MUY_00526
MUY_00526	MUY_00857
MUY_00047	MUY_00047
MUY_00527	MUY_00527
MUY_00527	MUY_00816
MUY_00532	MUY_00532
MUY_00532	MUY_00768
MUY_00533	MUY_00533
MUY_00534	MUY_00534
MUY_00535	MUY_00535
MUY_00535	MUY_00536
MUY_00535	MUY_00537
MUY_00535	MUY_00539
MUY_00535	MUY_00540
MUY_00536	MUY_00536
MUY_00536	MUY_00537

MUY_00536	MUY_00539
MUY_00536	MUY_00540
MUY_00537	MUY_00537
MUY_00537	MUY_00539
MUY_00537	MUY_00540
MUY_00537	MUY_00542
MUY_00537	MUY_00070
MUY_00538	MUY_00538
MUY_00538	MUY_00539
MUY_00539	MUY_00539
MUY_00539	MUY_00540
MUY_00540	MUY_00540
MUY_00540	MUY_00541
MUY_00540	MUY_00542
MUY_00540	MUY_00070
MUY_00541	MUY_00541
MUY_00541	MUY_00571
MUY_00541	MUY_00628
MUY_00542	MUY_00542
MUY_00542	MUY_00070
MUY_00049	MUY_00049
MUY_00551	MUY_00551
MUY_00551	MUY_00599
MUY_00551	MUY_00602
MUY_00551	MUY_00628
MUY_00551	MUY_00691
MUY_00551	MUY_00795
MUY_00551	MUY_00086
MUY_00551	MUY_00973
MUY_00553	MUY_00979
MUY_00554	MUY_00554
MUY_00050	MUY_00050
MUY_00556	MUY_00556
MUY_00556	MUY_00710
MUY_00557	MUY_00557
MUY_00557	MUY_00583
MUY_00557	MUY_00674
MUY_00558	MUY_00558
MUY_00559	MUY_00559
MUY_00560	MUY_00560
MUY_00560	MUY_00855
MUY_00560	MUY_00904
MUY_00052	MUY_00052
MUY_00561	MUY_00561
MUY_00561	MUY_00734
MUY_00563	MUY_00563
MUY_00567	MUY_00567

MUY_00571	MUY_00571
MUY_00571	MUY_00572
MUY_00571	MUY_00628
MUY_00053	MUY_00053
MUY_00053	MUY_00074
MUY_00572	MUY_00572
MUY_00572	MUY_00697
MUY_00573	MUY_00573
MUY_00575	MUY_00575
MUY_00575	MUY_00576
MUY_00576	MUY_00576
MUY_00576	MUY_00790
MUY_00576	MUY_00961
MUY_00054	MUY_00054
MUY_00577	MUY_00577
MUY_00055	MUY_00055
MUY_00578	MUY_00578
MUY_00056	MUY_00056
MUY_00580	MUY_00580
MUY_00580	MUY_00740
MUY_00580	MUY_00741
MUY_00580	MUY_00742
MUY_00580	MUY_00744
MUY_00581	MUY_00581
MUY_00582	MUY_00582
MUY_00582	MUY_00584
MUY_00582	MUY_00072
MUY_00583	MUY_00583
MUY_00583	MUY_00674
MUY_00584	MUY_00584
MUY_00584	MUY_00072
MUY_00585	MUY_00585
MUY_00585	MUY_00800
MUY_00585	MUY_00815
MUY_00585	MUY_00937
MUY_00585	MUY_00938
MUY_00585	MUY_00939
MUY_00585	MUY_00968
MUY_00585	MUY_00975
MUY_00585	MUY_00996
MUY_00585	MUY_01000
MUY_00587	MUY_00587
MUY_00588	MUY_00588
MUY_00588	MUY_00773
MUY_00589	MUY_00589
MUY_00589	MUY_00590
MUY_00589	MUY_00741

MUY_00589	MUY_00780
MUY_00590	MUY_00741
MUY_00590	MUY_00780
MUY_00593	MUY_00593
MUY_00057	MUY_00057
MUY_00057	MUY_00074
MUY_00598	MUY_00598
MUY_00599	MUY_00599
MUY_00599	MUY_00602
MUY_00599	MUY_00628
MUY_00599	MUY_00691
MUY_00599	MUY_00795
MUY_00599	MUY_00086
MUY_00599	MUY_00973
MUY_00602	MUY_00602
MUY_00602	MUY_00628
MUY_00602	MUY_00691
MUY_00602	MUY_00795
MUY_00602	MUY_00086
MUY_00602	MUY_00973
MUY_00606	MUY_00606
MUY_00609	MUY_00609
MUY_00058	MUY_00058
MUY_00610	MUY_00610
MUY_00618	MUY_00618
MUY_00628	MUY_00628
MUY_00628	MUY_00691
MUY_00628	MUY_00795
MUY_00628	MUY_00086
MUY_00628	MUY_00973
MUY_00630	MUY_00630
MUY_00059	MUY_00059
MUY_00631	MUY_00631
MUY_00640	MUY_00640
MUY_00643	MUY_00643
MUY_00648	MUY_00648
MUY_00648	MUY_00072
MUY_00656	MUY_00656
MUY_00662	MUY_00662
MUY_00674	MUY_00674
MUY_00675	MUY_00675
MUY_00677	MUY_00677
MUY_00677	MUY_00749
MUY_00677	MUY_00779
MUY_00677	MUY_00882
MUY_00678	MUY_00678
MUY_00681	MUY_00681

MUY_00681	MUY_00721
MUY_00681	MUY_00933
MUY_00682	MUY_00682
MUY_00684	MUY_00684
MUY_00688	MUY_00688
MUY_00688	MUY_00971
MUY_00689	MUY_00689
MUY_00062	MUY_00062
MUY_00691	MUY_00691
MUY_00691	MUY_00795
MUY_00691	MUY_00086
MUY_00691	MUY_00973
MUY_00695	MUY_00695
MUY_00695	MUY_00906
MUY_00696	MUY_00696
MUY_00698	MUY_00698
MUY_00698	MUY_00871
MUY_00699	MUY_00699
MUY_00700	MUY_00700
MUY_00701	MUY_00701
MUY_00702	MUY_00702
MUY_00706	MUY_00706
MUY_00708	MUY_00708
MUY_00709	MUY_00709
MUY_00008	MUY_00008
MUY_00064	MUY_00064
MUY_00710	MUY_00710
MUY_00713	MUY_00713
MUY_00719	MUY_00719
MUY_00721	MUY_00721
MUY_00722	MUY_00722
MUY_00065	MUY_00065
MUY_00729	MUY_00729
MUY_00066	MUY_00066
MUY_00730	MUY_00730
MUY_00730	MUY_00080
MUY_00730	MUY_00081
MUY_00734	MUY_00734
MUY_00736	MUY_00736
MUY_00736	MUY_00739
MUY_00738	MUY_00738
MUY_00068	MUY_00068
MUY_00068	MUY_00780
MUY_00739	MUY_00739
MUY_00740	MUY_00740
MUY_00740	MUY_00741
MUY_00740	MUY_00742

MUY_00740	MUY_00744
MUY_00741	MUY_00741
MUY_00741	MUY_00742
MUY_00741	MUY_00744
MUY_00742	MUY_00742
MUY_00742	MUY_00744
MUY_00743	MUY_00743
MUY_00743	MUY_00074
MUY_00744	MUY_00744
MUY_00745	MUY_00745
MUY_00746	MUY_00746
MUY_00747	MUY_00747
MUY_00749	MUY_00749
MUY_00749	MUY_00779
MUY_00749	MUY_00882
MUY_00750	MUY_00750
MUY_00750	MUY_00811
MUY_00070	MUY_00070
MUY_00754	MUY_00754
MUY_00755	MUY_00758
MUY_00756	MUY_00756
MUY_00757	MUY_00757
MUY_00758	MUY_00758
MUY_00759	MUY_00759
MUY_00765	MUY_00765
MUY_00766	MUY_00766
MUY_00767	MUY_00767
MUY_00768	MUY_00768
MUY_00769	MUY_00769
MUY_00773	MUY_00773
MUY_00774	MUY_00774
MUY_00774	MUY_00775
MUY_00775	MUY_00775
MUY_00071	MUY_00071
MUY_00071	MUY_00780
MUY_00778	MUY_00778
MUY_00779	MUY_00779
MUY_00779	MUY_00882
MUY_00780	MUY_00780
MUY_00780	MUY_00075
MUY_00780	MUY_00895
MUY_00782	MUY_00782
MUY_00782	MUY_00920
MUY_00783	MUY_00783
MUY_00072	MUY_00072
MUY_00784	MUY_00784
MUY_00785	MUY_00785

MUY_00787	MUY_00787
MUY_00787	MUY_00838
MUY_00787	MUY_00886
MUY_00789	MUY_00789
MUY_00790	MUY_00790
MUY_00790	MUY_00961
MUY_00792	MUY_00792
MUY_00073	MUY_00073
MUY_00795	MUY_00795
MUY_00795	MUY_00086
MUY_00795	MUY_00973
MUY_00797	MUY_00797
MUY_00800	MUY_00800
MUY_00800	MUY_00815
MUY_00800	MUY_00937
MUY_00800	MUY_00938
MUY_00800	MUY_00939
MUY_00800	MUY_00968
MUY_00800	MUY_00975
MUY_00800	MUY_00996
MUY_00800	MUY_01000
MUY_00802	MUY_00802
MUY_00803	MUY_00803
MUY_00804	MUY_00804
MUY_00804	MUY_00963
MUY_00074	MUY_00074
MUY_00807	MUY_00807
MUY_00807	MUY_00809
MUY_00807	MUY_00944
MUY_00808	MUY_00808
MUY_00808	MUY_00840
MUY_00809	MUY_00809
MUY_00809	MUY_00944
MUY_00810	MUY_00810
MUY_00810	MUY_00869
MUY_00811	MUY_00811
MUY_00812	MUY_00812
MUY_00813	MUY_00813
MUY_00815	MUY_00815
MUY_00815	MUY_00937
MUY_00815	MUY_00938
MUY_00815	MUY_00939
MUY_00815	MUY_00968
MUY_00815	MUY_00975
MUY_00815	MUY_00996
MUY_00815	MUY_01000
MUY_00816	MUY_00816

MUY_00818	MUY_00818
MUY_00824	MUY_00824
MUY_00825	MUY_00825
MUY_00826	MUY_00826
MUY_00828	MUY_00828
MUY_00829	MUY_00829
MUY_00830	MUY_00830
MUY_00833	MUY_00833
MUY_00076	MUY_00076
MUY_00838	MUY_00838
MUY_00838	MUY_00886
MUY_00840	MUY_00840
MUY_00077	MUY_00077
MUY_00845	MUY_00845
MUY_00845	MUY_00851
MUY_00847	MUY_00847
MUY_00847	MUY_00921
MUY_00849	MUY_00849
MUY_00851	MUY_00851
MUY_00852	MUY_00852
MUY_00853	MUY_00853
MUY_00854	MUY_00854
MUY_00855	MUY_00855
MUY_00855	MUY_00904
MUY_00856	MUY_00856
MUY_00856	MUY_00917
MUY_00856	MUY_00945
MUY_00857	MUY_00857
MUY_00861	MUY_00861
MUY_00866	MUY_00866
MUY_00080	MUY_00080
MUY_00080	MUY_00081
MUY_00869	MUY_00869
MUY_00871	MUY_00871
MUY_00872	MUY_00872
MUY_00875	MUY_00875
MUY_00875	MUY_00931
MUY_00882	MUY_00882
MUY_00081	MUY_00081
MUY_00886	MUY_00886
MUY_00888	MUY_00888
MUY_00082	MUY_00082
MUY_00890	MUY_00890
MUY_00892	MUY_00892
MUY_00893	MUY_00893
MUY_00893	MUY_00894
MUY_00894	MUY_00894

MUY_00895	MUY_00895
MUY_00896	MUY_00896
MUY_00083	MUY_00083
MUY_00083	MUY_00085
MUY_00899	MUY_00899
MUY_00900	MUY_00900
MUY_00902	MUY_00902
MUY_00904	MUY_00904
MUY_00084	MUY_00084
MUY_00084	MUY_00085
MUY_00906	MUY_00906
MUY_00907	MUY_00907
MUY_00907	MUY_00908
MUY_00908	MUY_00908
MUY_00085	MUY_00085
MUY_00909	MUY_00909
MUY_00911	MUY_00911
MUY_00912	MUY_00912
MUY_00086	MUY_00086
MUY_00086	MUY_00973
MUY_00915	MUY_00915
MUY_00087	MUY_00087
MUY_00916	MUY_00916
MUY_00917	MUY_00917
MUY_00917	MUY_00945
MUY_00920	MUY_00920
MUY_00921	MUY_00921
MUY_00927	MUY_00927
MUY_00931	MUY_00931
MUY_00933	MUY_00933
MUY_00937	MUY_00937
MUY_00937	MUY_00938
MUY_00937	MUY_00939
MUY_00937	MUY_00968
MUY_00937	MUY_00975
MUY_00937	MUY_00996
MUY_00937	MUY_01000
MUY_00938	MUY_00938
MUY_00938	MUY_00939
MUY_00938	MUY_00968
MUY_00938	MUY_00975
MUY_00938	MUY_00996
MUY_00938	MUY_01000
MUY_00939	MUY_00939
MUY_00939	MUY_00968
MUY_00939	MUY_00975
MUY_00939	MUY_00996

MUY_00939	MUY_01000
MUY_00940	MUY_00965
MUY_00944	MUY_00944
MUY_00945	MUY_00945
MUY_00946	MUY_00946
MUY_00961	MUY_00961
MUY_00963	MUY_00963
MUY_00965	MUY_00965
MUY_00967	MUY_00967
MUY_00968	MUY_00968
MUY_00968	MUY_00975
MUY_00968	MUY_00996
MUY_00968	MUY_01000
MUY_00969	MUY_00969
MUY_00971	MUY_00971
MUY_00973	MUY_00973
MUY_00975	MUY_00975
MUY_00975	MUY_00996
MUY_00975	MUY_01000
MUY_00979	MUY_00979
MUY_00979	MUY_00998
MUY_00983	MUY_00983
MUY_00991	MUY_00991
MUY_00993	MUY_00993
MUY_00996	MUY_00996
MUY_00996	MUY_01000
MUY_00998	MUY_00998
MUY_01000	MUY_01000

Supplementary Table S2. The functional categories of proteins in MDS.

COG functional catogories	Number of proteins in PPI network (exclude homomeric interactions)	Number of proteins in MDS	p-value
C Energy production and conversion	134	22	0.794645
D Cell cycle control, cell divesion, chromosome partitioning	24	6	0.286462
E Amino acid transport and metabolism	202	38	0.522338
F Nucleotide transport and metabolism	56	9	0.750342
G Carbohydrate transport and metabolism	161	40	0.028165
H Coenzyme transport and metabolism	91	18	0.442295
I Lipid transport and metabolism	83	11	0.931958
J Translation, ribosomal structure and biogenesis	126	26	0.323716
K Transcription	267	30	0.999856
L Replication, recombination and repair	100	27	0.024499
M Cell wall/membrane/envelope biogenesis	120	22	0.587086
N Cell motility	36	8	0.359046
O Posttranslational modification, protein turnover, chaperones	75	12	0.776378
P Inorganic ion transport and metabolism	89	14	0.81033
Q Secondary metabolites biosynthesis, transport and catabolism	31	7	0.359406
R General function prediction only	201	46	0.071573
S Function unknown	196	52	0.003131
T Signal transduction mechanisms	109	11	0.99621
U Intracellular trafficking, secretion, and vesicular transport	15	3	0.554359
V Defense mechanisms	49	4	0.98919

Supplementary Table S3. The predicted protein complexes.

Protein Complex 1	MUY_01012	MUY_03388	MUY_00578
Protein Complex 2	MUY_01015	MUY_01060	MUY_01062	MUY_01492	MUY_00010	MUY_01719	MUY_01857	MUY_02376	MUY_02468	MUY_02991	MUY_03222	MUY_03263	MUY_03292	MUY_03434	MUY_04020	MUY_04022
Protein Complex 3	MUY_01016	MUY_00200	MUY_02834	MUY_03186	MUY_03876	MUY_04337	MUY_00708
Protein Complex 4	MUY_01017	MUY_01049	MUY_01075	MUY_01244	MUY_01609	MUY_01693	MUY_01735	MUY_02030	MUY_02361	MUY_02588	MUY_00297	MUY_03518	MUY_03667	MUY_04463	MUY_00557	MUY_00583	MUY_00674
Protein Complex 5	MUY_01023	MUY_01419	MUY_01422	MUY_01936	MUY_02132	MUY_03965	MUY_00554
Protein Complex 6	MUY_01026	MUY_02095	MUY_03170	MUY_03691	MUY_04192	MUY_04434
Protein Complex 7	MUY_01027	MUY_01163	MUY_01682	MUY_01683	MUY_02127	MUY_02160	MUY_02171	MUY_02201	MUY_02269	MUY_02619	MUY_02648	MUY_02841	MUY_02914	MUY_02941	MUY_03136	MUY_03232	MUY_03279	MUY_03513	MUY_03515	MUY_03524	MUY_03585	MUY_03840	MUY_04073	MUY_00459	MUY_00461	MUY_00523	MUY_00861	MUY_00895
Protein Complex 8	MUY_01030	MUY_01152	MUY_01382	MUY_02707	MUY_02708	MUY_02882	MUY_02883	MUY_03769	MUY_03778	MUY_03830	MUY_04185	MUY_04271	MUY_04291	MUY_04501	MUY_04505	MUY_00551	MUY_00599	MUY_00602	MUY_00691	MUY_00795	MUY_00086	MUY_00973
Protein Complex 9	MUY_01033	MUY_02054	MUY_04188	MUY_00993
Protein Complex 10	MUY_01036	MUY_02129	MUY_03015	MUY_03490	MUY_04046
Protein Complex 11	MUY_01040	MUY_01372	MUY_01406	MUY_01623	MUY_01969	MUY_02062	MUY_02258	MUY_02321	MUY_02508	MUY_02515	MUY_00021	MUY_03050
Protein Complex 12	MUY_01041	MUY_01365	MUY_01368	MUY_01634	MUY_00207	MUY_02203	MUY_02978	MUY_00290	MUY_03320	MUY_03640	MUY_03984	MUY_04198	MUY_00401	MUY_00563
Protein Complex 13	MUY_01042	MUY_03206	MUY_04243
Protein Complex 14	MUY_01044	MUY_01090	MUY_01164	MUY_01245	MUY_01280	MUY_01329	MUY_01421	MUY_01611	MUY_01625	MUY_01923	MUY_01973	MUY_02100	MUY_02153	MUY_02211	MUY_02331	MUY_02615	MUY_02633	MUY_02950	MUY_02958	MUY_00292	MUY_03128	MUY_03225	MUY_00313	MUY_03494
	MUY_03606	MUY_03627	MUY_03638	MUY_03964	MUY_03998	MUY_04038	MUY_04045	MUY_00486	MUY_00681	MUY_00721	MUY_00933
Protein Complex 15	MUY_01046	MUY_01239	MUY_00392
Protein Complex 16	MUY_01047	MUY_01114	MUY_01141	MUY_01415	MUY_01437	MUY_01660	MUY_02198	MUY_02240	MUY_00255	MUY_00256	MUY_02780	MUY_02797	MUY_03314	MUY_03683	MUY_03967	MUY_03980	MUY_04043	MUY_04086	MUY_00385	MUY_04334	MUY_04343	MUY_00435	MUY_00483	MUY_00514	MUY_00589	MUY_00590	MUY_00722	MUY_00780
Protein Complex 17	MUY_01048	MUY_01237	MUY_01418	MUY_01520	MUY_01616	MUY_01919	MUY_01937	MUY_01989	MUY_00190	MUY_02094	MUY_02152	MUY_02439	MUY_02467	MUY_02627	MUY_00261	MUY_00308	MUY_03333	MUY_03386	MUY_03449	MUY_03454	MUY_03637	MUY_03810	MUY_04070	MUY_04242	MUY_00382	MUY_04302	MUY_04383	MUY_04433	MUY_04483	MUY_00450	MUY_00464	MUY_00808	MUY_00840
Protein Complex 18	MUY_01052	MUY_00105	MUY_00114	MUY_00115	MUY_01390	MUY_01504	MUY_01696	MUY_01760	MUY_01761	MUY_01879	MUY_00181	MUY_00182	MUY_02516	MUY_02560	MUY_02784	MUY_02845	MUY_02852	MUY_02889	MUY_02892	MUY_02900	MUY_03708	MUY_03918	MUY_04026	MUY_04258	MUY_04322	MUY_04504	MUY_00541	MUY_00571	MUY_00628
Protein Complex 19	MUY_01053	MUY_01176	MUY_01230	MUY_01523	MUY_03721	MUY_00512
Protein Complex 20	MUY_01059	MUY_01140	MUY_01374	MUY_01970	MUY_01999	MUY_02036	MUY_02049	MUY_02231	MUY_02238	MUY_02297	MUY_02580	MUY_02982	MUY_03140	MUY_03250	MUY_03431	MUY_00323	MUY_03553	MUY_03612	MUY_00445	MUY_00677	MUY_00749	MUY_00779	MUY_00882
Protein Complex 21	MUY_01061	MUY_01494	MUY_02015	MUY_02134	MUY_00241	MUY_02947	MUY_00277	MUY_00337	MUY_00433	MUY_00813
Protein Complex 22	MUY_01066	MUY_00160	MUY_01993	MUY_02659
Protein Complex 23	MUY_01038	MUY_00089	MUY_01313	MUY_00022	MUY_03162	MUY_03485	MUY_04035	MUY_00935
Protein Complex 24	MUY_01069	MUY_02658	MUY_04307	MUY_04400
Protein Complex 25	MUY_01076	MUY_00165	MUY_01907	MUY_02518	MUY_04301	MUY_00698	MUY_00871
Protein Complex 26	MUY_01078	MUY_03103	MUY_00082
Protein Complex 27	MUY_01081	MUY_01344	MUY_03317	MUY_03934	MUY_04183	MUY_04335	MUY_04430	MUY_00847	MUY_00921
Protein Complex 28	MUY_01093	MUY_01506	MUY_03242	MUY_00050
Protein Complex 29	MUY_00092	MUY_01531	MUY_01846	MUY_01847	MUY_02012	MUY_03091	MUY_03093	MUY_00024	MUY_04196	MUY_00036
Protein Complex 30	MUY_01103	MUY_01260	MUY_01676	MUY_03969	MUY_03995	MUY_04080	MUY_04404	MUY_00474
Protein Complex 31	MUY_01106	MUY_01322	MUY_01671	MUY_01955	MUY_00218	MUY_03393	MUY_03438
Protein Complex 32	MUY_01740	MUY_01741	MUY_01750	MUY_00068
Protein Complex 33	MUY_01115	MUY_01624	MUY_02506	MUY_03121	MUY_03418
Protein Complex 34	MUY_01117	MUY_02233	MUY_02448	MUY_02829	MUY_03251	MUY_03847	MUY_04122
Protein Complex 35	MUY_01124	MUY_01482	MUY_01497	MUY_01528	MUY_01701	MUY_02050	MUY_02216	MUY_02232	MUY_02364	MUY_02695	MUY_00272	MUY_03126	MUY_03240	MUY_03659	MUY_03701	MUY_03880	MUY_04014	MUY_04194	MUY_00556	MUY_00710
Protein Complex 36	MUY_01128	MUY_01987	MUY_02916	MUY_02918	MUY_03014	MUY_03054	MUY_03244	MUY_03556	MUY_03573	MUY_03574	MUY_03660	MUY_00766
Protein Complex 37	MUY_01129	MUY_02128	MUY_02827
Protein Complex 38	MUY_01135	MUY_01943	MUY_02589
Protein Complex 39	MUY_01136	MUY_01510	MUY_01742	MUY_02434	MUY_00251	MUY_02996	MUY_03459	MUY_04116
Protein Complex 40	MUY_01139	MUY_01702	MUY_01952	MUY_01953	MUY_03567	MUY_04127
Protein Complex 41	MUY_01148	MUY_01673	MUY_01986	MUY_02136	MUY_02622	MUY_03257
Protein Complex 42	MUY_01149	MUY_01246	MUY_01353	MUY_02149	MUY_02242	MUY_02447	MUY_02696	MUY_02788	MUY_03055	MUY_04126	MUY_04239	MUY_00469	MUY_00485	MUY_00696
Protein Complex 43	MUY_01150	MUY_01198	MUY_02176	MUY_02182	MUY_02410	MUY_00273	MUY_03141	MUY_03241	MUY_03261	MUY_03373	MUY_00376	MUY_04033	MUY_04034	MUY_04036	MUY_00377	MUY_00378	MUY_04228	MUY_04230	MUY_00379
Protein Complex 44	MUY_01156	MUY_01315	MUY_01490	MUY_01756	MUY_02229	MUY_00275	MUY_04159
Protein Complex 45	MUY_01158	MUY_02730	MUY_02977	MUY_03601	MUY_03792	MUY_04042	MUY_04408	MUY_00443
Protein Complex 46	MUY_01161	MUY_01486	MUY_01727	MUY_02295	MUY_02578	MUY_02830	MUY_03356	MUY_03860	MUY_03951	MUY_04473	MUY_00912
Protein Complex 47	MUY_01169	MUY_01661	MUY_02711	MUY_00719
Protein Complex 48	MUY_01175	MUY_01639	MUY_02355	MUY_02618	MUY_03173	MUY_03557
Protein Complex 49	MUY_01189	MUY_01232	MUY_01602	MUY_02159	MUY_03267	MUY_03644	MUY_03962	MUY_03975	MUY_04082	MUY_04328	MUY_04428	MUY_00700
Protein Complex 50	MUY_01193	MUY_00307	MUY_04235	MUY_00899
Protein Complex 51	MUY_01195	MUY_01197	MUY_02045	MUY_04018
Protein Complex 52	MUY_01205	MUY_03580	MUY_00371	MUY_00702
Protein Complex 53	MUY_01207	MUY_01208	MUY_01341	MUY_00758
Protein Complex 54	MUY_01209	MUY_01321	MUY_01959	MUY_02025	MUY_02289	MUY_02620	MUY_03341	MUY_03375	MUY_03451	MUY_03775	MUY_00526	MUY_00857
Protein Complex 55	MUY_01225	MUY_02814	MUY_02821
Protein Complex 56	MUY_01226	MUY_01776	MUY_01786	MUY_01885	MUY_02202	MUY_02413	MUY_03025	MUY_03345	MUY_04174	MUY_00053	MUY_00588	MUY_00057	MUY_00743	MUY_00773	MUY_00074
Protein Complex 57	MUY_01228	MUY_01265	MUY_01477	MUY_01479	MUY_01804	MUY_01913	MUY_02187	MUY_03056	MUY_03134	MUY_04317	MUY_04385	MUY_00439	MUY_00745
Protein Complex 58	MUY_01231	MUY_01370	MUY_01524	MUY_02039	MUY_02115	MUY_02118	MUY_02124	MUY_02307	MUY_02411	MUY_02574	MUY_00250	MUY_03442	MUY_04171	MUY_04318	MUY_04410	MUY_00504	MUY_00782	MUY_00920
Protein Complex 59	MUY_01233	MUY_03310	MUY_03422	MUY_03703	MUY_04079	MUY_04391	MUY_00852
Protein Complex 60	MUY_01238	MUY_01266	MUY_02358	MUY_03082	MUY_00398	MUY_00451	MUY_00807	MUY_00809	MUY_00944
Protein Complex 61	MUY_01240	MUY_01243	MUY_03597
Protein Complex 62	MUY_01247	MUY_01248	MUY_00238	MUY_04245	MUY_00083
Protein Complex 63	MUY_01254	MUY_02373	MUY_03923
Protein Complex 64	MUY_01255	MUY_02454	MUY_02762	MUY_03902	MUY_04312	MUY_00561	MUY_00734
Protein Complex 65	MUY_01261	MUY_02334	MUY_03318	MUY_03491	MUY_00854
Protein Complex 66	MUY_01263	MUY_01912	MUY_02179	MUY_03099
Protein Complex 67	MUY_01268	MUY_01710	MUY_01782	MUY_02181	MUY_02316	MUY_02675
Protein Complex 68	MUY_01269	MUY_01779	MUY_04315
Protein Complex 69	MUY_01291	MUY_03034	MUY_03260	MUY_04136	MUY_00709
Protein Complex 70	MUY_01299	MUY_01348	MUY_02537	MUY_02576
Protein Complex 71	MUY_01300	MUY_02723	MUY_03589
Protein Complex 72	MUY_01319	MUY_02064	MUY_03237	MUY_00040
Protein Complex 73	MUY_01320	MUY_01775	MUY_02528	MUY_02668	MUY_03253	MUY_03299	MUY_00065
Protein Complex 74	MUY_01325	MUY_01326	MUY_01327	MUY_01646	MUY_01650	MUY_00012	MUY_03017
Protein Complex 75	MUY_01328	MUY_01659	MUY_03681	MUY_03961	MUY_03976	MUY_04153	MUY_04218	MUY_04395
Protein Complex 76	MUY_01345	MUY_01346	MUY_01363	MUY_01638	MUY_00243	MUY_03342	MUY_03382	MUY_03383	MUY_03945	MUY_00056
Protein Complex 77	MUY_01350	MUY_04039	MUY_00384
Protein Complex 78	MUY_01352	MUY_01474	MUY_00193	MUY_02234
Protein Complex 79	MUY_01354	MUY_01355	MUY_02990
Protein Complex 80	MUY_01357	MUY_02207	MUY_03786	MUY_04432	MUY_00423
Protein Complex 81	MUY_01359	MUY_00167	MUY_00174	MUY_01964	MUY_00301
Protein Complex 82	MUY_01366	MUY_01635	MUY_01637	MUY_01915	MUY_02210	MUY_02616	MUY_02697	MUY_02725	MUY_02910	MUY_02917	MUY_02981	MUY_03284	MUY_03304	MUY_00316	MUY_03699	MUY_03706	MUY_04467	MUY_00750	MUY_00811	MUY_00824
Protein Complex 83	MUY_02052	MUY_02606	MUY_04085	MUY_04206	MUY_00695	MUY_00906
Protein Complex 84	MUY_00119	MUY_00120	MUY_01699	MUY_01788	MUY_01882	MUY_02193	MUY_02816
Protein Complex 85	MUY_01379	MUY_02550	MUY_02614
Protein Complex 86	MUY_01381	MUY_01550	MUY_03771	MUY_00598
Protein Complex 87	MUY_01392	MUY_03109	MUY_00631
Protein Complex 88	MUY_01754	MUY_01755	MUY_01768	MUY_03146	MUY_03248
Protein Complex 89	MUY_01417	MUY_01572	MUY_02500	MUY_03715	MUY_00656
Protein Complex 90	MUY_00121	MUY_01805	MUY_02673
Protein Complex 91	MUY_01605	MUY_02135	MUY_03856
Protein Complex 92	MUY_01443	MUY_02371	MUY_04249	MUY_00481	MUY_00573
Protein Complex 93	MUY_01445	MUY_02582	MUY_02970	MUY_03658	MUY_04399
Protein Complex 94	MUY_01447	MUY_04424	MUY_00424
Protein Complex 95	MUY_01011	MUY_00126	MUY_01832	MUY_01909	MUY_03010
Protein Complex 96	MUY_00127	MUY_00132	MUY_00136	MUY_00139	MUY_00156	MUY_00157	MUY_01980	MUY_02805	MUY_02820	MUY_03062	MUY_03135	MUY_03774	MUY_04091	MUY_04205	MUY_04244	MUY_04494	MUY_00794	MUY_00979	MUY_00998
Protein Complex 97	MUY_01456	MUY_01730	MUY_01888	MUY_02665	MUY_00312	MUY_03608	MUY_03896	MUY_04466
Protein Complex 98	MUY_01457	MUY_02188	MUY_02617	MUY_02826
Protein Complex 99	MUY_01464	MUY_02196	MUY_03372	MUY_04216
Protein Complex 100	MUY_01465	MUY_00320	MUY_03970
Protein Complex 101	MUY_01468	MUY_02081	MUY_03129	MUY_03476
Protein Complex 102	MUY_01469	MUY_02190	MUY_00242	MUY_03031	MUY_03440
Protein Complex 103	MUY_01471	MUY_00279	MUY_03223	MUY_00326	MUY_04247
Protein Complex 104	MUY_01481	MUY_01975	MUY_02000	MUY_00866
Protein Complex 105	MUY_03367	MUY_04090	MUY_04486
Protein Complex 106	MUY_01485	MUY_02538	MUY_02541	MUY_02794	MUY_00023
Protein Complex 107	MUY_01514	MUY_02913	MUY_00397
Protein Complex 108	MUY_01518	MUY_00199	MUY_03287
Protein Complex 109	MUY_01535	MUY_01806	MUY_01938	MUY_02353	MUY_02808	MUY_02815	MUY_03543	MUY_04049	MUY_00810	MUY_00869
Protein Complex 110	MUY_01538	MUY_01539	MUY_01562	MUY_01845	MUY_02566
Protein Complex 111	MUY_00144	MUY_01598	MUY_02688	MUY_00833
Protein Complex 112	MUY_01575	MUY_01632	MUY_01669	MUY_03777	MUY_00042	MUY_00534
Protein Complex 113	MUY_01596	MUY_01641	MUY_01642	MUY_02043	MUY_02051	MUY_02077	MUY_02333	MUY_02526	MUY_02896	MUY_04163	MUY_04439	MUY_00447	MUY_00856	MUY_00917	MUY_00945
Protein Complex 114	MUY_01597	MUY_02320	MUY_03670	MUY_03686
Protein Complex 115	MUY_01603	MUY_04157	MUY_04346	MUY_00789
Protein Complex 116	MUY_01604	MUY_00176	MUY_00240	MUY_04156	MUY_04345	MUY_04361	MUY_04485	MUY_00787	MUY_00838	MUY_00886
Protein Complex 117	MUY_01612	MUY_02141	MUY_02634	MUY_04436
Protein Complex 118	MUY_00158	MUY_02300	MUY_03044
Protein Complex 119	MUY_01647	MUY_01929	MUY_03229
Protein Complex 120	MUY_00161	MUY_03067	MUY_03828	MUY_04502
Protein Complex 121	MUY_01672	MUY_01780	MUY_02286	MUY_04131	MUY_00754
Protein Complex 122	MUY_01385	MUY_01680	MUY_01681	MUY_02065	MUY_02649	MUY_02650	MUY_02669	MUY_03190	MUY_04476	MUY_00472	MUY_00473	MUY_00610	MUY_00893	MUY_00894
Protein Complex 123	MUY_01684	MUY_01992	MUY_03149	MUY_03246	MUY_03791	MUY_04098	MUY_04304
Protein Complex 124	MUY_01685	MUY_01991	MUY_03150	MUY_03247	MUY_03790	MUY_04099	MUY_04305	MUY_00630
Protein Complex 125	MUY_01713	MUY_01749	MUY_02783	MUY_00303	MUY_03505	MUY_03901	MUY_04182
Protein Complex 126	MUY_01744	MUY_03076	MUY_03274	MUY_03974	MUY_00525
Protein Complex 127	MUY_01764	MUY_00215	MUY_02339	MUY_02984	MUY_02993	MUY_00792
Protein Complex 128	MUY_01767	MUY_03404	MUY_00532	MUY_00768
Protein Complex 129	MUY_01772	MUY_03077	MUY_00043
Protein Complex 130	MUY_01792	MUY_01794	MUY_00041	MUY_00463	MUY_00064
Protein Complex 131	MUY_01808	MUY_03041	MUY_00310	MUY_00366	MUY_00582	MUY_00584	MUY_00648	MUY_00072
Protein Complex 132	MUY_00011	MUY_02531	MUY_02563	MUY_00253	MUY_03605
Protein Complex 133	MUY_01836	MUY_01947	MUY_02489	MUY_02793	MUY_03058	MUY_03089	MUY_04497	MUY_04508	MUY_00804	MUY_00963
Protein Complex 134	MUY_01840	MUY_02154	MUY_02169	MUY_03036	MUY_00344	MUY_03963	MUY_04024	MUY_04324	MUY_04482	MUY_00839
Protein Complex 135	MUY_01668	MUY_01843	MUY_02259	MUY_02424	MUY_02463	MUY_02510	MUY_02736	MUY_02765	MUY_02812	MUY_02869	MUY_03012	MUY_03095	MUY_03586	MUY_03863	MUY_03904	MUY_03922	MUY_04367	MUY_00496	MUY_00046	MUY_00527	MUY_00008	MUY_00816
Protein Complex 136	MUY_01844	MUY_04507	MUY_00896
Protein Complex 137	MUY_00177	MUY_00178	MUY_02935	MUY_03680	MUY_00900
Protein Complex 138	MUY_01733	MUY_01850	MUY_01860	MUY_00342	MUY_04011
Protein Complex 139	MUY_01856	MUY_01871	MUY_03907	MUY_03908	MUY_03909	MUY_03911
Protein Complex 140	MUY_00180	MUY_04112	MUY_00400	MUY_00559
Protein Complex 141	MUY_01889	MUY_00014	MUY_03020	MUY_03871	MUY_03872
Protein Complex 142	MUY_01897	MUY_01906	MUY_01932	MUY_03123
Protein Complex 143	MUY_01914	MUY_00257	MUY_03633	MUY_03837
Protein Complex 144	MUY_01916	MUY_02803	MUY_03742	MUY_03844	MUY_04025
Protein Complex 145	MUY_01949	MUY_02047	MUY_04028
Protein Complex 146	MUY_01979	MUY_02497	MUY_00020
Protein Complex 147	MUY_01988	MUY_02568	MUY_02758	MUY_04263	MUY_00946
Protein Complex 148	MUY_00191	MUY_02245	MUY_03066	MUY_00449	MUY_00828
Protein Complex 149	MUY_02096	MUY_03169	MUY_03694
Protein Complex 150	MUY_02108	MUY_02214	MUY_02215	MUY_02268	MUY_02460	MUY_04052	MUY_00460
Protein Complex 151	MUY_01904	MUY_02140	MUY_02305	MUY_00258	MUY_03400	MUY_00347	MUY_04132	MUY_04150	MUY_04397	MUY_04438	MUY_00453	MUY_00797
Protein Complex 152	MUY_02142	MUY_02183	MUY_03137	MUY_03138	MUY_03590	MUY_04103	MUY_04104	MUY_00519
Protein Complex 153	MUY_02177	MUY_02639	MUY_03213	MUY_03214
Protein Complex 154	MUY_02184	MUY_02301	MUY_03704	MUY_03831	MUY_00360	MUY_00911
Protein Complex 155	MUY_02185	MUY_02332	MUY_02937	MUY_02972	MUY_03839	MUY_00560	MUY_00855	MUY_00904
Protein Complex 156	MUY_03819	MUY_03821	MUY_03824	MUY_03931	MUY_04152	MUY_00927
Protein Complex 157	MUY_02212	MUY_02296	MUY_00678
Protein Complex 158	MUY_02227	MUY_03424	MUY_03426
Protein Complex 159	MUY_01826	MUY_01828	MUY_02228	MUY_03157	MUY_03277
Protein Complex 160	MUY_02253	MUY_02394	MUY_02449	MUY_02740	MUY_03344	MUY_03381	MUY_03823	MUY_03936	MUY_03949	MUY_00849
Protein Complex 161	MUY_02257	MUY_02586	MUY_03519
Protein Complex 162	MUY_02285	MUY_03113	MUY_03114
Protein Complex 163	MUY_02318	MUY_04053	MUY_04054	MUY_00071
Protein Complex 164	MUY_02342	MUY_02564	MUY_02992
Protein Complex 165	MUY_03098	MUY_03205	MUY_03897
Protein Complex 166	MUY_00230	MUY_03446	MUY_03632	MUY_03836	MUY_00872
Protein Complex 167	MUY_02436	MUY_00875	MUY_00931
Protein Complex 168	MUY_02437	MUY_03191	MUY_00609
Protein Complex 169	MUY_02472	MUY_03561	MUY_03562	MUY_04373
Protein Complex 170	MUY_01267	MUY_01781	MUY_02473	MUY_03866	MUY_03868	MUY_04037	MUY_04100	MUY_00730	MUY_00080	MUY_00081
Protein Complex 171	MUY_02479	MUY_02670	MUY_03480	MUY_00061
Protein Complex 172	MUY_02514	MUY_03100	MUY_03536	MUY_03833	MUY_03850	MUY_04207
Protein Complex 173	MUY_02517	MUY_03927	MUY_03942	MUY_03968	MUY_00492
Protein Complex 174	MUY_02561	MUY_02562	MUY_02722	MUY_03848	MUY_04492	MUY_00535	MUY_00536	MUY_00537	MUY_00539	MUY_00540	MUY_00542	MUY_00070
Protein Complex 175	MUY_01890	MUY_00003	MUY_02602	MUY_02635	MUY_03200
Protein Complex 176	MUY_02637	MUY_02642	MUY_02680	MUY_04060	MUY_04061	MUY_04062	MUY_04063	MUY_04064	MUY_04065	MUY_04066	MUY_04067	MUY_04510
Protein Complex 177	MUY_02647	MUY_03289	MUY_00784
Protein Complex 178	MUY_02764	MUY_03692	MUY_04193	MUY_04389	MUY_04390	MUY_04393
Protein Complex 179	MUY_02870	MUY_04004	MUY_04495
Protein Complex 180	MUY_02959	MUY_03842	MUY_04155	MUY_04326
Protein Complex 181	MUY_03026	MUY_03220	MUY_04017
Protein Complex 182	MUY_03053	MUY_03130	MUY_03131
Protein Complex 183	MUY_03070	MUY_03929	MUY_03950	MUY_03952	MUY_03953
Protein Complex 184	MUY_03102	MUY_03376	MUY_03983	MUY_04394	MUY_00501
Protein Complex 185	MUY_03144	MUY_03216	MUY_03249	MUY_03989
Protein Complex 186	MUY_00099	MUY_02502	MUY_00298	MUY_03702	MUY_04290	MUY_00774	MUY_00775
Protein Complex 187	MUY_03168	MUY_03399	MUY_04405
Protein Complex 188	MUY_03196	MUY_00907	MUY_00908
Protein Complex 189	MUY_03199	MUY_04007	MUY_04401
Protein Complex 190	MUY_02671	MUY_03227	MUY_03425	MUY_03613	MUY_04289	MUY_00507	MUY_00738
Protein Complex 191	MUY_03258	MUY_03986	MUY_04376	MUY_00495
Protein Complex 192	MUY_00327	MUY_00328	MUY_00329
Protein Complex 193	MUY_03288	MUY_03384	MUY_03385
Protein Complex 194	MUY_03364	MUY_03849	MUY_00888
Protein Complex 195	MUY_00318	MUY_03802	MUY_04161	MUY_00688	MUY_00971
Protein Complex 196	MUY_03398	MUY_03639	MUY_00830
Protein Complex 197	MUY_03423	MUY_03689	MUY_03794	MUY_04260	MUY_04262	MUY_04342	MUY_00892
Protein Complex 198	MUY_03469	MUY_03470	MUY_03471	MUY_03472	MUY_03473	MUY_03474	MUY_03475	MUY_00890
Protein Complex 199	MUY_01279	MUY_03815	MUY_04081	MUY_00845	MUY_00851
Protein Complex 200	MUY_04134	MUY_04219	MUY_00480

Protein Complex 201	MUY_00580	MUY_00740	MUY_00741	MUY_00742	MUY_00744																						
Protein Complex 202	MUY_01017	MUY_01049	MUY_01075	MUY_01244	MUY_01609	MUY_01693	MUY_01735	MUY_02030	MUY_02361	MUY_02588	MUY_00297	MUY_03518	MUY_03667	MUY_04463	MUY_00583	MUY_00674											
Protein Complex 203	MUY_01027	MUY_01163	MUY_01682	MUY_01683	MUY_02127	MUY_02160	MUY_02171	MUY_02201	MUY_02269	MUY_02619	MUY_02648	MUY_02841	MUY_02941	MUY_03136	MUY_03279	MUY_03513	MUY_03515	MUY_03524	MUY_03585	MUY_03840	MUY_04073	MUY_00459	MUY_00461	MUY_00523	MUY_00861		
Protein Complex 204	MUY_01030	MUY_01152	MUY_02707	MUY_02708	MUY_02882	MUY_02883	MUY_03778	MUY_03830	MUY_04185	MUY_04271	MUY_04291	MUY_04501	MUY_04505	MUY_00602	MUY_00691	MUY_00795	MUY_00086	MUY_00973									
Protein Complex 205	MUY_01047	MUY_01437	MUY_02240	MUY_04043	MUY_00385	MUY_04343	MUY_00722																				
Protein Complex 206	MUY_01114	MUY_01141	MUY_01415	MUY_01660	MUY_01740	MUY_01741	MUY_01750	MUY_01825	MUY_02198	MUY_00255	MUY_00256	MUY_02780	MUY_02797	MUY_03683	MUY_03967	MUY_04086	MUY_04334	MUY_00435	MUY_00483	MUY_00514	MUY_00589	MUY_00590	MUY_00068	MUY_00780			
Protein Complex 207	MUY_01052	MUY_00105	MUY_00114	MUY_00115	MUY_01504	MUY_01696	MUY_01760	MUY_01761	MUY_01879	MUY_00181	MUY_00182	MUY_02516	MUY_02560	MUY_02784	MUY_02845	MUY_02889	MUY_02900	MUY_03708	MUY_03918	MUY_04026	MUY_04258	MUY_04322	MUY_04504	MUY_00541	MUY_00571	MUY_00628	
Protein Complex 208	MUY_01053	MUY_01230	MUY_01523	MUY_03721	MUY_00512																						
Protein Complex 209	MUY_01059	MUY_01140	MUY_01374	MUY_01970	MUY_02036	MUY_02049	MUY_02231	MUY_02238	MUY_02580	MUY_02982	MUY_03140	MUY_03250	MUY_03431	MUY_00323	MUY_03553	MUY_03612	MUY_00445	MUY_00749	MUY_00779	MUY_00882							
Protein Complex 210	MUY_01061	MUY_01494	MUY_02015	MUY_00241	MUY_02947	MUY_00277	MUY_00337	MUY_00433	MUY_00813																		
Protein Complex 211	MUY_01076	MUY_00165	MUY_01907	MUY_04301	MUY_00698	MUY_00871																					
Protein Complex 212	MUY_01124	MUY_01482	MUY_01497	MUY_01528	MUY_01701	MUY_02050	MUY_02216	MUY_02232	MUY_02364	MUY_02695	MUY_00272	MUY_03126	MUY_03240	MUY_03659	MUY_03701	MUY_03880	MUY_04194	MUY_00556	MUY_00710								
Protein Complex 213	MUY_01128	MUY_01987	MUY_02916	MUY_03014	MUY_03054	MUY_03244	MUY_03556	MUY_03573	MUY_03574	MUY_03660	MUY_00766																
Protein Complex 214	MUY_01193	MUY_04235	MUY_00899																								
Protein Complex 215	MUY_01325	MUY_01326	MUY_01327	MUY_01646	MUY_01649	MUY_01650	MUY_00012	MUY_03017	MUY_04481																		
Protein Complex 216	MUY_01357	MUY_02207	MUY_04432	MUY_00423																							
Protein Complex 217	MUY_01366	MUY_01637	MUY_01915	MUY_02210	MUY_02616	MUY_02697	MUY_02725	MUY_02910	MUY_02917	MUY_02981	MUY_03284	MUY_03304	MUY_00316	MUY_03699	MUY_03706	MUY_04467	MUY_00750	MUY_00811	MUY_00824								
Protein Complex 218	MUY_02606	MUY_04085	MUY_04206	MUY_00695	MUY_00906																						
Protein Complex 219	MUY_00127	MUY_00132	MUY_00136	MUY_00139	MUY_00156	MUY_00157	MUY_01980	MUY_02805	MUY_02820	MUY_03062	MUY_03135	MUY_03774	MUY_04205	MUY_04244	MUY_04494	MUY_00794	MUY_00979	MUY_00998									
Protein Complex 220	MUY_01456	MUY_01730	MUY_01888	MUY_02338	MUY_02500	MUY_02665	MUY_00312	MUY_03608	MUY_03896	MUY_04466																	
Protein Complex 221	MUY_01471	MUY_03223	MUY_00326																								
Protein Complex 222	MUY_01539	MUY_01562	MUY_01845	MUY_02566																							
Protein Complex 223	MUY_01596	MUY_01641	MUY_01642	MUY_02043	MUY_02077	MUY_02333	MUY_02526	MUY_04163	MUY_04439	MUY_00447	MUY_00856	MUY_00917	MUY_00945														
Protein Complex 224	MUY_01604	MUY_00176	MUY_04156	MUY_04345	MUY_04361	MUY_04485	MUY_00787	MUY_00886																			
Protein Complex 225	MUY_01639	MUY_02355	MUY_02618	MUY_03173	MUY_03557																						
Protein Complex 226	MUY_01385	MUY_01680	MUY_01681	MUY_02065	MUY_02649	MUY_02650	MUY_02669	MUY_03190	MUY_04476	MUY_00472	MUY_00473	MUY_00610															
Protein Complex 227	MUY_01684	MUY_01992	MUY_03149	MUY_03246	MUY_03791	MUY_04098																					
Protein Complex 228	MUY_01685	MUY_01991	MUY_03247	MUY_03790	MUY_04099																						
Protein Complex 229	MUY_01702	MUY_03567	MUY_04127																								
Protein Complex 230	MUY_01836	MUY_01947	MUY_02489	MUY_02793	MUY_03058	MUY_03089	MUY_04497	MUY_04508	MUY_00804																		
Protein Complex 231	MUY_01840	MUY_02169	MUY_00344	MUY_04024	MUY_04324	MUY_04482	MUY_00839																				
Protein Complex 232	MUY_01668	MUY_01843	MUY_02259	MUY_02424	MUY_02463	MUY_02510	MUY_02736	MUY_02765	MUY_02812	MUY_03012	MUY_03095	MUY_03586	MUY_03863	MUY_03904	MUY_03922	MUY_04367	MUY_00496	MUY_00046	MUY_00527	MUY_00008	MUY_00816						
Protein Complex 233	MUY_01890	MUY_00003	MUY_03200																								
Protein Complex 234	MUY_01914	MUY_03633	MUY_03837																								
Protein Complex 235	MUY_02185	MUY_02332	MUY_02972	MUY_03839	MUY_00855	MUY_00904																					
Protein Complex 236	MUY_02730	MUY_02977	MUY_03601	MUY_03792	MUY_04042	MUY_04408	MUY_00443																				
Protein Complex 237	MUY_01016	MUY_00200	MUY_02834	MUY_03186	MUY_03876	MUY_00708																					
Protein Complex 238	MUY_01027	MUY_01163	MUY_01682	MUY_01683	MUY_02127	MUY_02160	MUY_02171	MUY_02201	MUY_02269	MUY_02619	MUY_02648	MUY_02841	MUY_02941	MUY_03136	MUY_03232	MUY_03279	MUY_03513	MUY_03515	MUY_03524	MUY_03585	MUY_03840	MUY_04073	MUY_00459	MUY_00461	MUY_00523	MUY_00861	MUY_00895
Protein Complex 239	MUY_01030	MUY_01152	MUY_02708	MUY_02882	MUY_02883	MUY_03778	MUY_03830	MUY_04185	MUY_04271	MUY_04291	MUY_04501	MUY_04505	MUY_00599	MUY_00691	MUY_00795	MUY_00086	MUY_00973										
Protein Complex 240	MUY_01047	MUY_01437	MUY_02240	MUY_03069	MUY_03980	MUY_04043	MUY_00385	MUY_04343	MUY_00722																		
Protein Complex 241	MUY_01052	MUY_00105	MUY_00114	MUY_00115	MUY_01390	MUY_01504	MUY_01696	MUY_01760	MUY_01761	MUY_01879	MUY_00181	MUY_00182	MUY_02516	MUY_02560	MUY_02784	MUY_02845	MUY_02889	MUY_02900	MUY_03708	MUY_03918	MUY_04026	MUY_04258	MUY_04504	MUY_00541	MUY_00571	MUY_00628	
Protein Complex 242	MUY_01059	MUY_01140	MUY_01374	MUY_01970	MUY_01999	MUY_02036	MUY_02049	MUY_02231	MUY_02238	MUY_02580	MUY_02982	MUY_03140	MUY_03250	MUY_03431	MUY_00323	MUY_03553	MUY_03612	MUY_00445	MUY_00749	MUY_00779	MUY_00882						
Protein Complex 243	MUY_01139	MUY_01953	MUY_03567																								
Protein Complex 244	MUY_01161	MUY_01486	MUY_01727	MUY_02578	MUY_02830	MUY_03356	MUY_03860	MUY_03951	MUY_04473	MUY_00912																	
Protein Complex 245	MUY_01209	MUY_01321	MUY_01959	MUY_02025	MUY_02620	MUY_03341	MUY_03375	MUY_03451	MUY_03775	MUY_00526	MUY_00857																
Protein Complex 246	MUY_01226	MUY_01776	MUY_01786	MUY_01885	MUY_02202	MUY_02413	MUY_03025	MUY_03345	MUY_00053	MUY_00588	MUY_00057	MUY_00743	MUY_00773	MUY_00074													
Protein Complex 247	MUY_01233	MUY_03310	MUY_03422	MUY_03703	MUY_04079	MUY_04391																					
Protein Complex 248	MUY_01221	MUY_01291	MUY_03034	MUY_03260	MUY_04136	MUY_00709																					
Protein Complex 249	MUY_02210	MUY_02981	MUY_03699	MUY_00750	MUY_00811																						
Protein Complex 250	MUY_01366	MUY_01915	MUY_02616	MUY_02697	MUY_02725	MUY_02910	MUY_03284	MUY_03304	MUY_00316	MUY_03706	MUY_04467	MUY_00824															
Protein Complex 251	MUY_01438	MUY_01443	MUY_02371	MUY_04249	MUY_00481	MUY_00573																					
Protein Complex 252	MUY_01471	MUY_03223	MUY_00326	MUY_04247																							
Protein Complex 253	MUY_01604	MUY_00176	MUY_04156	MUY_04345	MUY_04361	MUY_04485	MUY_00787	MUY_00838	MUY_00886																		
Protein Complex 254	MUY_01680	MUY_01681	MUY_02065	MUY_02649	MUY_02650	MUY_02669	MUY_02671	MUY_03190	MUY_04476	MUY_00472	MUY_00473	MUY_00610															
Protein Complex 255	MUY_02185	MUY_02332	MUY_02972	MUY_03839	MUY_00560	MUY_00855	MUY_00904																				
Protein Complex 256	MUY_02253	MUY_02394	MUY_02449	MUY_02740	MUY_03381	MUY_03823	MUY_03936	MUY_03949																			
Protein Complex 257	MUY_03227	MUY_03425	MUY_03613	MUY_04289	MUY_00507	MUY_00738																					
Protein Complex 258	MUY_03258	MUY_03986	MUY_04376																								
Protein Complex 259	MUY_03469	MUY_03470	MUY_03471	MUY_03472	MUY_03473	MUY_03474	MUY_03475																				
Protein Complex 260	MUY_03815	MUY_04081	MUY_00845																								
Protein Complex 261	MUY_01019	MUY_01088	MUY_01089	MUY_01095	MUY_01113	MUY_01130	MUY_00110	MUY_01283	MUY_01284	MUY_01297	MUY_01298	MUY_01461															
	MUY_01466	MUY_00134	MUY_00152	MUY_00153	MUY_01652	MUY_01653	MUY_01656	MUY_01664	MUY_01762	MUY_00189	MUY_00194	MUY_02151	MUY_02208														
	MUY_02209	MUY_02225	MUY_02597	MUY_02598	MUY_02609	MUY_02643	MUY_02700	MUY_02744	MUY_02745	MUY_02760	MUY_00265	MUY_02894	MUY_02929														
	MUY_02945	MUY_02946	MUY_00285	MUY_03154	MUY_03330	MUY_03332	MUY_03354	MUY_00322	MUY_03575	MUY_03576	MUY_03582	MUY_03598	MUY_03634														
	MUY_03649	MUY_03676	MUY_03785	MUY_03805	MUY_03900	MUY_03946	MUY_00372	MUY_03978	MUY_04144	MUY_04172	MUY_04248	MUY_04254	MUY_04266														
	MUY_04269	MUY_04282	MUY_04296	MUY_00388	MUY_04348	MUY_04357	MUY_00394	MUY_04403	MUY_04416	MUY_04445	MUY_04453	MUY_00420	MUY_00434														
	MUY_00442	MUY_00516	MUY_00585	MUY_00800	MUY_00815	MUY_00937	MUY_00938	MUY_00939	MUY_00968	MUY_00975	MUY_00996	MUY_01000															
Protein Complex 262	MUY_01032	MUY_01108	MUY_01109	MUY_01203	MUY_01308	MUY_01431	MUY_01432	MUY_01527	MUY_01582	MUY_01626	MUY_01670	MUY_01874	MUY_01875														
	MUY_00205	MUY_02178	MUY_02366	MUY_00229	MUY_00231	MUY_02520	MUY_02521	MUY_02523	MUY_00249	MUY_02655	MUY_02663	MUY_00263	MUY_00264														
	MUY_02912	MUY_02939	MUY_02940	MUY_02964	MUY_00288	MUY_00289	MUY_03059	MUY_03117	MUY_03127	MUY_03139	MUY_03183	MUY_03201	MUY_03202														
	MUY_03252	MUY_03306	MUY_03374	MUY_03463	MUY_03477	MUY_03478	MUY_03483	MUY_03609	MUY_03610	MUY_03619	MUY_03620	MUY_03647	MUY_03648														
	MUY_03924	MUY_04096	MUY_04146	MUY_04177	MUY_04211	MUY_04212	MUY_04293	MUY_04294	MUY_04358	MUY_04359	MUY_00393	MUY_04379	MUY_04417														
	MUY_04418	MUY_04420	MUY_04447	MUY_04448	MUY_04470	MUY_04471	MUY_00418	MUY_00436	MUY_00438	MUY_00575	MUY_00576	MUY_00790	MUY_00961														
Protein Complex 263	MUY_01019	MUY_01088	MUY_01089	MUY_01095	MUY_01113	MUY_01130	MUY_00110	MUY_01283	MUY_01284	MUY_01297	MUY_01298	MUY_01461	MUY_01466														
	MUY_00134	MUY_00152	MUY_00153	MUY_01652	MUY_01653	MUY_01656	MUY_01664	MUY_01762	MUY_00189	MUY_00194	MUY_02151	MUY_02208	MUY_02209														
	MUY_02225	MUY_02597	MUY_02598	MUY_02609	MUY_02643	MUY_02700	MUY_02744	MUY_02745	MUY_02760	MUY_00265	MUY_02929	MUY_02945	MUY_02946														
	MUY_00285	MUY_03330	MUY_03332	MUY_03354	MUY_03575	MUY_03576	MUY_03582	MUY_03598	MUY_03634	MUY_03649	MUY_03676	MUY_03785	MUY_03805														
	MUY_03900	MUY_03946	MUY_00372	MUY_03978	MUY_04144	MUY_04172	MUY_04248	MUY_04254	MUY_04266	MUY_04269	MUY_04282	MUY_04296	MUY_00388														
	MUY_04348	MUY_04357	MUY_00394	MUY_04403	MUY_04416	MUY_04445	MUY_04453	MUY_00420	MUY_00434	MUY_00442	MUY_00516	MUY_00585	MUY_00800														
	MUY_00815	MUY_00937	MUY_00938	MUY_00939	MUY_00968	MUY_00975	MUY_00996	MUY_01000																			
Protein Complex 264	MUY_01032	MUY_01108	MUY_01109	MUY_01203	MUY_01308	MUY_01431	MUY_01432	MUY_01527	MUY_01626	MUY_01670	MUY_01874	MUY_01875	MUY_00205														
	MUY_02178	MUY_00231	MUY_02520	MUY_02521	MUY_02523	MUY_00249	MUY_02655	MUY_02663	MUY_02912	MUY_02939	MUY_02940	MUY_02964	MUY_00288														
	MUY_00289	MUY_03059	MUY_03117	MUY_03127	MUY_03139	MUY_03183	MUY_03201	MUY_03202	MUY_03306	MUY_03374	MUY_03463	MUY_03477	MUY_03478														
	MUY_03483	MUY_03609	MUY_03610	MUY_03619	MUY_03620	MUY_03647	MUY_03648	MUY_03924	MUY_04096	MUY_04146	MUY_04177	MUY_04211	MUY_04212														
	MUY_04293	MUY_04294	MUY_04358	MUY_04359	MUY_00393	MUY_04417	MUY_04418	MUY_04447	MUY_04448	MUY_04470	MUY_04471	MUY_00418	MUY_00436														
	MUY_00438	MUY_00575	MUY_00576	MUY_00790	MUY_00961																						
Protein Complex 265	MUY_01048	MUY_01237	MUY_01418	MUY_01520	MUY_01616	MUY_01919	MUY_01937	MUY_01989	MUY_00190	MUY_02094	MUY_02152	MUY_02439	MUY_02467														
	MUY_02627	MUY_00261	MUY_00308	MUY_03333	MUY_03386	MUY_03449	MUY_03454	MUY_03637	MUY_03810	MUY_04070	MUY_04242	MUY_00382	MUY_04383														
	MUY_04433	MUY_04483	MUY_00450	MUY_00464	MUY_00808	MUY_00840																					
Protein Complex 266	MUY_01032	MUY_01108	MUY_01109	MUY_01203	MUY_01308	MUY_01431	MUY_01432	MUY_01527	MUY_01582	MUY_01626	MUY_01670	MUY_01874	MUY_01875														
	MUY_00205	MUY_02178	MUY_02366	MUY_00231	MUY_02520	MUY_02521	MUY_02523	MUY_00249	MUY_02655	MUY_02663	MUY_00263	MUY_00264	MUY_02912														
	MUY_02939	MUY_02940	MUY_02964	MUY_00288	MUY_00289	MUY_03059	MUY_03117	MUY_03127	MUY_03139	MUY_03183	MUY_03201	MUY_03202	MUY_03306														
	MUY_03374	MUY_03463	MUY_03477	MUY_03478	MUY_03483	MUY_03609	MUY_03610	MUY_03619	MUY_03620	MUY_03647	MUY_03648	MUY_03924	MUY_03925														
	MUY_04096	MUY_04145	MUY_04146	MUY_04177	MUY_04211	MUY_04212	MUY_04293	MUY_04294	MUY_04358	MUY_04359	MUY_00393	MUY_04417	MUY_04418														
	MUY_04420	MUY_04447	MUY_04448	MUY_04470	MUY_04471	MUY_00418	MUY_00436	MUY_00438	MUY_00575	MUY_00576	MUY_00790	MUY_00961															
Protein Complex 267	MUY_01061	MUY_01114	MUY_01136	MUY_01141	MUY_01494	MUY_01510	MUY_01660	MUY_01740	MUY_01741	MUY_01742	MUY_01750	MUY_02015	MUY_02198														
	MUY_02434	MUY_00241	MUY_00251	MUY_00255	MUY_02780	MUY_02797	MUY_02947	MUY_00277	MUY_02996	MUY_03459	MUY_03496	MUY_00337	MUY_03683														
	MUY_04086	MUY_04116	MUY_04334	MUY_00433	MUY_00483	MUY_00514	MUY_00589	MUY_00590	MUY_00068	MUY_00780	MUY_00813																

Supplementary Table S4. Functional annotation for poorly uncharacterized proteins

Proteins with unknown function	COG functional catogories
MUY_00021	Cell wall/membrane/envelope biogenesis (**)
MUY_00041	Coenzyme transport and metabolism (***)
MUY_00064	Coenzyme transport and metabolism (***)
MUY_00072	Posttranslational modification, protein turnover, chaperones (*)
MUY_00178	Transcription (*)
MUY_00243	Cell wall/membrane/envelope biogenesis (**)
MUY_00275	Posttranslational modification, protein turnover, chaperones (***)
MUY_00320	Cell wall/membrane/envelope biogenesis (**)
MUY_00360	Lipid transport and metabolism (*)
MUY_00366	Posttranslational modification, protein turnover, chaperones (*)
MUY_00514	Cell wall/membrane/envelope biogenesis (*) Intracellular trafficking, secretion, and vesicular transport (***) Cell cycle control, cell divesion, chromosome partitioning (*)
MUY_00557	Transcription (***)
MUY_00583	Transcription (***)
MUY_00585	Defense mechanisms (***) Inorganic ion transport and metabolism (***) Amino acid transport and metabolism (**)
MUY_00588	Nucleotide transport and metabolism (***)
MUY_00602	Transcription (***)
MUY_00628	Transcription (***)
MUY_00630	Signal transduction mechanisms (***)
MUY_00674	Transcription (***)
MUY_00691	Transcription (***)
MUY_00695	Amino acid transport and metabolism (**)
MUY_00790	Signal transduction mechanisms (***) Cell motility (***)
MUY_00800	Defense mechanisms (***) Inorganic ion transport and metabolism (***) Amino acid transport and metabolism (**)
MUY_00815	Defense mechanisms (***) Inorganic ion transport and metabolism (***) Amino acid transport and metabolism (**)
MUY_00824	Inorganic ion transport and metabolism (**)
MUY_00869	Coenzyme transport and metabolism (***)
MUY_00906	Amino acid transport and metabolism (**)
MUY_00911	Lipid transport and metabolism (*)
MUY_00921	Cell wall/membrane/envelope biogenesis (***)
MUY_00935	Secondary metabolites biosynthesis, transport and catabolism (***)
MUY_00961	Signal transduction mechanisms (***) Cell motility (***)
MUY_01015	Amino acid transport and metabolism (*)
MUY_01017	Transcription (***)
MUY_01019	Defense mechanisms (***) Inorganic ion transport and metabolism (***) Amino acid transport and metabolism (**)
MUY_01038	Secondary metabolites biosynthesis, transport and catabolism (***)
MUY_01060	Amino acid transport and metabolism (*)
MUY_01062	Amino acid transport and metabolism (*)
MUY_01075	Transcription (***)
MUY_01109	Signal transduction mechanisms (***) Cell motility (***)
MUY_01113	Defense mechanisms (***) Inorganic ion transport and metabolism (***) Amino acid transport and metabolism (**)
MUY_01141	Cell wall/membrane/envelope biogenesis (*) Intracellular trafficking, secretion, and vesicular transport (***) Cell cycle control, cell divesion, chromosome partitioning (*)
MUY_01176	Carbohydrate transport and metabolism (***)
MUY_01195	Inorganic ion transport and metabolism (**)
MUY_01372	Cell wall/membrane/envelope biogenesis (**)
MUY_01406	Cell wall/membrane/envelope biogenesis (**)
MUY_01415	Cell wall/membrane/envelope biogenesis (*) Intracellular trafficking, secretion, and vesicular transport (***) Cell cycle control, cell divesion, chromosome partitioning (*)
MUY_01419	Amino acid transport and metabolism (*)
MUY_01523	Carbohydrate transport and metabolism (**)
MUY_01575	Transcription (*)
MUY_01582	Signal transduction mechanisms (***) Cell motility (***)
MUY_01623	Cell wall/membrane/envelope biogenesis (**)
MUY_01632	Transcription (*)
MUY_01639	Amino acid transport and metabolism (**)
MUY_01664	Defense mechanisms (***) Inorganic ion transport and metabolism (***) Amino acid transport and metabolism (**)
MUY_01768	Cell cycle control, cell divesion, chromosome partitioning (***)
MUY_01806	Coenzyme transport and metabolism (***)

MUY_01808	Posttranslational modification, protein turnover, chaperones (*)
MUY_01809	Inorganic ion transport and metabolism (***)
MUY_01857	Amino acid transport and metabolism (*)
MUY_01909	Translation, ribosomal structure and biogenesis (*)
MUY_01915	Inorganic ion transport and metabolism (**)
MUY_01932	Translation, ribosomal structure and biogenesis (*)
MUY_01969	Cell wall/membrane/envelope biogenesis (**)
MUY_01980	Translation, ribosomal structure and biogenesis (***)
MUY_02030	Transcription (***)
MUY_02062	Cell wall/membrane/envelope biogenesis (**)
MUY_02132	Amino acid transport and metabolism (*)
MUY_02141	Carbohydrate transport and metabolism (*)
MUY_02184	Lipid transport and metabolism (*)
MUY_02225	Defense mechanisms (***) Inorganic ion transport and metabolism (***) Amino acid transport and metabolism (**)
MUY_02228	Intracellular trafficking, secretion, and vesicular transport (***)
MUY_02258	Cell wall/membrane/envelope biogenesis (**)
MUY_02268	Energy production and conversion (***)
MUY_02269	Energy production and conversion (***)
MUY_02424	Replication, recombination and repair (***)
MUY_02508	Cell wall/membrane/envelope biogenesis (**)
MUY_02588	Transcription (***)
MUY_02602	Replication, recombination and repair (***)
MUY_02615	Lipid transport and metabolism (***)
MUY_02616	Inorganic ion transport and metabolism (**)
MUY_02642	Energy production and conversion (***)
MUY_02680	Energy production and conversion (***)
MUY_02725	Inorganic ion transport and metabolism (**)
MUY_02780	Cell wall/membrane/envelope biogenesis (*) Intracellular trafficking, secretion, and vesicular transport (***) Cell cycle control, cell divesion, chromosome partitioning (*)
MUY_02788	Transcription (***)
MUY_02797	Cell wall/membrane/envelope biogenesis (*) Intracellular trafficking, secretion, and vesicular transport (***) Cell cycle control, cell divesion, chromosome partitioning (*)
MUY_02841	Energy production and conversion (***) Posttranslational modification, protein turnover, chaperones (**)
MUY_02882	Transcription (***)
MUY_02910	Inorganic ion transport and metabolism (**)
MUY_02970	Energy production and conversion (**)
MUY_03010	Translation, ribosomal structure and biogenesis (*)
MUY_03041	Posttranslational modification, protein turnover, chaperones (*)
MUY_03055	Transcription (***)
MUY_03128	Lipid transport and metabolism (***)
MUY_03146	Cell cycle control, cell divesion, chromosome partitioning (***)
MUY_03277	Intracellular trafficking, secretion, and vesicular transport (***)
MUY_03284	Inorganic ion transport and metabolism (**)
MUY_03342	Cell wall/membrane/envelope biogenesis (**)
MUY_03434	Amino acid transport and metabolism (*)
MUY_03496	Cell wall/membrane/envelope biogenesis (*) Intracellular trafficking, secretion, and vesicular transport (***) Cell cycle control, cell divesion, chromosome partitioning (*)
MUY_03658	Energy production and conversion (**)
MUY_03706	Inorganic ion transport and metabolism (**)
MUY_03769	Transcription (***)
MUY_03831	Lipid transport and metabolism (*)
MUY_03836	Energy production and conversion (***)
MUY_03863	Replication, recombination and repair (***)
MUY_03907	Cell motility (***)
MUY_04020	Amino acid transport and metabolism (*)
MUY_04033	Lipid transport and metabolism (***) Secondary metabolites biosynthesis, transport and catabolism (***)
MUY_04045	Lipid transport and metabolism (***)
MUY_04185	Transcription (***)
MUY_04205	Translation, ribosomal structure and biogenesis (***)
MUY_04244	Translation, ribosomal structure and biogenesis (***)
MUY_04348	Defense mechanisms (***) Inorganic ion transport and metabolism (***) Amino acid transport and metabolism (**)
MUY_04403	Defense mechanisms (***) Inorganic ion transport and metabolism (***) Amino acid transport and metabolism (**)

MUY_04410	Translation, ribosomal structure and biogenesis (***)
MUY_04467	Inorganic ion transport and metabolism (**)

P -values were calculated for each function (*P<0.05, **P<0.01, ***P<0.001)
